# Probiotic interventions maintain intestinal barrier function and alleviate necrotizing enterocolitis by inhibiting ferroptosis in intestinal PMN-MDSCs

**DOI:** 10.1038/s41419-026-08869-w

**Published:** 2026-05-15

**Authors:** Meiqi Chen, Qing Zhao, Laiqin Peng, Ziyang Chen, Shuaijun Lv, Zekai Zhuang, Shudan Zheng, Jiaxiu Ye, Junyu He, Yizhuang Lu, Gang Xiao, Yuxiong Guo, Yumei He

**Affiliations:** 1 Pediatric Intensive Care Unit, Guangdong Provincial People’s Hospital (Guangdong Academy of Medical Sciences); Department of Immunology, School of Basic Medical Sciences; Department of Clinical Laboratory, the Third Affiliated Hospital of Southern Medical University, Southern Medical University, Guangzhou, China; 2 Department of Immunology, School of Basic Medical Sciences; Guangdong Provincial Key Laboratory of Single-cell and Extracellular Vesicles; Southern Medical University, Guangzhou, China; 3 Department of Gynecology and Obstetrics, Huizhou Central People’s Hospital, Huizhou, China; 4 Department of Neurosurgery, Guangdong Sanjiu Brain Hospital, Guangzhou, China; 5 Pediatric Intensive Care Unit, Guangdong Provincial People’s Hospital (Guangdong Academy of Medical Sciences), Southern Medical University; Guangdong Provincial Cardiovascular Institute, Guangdong Provincial People’s Hospital, Guangdong Academy of Medical Sciences, Guangzhou, China; 6 Department of Clinical Laboratory, the Third Affiliated Hospital of Southern Medical University, Southern Medical University, Guangzhou, China

**Keywords:** Neutrophils, Immune cell death, Inflammatory bowel disease

## Abstract

Circulating polymorphonuclear myeloid-derived suppressor cells (PMN-MDSCs) and gut probiotics are crucial for alleviating experimental necrotizing enterocolitis (NEC) in mice, yet the mechanisms linking intestinal PMN-MDSCs (iPMN-MDSCs) to specific microbiota remain unclear. Herein, we identified *Lactobacillus* (*L*.) *reuteri* and *L. rhamnosus* as two key strains significantly reduced under NEC conditions; their combined supplementation increased iPMN-MDSC abundance and olfactomedin 4 (OLFM4) expression, thereby improving intestinal epithelial cell (IEC) function and attenuating NEC. *Olfm4* deficiency in neutrophils exacerbated NEC, disrupted intestinal barrier integrity, and induced microbial dysbiosis. Mechanistically, OLFM4 inhibited iPMN-MDSC ferroptosis by enhancing activating transcription factor 4 (ATF4) activity and upregulating its targets solute carrier family 7a member 11 (Slc7a11) and glutathione peroxidase 4 (Gpx4). Downregulation of Atf4 or Gpx4 recapitulated the phenotypic alterations observed in *Olfm4*-deficient mice, including aggravated NEC and impaired iPMN-MDSC function. Treatment with indole-3-aldehyde, an effector metabolite of probiotics, alleviated NEC by restoring the OLFM4-driven anti-ferroptosis axis in iPMN-MDSCs. In patients with NEC, reduced intestinal LOX1^+^PMN-MDSCs and a weakened anti-ferroptosis pathway were associated with disease progression. These findings offer a therapeutic strategy for NEC by targeting iPMN-MDSC ferroptosis via probiotic- or metabolite-based interventions.

## Introduction

The early-life establishment of gut microbiota is fundamental to infant health, particularly in preterm newborns [1, 2]. Dysregulated gut microbial composition and diversity, along with aberrant microbial colonization, are strongly associated with serious neonatal diseases, most notably necrotizing enterocolitis (NEC) [3]. NEC is a life-threatening gastrointestinal emergency featuring rapid-onset intestinal ischemia and necrosis, with risks of progression to sepsis, peritonitis, or perforation [4, 5]. Although the mortality rate of fulminant NEC approaches 80% within 48 h of diagnosis [6], specific and effective treatment options remain limited. Probiotic interventions beneficially reshape intestinal microbial succession, maintain barrier integrity, and alleviate NEC [7–9]; however, the precise molecular mechanisms underlying these effects remain unclear.

The etiology of NEC is multifactorial and incompletely understood. Recent evidence implicates dysregulated cell death pathways, especially ferroptosis, in intestinal barrier dysfunction and excessive inflammatory responses in NEC [10]. Ferroptosis is an iron-dependent form of regulated cell death characterized by the lethal accumulation of peroxidized phospholipids and reactive oxygen species (ROS) [11], leading to mitochondrial damage, membrane disruption, and cell death [12]. This process underlies various human diseases, including cancer [13], ulcerative colitis [14], rheumatoid arthritis [15], and ischemia/reperfusion injury [16]. Premature infants are particularly vulnerable to such oxidative damage owing to their immature antioxidative defense systems [17]. In the context of NEC, enhanced ferroptosis in regulatory T cells (Tregs) and intestinal epithelial cells (IECs) exacerbates intestinal tissue injury and amplifies the inflammatory response in neonates [18, 19]. The xCT (solute carrier family 7a member 11, SLC7A11)/glutathione peroxidase 4 (GPX4) axis constitutes a crucial antioxidant system for ferroptosis [20]. While the relationship between ferroptosis and NEC pathogenesis has been proposed, the precise mechanisms linking the antioxidant pathways to NEC development remain unclear.

Polymorphonuclear myeloid-derived suppressor cells (PMN-MDSCs) regulate neonatal anti-inflammatory responses and immunosuppressive activity [21–24]. We previously reported that impaired maternal PMN-MDSCs increase offspring susceptibility to NEC [25], and the adoptive transfer of neonatal splenic PMN-MDSCs attenuates NEC severity and improves survival [23, 26]. While most existing studies have focused on circulating PMN-MDSCs, intestinal PMN-MDSCs (iPMN-MDSCs) remain understudied. Crucially, intestinal immune cells seem to be regulated by gut microbiota-derived signals [27, 28]; for instance, specific *Lactobacillus* species sustain RORγt^+^ Treg cell-mediated immunotolerance [29], and *Bifidobacterial* abundance influences intestinal type 17 helper T (Th17) cell levels [30]. Nevertheless, the interaction between the gut microbiota and iPMN-MDSCs remains largely unexplored. Further, given that ferroptosis critically regulates MDSC survival and function [31, 32], whether and how the microbiota modulates iPMN-MDSC homeostasis through ferroptosis remains unclear.

A pathological hallmark of NEC is the breakdown of intestinal barrier integrity, characterized by disrupted tight junction (TJ) complexes and impaired IEC function, which facilitates the translocation of pathogens and foreign microorganisms [33, 34]. The restoration of this protective barrier is a crucial therapeutic target for NEC [35, 36], as evidenced by strategies targeting IEC regeneration or immune modulation [37–39]. However, despite the known roles of various immune cells in epithelial homeostasis, whether and how iPMN-MDSCs contribute to IEC function and intestinal barrier integrity remains unknown.

In this study, we identify an olfactomedin 4 (OLFM4)-driven anti-ferroptosis axis within iPMN-MDSCs, which is activated by the probiotic metabolite indole-3-aldehyde and is essential for barrier repair and NEC alleviation. This axis is impaired in infants with NEC, highlighting its clinical relevance. Collectively, our work establishes iPMN-MDSC ferroptosis as a pivotal mechanism linking the gut microbiota to intestinal homeostasis and proposes targeting this axis via metabolites as a therapeutic strategy for NEC.

## Results

### A probiotic combination of *L. reuteri* and *L. rhamnosus* alleviates NEC by promoting iPMN-MDSC accumulation and restoring IL-18-mediated intestinal barrier function

Given the critical role of the neonatal microbiome in intestinal homeostasis, we first assessed the impact of gut microbiota depletion on experimental NEC. Five-day-old neonates were treated with a broad-spectrum antibiotic for 48 h prior to NEC induction [40, 41] (Fig. S1A), and successful depletion of the gut microbiota was verified (Fig. S1B, C). Antibiotic-treated pups exhibited significantly more severe NEC phenotypes, characterized by decreased survival rate, exacerbated intestinal inflammation, and aggravated weight loss (Fig. S1D–G). These findings demonstrate that the gut microbiota plays a protective role in experimental NEC pathogenesis, with its depletion exacerbating disease severity.

**Fig. 1:**
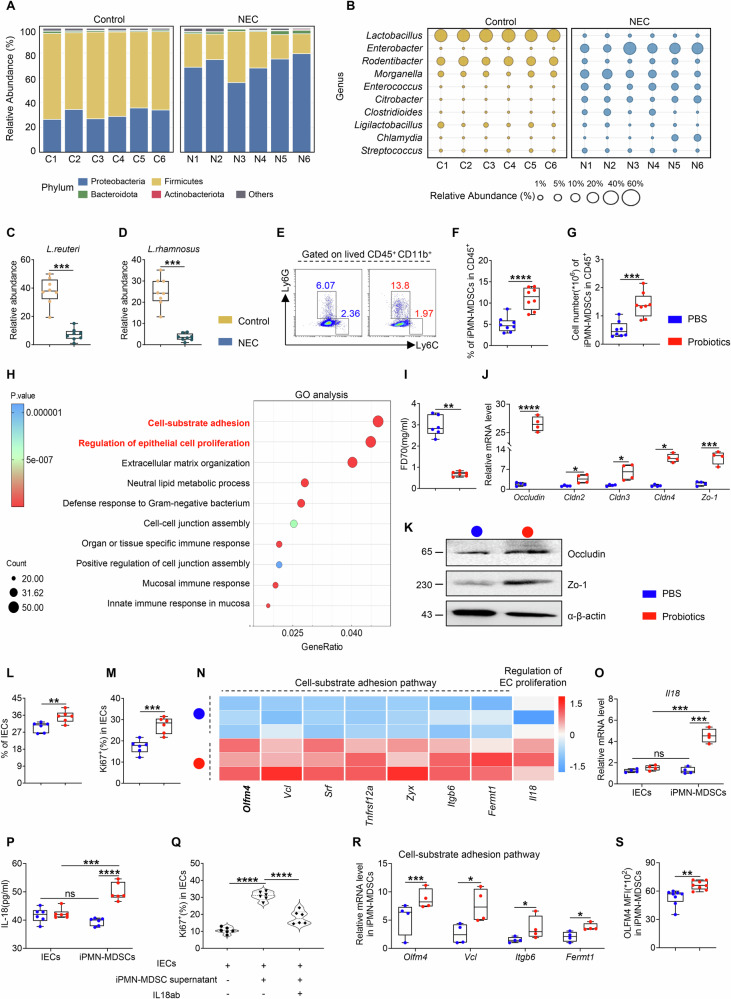
A probiotic combination of *L. reuteri* and *L. rhamnosus* alleviates NEC by promoting iPMN-MDSC accumulation and restoring intestinal barrier function. Gut microbial composition at the phylum (**A**) and genus (**B**) levels in control and NEC pups (*n* = 6). The relative abundance of *L*. *reuteri* (**C**) and *L. rhamnosus* (**D**) in fecal samples (*n* = 8). Representative flow cytometry plots (**E**) and statistical analysis of intestinal PMN-MDSCs (iPMN-MDSCs) among CD45^+^ cells (**F**, **G**, *n* = 8). **H** Gene Ontology analysis of differentially expressed genes in iPMN-MDSCs from PBS- or probiotic-treated pups. **I** Intestinal permeability was assessed by fluorescein isothiocyanate-dextran 70 (FD70) levels (*n* = 6). mRNA (**J**, *n* = 4 biological replicates) and protein (**K**, replicated three times) levels of tight junction (TJ) proteins in the intestine. Percentage (**L**) and proliferation (**M**) of intestinal epithelial cells (IECs) from the indicated treatment groups (*n* = 6). **N** Heatmap of upregulated genes within the top two enriched signaling pathways identified in H. **O** mRNA expression levels of *Il18* in IECs and iPMN-MDSCs (*n* = 4 biological replicates). **P** Secretion levels of IL-18 in IECs and iPMN-MDSCs (*n* = 6). **Q** Proliferation of IECs in the co-culture system (*n* = 6). **R** mRNA expression levels of *Olfm4*, *Vcl*, *Itgb6*, and *Fermt1* in iPMN-MDSCs (*n* = 4 biological replicates). **S** Mean fluorescence intensity (MFI) of OLFM4 in iPMN-MDSCs (*n* = 8). Data are presented as mean ± SEM. Each symbol represents one pup in a litter. ns, not significant; *p < 0.05, **p < 0.01, ***p < 0.001, ****p < 0.0001. Statistical significance was determined using a Student’s *t* test (**C**, **F**, **G**, **J**, **L**, **M**, **O**–**S**) or Mann–Whitney test (**C**, **D**, **I**, **J**).

To identify the specific microbiota involved, we profiled the gut microbiota in NEC model mice using 16S ribosomal RNA (rRNA) gene sequencing. Principal coordinate analysis revealed a distinct microbial community composition in neonates with NEC, as evidenced by increased α-diversity (Fig. S2A–E). Notably, the genus *Lactobacillus* was significantly reduced (Fig. 1A, B). Quantitative real-time PCR (qPCR) analysis confirmed the specific decrease in *Lactobacillus* (*L*.) *reuteri* and *L. rhamnosus* (Fig. 1C, D). Given their known beneficial effects and the observed reduction, we hypothesized that restoring these species might alleviate NEC. Therefore, seven-day-old wild-type pups received vehicle control, *L. reuteri*, *L. rhamnosus*, or a combination of both strains during NEC induction (Fig. S3A). Notably, the combination of both strains significantly improved survival, reduced intestinal inflammation, and decreased weight loss compared to single administration (Fig. S3B–E). To determine whether this effect was direct, we employed a microbiota-depletion model (Fig. S3F). Antibiotic-pretreated pups that received the probiotic combination showed improved outcomes relative to those administered the PBS control (Fig. S3G–J). These data demonstrate that the combined treatment of *L. reuteri* and *L. rhamnosus* effectively mitigates NEC independently of the resident gut microbiota.

Given the established roles of MDSCs in neonatal inflammation and NEC [21–23, 26], we next assessed the effect of probiotic treatment on this population. Probiotic treatment specifically induced the accumulation of PMN-MDSCs in the intestine (iPMN-MDSCs), but not monocytic MDSCs (iM-MDSCs) (Figs. 1E–G and S4A, B). Moreover, no significant differences were observed in splenic MDSC subsets (Fig. S4C–F), suggesting a specific role for iPMN-MDSCs in mediating probiotic effects. RNA sequencing (RNA-seq) of sorted iPMN-MDSCs revealed upregulation of PMN-MDSC-related genes upon probiotic treatment, which was validated by qPCR (Fig. S4G, H). Gene Ontology analysis highlighted significant enrichment in pathways related to cell–substrate adhesion and the regulation of epithelial cell proliferation (Fig. 1H). Consistently, probiotic-treated neonates exhibited decreased intestinal permeability, increased TJ protein expression, and improved IEC abundance and function (Fig. 1I–M). Notably, a heatmap of upregulated genes related to epithelial cell (EC) proliferation featured *Il18* (Fig. 1N). Further examination confirmed that probiotic treatment specifically increased IL-18 expression and secretion in iPMN-MDSCs, but not in IECs (Fig. 1O, P).

To delineate the role of IL-18 and iPMN-MDSC-derived IL-18 in experimental NEC, three groups of neonates were subjected to NEC induction: *Il18*^*+*/+^ mice, *Il18*^−/−^ mice, and *Il18*^−/−^ mice adoptively transferred with wild-type iPMN-MDSCs (Fig. S5A). Compared with *Il18*^*+*/+^ littermates, *Il18*^−/−^ mice showed markedly exacerbated NEC severity, as reflected by reduced survival, increased intestinal inflammation, and compromised intestinal barrier integrity (Fig. S5B–G). This aggravated phenotype was accompanied by decreased abundance of IECs and iPMN-MDSCs (Fig. S5H–J), whereas other MDSC subsets were unaffected (Fig. S5K–M). Importantly, adoptive transfer of iPMN-MDSCs significantly reversed these pathological changes, restoring IEC abundance and function as well as iPMN-MDSC abundance in *Il18*^−/−^ neonates (Fig. S5B–J). Molecular validation further confirmed that *Il18* mRNA was undetectable in iPMN-MDSCs from *Il18*^−/−^ neonates, but its expression was restored following adoptive transfer of wild-type iPMN-MDSCs (Fig. S5N). Additionally, all measured pathological and cellular parameters were indistinguishable between *Il18*^*+*/+^ neonates and *Il18*^−/−^ neonates that received iPMN-MDSC transfer (Fig. S5B–N), confirming that iPMN-MDSC-derived IL-18 is essential for sustaining intestinal epithelial cell homeostasis and ameliorating NEC.

Intriguingly, transcriptomic analysis also revealed upregulation of *Olfm4*—a key intestinal stem cell marker—in probiotic-induced iPMN-MDSCs (Fig. 1N). Both qPCR and flow cytometry analyses confirmed increased OLFM4 expression in these cells following probiotic treatment (Fig. 1R, S). In summary, the probiotic combination of *L. reuteri* and *L. rhamnosus* attenuates NEC by expanding iPMN-MDSCs and restoring barrier integrity, which may be associated with the upregulation of OLFM4.

### Neutrophil *Olfm4* deficiency increases sensitivity to NEC and exacerbates intestinal dysbiosis

**Fig. 2:**
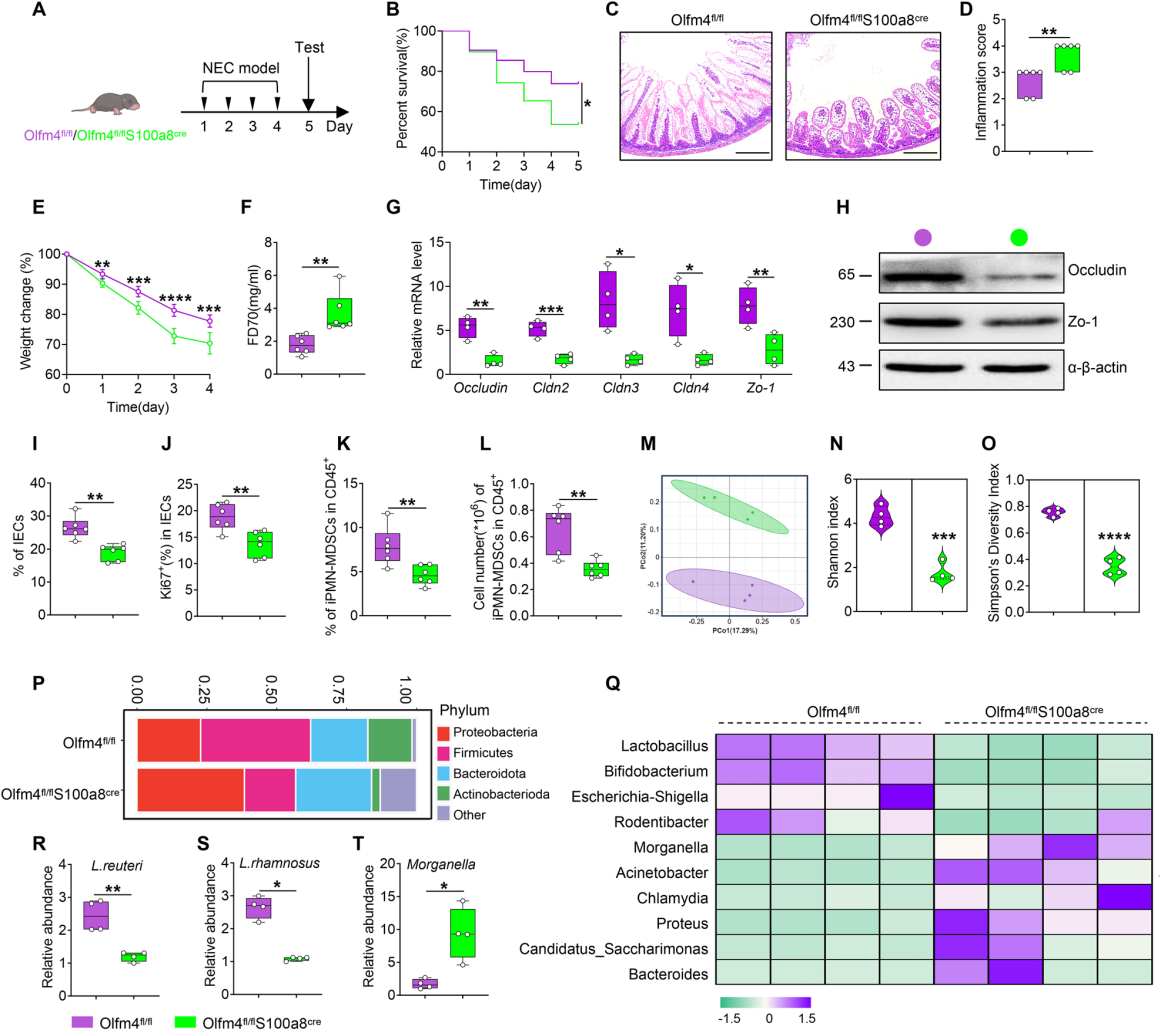
Neutrophil *Olfm4* deficiency increases sensitivity to NEC and exacerbates intestinal dysbiosis. Schematic of NEC induction in Olfm4^fl/fl^ and Olfm4^fl/fl^S100a8^cre^ neonates (**A**) and their survival rates (**B**, *n* = 50). Representative hematoxylin and eosin (H&E) staining of the intestine (**C**) and statistical analysis of inflammation scores (**D**, *n* = 6, scale bar: 50 μm). **E** Statistical analysis of weight change (*n* = 33 and 21). **F** Intestinal permeability was assessed by FD70 levels (*n* = 6). mRNA (**G**, *n* = 4 biological replicates) and protein (**H**, replicated three times) levels of TJ proteins in the intestine. Percentage (**I**) and proliferation (**J**) of IECs in Olfm4^fl/fl^ and Olfm4^fl/fl^S100a8^cre^ neonates (*n* = 6). **K,** **L** Statistical analysis of iPMN-MDSCs among CD45^+^ cells (*n* = 6). **M** Principal component analysis of the intestinal microbiota from Olfm4^fl/fl^ and Olfm4^fl/fl^S100a8^cre^ neonates. α-diversity of the intestinal microbiota was shown by Shannon (**N**) and Simpson’s (**O**) indices. Composition of the intestinal microbiota at the phylum (**P**) and genus (**Q**) levels (*n* = 4). The relative abundance of *L. reuteri* (**R**), *L. rhamnosus* (**S**), and *Morganella* (**T**) in fecal samples (*n* = 4). Data are presented as mean ± SEM. Each symbol represents one pup in a litter. ns, not significant; *p < 0.05, **p < 0.01, ***p < 0.001, ****p < 0.0001. Statistical significance was determined using a Student’s *t* test (**D–G,** **I–K,** **N,** **O**, and **R**), Mann–Whitney test (**G,** **L,** **S**, **T**), or log-rank (Mantel–Cox) test (**B**).

To define the functional role of OLFM4 in iPMN-MDSCs under NEC conditions, we generated neutrophil-specific *Olfm4*-deficient neonates (Olfm4^fl/fl^S100a8^cre^). Under physiological conditions, these pups showed minor differences from Olfm4^fl/fl^ controls in intestinal inflammation and MDSC subsets (Fig. S6A–F), with efficient *Olfm4* deletion confirmed (Fig. S6G, H). We therefore subjected them to NEC induction (Fig. 2A). Following NEC induction, *Olfm4*-deficient neonates developed more severe disease, as evidenced by reduced survival rate, increased intestinal inflammation, and pronounced weight loss (Fig. 2B–E). In addition, these neonates also exhibited increased intestinal permeability and decreased TJ protein expression (Fig. 2F–H), alongside diminished IEC abundance and proliferation (Fig. 2I, J). Crucially, *Olfm4* deficiency specifically reduced iPMN-MDSC levels, without affecting other MDSC subsets (Figs. 2K, L and S6I–N). Successful *Olfm4* deletion under NEC conditions was also confirmed at both the mRNA and protein levels (Fig. S6O, P). These results demonstrate that OLFM4 is essential for maintaining iPMN-MDSC abundance, and its deficiency in neutrophils aggravates NEC.

Building on our findings that link the probiotic cocktail and gut microbiota to NEC alleviation, we next asked whether the aggravated NEC phenotype in *Olfm4*-deficient neonates was associated with alterations in gut microbiota. Principal coordinate analysis revealed a distinct microbial profile in *Olfm4*-deficient neonates, with reduced Shannon and Simpson’s diversity indices and substantial microbiota remodeling at the phylum level (Fig. 2M–P). Specifically, the abundances of *Lactobacillus* and *Bifidobacterium* were decreased, whereas those of the genera *Morganella* and *Acinetobacter* were increased (Fig. 2Q). These microbial changes were further validated using qPCR (Fig. 2R–T). These data indicate that *Olfm4* deficiency in neutrophils drives microbial dysbiosis in the NEC model mice.

### Gut microbial interventions regulate NEC susceptibility associated with *Olfm4* deficiency

**Fig. 3:**
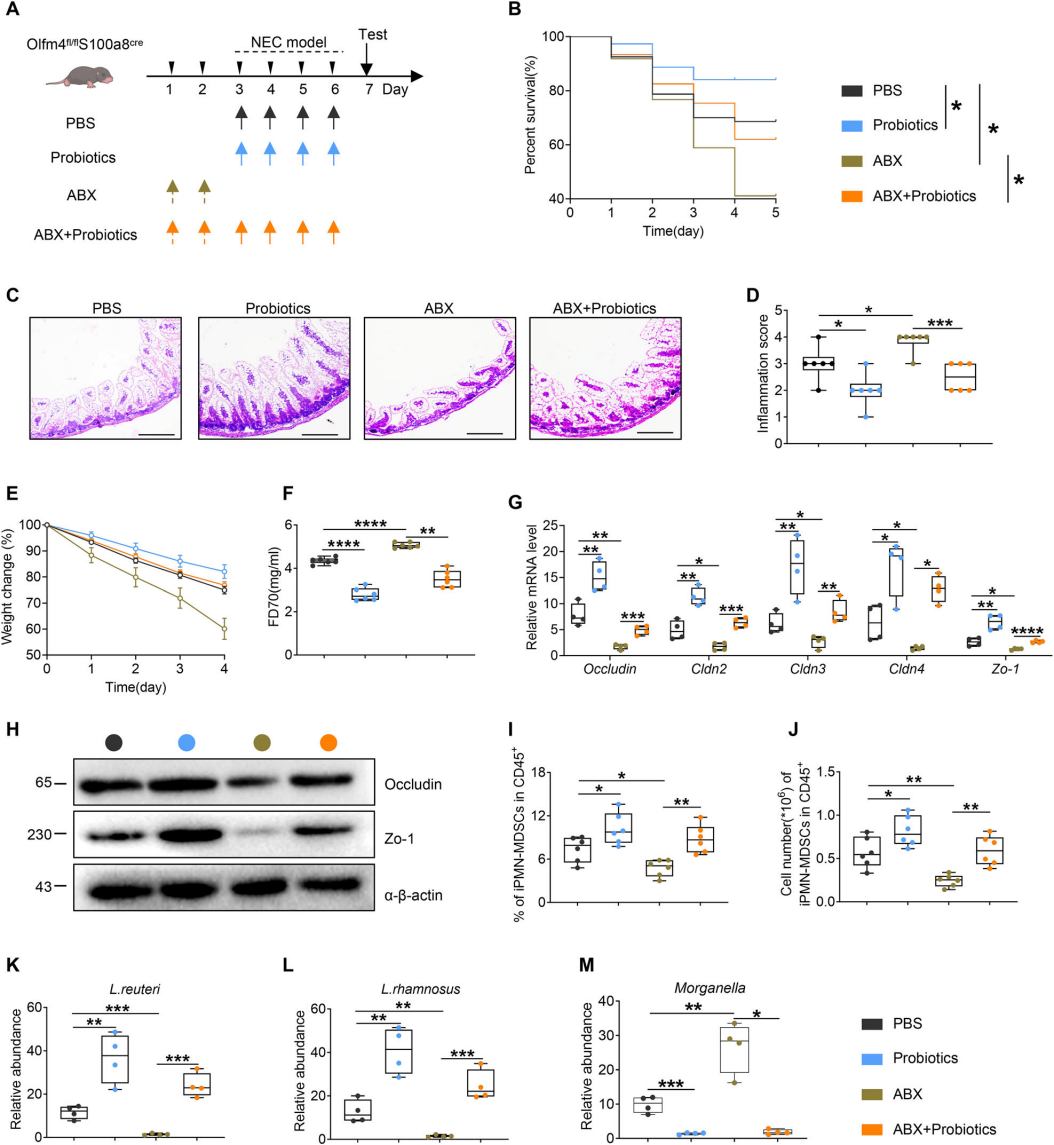
Gut microbial interventions regulate NEC susceptibility associated with *Olfm4* deficiency. **A** Olfm4^fl/fl^S100a8^cre^ pups were treated by oral gavage with one of the following: vehicle control, probiotic combination (*L. reuteri* and *L. rhamnosus*), a broad-spectrum antibiotic (ABX; 83 mg/kg/day metronidazole, ampicillin, neomycin sulfate, and vancomycin), or antibiotics followed by probiotics. **B** The survival rates of each group (*n* = 52, 43, 73, and 58). Representative H&E staining of the intestine (**C**) and statistical analysis of the inflammation scores (**D**, *n* = 6, scale bar: 50 μm). **E** Statistical analysis of weight change (*n* = 37, 31, 30, and 32). **F** Intestinal permeability was assessed by FD70 levels (*n* = 6). **G,** **H** mRNA (G, *n* = 4 biological replicates) and protein (H, replicated three times) levels of TJ proteins in the intestine. **I,** **J** Statistical analysis of iPMN-MDSCs among CD45^+^ cells (*n* = 6). The relative abundance of *L. reuteri* (**K**), *L. rhamnosus* (**L**), and *Morganella* (**M**) in fecal samples (*n* = 4). Data are presented as mean ± SEM. Each symbol represents one pup in a litter. ns, not significant; *p < 0.05, **p < 0.01, ***p < 0.001, ****p < 0.0001. Statistical significance was determined using one-way ANOVA (**D,** **F,** **G**, **I–M**) or log-rank (Mantel–Cox) test (**B**). Post**-**hoc analyses were performed using Tukey’s test (**D,** **F,** **G**, **I–M**).

Given the exacerbated NEC severity and microbial dysbiosis observed in *Olfm4*-deficient mice, we sought to determine whether the altered microbiota mediated the aggravated disease in these neonates. Specifically, seven-day-old *Olfm4*-deficient neonates received one of four treatments during NEC induction: vehicle control, probiotic cocktail (*L. reuteri* and *L. rhamnosus*), antibiotic treatment (ABX), and antibiotic pretreatment followed by probiotic cocktail (Fig. 3A). Probiotic treatment significantly alleviated NEC, as evidenced by improved survival, reduced intestinal inflammation, attenuated weight loss, and enhanced intestinal barrier integrity (Fig. 3B–H). In contrast, antibiotic treatment aggravated all these disease-related parameters. Critically, the exacerbating effects of antibiotics were reversed by subsequent probiotic administration (Fig. 3B–H). Flow cytometry analysis showed that the probiotic-mediated improvement was associated with increased iPMN-MDSC levels, but not other MDSC subsets (Figs. 3I, J and S7A–F). Consistent with these findings, probiotic treatment increased the abundances of *L. reuteri* and *L. rhamnosus* and decreased *Morganella* (Fig. 3K–M). Antibiotic treatment exhibited the opposite effects on both iPMN-MDSC levels and microbial community composition, which were restored by subsequent probiotic administration (Fig. 3I–M). Collectively, these data demonstrate that increased NEC susceptibility in *Olfm4*-deficient mice is microbiota-dependent and can be alleviated by probiotic-mediated microbial restoration.

### OLFM4-driven anti-ferroptosis axis in iPMN-MDSCs is essential for NEC alleviation

**Fig. 4:**
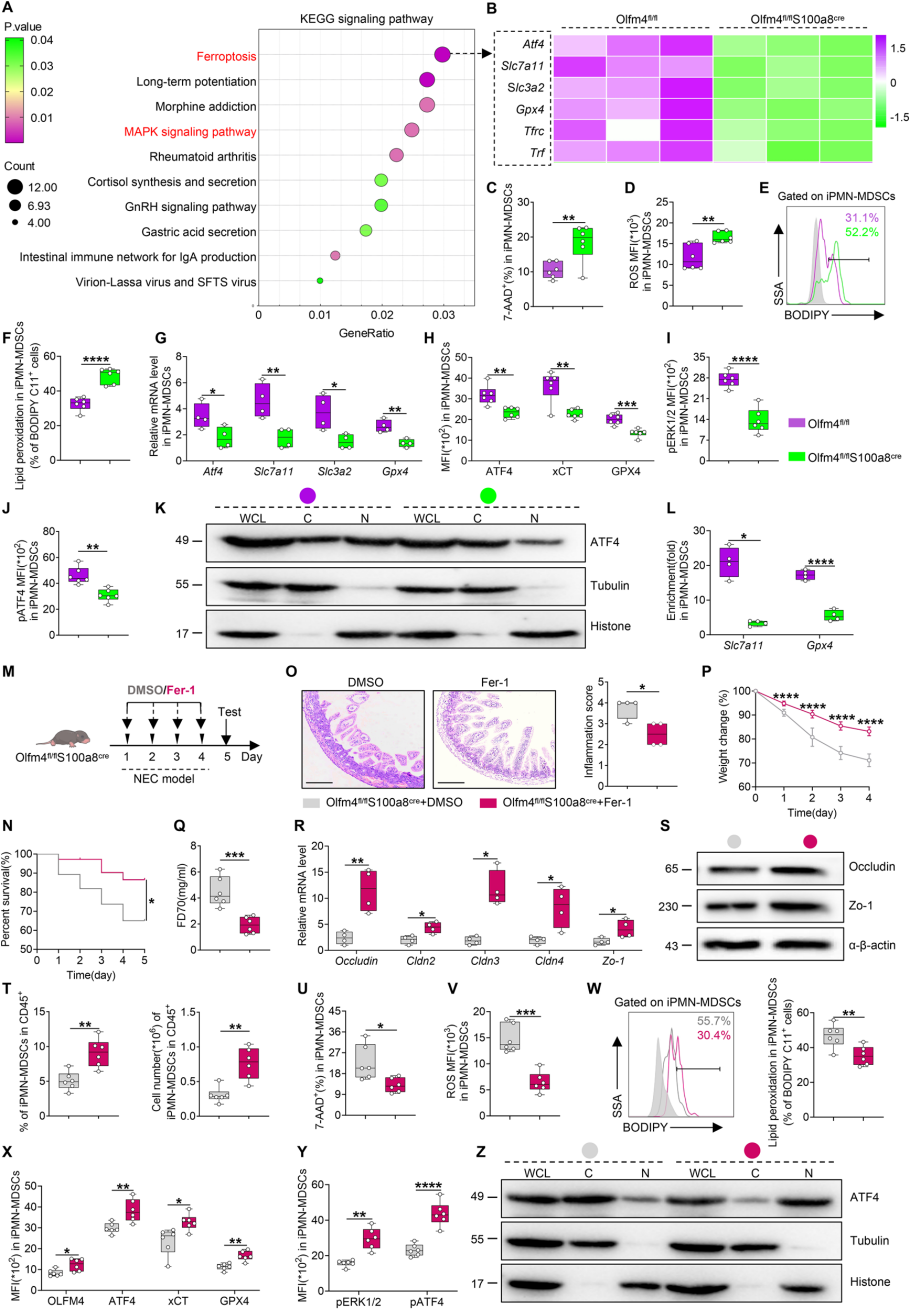
OLFM4-driven anti-ferroptosis axis in iPMN-MDSCs is essential for NEC alleviation. **A** Kyoto Encyclopedia of Genes and Genomes (KEGG) analysis of differently expressed genes in iPMN-MDSCs from Olfm4^fl/fl^ and Olfm4^fl/fl^S100a8^cre^ neonates. **B** Heatmap of downregulated genes in the ferroptosis signaling pathways (*n* = 3). **C** Cell viability of iPMN-MDSCs was assessed by 7-AAD staining (*n* = 6). **D** Reactive oxygen species (ROS) levels in iPMN-MDSCs (*n* = 6). Representative flow cytometry plots (**E**) and percentage of BODIPY C11^+^ cells (lipid peroxidation) in iPMN-MDSCs (**F**, *n* = 6). **G** mRNA expression levels of *Atf4*, *Slc7a11*, *Slc3a2*, and *Gpx4* in iPMN-MDSCs (*n* = 4 biological replicates). **H** Levels of ATF4, xCT, and GPX4 in iPMN-MDSCs (*n* = 6). **I,** **J** MFI of phosphorylated ERK1/2 (pERK1/2) and phosphorylated ATF4 (pATF4) in iPMN-MDSCs (*n* = 6). **K** Western blot analysis of ATF4 distribution in whole-cell lysate (WCL), cytoplasm (C), and nucleus (N) of iPMN-MDSCs sorted from Olfm4^fl/fl^ and Olfm4^fl/fl^S100a8^cre^ neonates (replicated three times). **L** ATF4 enrichment at the Slc7a11 and Gpx4 promoter regions of iPMN-MDSCs from Olfm4^fl/fl^ and Olfm4^fl/fl^S100a8^cre^ pups (*n* = 4). **M,** **N** Olfm4^fl/fl^S100a8^cre^ neonates were treated with dimethyl sulfoxide (DMSO) or 1 mg/kg/day ferrostatin-1 (Fer-1) by oral gavage during NEC induction (M), and the survival rates were calculated (N, *n* = 25). **O** Representative H&E staining of the intestine and statistical analysis of inflammation scores (*n* = 4, scale bar: 50 μm). **P** Statistical analysis of weight change (*n* = 14 and 21). **Q** FD70 levels in the indicated groups (*n* = 6). **R,** **S** mRNA (R, *n* = 4 biological replicates) and protein (S, replicated three times) levels of TJ proteins in the intestine. **T** Statistical analysis of iPMN-MDSCs among CD45^+^ cells (*n* = 6). **U–W** Statistical analysis of 7-AAD staining (U), ROS levels (V), and lipid peroxidation (W) in iPMN-MDSCs (*n* = 6). **X** Levels of OLFM4, ATF4, xCT, and GPX4 in iPMN-MDSCs (*n* = 6). **Y** MFI of pERK1/2 and pATF4 in iPMN-MDSCs (*n* = 6). **Z** Western blot analysis of ATF4 distribution in whole-cell lysate (WCL), cytoplasm (C), and nucleus (N) of iPMN-MDSCs sorted from Olfm4^fl/fl^S100a8^cre^ neonates treated with DMSO or Fer-1 (replicated twice). Data are presented as mean ± SEM. Each symbol represents one pup in a litter. ns, not significant; *p < 0.05, **p < 0.01, ***p < 0.001, ****p < 0.0001. Statistical significance was determined using a Student’s *t* test (**C,** **D,** **F–J,** **L,** **O–R**, **T–Y**), Mann–Whitney test (**L,** **R**, **Y**), or log-rank (Mantel–Cox) test (**N**).

Given the essential role of OLFM4 in iPMN-MDSCs, we next investigated its underlying mechanisms. RNA-seq analysis revealed that differentially expressed genes in iPMN-MDSCs from Olfm4^fl/fl^ versus Olfm4^fl/fl^S100a8^cre^ pups were significantly enriched in the ferroptosis signaling pathways (Fig. 4A). Gene set enrichment analysis (GSEA) further demonstrated upregulation of ROS biosynthesis and downregulation of ferrous iron binding in *Olfm4*-deficient iPMN-MDSCs (Fig. S8A, B). Notably, key anti-ferroptosis genes, including *Atf4*, *Slc7a11*, *Slc3a2*, and *Gpx4*, were downregulated (Fig. 4B). Consistent with this transcriptional profile, *Olfm4* deficiency resulted in increased ferroptosis—evidenced by reduced cell viability and elevated ROS and lipid ROS levels—but not apoptosis (Figs. 4C–F and S8C). Correspondingly, both mRNA and protein levels of ATF4, SLC7A11, SLC3A2, and GPX4 were downregulated in *Olfm4*-deficient iPMN-MDSCs (Fig. 4G, H). Given the concurrent enrichment of the MAPK pathway in these cells (Fig. 4A), we assessed its activation and found that phosphorylated ERK1/2 (pERK1/2), rather than other tested MAPK components, was downregulated in *Olfm4*-deficient iPMN-MDSCs (Figs. 4I and S8D). As ERK1/2 phosphorylation modulates ATF4 nuclear translocation and transcriptional activity [42, 43], we next assessed the activation and subcellular localization of ATF4. Flow cytometry analysis revealed decreased levels of phosphorylated ATF4 (pATF4) in *Olfm4*-deficient iPMN-MDSCs (Fig. 4J). Consistently, ATF4 nuclear translocation was impaired, with a shift in its subcellular localization from predominantly nuclear to cytoplasmic (Fig. 4K). Hence, OLFM4 promotes ATF4 phosphorylation and nuclear localization via the ERK1/2 pathway, thereby suppressing iPMN-MDSC ferroptosis.

We next investigated whether ATF4 directly regulates the transcription of anti-ferroptosis-related genes. Using the JASPAR database, we identified potential ATF4 binding sites in the Slc7a11 and Gpx4 promoters. Chromatin immunoprecipitation (ChIP) assays performed with an anti-ATF4 antibody confirmed ATF4 binding at these promoters in iPMN-MDSCs (Fig. S9A–D); this binding was significantly diminished in *Olfm4*-deficient iPMN-MDSCs (Fig. 4L). Given the concurrent downregulation of IL-18 (Fig. S9E–G), we also examined the Il18 promoter and confirmed ATF4 binding, which was similarly impaired upon *Olfm4* deficiency (Fig. S9H–J). These findings indicate that OLFM4 regulates the transcriptional activity of ATF4, driving the expression of anti-ferroptosis genes and IL-18 in iPMN-MDSCs. Based on the fact that probiotic interventions sustain iPMN-MDSC homeostasis and upregulate OLFM4, we hypothesized that probiotic-mediated effects involve the inhibition of ferroptosis in iPMN-MDSCs. In probiotic-treated NEC pups, iPMN-MDSCs exhibited attenuated ferroptosis alongside activation of the ERK–ATF4–xCT/GPX4 axis (Fig. S10A–H). These data indicate that the probiotic treatment alleviates NEC by inhibiting iPMN-MDSC ferroptosis.

Given the role of OLFM4 in regulating iPMN-MDSC ferroptosis, we next tested whether inhibiting this process could rescue the aggravated NEC in *Olfm4*-deficient mice. Seven-day-old *Olfm4*-deficient pups received either dimethyl sulfoxide (DMSO) or ferrostatin-1 (Fer-1) by oral gavage during NEC induction (Fig. 4M). Fer-1 treatment significantly alleviated NEC severity, as evidenced by improved survival, reduced intestinal inflammation, attenuated weight loss, decreased intestinal permeability, and restored TJ protein expression (Fig. 4N–S). This alleviation was associated with increased iPMN-MDSC levels and viability, alongside reduced ferroptosis, without affecting other MDSCs or apoptosis (Figs. 4T–W and S11A–G). Furthermore, Fer-1 treatment activated the OLFM4–ERK–ATF4–xCT/GPX4 signaling axis and promoted ATF4 nuclear translocation (Fig. 4X–Z). These data demonstrate that inhibiting iPMN-MDSC ferroptosis effectively mitigates NEC in the context of *Olfm4* deficiency.

### Inhibiting Atf4 or Gpx4 expression in iPMN-MDSCs aggravates NEC severity

**Fig. 5:**
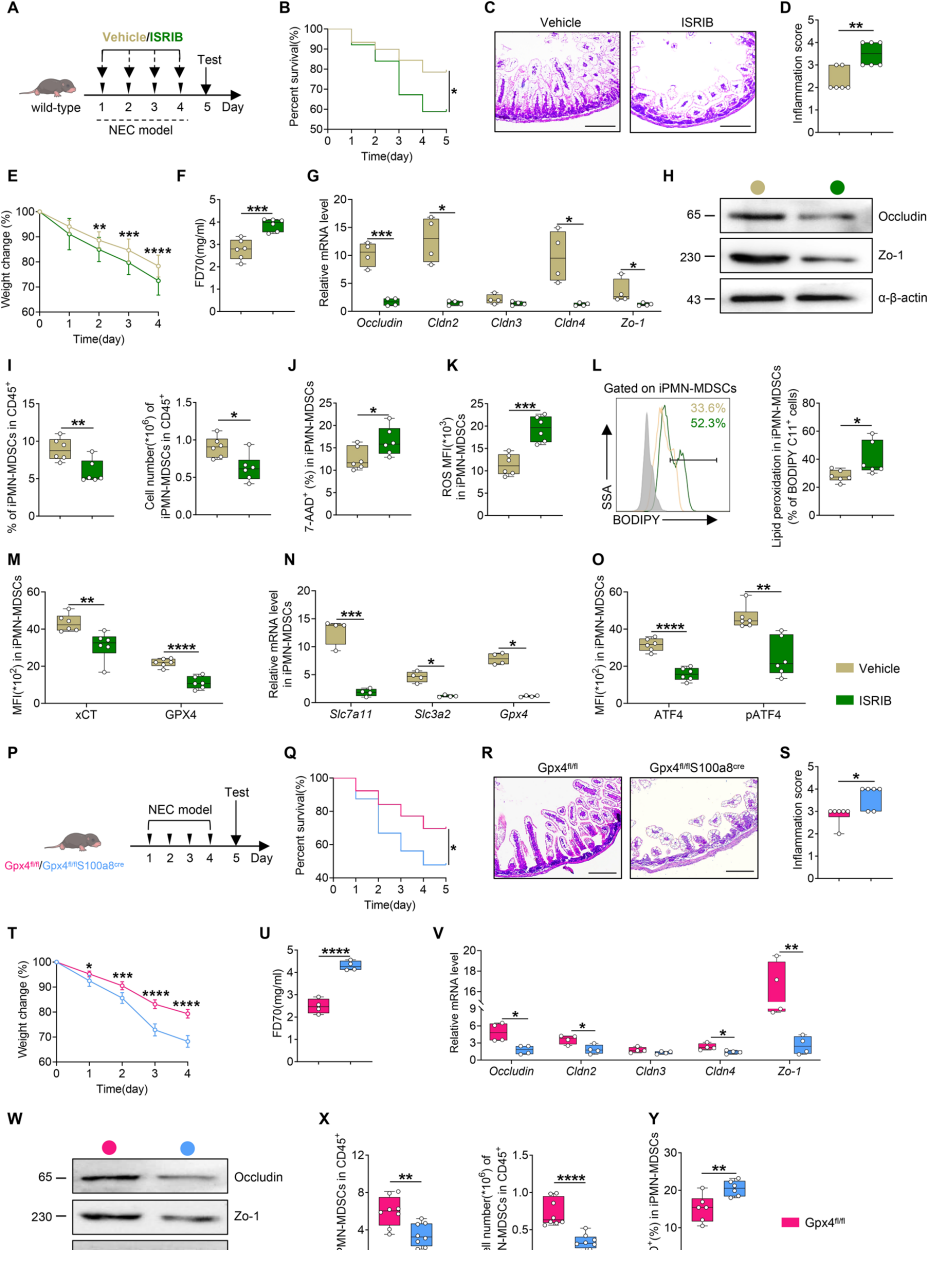
Inhibiting Atf4 or Gpx4 expression in iPMN-MDSCs aggravates NEC severity. **A** Seven-day-old wild-type pups were treated with a vehicle control or 0.25 mg/kg/day ISRIB by oral gavage during NEC induction. **B** The survival rates of each group were recorded over 24 h (*n* = 46). Representative H&E staining of the intestine (**C**) and statistical analysis of inflammation scores (**D**, *n* = 6, scale bar: 50 μm). **E** Statistical analysis of weight change (*n* = 37 and 20). **F** Intestinal permeability was assessed by FD70 levels (*n* = 6). mRNA (**G**, *n* = 4 biological replicates) and protein (**H**, replicated three times) levels of TJ proteins in the intestine. **I** Statistical analysis of iPMN-MDSCs among CD45^+^ cells (*n* = 6). **J**–**L** Statistical analysis of 7-AAD staining (**J**), ROS levels (**K**), and lipid peroxidation (L) in iPMN-MDSCs (*n* = 6). **M** Levels of xCT and GPX4 in iPMN-MDSCs (*n* = 6). **N** mRNA expression levels of *Slc7a11*, *Slc3a2*, and *Gpx4* in iPMN-MDSCs (*n* = 4 biological replicates). **O** MFI of ATF4 and pATF4 in iPMN-MDSCs (*n* = 6). Schematic of NEC induction in Gpx4^fl/fl^ and Gpx4^fl/fl^S100a8^cre^ neonates (**P**), and the survival rates of mice were calculated (**Q**, *n* = 40). Representative H&E staining of the intestine (**R**) and statistical analysis of inflammation scores (**S**, *n* = 6, scale bar: 50 μm). **T** Statistical analysis of weight change (*n* = 26 and 16). **U** FD70 levels in the indicated groups (*n* = 4). mRNA (**V**, *n* = 4 biological replicates) and protein (**W**, replicated three times) levels of TJ proteins in the intestine. **X** Statistical analysis of iPMN-MDSCs among CD45^+^ cells (*n* = 8). **Y** Statistical analysis of 7-AAD^+^ cells in iPMN-MDSCs (*n* = 6). Data are presented as mean ± SEM. Each symbol represents one pup in a litter. ns, not significant; *p < 0.05, **p < 0.01, ***p < 0.001, ****p < 0.0001. ns, not significant; *p < 0.05, **p < 0.01, ***p < 0.001, ****p < 0.0001. Statistical significance was determined using a Student’s *t* test (**D–G,** **I–K,** **M–O,** **S–V,** **X**, **Y**), Mann–Whitney test (**G,** **L**, **N**), or log-rank (Mantel–Cox) test (**B**, **Q**).

In light of the central roles of ATF4 and GPX4 in the ferroptosis pathways, we next investigated their functional requirement in iPMN-MDSCs during NEC induction. We first targeted ATF4 using the integrated stress response inhibitor (ISRIB), a specific inhibitor that suppresses ATF4 transcription [44, 45]. Following NEC induction (Fig. 5A), ISRIB treatment significantly exacerbated disease severity, manifested as reduced survival rates, increased intestinal inflammation, elevated weight loss, enhanced intestinal permeability, and decreased TJ protein expression (Fig. 5B–H). Flow cytometry analysis revealed that this exacerbation was specifically associated with reduced iPMN-MDSC levels, without affecting other MDSC subsets (Figs. 5I and S12A–F). In addition, ISRIB promoted ferroptosis in iPMN-MDSCs by suppressing SLC7A11 and GPX4 expression, without affecting apoptosis or OLFM4 expression (Figs. 5J–N and S12G, H). Moreover, IL-18 expression and secretion in iPMN-MDSCs were also reduced following ISRIB treatment (Fig. S12I, J). As expected, ISRIB effectively inhibited both ATF4 expression and phosphorylation in iPMN-MDSCs (Fig. 5O). These data demonstrate that inhibiting ATF4 exacerbates NEC by reducing iPMN-MDSC levels and enhancing ferroptosis within these cells.

We next employed neutrophil-specific *Gpx4*-deficient mice to assess the role of this key ferroptosis effector. Under physiological conditions, no significant differences were observed in intestinal inflammation or MDSC levels between Gpx4^fl/fl^ and Gpx4^fl/fl^S100a8^cre^ littermate neonates, with confirmed *Gpx4* deletion in PMN-MDSCs (Fig. S13A–H). However, upon NEC induction (Fig. 5P), *Gpx4*-deficient neonates developed more severe disease, characterized by reduced survival rates, higher intestinal inflammation, enhanced weight loss, increased intestinal permeability, and decreased TJ protein expression (Fig. 5Q–W). This aggravated phenotype was associated with a marked reduction in iPMN-MDSC abundance and viability, without significant changes in other MDSC subsets or in apoptosis (Figs. 5X, Y and S13I–O). Moreover, no significant differences were observed in the expression of the OLFM4–ATF4–xCT axis (Fig. S13P). Successful *Gpx4* deletion in PMN-MDSCs under NEC conditions was further confirmed at both the mRNA and protein levels (Fig. S13Q, R). These data highlight the crucial role of GPX4 in maintaining iPMN-MDSC function specifically under NEC conditions.

Based on our in vivo observations that *Gpx4* deficiency did not affect iPMN-MDSC survival under physiological conditions but significantly impaired it during NEC (Figs. 5X and S13C), we established an in vitro model using the GPX4-specific inhibitor RSL3 to directly assess this context-dependent essentiality [31]. iPMN-MDSCs sorted from neonates under physiological or NEC conditions were treated with increasing concentrations of RSL3. Notably, NEC-derived iPMN-MDSCs exhibited significantly greater sensitivity to RSL3, as evidenced by a more marked decrease in cell viability (Fig. S14A). This heightened sensitivity was associated with an upregulated GPX4 expression under NEC conditions. Importantly, upon RSL3 treatment, GPX4 inhibition was more pronounced in NEC-derived iPMN-MDSCs than in those isolated from physiological conditions (Fig. S14B). Together, these data indicate that iPMN-MDSC survival relies on GPX4 in a context-dependent manner, particularly under NEC conditions.

### Probiotic intervention inhibits iPMN-MDSC ferroptosis and ameliorates NEC through the metabolite I3A–AHR axis

To explore how probiotic interventions alleviate NEC by targeting iPMN-MDSC ferroptosis, we profiled tryptophan derivatives—the main byproducts of *L. reuteri* and *L. rhamnosus*—in fecal samples using liquid chromatography-mass spectrometry (LC-MS/MS). The results identified indole-3-aldehyde (I3A) as the most markedly increased metabolite following probiotic treatment (Fig. S15A). In contrast, other reported tryptophan derivatives, such as indole-3-carbinol (I3C) and indole-3-propionic acid (IPA), remained unchanged (Fig. S15A). Enzyme-linked immunosorbent assay (ELISA) further confirmed that I3A, rather than I3C or IPA, was specifically and significantly increased in feces, intestine, and plasma (Fig. S15B–D). We then tested the effects of these metabolites on iPMN-MDSC ferroptosis in vitro at physiologically relevant concentrations (200 μM) (Fig. S15E). Notably, I3A inhibited ferroptosis in iPMN-MDSCs more potently than the other two metabolites, as shown by increased cell viability and decreased levels of ROS and lipid ROS (Fig. S15F–H). Furthermore, I3A activated the anti-ferroptosis axis OLFM4–ATF4–xCT/GPX4 in iPMN-MDSCs (Fig. S15I). I3A levels were also quantified in healthy controls and NEC model mice. Compared to the controls, I3A was reduced in feces, intestine, and within iPMN-MDSCs of NEC neonates (Fig. S16A–C). Moreover, I3A was significantly increased in pups treated with the combined probiotics compared to those treated with a single strain (Fig. S16D). Overall, I3A is a key probiotic-derived metabolite that is reduced in the NEC model mice and restored by combined probiotic treatment. Critically, the specific inhibition of I3A in iPMN-MDSC ferroptosis provides a mechanistic link between its restoration and NEC alleviation.

**Fig. 6:**
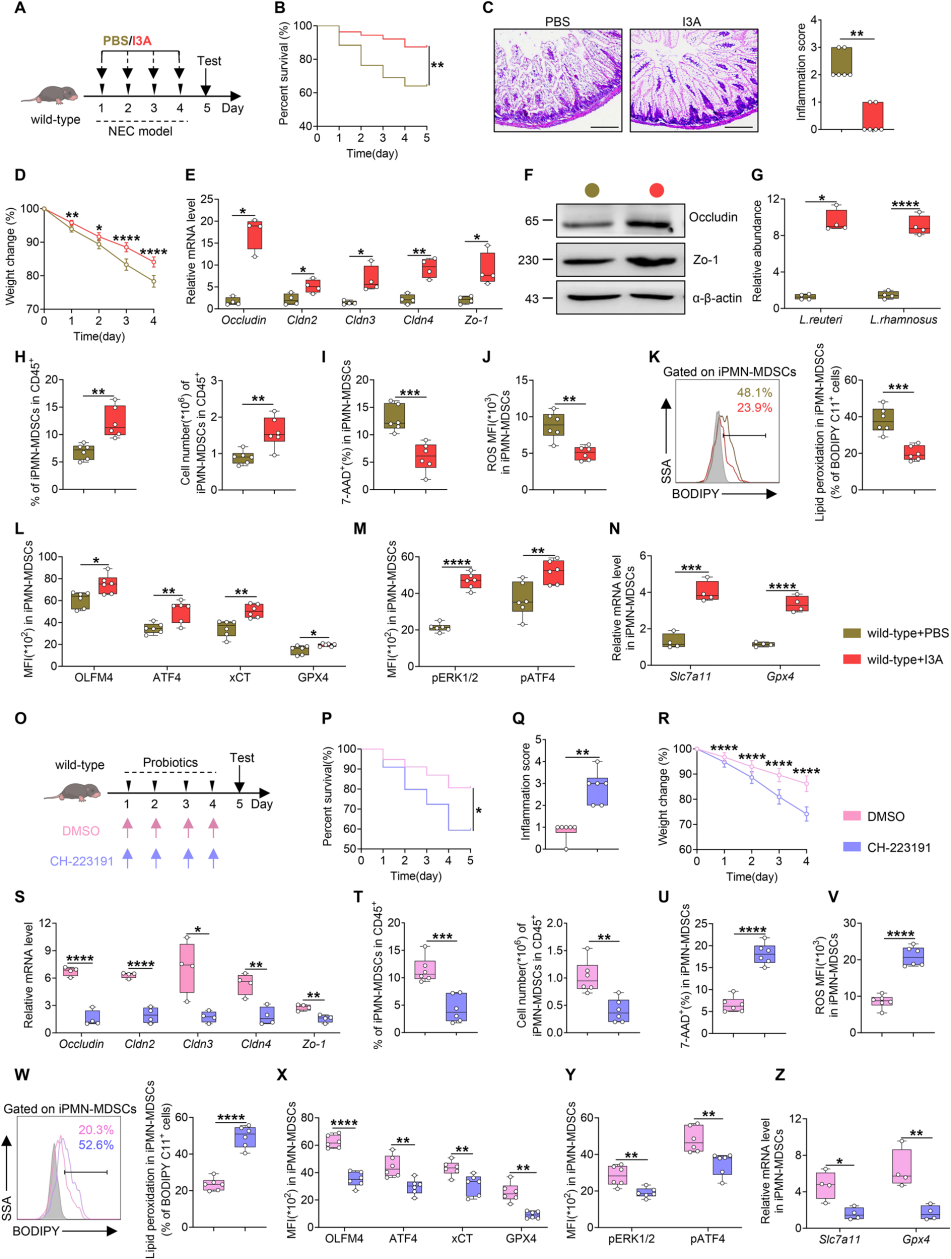
Probiotic intervention inhibits iPMN-MDSC ferroptosis and ameliorates NEC through the metabolite I3A–AHR axis. Seven-day-old wild-type neonates were treated with PBS or 25 mg/kg/day I3A by oral gavage during NEC induction (**A**), and the survival rates of each group were calculated (**B**, *n* = 42). **C** Representative H&E staining of the intestine and statistical analysis of inflammation scores (*n* = 6, scale bar: 50 μm). **D** Statistical analysis of weight change (*n* = 25 and 36). mRNA (**E**, *n* = 4 biological replicates) and protein (**F**, replicated three times) levels of TJ proteins in the intestine. **G** The relative abundance of *L. reuteri* and *L. rhamnosus* in fecal samples (*n* = 4). **H** Statistical analysis of iPMN-MDSCs among CD45^+^ cells (*n* = 6). Statistical analysis of 7-AAD staining (**I**), ROS levels (**J**), and lipid peroxidation (**K**) in iPMN-MDSCs (*n* = 6). **L** Levels of OLFM4, ATF4, xCT, and GPX4 in iPMN-MDSCs (*n* = 6). **M** MFI of pERK1/2 and pATF4 in iPMN-MDSCs (*n* = 6). **N** mRNA epxression levels of *Slc7a11* and *Gpx4* in iPMN-MDSCs (*n* = 4 biological replicates). **O** Seven-day-old wild-type pups were orally administered probiotics (*L. reuteri* and *L. rhamnosus*) and concurrently injected intraperitoneally with either DMSO or 10 mg/kg/day CH-223191 during NEC induction. **P** The survival rates of each group (*n* = 44 and 54). **Q** Statistical analysis of inflammation scores (*n* = 6). **R** Statistical analysis of weight change (*n* = 34 and 32). **S** mRNA expression levels of TJ proteins in the intestine (*n* = 4 biological replicates). **T** Statistical analysis of iPMN-MDSCs among CD45^+^ cells (*n* = 6). Statistical analysis of 7-AAD staining (**U**), ROS levels (**V**), and lipid peroxidation (**W**) in iPMN-MDSCs (*n* = 6). **X** Levels of OLFM4, ATF4, xCT, and GPX4 in iPMN-MDSCs (*n* = 6). **Y** MFI of pERK1/2 and pATF4 in iPMN-MDSCs (*n* = 6). **Z** mRNA expression levels of *Slc7a11* and *Gpx4* in iPMN-MDSCs (*n* = 4 biological replicates). Data are presented as mean ± SEM. Each symbol represents one pup in a litter. ns, not significant; *p < 0.05, **p < 0.01, ***p < 0.001, ****p < 0.0001. ns, not significant; *p < 0.05, **p < 0.01, ***p < 0.001, ****p < 0.0001. Statistical significance was determined using a Student’s *t* test (**D,** **E,** **G–N**, **R–Z**), Mann–Whitney test (**C,** **E,** **G,** **L,** **Q,** **S**, **X**), or log-rank (Mantel–Cox) test (**B**, **P**).

We next explored the therapeutic potential of I3A in vivo. Seven-day-old wild-type pups received either vehicle control or I3A by oral gavage during NEC induction (Fig. 6A). I3A treatment significantly alleviated NEC, as evidenced by improved survival, reduced intestinal inflammation, attenuated weight loss, and restored barrier integrity (Fig. 6B–F). Following I3A treatment, the abundances of *L. reuteri* and *L. rhamnosus* were increased, while those of *Morganella* were decreased (Figs. 6G and S17A). Notably, I3A specifically increased iPMN-MDSC abundance and inhibited ferroptosis in these cells, with minimal effects on other MDSC subsets or on apoptosis (Figs. 6H–K and S17B–H). Moreover, I3A increased the expression of OLFM4, ATF4, xCT, and GPX4 in iPMN-MDSCs, along with pERK1/2 and pATF4 levels (Fig. 6L, M). This enhanced ATF4 activity was associated with the upregulation of *Slc7a11*, *Gpx4*, and *Il18* (Figs. 6N and S17I, J). Although I3A did not alter aryl hydrocarbon receptor (*Ahr*) expression in iPMN-MDSCs, it upregulated *Cyp1a1*, a marker of AHR signaling activation (Fig. S17K, L). Given the increased abundance of *L. reuteri* and *L. rhamnosus* observed upon I3A treatment, we further examined its direct effects on these two strains. I3A markedly promoted their proliferation in vitro, particularly during the logarithmic growth phase (Fig. S18A, B). Together, these results indicate that I3A mitigates NEC by activating the OLFM4-mediated anti-ferroptosis pathway and promoting the proliferation of *L. reuteri* and *L. rhamnosus*.

Based on the upregulation of *Cyp1a1* in iPMN-MDSCs upon I3A treatment, we next examined whether this pathway is necessary for mediating the probiotic effects. Seven-day-old wild-type neonates were co-treated with the probiotic cocktail and either DMSO or the AHR-specific inhibitor CH-223191 via intraperitoneal injection during NEC induction (Fig. 6O). AHR inhibition abrogated the protective effects of the probiotics, as evidenced by reduced survival rates, increased intestinal inflammation, greater weight loss, and exacerbated barrier damage (Figs. 6P–S and S19A). Flow cytometry analysis showed that AHR inhibition reversed the probiotic-induced accumulation of iPMN-MDSCs and promoted ferroptosis in these cells, without affecting other MDSC subsets or apoptosis (Figs. 6T–W and S19B–H). Correspondingly, AHR inhibition suppressed activation of the downstream OLFM4**–**ERK**–**ATF4**–**xCT/GPX4 signaling pathway (Fig. 6X–Z). Successful AHR inhibition was confirmed by reduced expression of its target *Cyp1a1* (Fig. S19I). These data indicate that the protective effect of probiotics is mediated through the AHR signaling—a critical mechanism for inhibiting iPMN-MDSC ferroptosis and ameliorating NEC.

### OLFM4 enhances the therapeutic efficacy of I3A by strengthening the anti-ferroptosis response in iPMN-MDSCs

**Fig. 7:**
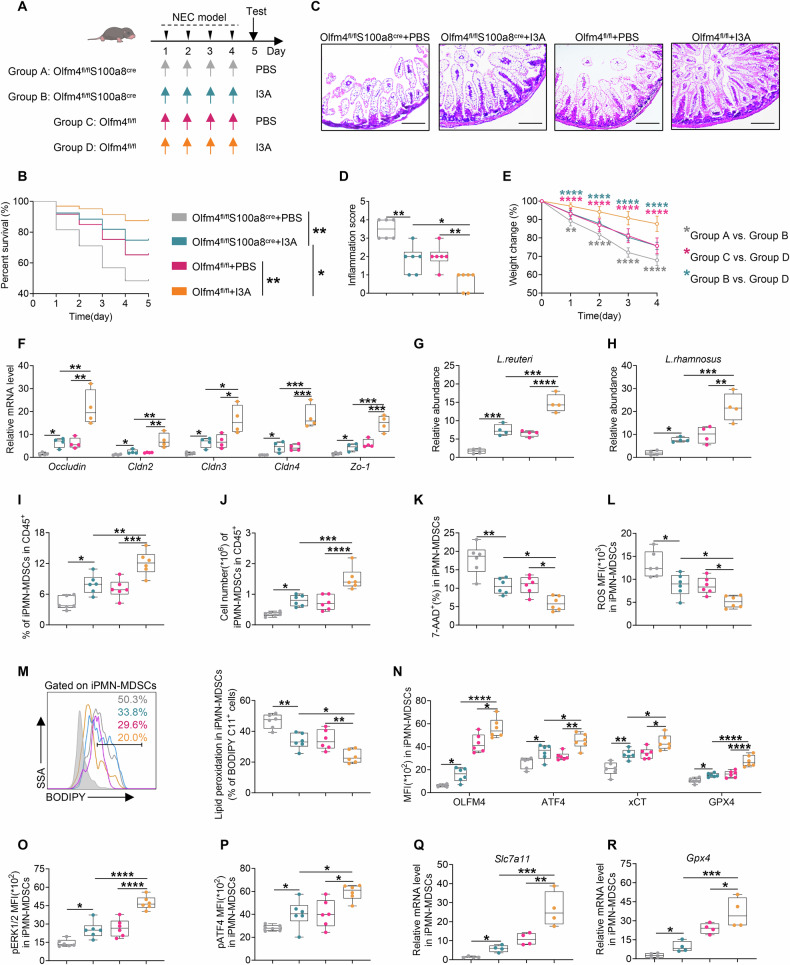
OLFM4 enhances the therapeutic efficacy of I3A by strengthening the anti-ferroptosis response in iPMN-MDSCs. **A** Olfm4^fl/fl^S100a8^cre^ and Olfm4^fl/fl^ littermate neonates were treated with PBS or 25 mg/kg/day I3A by oral gavage during NEC induction. **B** The survival rates of each group (*n* = 35, 41, 41, and 48). Representative H&E staining of the intestine (**C**) and statistical analysis of inflammation scores (**D**, *n* = 6, scale bar: 50 μm). **E** Statistical analysis of weight change (*n* = 16, 30, 25, and 36). **F** mRNA expression levels of TJ proteins in the intestine (*n* = 4 biological replicates). The relative abundance of *L. reuteri* (**G**) and *L. rhamnosu*s (**H**) in fecal samples (*n* = 4). **I,** **J** Statistical analysis of iPMN-MDSCs among CD45^+^ cells (*n* = 6). Statistical analysis of 7-AAD staining (**K**), ROS levels (**L**), and lipid peroxidation (**M**) in iPMN-MDSCs (*n* = 6). **N** Levels of OLFM4, ATF4, xCT, and GPX4 in iPMN-MDSCs (*n* = 6). MFI of pERK1/2 (**O**) and pATF4 (**P**) in iPMN-MDSCs (*n* = 6). mRNA expression levels of *Slc7a11* (**Q**) and *Gpx4* (**R**) in iPMN-MDSCs (*n* = 4 biological replicates). Data are presented as mean ± SEM. Each symbol represents one pup in a litter. ns, not significant; *p < 0.05, **p < 0.01, ***p < 0.001, ****p < 0.0001. ns, not significant; *p < 0.05, **p < 0.01, ***p < 0.001, ****p < 0.0001. Statistical significance was determined using one-way ANOVA (**D–R**) or log-rank (Mantel–Cox) test (**B**). Post-hoc analyses were performed using Tukey’s test (**D–R**).

To determine whether the therapeutic effect of I3A requires OLFM4, we compared its efficacy in Olfm4^fl/fl^S100a8^cre^ and Olfm4^fl/fl^ littermate neonates, with PBS treatment serving as the control (Fig. 7A). Compared with PBS, I3A treatment alleviated NEC in both genotypes, as reflected by improved survival, reduced intestinal inflammation, mitigated weight loss, and preserved intestinal barrier integrity. Notably, these beneficial effects were significantly more robust in I3A-treated Olfm4^fl/fl^ neonates than in their *Olfm4*-deficient counterparts (Fig. 7B–F). Consistently, I3A-driven modulation of the gut microbiota—including increased *L. reuteri* and *L. rhamnosus* and reduced *Morganella*—was also more pronounced in the presence of OLFM4 (Figs. 7G, H and S20A). Flow cytometry analysis showed that I3A increased iPMN-MDSC abundance and suppressed ferroptosis in these cells in both I3A-treated groups compared with PBS controls, with these changes again being significantly stronger in Olfm4^fl/fl^ neonates (Fig. 7I–M). In contrast, no comparable differences were observed in other MDSC subsets or in apoptosis (Fig. S20B–H). Additionally, I3A treatment activated the OLFM4**–**ERK**–**ATF4**–**xCT/GPX4 signaling axis in iPMN-MDSCs, and this activation, together with the upregulation of *Il18* and *Cyp1a1*, was markedly enhanced in Olfm4^fl/fl^ neonates (Figs. 7N–R and S20I–K). Collectively, these findings indicate that OLFM4 potentiates the therapeutic effect of I3A by bolstering the anti-ferroptosis response in iPMN-MDSCs.

### Reduced intestinal LOX1^+^ PMN-MDSC levels and impaired anti-ferroptosis axis correlate with NEC progression

**Fig. 8:**
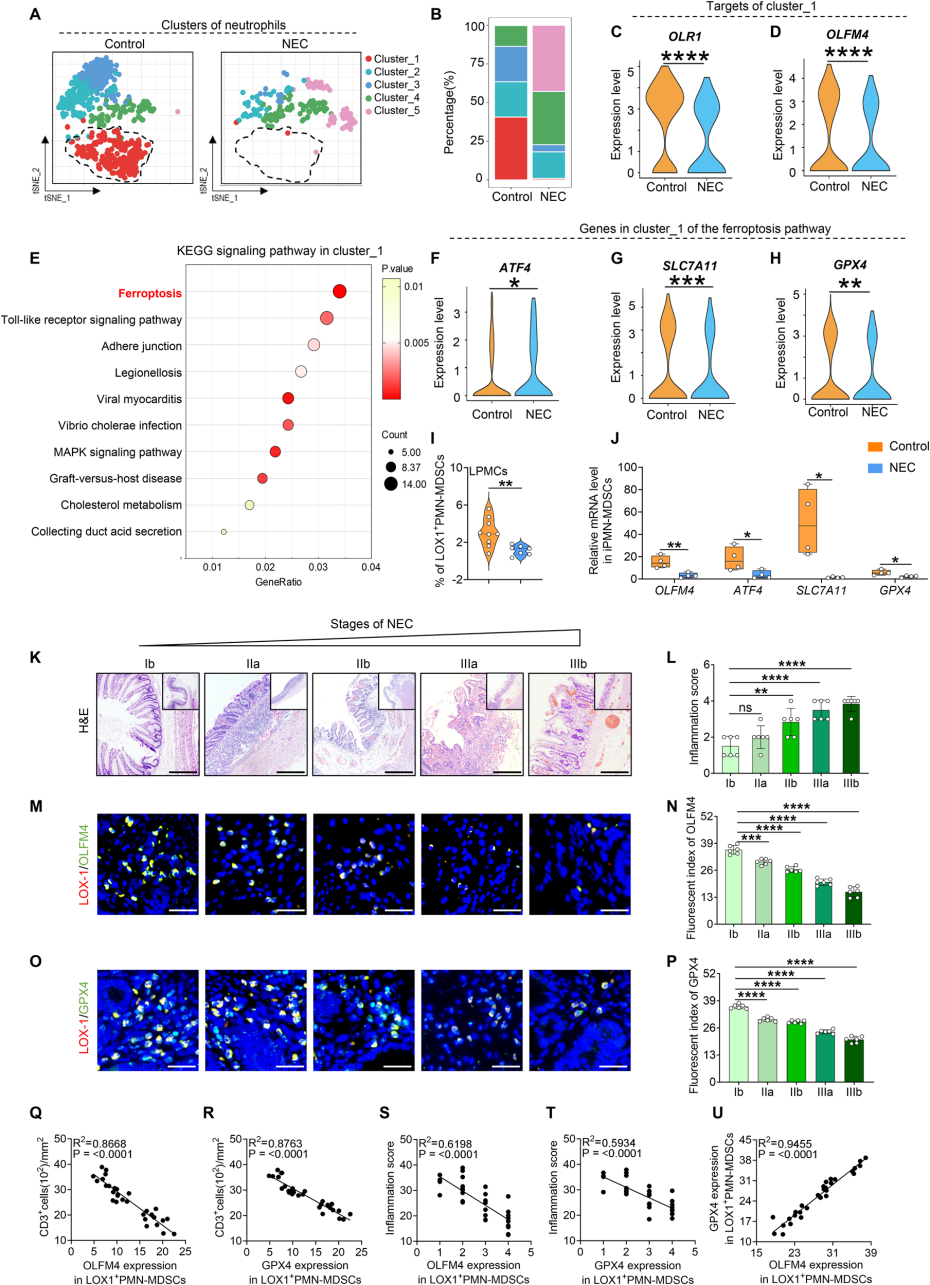
Reduced intestinal LOX1^+^ PMN-MDSC levels and impaired anti-ferroptosis axis correlate with NEC progression. **A,** **B** t-distributed stochastic neighbor embedding (t-SNE) visualization revealed five major neutrophil clusters and demonstrated a reduction in cluster_1 in infants with NEC (*n* = 5). Violin plots of *OLR1* (**C**) and *OLFM4* (**D**) expression in cluster_1 neutrophils. **E** KEGG analysis of differentially expressed genes in cluster_1 neutrophils from controls and infants with NEC. Violin plots of *ATF4* (**F**), *SLC7A11* (**G**), and *GPX4* (**H**) expression in cluster_1 neutrophils. **I** Statistical analysis of intestinal LOX1^+^ PMN-MDSCs (*n* = 9 and 7). **J** mRNA expression levels of *OLFM4*, *ATF4*, *SLCA711*, and *GPX4* in intestinal LOX1^+^ PMN-MDSCs (*n* = 4 biological replicates). Representative H&E staining (**K**) and statistical analysis of inflammation scores (**L**) in the intestinal samples from different periods of NEC (*n* = 6, scale bar: 50 μm). **M–P** Immunofluorescence staining showing the colocalization of OLFM4 and GPX4 with LOX1 (*n* = 6, scale bar: 50 μm). **Q–U** Correlation analyses between CD3^+^ T cells/mm^2^, as well as the inflammation scores, and OLFM4 and GPX4 expression in intestinal LOX1^+ ‘^PMN-MDSCs (*n* = 30). Data are presented as mean ± SEM. Each dot represents an individual infant. ns, not significant; *p < 0.05, **p < 0.01, ***p < 0.001, ****p < 0.0001. Statistical significance was determined using a Student’s *t* test (**J**), Mann–Whitney test (**I**, **J**), one-way ANOVA (**L,** **N**, **P**), Wilcoxon test (**C,** **D**, **F–H**), or Pearson correlation coefficient (**Q–U**). Post-hoc analyses were performed using Dunnett’s test (**L,** **N**, **P**).

To translate our murine findings to human NEC, we reanalyzed single-cell RNA sequencing (scRNA-seq) data from intestinal tissues of infants with NEC and non-NEC controls [46]. After quality control, we identified the neutrophil cluster based on classic marker genes (Fig. S21A, B). This neutrophil cluster was further regrouped into five major clusters. Strikingly, cluster_1—the most abundant cluster—was significantly reduced in patients with NEC (Fig. 8A, B). Furthermore, both *OLR1* (encoding the human PMN-MDSC marker LOX1) and *OLFM4* were downregulated in cluster_1 neutrophils from infants with NEC (Fig. 8C, D). Analysis of cluster_1 revealed significant enrichment for the ferroptosis pathway and downregulation of key anti-ferroptosis genes, including *ATF4*, *SLC7A11*, and *GPX4* (Fig. 8E–H). We next validated these transcriptomic findings in fresh intestinal tissues. Flow cytometry analysis confirmed a lower proportion of intestinal LOX1^+^ PMN-MDSCs in infants with NEC, accompanied by impaired expression of *OLFM4*, *ATF4*, *SLC7A11*, and *GPX4* in these cells (Fig. 8I, J). These results indicate that intestinal LOX1^+^ PMN-MDSCs and the anti-ferroptosis axis OLFM4**–**ATF4**–**xCT/GPX4 are compromised in infants with NEC.

To establish clinical relevance, we analyzed intestinal tissues across different disease stages. Histological analysis showed that intestinal inflammation became more severe with NEC progression (Figs. 8K, L and S22A, B). Meanwhile, immunofluorescence revealed a marked decrease in the co-localization of OLFM4/GPX4 with intestinal LOX1^+^ cells (Figs. 8M–P and S23A, B). Linear regression analysis revealed that OLFM4 and GPX4 expression in intestinal LOX1^+^ PMN-MDSCs negatively correlated with disease severity, including inflammation scores and CD3^+^ T cells/mm^2^ (Fig. 8Q–T). Moreover, OLFM4 expression displayed a significant positive correlation with GPX4 expression in intestinal LOX1^+^ PMN-MDSCs (Fig. 8U). Intriguingly, mirroring murine findings, fecal I3A levels were significantly lower in NEC infants than in the controls (Fig. S24A). Collectively, these clinical data reveal that human NEC progression is associated with a loss of intestinal LOX1^+^ PMN-MDSCs and impairment of the OLFM4-mediated anti-ferroptosis pathway in these cells.

## Discussion

While circulating PMN-MDSCs have well-established roles in regulating experimental NEC in murine models [21–23], the function and precise regulation underlying iPMN-MDSCs remain poorly defined. Our study reveals that treatment with a probiotic cocktail (*L. reuteri* and *L. rhamnosus*) or their effector metabolite I3A specifically increases iPMN-MDSC abundance by activating the OLFM4-driven anti-ferroptosis pathway, thereby improving IEC function, promoting intestinal barrier repair, and ameliorating NEC.

The therapeutic application of probiotics for NEC is constrained by safety concerns in immunocompromised neonates, underscoring the need to define precise mechanisms [47, 48]. Our findings identify microbial metabolites as promising alternatives for NEC intervention. In particular, I3A, a tryptophan-derived metabolite whose levels are reduced in NEC, recapitulates the beneficial effects of its parental *Lactobacilli* strains. These observations align with accumulating evidence that specific microbial metabolites, rather than live bacteria, mediate key host-microbiota interactions [49], and underscore the potential for developing metabolite-based precision therapeutics for NEC. Future studies employing genetic ablation of the I3A biosynthetic pathway in these *Lactobacillus* strains will be critical to provide direct causal evidence supporting this strategy. Notably, future clinical translation of I3A for human NEC should account for key confounding factors, particularly gestational age and birth weight, which influence intestinal development, immune maturation, and treatment responsiveness in preterm infants.

Of note, I3A treatment retained partial efficacy in *Olfm4*-deficient neonates compared to PBS controls, suggesting the existence of OLFM4-independent pathways. This observation aligns with reports that I3A can suppress ferroptosis through alternative mechanisms such as chelating lipid peroxides and nuclear factor erythroid 2-related factor 2 (NRF2) activation [50, 51]. However, direct comparison between Olfm4-deficient and control littermates showed that the presence of OLFM4 profoundly enhanced I3A-induced ferroptosis resistance and NEC alleviation. This dependency establishes the OLFM4-driven anti-ferroptosis axis as the predominant mechanism underlying I3A-mediated protection.

The biological activity of such metabolites is inherently linked to their tissue distribution. I3A is primarily produced and exerts its effects within the intestinal niche, with minimal systemic exposure [52]. This spatial restriction explains its specific regulatory effect on iPMN-MDSCs rather than their circulating counterparts, illustrating how the localized release of microbial signals ensures precise, site-specific immunomodulation in the gut—an observation also reported for other gut-originated metabolites, such as bile acids [53]. I3A increases iPMN-MDSC abundance by activating the AHR, a signaling pathway critical in regulating intestinal inflammation and highly expressed in both human and murine PMN-MDSCs [54]. Notably, I3A enhances AHR signaling activity, likely acting as a ligand, without altering receptor expression itself. This signaling cascade upregulates OLFM4, which subsequently activates the ERK–ATF4–xCT/GPX4 axis to suppress ferroptosis in iPMN-MDSCs.

Besides, we demonstrate a context-dependent essentiality of GPX4 in maintaining iPMN-MDSC survival. *Gpx4* deletion compromises iPMN-MDSC survival specifically under NEC-induced inflammatory conditions, but not under homeostatic conditions. This finding implies that under physiological conditions, redox balance in iPMN-MDSCs may be maintained by compensatory pathways, such as the FSP1-CoQ10 system or microenvironmental antioxidants [55, 56]. This context-dependent vulnerability aligns with previous reports that inflammatory milieus can render immune cells more susceptible to ferroptosis [14]. Our work identifies OLFM4 as a critical downstream effector of microbial metabolites in iPMN-MDSCs, revealing a novel immune-metabolic adaptation in the neonatal intestine. Future studies should elucidate the mechanism directly coupling AHR signaling to OLFM4 expression and determine whether developmental or disease-specific cues modulate this axis in the neonatal gut.

Notably, iPMN-MDSC activation drives IL-18 production, which we have identified as a direct factor for IEC proliferation in vitro. This model integrates microbial signals, immune function, and tissue repair into a coordinated host response to NEC-induced intestinal inflammation. Further in vivo studies using neutrophil-specific *Il18*-deficient mice are necessary to confirm this immune–epithelial crosstalk axis and will be a focus of our upcoming investigations.

In summary, our study highlights a critical microbiota–iPMN-MDSC axis in the neonatal gut. We identify OLFM4 as a novel anti-ferroptosis regulator in iPMN-MDSCs, demonstrating that its deficiency exacerbates NEC and compromises microbial homeostasis. Treatment with a probiotic cocktail or its effector metabolite I3A activates the OLFM4–ERK–ATF4–xCT/GPX4 axis in iPMN-MDSCs, thereby promoting barrier repair and alleviating NEC. These findings establish a therapeutic framework for NEC via metabolite-based targeting of iPMN-MDSC ferroptosis.

## Materials and methods

### Human subjects

Intestinal and fecal samples of infants aged 1–30-day-old were collected from Guangdong Provincial People’s Hospital of Southern Medical University (Guangzhou, China). The diagnosis of NEC was based on established clinical and radiological criteria, including intestinal dilation, fixed loop, gas in the intestinal wall and portal vein, and abdominal fluid accumulation on X-ray examinations, along with intestinal wall echo, blood perfusion, and free gas on clinical ultrasonography. Samples from the NEC group were obtained during surgery, and the clinical characteristics of these patients are listed in Table S1. The age-matched control group consisted of infants with spontaneous ileal perforation, duodenal atresia, false intestinal obstruction, and Hirschsprung disease (unaffected segments). The clinical characteristics are detailed in Table S2. Infants with pathological jaundice, fever, or other acute diseases were excluded from this study. Written informed consent was obtained from the newborn’s guardians after admission, and the study was approved by the Institutional Ethics Committee of Guangdong Provincial People’s Hospital of Southern Medical University (Approval number: KY2023-099-01).

### Human sample processing and isolation

Primary lamina propria mononuclear cells (LPMCs) were isolated from the intestine as previously described [14, 25]. Fresh intestinal tissues were washed with PBS, cut into 1-cm pieces, and pre-digested with a solution containing 5 mM EDTA (Solarbio, Beijing, China), 1 mM dithiothreitol (Amresco, Solon, OH, USA), and 1% penicillin/streptomycin (Biological Industries, Beit Shemesh, Jerusalem District, Israel) in Hank’s Balanced Salt Solution (HBSS). The pre-digestion was performed with shaking at 37 °C for 45 min. After washing with PBS, the fragments were added into Roswell Park Memorial Institute (RPMI)-1640 medium (Basal Media, Shanghai, China) containing 1 mg/mL collagenase type I (Gibco, Grand Island, NY, USA), 200 U/mL DNase I (Solarbio, Beijing, China), 1% penicillin/streptomycin, and 10% fetal bovine serum (FBS). The mixture was shaken for 45 min at 37 °C. Thereafter, the suspensions were filtered through a 70-μm cell filter, and LPMCs were enriched using 40%/80% Percoll density gradient centrifugation (Cytiva, Marlborough, MA, USA) with acceleration and deceleration set to 9, at 400 × *g* for 25 min. LPMCs were then washed with PBS for further use.

### Human fecal sample processing

Fecal samples from both control and NEC groups, as defined above, were collected. For I3A quantification, 200 mg of each fecal sample was homogenized in 2 mL PBS, sonicated with 15 s on/15 s off cycles for 10 min, and centrifuged at 2000 rpm for 10 min. The supernatant (10 μL) was collected and diluted to 50 μL. I3A levels were measured using a commercial ELISA kit (MEIMAIN, Shanghai, China) according to the manufacturer’s instructions.

### Human histopathological sections

Intestinal histopathological sections of infants with NEC were provided by Guangdong Provincial People’s Hospital of Southern Medical University (Guangzhou, China). These sections were evaluated by the pathologists according to the established grading criteria [21, 40]. The clinical classification of NEC was divided into five grades, which were used as quantitative indices for correlation analysis.

### Histopathological staining of human sections

For immunofluorescence staining, the paraffin sections were deparaffinized, rehydrated, and placed into citric acid retrieval solution (pH 6.0). The sections were then permeabilized with 0.2% Triton X-100 (BBI Life Sciences, Shanghai, China) and blocked with 5% bovine serum albumin (Sigma-Aldrich, Burlington, MA, USA) at 37 °C for 1 h. After washing with PBS, the samples were probed with the primary antibodies at 4 °C overnight, followed by Alexa Fluor488–conjugated rabbit secondary antibody (Abcam, Cambridge, UK) and counterstained with 4’,6-diamidino-2-phenylindole (DAPI, Beyotime, Shanghai, China), respectively. The primary antibodies used in this study were anti-human CD3 (BioLegend, San Diego, CA, USA), anti-mouse/human OLFM4 (Zenbio, Chengdu, Sichuan, China), and anti-mouse/human GPX4 (Abmart, Shanghai, China) antibodies, as detailed in Table S3. For H&E staining, the paraffin sections were deparaffinized, rehydrated, and stained with hematoxylin and eosin (Servicebio, Wuhan, Hubei, China). All the sections were imaged under a microscope (Nikon, Shinagawa-ku, Tokyo, Japan) and analyzed using NIS viewer software.

### Mouse strains

Seven-day-old C57BL/6 wild-type mice were purchased from the Center of Laboratory Animals of Southern Medical University. S100a8^cre^ mice were generously provided by Cyagen Biosciences, Inc. Olfm4^fl/fl^ mice were supplied by Professor Zhipeng Zou of Southern Medical University (Guangzhou, China). Olfm4^fl/fl^S100a8^cre^ neonates were generated by crossing Olfm4^fl/fl^S100a8^cre^ males with Olfm4^fl/fl^ females. Gpx4^fl/fl^ mice were purchased from Shanghai Model Organisms Center Inc., and Gpx4^fl/fl^S100a8^cre^ neonates were created by crossing Gpx4^fl/fl^S100a8^cre^ males with Gpx4^fl/fl^ females. *Il18*^–*/*–^ mice were generously provided by Professor Shu Zhu of the University of Science and Technology of China (Hefei, China). All mice had free access to tap water and standard rodent particle food and were maintained in a specific-pathogen-free environment with 55% ± 5% humidity, at a temperature of 23 ± 2 °C, and under a 12-h light/dark cycle. Both sexes were included in the study. Mice were randomly assigned to the experimental groups based on initial body weight to ensure similar weight distribution across groups. All experimental protocols were approved by the Institutional Animal Care and Use Committee of Southern Medical University Experimental Animal Ethics Committee (Approval number: SMUL2019130).

### Mouse models of NEC

The experimental NEC model was established as previously described [25]. Seven-day-old neonates were fed with formula via oral gavage four times daily (50–150 μL per feeding) at 6:00, 12:00, 18:00, and 24:00. The formula consisted of Infant Formula Powder (Similac Advance, Columbus, OH, USA) and Puppy Milk Replacer Powder (PetAg, Hampshire, IL, USA) in a 2:1 ratio. Pups were stimulated to urinate before each gavage. During the modeling process, pups underwent two cycles of hypoxia (5% O_2_, 95% N_2_) for 90 s and cold stress (4 °C) for 8–10 min each day. All the neonates were housed at room temperature during NEC induction. The number and weight of the surviving neonates were recorded daily.

### Preparation and treatment of the probiotic cocktail

*L. reuteri* (strain CICC6132) and *L. rhamnosus* (strain ATCC53103) were provided by Professor Hongying Fan of Southern Medical University (Guangzhou, China). Each strain was anaerobically cultured in deMan-Rogosa-Sharpe medium at 37 °C for 16–20 h. Bacterial concentration was determined by measuring the optical density at 600 nm (OD_600_) and converting to colony-forming unit (CFU) per mL using a pre-established standard curve. The final suspensions reached an OD_600_ of 1.2–1.3, corresponding to approximately 5 × 10^9^ CFU/mL. The required volumes to achieve 5 × 10^9^ CFU per strain were calculated and combined, resulting in a total of 1 × 10^10^ CFU in a 1:1 CFU ratio. This probiotic cocktail was then thoroughly blended into the formula for the entire 4–day NEC induction. For treatment, seven-day-old neonates received this probiotic-supplemented formula daily by oral gavage, delivering a total dose of 1 × 10^10^ CFU per pup during NEC induction. Control pups received formula blended with an equal volume of PBS [57, 58]. Mice were randomly assigned to the groups, and both sexes were included in the study. The number and weight of the surviving neonates were recorded daily. On day 5 of NEC induction, mice were euthanized, and their intestines, feces, plasma, and spleen were harvested for further use and analysis.

### Treatment with a broad-spectrum antibiotic

To deplete the gut microbiota via antibiotic treatment (ABX), five-day-old littermate neonates received a broad-spectrum antibiotic or a vehicle control by oral gavage for 48 h [40, 41]. The antibiotic contained metronidazole, ampicillin, neomycin sulfate, and vancomycin, each at a dose of 83 mg/kg/day, and the mixture was blended into the formula. Following this pretreatment, neonates were then subjected to NEC induction as described above. Both sexes were included in the study. Mice were randomly assigned to the experimental groups based on initial body weight to ensure similar weight distribution across groups. The number and weight of the surviving neonates were recorded daily. On day 5 of NEC induction, mice were euthanized, and their intestinal tissues, feces, plasma, and spleen were harvested for further use and analysis.

### Treatment or intervention in vivo

ISRIB was dissolved in a 1:8:1 mixture of DMSO, PEG400 (Sigma-Aldrich, Burlington, MA, USA), and Tween 80 (Sigma-Aldrich, Burlington, MA, USA), and the same mixture of the solvent was used as the vehicle control. Seven-day-old pups received 0.25 mg/kg/day ISRIB or the vehicle control once daily by oral gavage during NEC induction [44, 45]. For Fer-1 treatment, seven-day-old neonates received either 1 mg/kg/day Fer-1 (Selleck, Houston, TX, USA) or an equivalent volume of the vehicle control once daily via oral gavage during NEC induction [14]. For in vivo I3A administration, seven-day-old pups received 25 mg/kg/day I3A (InvivoChem, Libertyville, IL, USA) by oral gavage once daily during NEC induction [52]; control pups received an equivalent volume of PBS. For AHR inhibition, seven-day-old pups were intraperitoneally injected with 4 μg/day CH-223191 (Selleck, Houston, TX, USA) dissolved in DMSO once daily during NEC induction; control pups were injected with an equivalent volume of vehicle [59]. Both sexes were included in the study. Mice were randomly assigned to the experimental groups based on initial body weight to ensure similar weight distribution across groups. The number and weight of the surviving neonates were recorded daily. On day 5 of NEC induction, mice were euthanized, and their intestines, feces, plasma, and spleen were harvested for further use and analysis.

### Mouse sample processing and isolation

For LPMC and IEC isolation, the murine intestines were washed with PBS, cut into 1-cm pieces, and transferred to HBSS buffer containing 5 mM EDTA (Solarbio, Beijing, China), 1 mM dithiothreitol (Amresco, Solon, OH, USA), and 1% penicillin/streptomycin (Biological Industries, Beit Shemesh, Jerusalem District, Israel) for a 45-min incubation at 37 °C. After washing with PBS, the intestinal tissues were digested in RPMI-1640 medium (Basal Media, Shanghai, China) containing 1 mg/mL collagenase type I (Solarbio, Beijing, China), 200 U/mL DNase I (Solarbio, Beijing, China), 1% penicillin/streptomycin, and 10% FBS for 45 min at 37 °C. After digestion and washing, IECs were prepared for further use. LPMCs were enriched by centrifugation using 40%/80% Percoll (Cytiva, Marlborough, MA, USA) with acceleration and deceleration set to 9 at 400 × *g* for 25 min. After centrifugation, the white membrane layer was collected and washed for subsequent testing.

### Cell purification

Human PMN-MDSCs were labeled with LOX1-PE (eBioscience, San Diego, CA, USA) and sorted using the EasySep^TM^ Human PE Positive Selection Kit II (StemCell Technologies, Vancouver, BC, Canada) according to the manufacturer’s instructions. Murine PMN-MDSCs and IECs were labeled with Ly6G-PE (eBioscience, San Diego, CA, USA) and CD326-PE (eBioscience, San Diego, CA, USA), respectively, and purified using the EasySep^TM^ Mouse PE Positive Selection Kit II (StemCell Technologies, Vancouver, BC, Canada) according to the manufacturer’s instructions. The purity of the sorted cells was assessed by flow cytometry, ensuring at least 70% positivity for the target marker.

For flow cytometric sorting, iPMN-MDSCs were isolated based on a Live/Dead^−^CD45^+^CD11b^+^Ly6G^+^Ly6C^−/low^ gating strategy using a CytoFLEX STR flow cytometer (Beckman Coulter Life Sciences, Indianapolis, IN, USA). The gating strategies and purified efficiency are described in Fig. S25. The antibodies used are listed in Table S3.

### Cell adoptive transfer

iPMN-MDSCs were isolated from wild-type neonates after NEC induction, following the processing and isolation methods described above. Prior to NEC induction, *Il18*^−/−^ recipients received an intraperitoneal injection of 1 × 10^5^ iPMN-MDSCs (resuspended in 50 μL PBS) and then underwent the 4-day NEC induction protocol as described above [25]. Recipients from the same litter were used to control for litter-specific effects and were randomly assigned to experimental groups.

### Intestinal permeability assay

Intestinal permeability was evaluated as previously reported, with minor modifications [25]. NEC-induced pups were treated with 2 ng/kg of fluorescein isothiocyanate-dextran 70 (Sigma-Aldrich, Burlington, MA, USA) via oral gavage 4 h prior to euthanasia. Thereafter, the blood was collected and centrifuged at 2000 rpm for 8 min. Plasma was obtained (approximately 10 μL), and fluorescence was measured at excitation/emission wavelengths of 490/520 nm using a microplate reader (Thermo Fisher Scientific, Waltham, MA, USA). The concentration of FD70 was calculated based on a pre-established standard curve using serial dilutions of FD70 in PBS (0.1–10 mg/mL).

### iPMN-MDSC or IEC culture and supernatant assay

iPMN-MDSCs or IECs were obtained as described above and cultured at a density of 1–2 × 10^5^ cells/well in 96-well U-bottom plates with RPMI-1640 medium containing 10% FBS at 37 °C under a 5% CO_2_ environment. After 8 h, the supernatant was collected, and IL-18 levels were quantified using an ELISA kit (Dogesce, Beijing, China) according to the manufacturer’s instructions.

### IEC culture and proliferation assay

The culture supernatants from iPMN-MDSCs were obtained as described above. IECs were sorted from neonates under physiological conditions and cultured in 96-well U-bottom plates at a density of 5 × 10^5^ cells/well in RPMI-1640 medium containing 10% FBS at 37 °C under a 5% CO_2_ environment as follows: (1) IECs only, (2) IECs with 50 μL iPMN-MDSC supernatant, and (3) IECs with iPMN-MDSC supernatant supplemented with 2 μg/mL IL-18 mAb (Selleck, Houston, TX, USA). After 24 h, IECs were collected, stained with anti-Ki-67 antibody, and the proliferation was analyzed using a CytoFLEX flow cytometer (Beckman Coulter, Brea, CA, USA).

### In vitro co-culture of iPMN-MDSCs and RSL3

iPMN-MDSCs were isolated from neonates under physiological conditions or after NEC induction. Sorted PMN-MDSCs (at a density of 2 × 10^5^ cells/well) were seeded into 96-well plates in RPMI-1640 medium containing 10% FBS and treated with the GPX4-specific inhibitor RSL3 at the concentrations of 0, 2, 4, and 8 μM for 16 h at 37 °C under a 5% CO_2_ atmosphere. Following treatments, cells were harvested for further analysis [31].

### In vitro co-culture of iPMN-MDSCs and I3A, I3C, and IPA

Sorted iPMN-MDSCs (at a density of 2 × 10^5^ cells/well) were seeded into 96-well plates in RPMI-1640 medium containing 10% FBS and co-cultured with I3A, I3C, or IPA (InvivoChem, Libertyville, IL, USA) at the concentrations of 0 and 200 μM in vitro. After 12 h of culture, cells were collected and measured using a CytoFLEX flow cytometer (Beckman Coulter, Brea, CA, USA).

### In vitro co-culture of bacterial and I3A

*L. reuteri* and *L. rhamnosus* were adjusted to an OD_600_ of 0.200 and then treated with 200 μM I3A or an equivalent volume of PBS. These cultures were anaerobically incubated in deMan-Rogosa-Sharpe (MRS) medium at 37 °C for 20–24 h. Bacterial growth was monitored by measuring the OD_600_ every 4 h, and growth curves were generated based on the OD_600_ values measured at each time point [60].

### RNA isolation and quantitative real-time PCR

Total RNA was extracted from the intestinal tissues or cells using TRIzol reagent (ECOTOP SCIENTIFIC, Guangzhou, China), and cDNA was synthesized using the StarScript II First-strand cDNA Synthesis kit (GenStar, Beijing, China). Thereafter, quantitative real-time PCR was performed using RealStar Green Power Mixture (GenStar, Beijing, China) on a QuantStudio 6 Flex system (Applied Biosystems, Foster City, CA, USA). The relative expression of the target gene was normalized to the expression of *β-actin*. The primers used in this study are listed in Table S4.

### Chromatin immunoprecipitation (ChIP) assay

ChIP assay was performed as previously described, with minor modifications [25]. In brief, iPMN-MDSCs were cross-linked with 1% formaldehyde solution, terminated with 0.125 mol/L glycine, and the DNA fragments were interrupted using ultrasound with 10 s on/20 s off cycles for 12 min. The cell lysates were immunoprecipitated with anti-ATF4 (Cell Signaling Technology, Danvers, MA, USA) or anti-IgG (Cell Signaling Technology, Danvers, MA, USA) antibodies overnight at 4 °C. The antibody-chromatin complex was then collected using protein A/G-agarose (Thermo Fisher Scientific, Waltham, MA, USA). All the DNA samples were purified after de-crosslinking and quantified using PCR. The primers used for amplification are listed in Table S5. The first 10% of the immunoprecipitation lysate was used as the input control, and the ATF4 gene DNA fragment enrichment was normalized to this input control.

### Bacterial abundance assay

Total genomic DNA was extracted from the fecal samples as previously described, with minor modifications [61]. Briefly, fecal samples were collected into a 1.5 mL sterile tube containing 100 mM pH 8.0 Tris-HCl, 500 mM NaCl, 50 mM sterile EDTA, and 10% sterile SDS and stored at -20 °C until DNA extraction. Samples were incubated at 65 °C for 2 h, placed in an ice bath for 20 min, and then centrifuged at 12,000 rpm for 10 min. The supernatant was transferred to a new 1.5 mL Eppendorf tube, mixed with 400 μL of a mixture of Tris-saturated phenol and chloroform for 20 min, and centrifuged at 12,000 rpm for 10 min. The supernatant was then transferred to a new tube, mixed with an equal volume of isopropanol, and incubated at –20 °C overnight. The samples were centrifuged at 12,000 rpm for 10 min and allowed to air-dry for 15 min. DNA pellets were resuspended and stored at –20 °C. DNA quality and concentration were quantified using a Nanodrop spectrophotometer (Thermo Fisher Scientific, Waltham, MA, USA). All procedures were performed on a clean bench under aseptic conditions. The relative abundance of bacterial DNA was determined using the comparative Ct (2^−ΔΔCT^) method and normalized to the 16S rDNA level. The primers for the variable regions of the bacterial 16S rDNA gene sequence used in this study are listed in Table S6.

### Gut microbiota depletion analysis

Fecal samples were collected from SPF and ABX-treated neonates, weighed, and resuspended in 2 mL sterile PBS within a sterile 1.5-mL tube. Samples were centrifuged at 250 × *g* for 5 min, and the supernatants were collected. Aliquots (approximately 50 μL) were then plated on Lysogeny Broth (LB) agar plates and incubated aerobically at 37 °C for 24 h [62].

For fecal microbial load analysis, fecal samples were collected and immediately snap-frozen in liquid nitrogen before storage at –80 °C. Samples were weighed and then subjected to DNA extraction and bacterial abundance assay as described above. Fecal microbial load was normalized to the SPF group.

### Western blotting analysis

For total protein extraction, the intestinal tissues were ground into powder, mixed with RIPA buffer (Beyotime, Shanghai, China) containing a protease cocktail, and lysed at 4 °C for 30 min. Nuclear and cytoplasmic proteins were extracted according to the guidelines of the nuclear and cytoplasmic protein extraction kit (Beyotime, Shanghai, China). Protein concentrations were measured using the Bradford assay. Thereafter, proteins were separated using 8% SDS-PAGE and transferred onto a polyvinylidene difluoride membrane (Millipore, Burlington, MA, USA). The membrane was blocked with 5% nonfat dry milk and incubated with the primary antibodies at 4 °C overnight. After washing, the blot was labeled with horseradish peroxidase-conjugated secondary antibodies at room temperature for 1 h and detected with a chemiluminescent kit (Millipore, Burlington, MA, USA). The full-length, uncropped original western blots used in this study are provided in the Supplementary Information. The primary and secondary antibodies used in this study are listed in Table S3.

### H&E staining

Small intestines were fixed in 4% paraformaldehyde overnight, paraffin-embedded, and sliced into 3–5-μm-thick sections. After deparaffinization and rehydration, the sections were stained with hematoxylin and eosin (Servicebio, Wuhan, Hubei, China) and imaged under a microscope (Nikon, Shinagawa-ku, Tokyo, Japan).

An intestinal inflammation score was determined by two independent observers using a scoring system ranging from zero to four, where grade zero = no villus damage (none); grade one = sloughing of distal villus epithelial cells (mild); grade two = moderate sloughing of the submucosa and/or lamina propria (moderate); grade three = loss of the entire villus with preservation of the crypts (severe); grade four = transmural necrosis (necrosis). Mice with a pathological injury score above grade two were considered to exhibit NEC [25, 40]. The observer was blinded to group allocation during estimation. Group assignments were concealed until all data analyses were completed.

### Flow cytometry

For surface staining, single-cell suspensions (1 × 10^6^) were pre-incubated with 0.5 μg CD16/32 antibody (BioLegend, San Diego, CA, USA) to block Fc receptor binding, and then stained with Live/Dead and surface antibodies in 50 μL of FACS buffer at 4 °C in the dark for 30 min. The gating strategy for mice was as follows: PMN-MDSCs, live CD45^+^CD11b^+^ Ly6G^+^Ly6C^−/low^; M-MDSCs, live CD45^+^CD11b^+^Ly6G^−^Ly6C^high^; IECs, live CD45^−^ CD326^+^. The gating strategy for human was as follows: PMN-MDSCs, live CD45^+^ CD15^+^CD14^−^LOX1^+^. For intracellular staining of target proteins, 1 × 10^6^ cells were fixed and permeabilized using a Foxp3/Transcription Factor staining buffer set (eBioscience, San Diego, CA, USA) at 4 °C for 30 min. After washing, the samples were stained with the target proteins.

Flow cytometry analyses were performed on a LSR Fortessa flow cytometer (BD Biosciences, San Jose, CA, USA) and a CytoFLEX flow cytometer (Beckman Coulter, Brea, CA, USA). All stained samples were analyzed within 2 h. The data were analyzed using FlowJo software (v10.2) and CytExpert software, and the isotype was used to set the gate. The gating strategies are shown in Fig. S25. The antibodies for flow cytometric analyses are listed in Table S3.

### Cytosolic ROS and lipid ROS assays

Cytosolic ROS and lipid ROS assays were performed as previously described [14]. In brief, 1 × 10^6^ cells were resuspended in 500 µL of RPMI-1640 medium (Basal Media, Shanghai, China) and incubated with 5 µM DCFDA (cytosolic ROS) (Invitrogen, Carlsbad, California, USA) or 2 µM C11-BODIPY 581/591 (lipid ROS) (Invitrogen, Carlsbad, California, USA) at 37 °C with 5% CO_2_ for 30 min, followed by surface marker staining. The samples were analyzed using a CytoFLEX flow cytometer (Beckman Coulter, Brea, CA, USA) and analyzed using CytExpert software.

### Cell apoptosis and proliferation assays

First, 1 × 10^6^ cells were stained with surface antibodies and washed with PBS. Cells were then resuspended in a cocktail containing 200 µL of Annexin V Binding Buffer (BioLegend, San Diego, CA, USA), Annexin V-APC (BioLegend, San Diego, CA, USA), and 7-AAD Viability Staining Solution (BioLegend, San Diego, CA, USA), and incubated for 15–20 min in the dark at 37 °C. All stained samples were analyzed within 15 min. For the proliferation assay, surface-stained cells were fixed and permeabilized using a Foxp3/Transcription Factor staining buffer set (eBioscience, San Diego, CA, USA) and incubated with Ki67-APC antibodies (BioLegend, San Diego, CA, USA) at 4 °C for 30 min. The suspensions were detected using a CytoFLEX flow cytometer (Beckman Coulter, Brea, CA, USA) and analyzed using CytExpert software.

### Enzyme-linked immunosorbent assay (ELISA)

To quantify I3A, I3C, and IPA levels in feces, intestinal tissue, spleen, and isolated cells, samples were homogenized in 2 mL PBS, sonicated with 10 s on/20 s off cycles for 10 min, centrifuged at 2000 rpm for 8 min, and the supernatants were collected for analysis [63]. For plasma preparation, whole blood was centrifuged at 2000 rpm for 10 min, and the resulting plasma (approximately 10 µL) was diluted 1:4 with PBS prior to analysis. IL-18 levels were measured in cell culture supernatants after a 24-h culture. All assays were performed according to the manufacturer’s instructions for the respective ELISA kits.

### RNA sequencing (RNA-seq) analysis

RNA-seq was performed using the Illumina NovaSeq X Plus at Gene Denovo Biotechnology Co., Ltd. (Guangzhou, China). Differential expression analysis was performed using the R package DESeq2 (v.1.44.0), with a fold change of ≥ 1.5 and a false discovery rate (FDR) of < 0.05 used as the thresholds for significance to identify the differentially expressed genes. Kyoto Encyclopedia of Genes and Genomes and Gene Ontology analyses of the differentially expressed genes were performed using the R package clusterProfiler (v4.12.6).

### Single-cell RNA sequencing (scRNA-seq) analysis

scRNA-seq data of the human samples were downloaded from the Zenodo repository (10.5281/zenodo.5813397) [46]. The processed data were analyzed using the R package Seurat (v5.1.0) with t-distributed stochastic neighbor embedding for gene expression visualization. The differentially expressed genes with |logFC | > 0.25 and adjusted *p* value < 0.05 were used for Kyoto Encyclopedia of Genes and Genomes signaling pathway analysis. Feature and violin plots were created using the FeaturePlot and VlnPlot functions from the R Seurat package. Significant differences were analyzed using ggsignif (v 0.6.4).

### Liquid chromatography-mass spectrometry (LC-MS/MS) analysis of tryptophan metabolites

Tryptophan metabolites in the fecal samples were profiled by LC-MS/MS. Feces were collected from neonatal mice subjected to NEC induction that received either a vehicle control or the probiotic cocktail. Approximately 20 mg of each sample was transferred to a centrifuge tube, followed by the addition of 500 μL methanol and 20 μL of an internal standard working solution (250 ng/mL). The mixture was vortexed for 3 min and incubated at –20 °C for 30 min to precipitate proteins. Samples were then centrifuged at 12,000 rpm for 10 min at 4 °C, and 250 μL of the supernatant was transferred to a new tube. After a second centrifugation under the same conditions, 150 μL of the clarified supernatant was transferred to an autosampler vial and stored at –20 °C until LC-MS/MS analysis. LC-MS/MS analysis was performed at Wuhan Metware Biotechnology Co., Ltd. (Wuhan, China) using ultra-performance liquid chromatography (ExionLC^TM^ AD, SCIEX) and tandem mass spectrometry (QTRAP 6500 + , SCIEX). Data analysis was supported by the Metware Cloud platform (https://cloud.metware.cn/#/home). Tryptophan metabolite levels were quantified via external calibration curves and normalized to internal standards. Analytical stability was monitored by periodic injection of pooled quality-control (QC) samples. Data acquisition and peak integration were conducted using MultiQuant (v3.0.3). Final metabolite levels are presented as absolute concentrations normalized per 20 mg of feces.

### 16S ribosomal RNA (rRNA) sequencing analysis

Murine fecal DNA extraction, PCR amplification, rRNA pyrosequencing, and DNA library sequencing were performed by the Gene Denovo Biotechnology Co., Ltd. (Guangzhou, China) using the Illumina NovaSeq 6000 and PacBio Revivo platforms according to standard protocols. Bioinformatic analysis was performed using Omismart, a real-time interactive online platform for data analysis (http://www.omicsmart.com). Quality filtering of the raw sequences was performed following the Vsearch v2.13.3 quality-controlled process, and operational taxonomic units were clustered at a 97% similarity threshold. α-diversity indices (Ace, Chao1, Shannon, and Simpson) were calculated to assess species diversity and richness.

### Statistical analysis

Data are presented as mean ± SEM unless stated otherwise. Statistical significance is defined as *p* < 0.05, and “ns” indicates non-significant results in figures. Sample sizes are determined based on previous studies using similar NEC models to ensure reproducibility [21–25]. All animal experiments were performed with at least two independent replicates, and each dot in the graphs represents a mouse from a separate litter. For human samples, each dot represents an individual infant. Detailed Statistical methods for each experiment are provided in the corresponding figure legends. All data were analyzed using GraphPad Prism software (v10.1.2). Prior to statistical analysis, normality and homoscedasticity were evaluated using the Shapiro-Wilk test and Levene’s test, respectively; the results determined the use of parametric or non-parametric methods. For two-group comparisons, significance was assessed using an unpaired Student’s *t*-test when assumption were met; otherwise, a Mann–Whitney test (unpaired) or Wilcoxon signed-rank (paired) tests were applied. For multiple-group comparisons, one-way ANOVA followed by Tukey’s test (equal variances) or Dunnett’s test (unequal variances) was used for parametric data. The survival rate was analyzed using a log-rank test. Clinical correlation between two variables was assessed using Pearson’s correlation coefficient.

## Supplementary information

SUPPLEMENTAL MATERIAL

Original Data

## Data Availability

All data associated with this study are available within the main text or the Supplementary Information files. RNA-sequencing data, 16S ribosomal RNA-sequencing data, and liquid chromatography-mass spectrometry data generated in this study are available at 10.5281/zenodo.15239282, 10.5281/zenodo.15234720, 10.5281/zenodo.15234605, 10.5281/zenodo.18620006, and 10.5281/zenodo.18620156. Source data are provided along with this paper.
