# Supplementary material for: Probiotic interventions maintain intestinal barrier function and alleviate necrotizing enterocolitis by inhibiting ferroptosis in intestinal PMN-MDSCs
Source: Cell Death Dis. 2026 May 15;17(1):623. doi: 10.1038/s41419-026-08869-w (PMC13346608; doi:10.1038/s41419-026-08869-w)
Supplement: Supplementary file 1 — SUPPLEMENTAL MATERIAL [file 41419_2026_8869_MOESM1_ESM.docx]

**Supplementary Material**

**Supplementary Figures and Figure Legends**

**
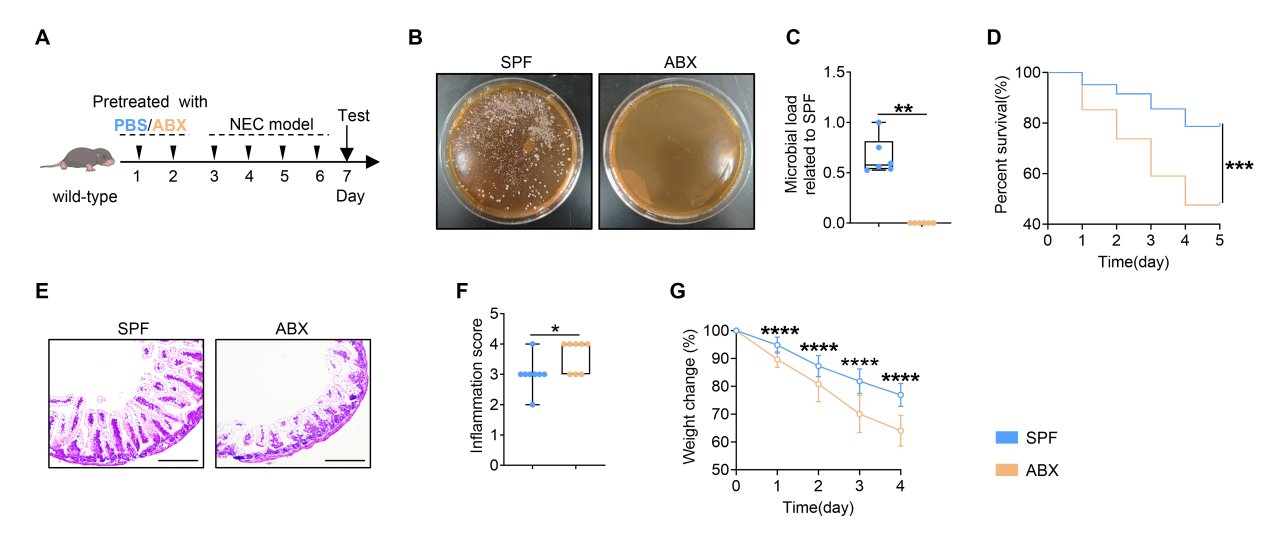
**

**Fig. S1** **Depletion of the gut microbiota reduces neonatal survival and aggravates intestinal inflammation. A** Five**-**day-old wild-type pups were pretreated with a vehicle control or a broad-spectrum antibiotic (ABX; 85 mg/kg/day metronidazole, ampicillin, neomycin sulfate, and vancomycin) for 48 h prior to NEC induction. **B**, **C** Efficacy of gut microbiota depletion (*n* = 6). **D** The survival rates of each group (*n* = 40 and 59). **E**, **F** Representative hematoxylin-eosin (H&E) staining of the intestine (E) and statistical analysis of inflammation scores (F, *n* = 8, scale bar: 50 μm). **G** Statistical analysis of weight change (*n* = 29 and 27). Data are presented as mean ± SEM. Each symbol represents one pup in a litter. ns, not significant; **p* <0.05, ***p* < 0.01, ****p* < 0.001, *****p* < 0.0001. Statistical significance was determined using a Student’s *t-*test (**F** and **G**), Mann–Whitney test (**C**), or log-rank (Mantel–Cox) test (**D**).


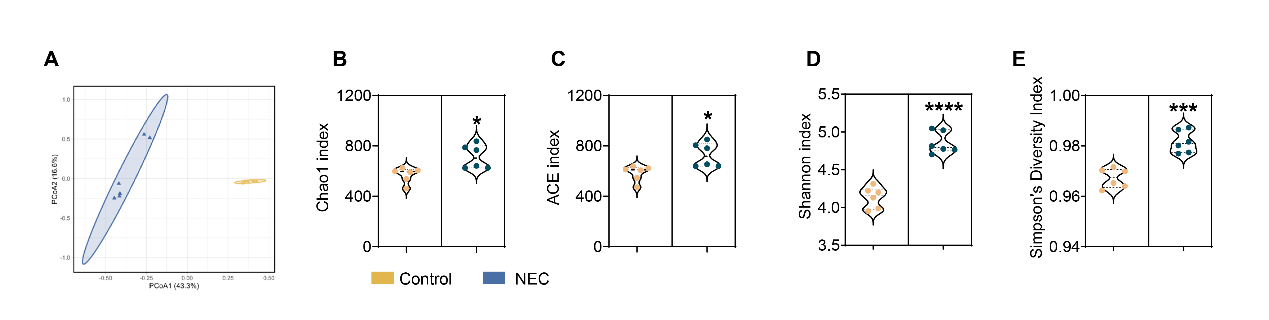


**Fig. S2 The intestinal microbiota is altered following NEC induction. A** Principal component analysis of the intestinal microbiota in control and NEC neonates (*n* = 6). **B–E** α-diversity indices of the intestinal microbiota, including Chao1 (B), ACE (C), Shannon (D), and Simpson’s (E) (*n* = 6). Data are presented as mean ± SEM. Each symbol represents one pup in a litter. ns, not significant; **p* <0.05, ***p* < 0.01, ****p* < 0.001, *****p* < 0.0001. Statistical significance was determined using a Student’s *t-*test (**B–E**).


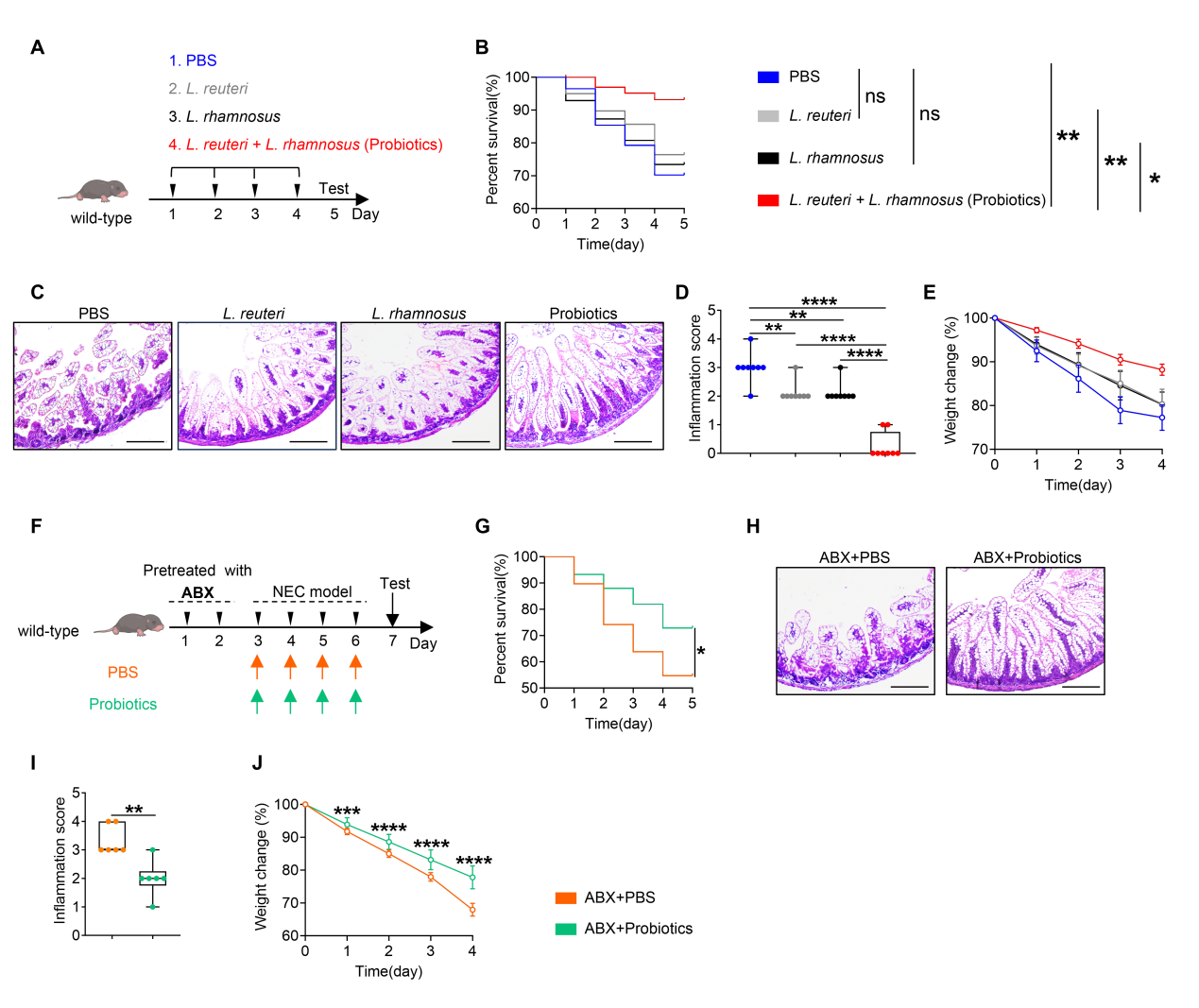


**Fig. S3 The combined treatment with *L. reuteri* and *L. rhamnosus* alleviates NEC independently of the resident gut microbiota. A** Seven-day-old wild-type pups received vehicle control, *Lactobacillus* (*L.*) *reuteri* , *L. rhamnosus*, or the combination of both strains by oral gavage during NEC induction. **B** The survival rates of each group (*n* = 45, 41, 38, and 45). **C**, **D** Representative H&E staining of the intestine (C) and statistical analysis of inflammation scores (D, *n* = 8, scale bar: 50 μm). **E** Statistical analysis of weight change (*n* = 27, 29, 26, and 40). **F** Five-day-old wild-type pups were pretreated with a broad-spectrum antibiotic for 48 h, followed by a vehicle control or the probiotic cocktail during NEC induction. **G** The survival rates of each group (*n* = 53 and 42). **H**, **I** Representative H&E staining of the intestine (H) and statistical analysis of inflammation scores (I, *n* = 6, scale bar: 50 μm). **J** Statistical analysis of weight change (*n* = 27 and 28). Data are presented as mean ± SEM. Each symbol represents one pup in a litter. ns, not significant; **p* <0.05, ***p* < 0.01, ****p* < 0.001, *****p* < 0.0001. Statistical significance was determined using a Student’s *t-*test (**I** and **J**), one-way ANOVA (**D**), or log-rank (Mantel–Cox) test (**B** and **G**). Post-hoc analyses were performed using Tukey’s test (**D**).

**
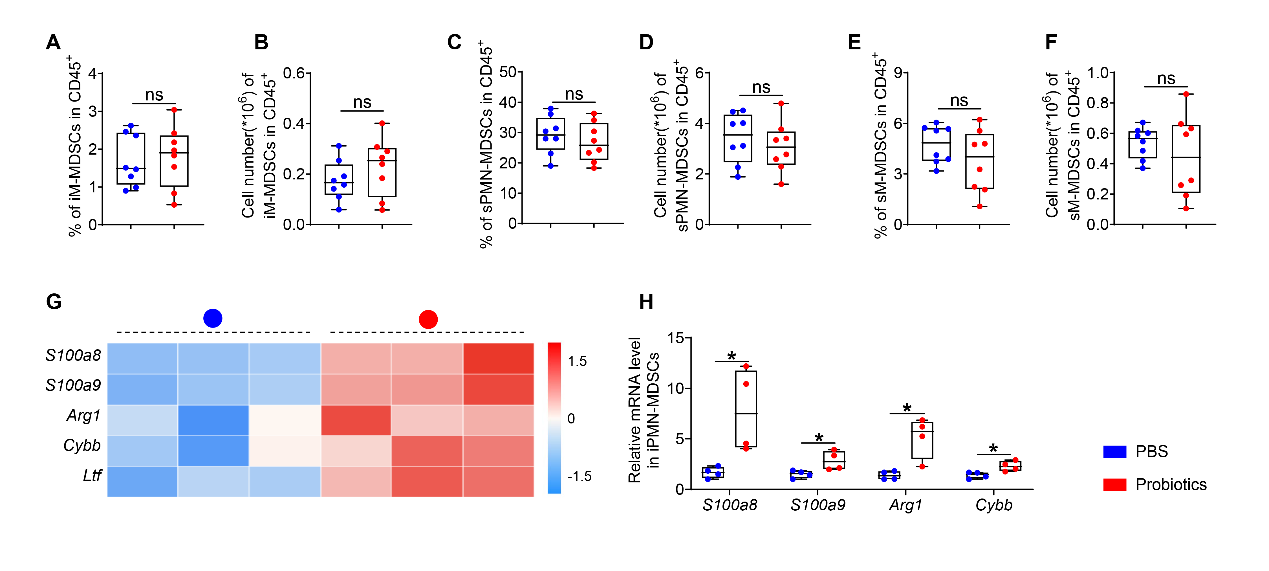
**

**Fig. S4 Probiotic treatment upregulates PMN-MDSC-related genes without altering the levels of iM-MDSCs or splenic MDSCs. A**, **B** Percentage (A) and cell number (B) of intestinal M-MDSCs (iM-MDSCs) among CD45^+^ cells (*n* = 8). **C**, **D** Percentage (C) and cell number (D) of splenic PMN-MDSCs (sPMN-MDSCs) among CD45^+^ cells (*n* = 8). **E**, **F** Percentage (E) and cell number (F) of splenic M-MDSCs (sM-MDSCs) among CD45^+^ cells (*n* = 8). **G** Heatmap of PMN-MDSC-related genes (*n* = 3). **H** mRNA expression levels of *S100a8*, *S100a9*, *Arg1*, and *Cybb* in intestinal PMN-MDSCs (iPMN-MDSCs) (*n* = 4 biological replicates). Data are presented as mean ± SEM. Each symbol represents one pup in a litter. ns, not significant; **p* <0.05, ***p* < 0.01, ****p* < 0.001, *****p* < 0.0001. Statistical significance was determined using a Student’s *t-*test (**A–F** and **H**) or Mann–Whitney test (**H**).

**
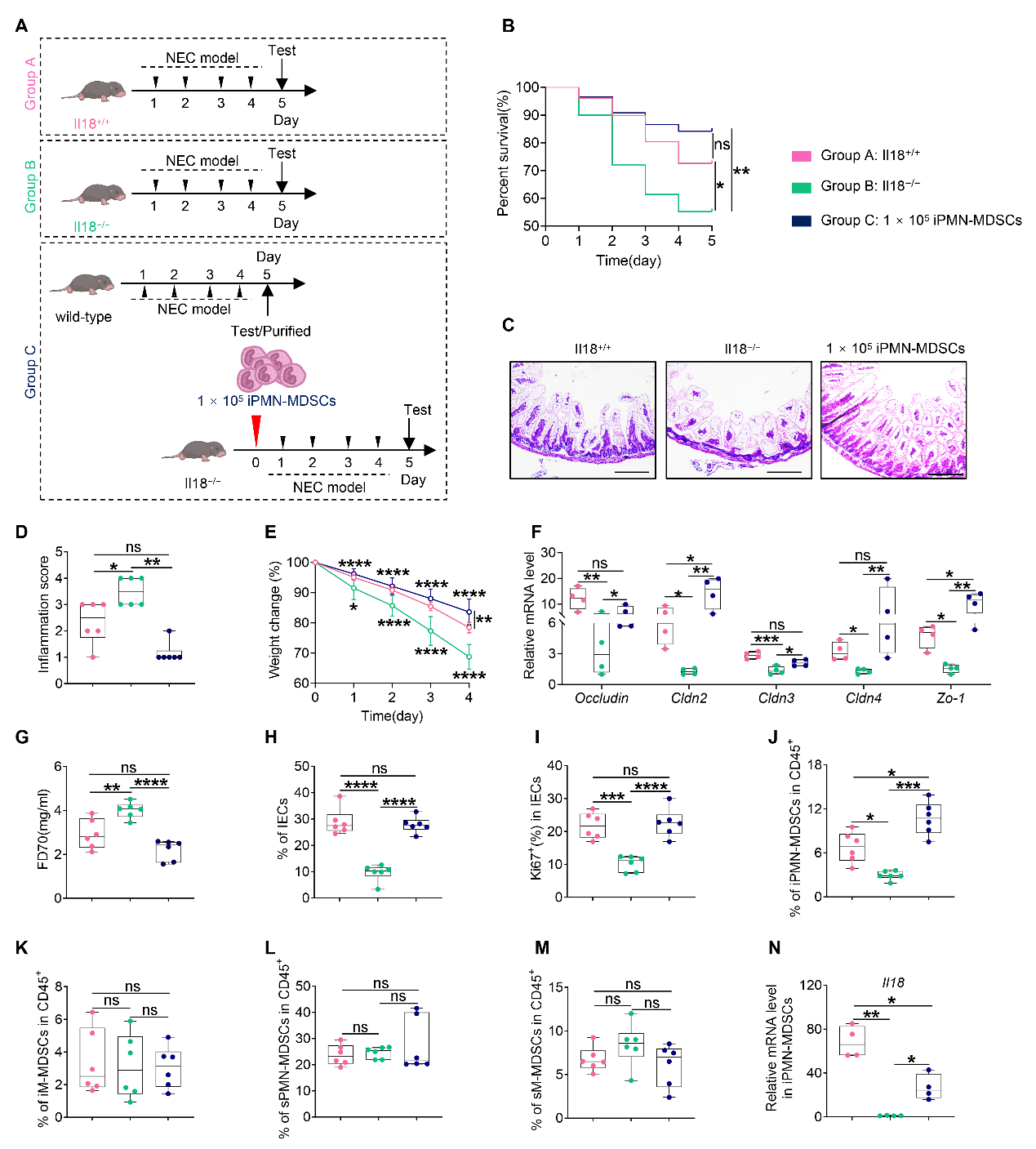
**

**Fig. S5 *Il18* deficiency exacerbates NEC severity, and adoptive transfer of iPMN-MDSCs alleviates the aggravated NEC phenotypes. A** Schematic of NEC induction in *Il18*^+/+^ (group A) and *Il18*^–/–^ (group B) neonates. For adoptive transfer experiment, *Il18*^–/–^ recipients received an intraperitoneal transfer of iPMN-MDSCs prior to NEC induction (group C). **B** The survival rates of each group (*n* = 40, 34, and 44). **C**, **D** Representative H&E staining of the intestine (C) and statistical analysis of inflammation scores (D, *n* = 6, scale bar: 50 μm). **E** Statistical analysis of weight change (*n* = 28, 17, and 32). **F** mRNA expression levels of tight junction (TJ) proteins in the intestine (*n* = 4 biological replicates). **G** Intestinal permeability was assessed by fluorescein isothiocyanate-dextran 70 (FD70) levels (*n* = 6). **H**, **I** Percentage (H) and proliferation (I) of intestinal epithelial cells (IECs) (*n* = 6). **J**, **K** Percentage of iPMN-MDSCs (J) and iM-MDSCs (K) among CD45^+^ cells (*n* = 6). **L**, **M** Percentage of sPMN-MDSCs (L) and sM-MDSCs (M) among CD45^+^ cells (*n* = 6). **N** mRNA expression levels of *Il18* in iPMN-MDSCs (*n* = 4 biological replicates). Data are presented as mean ± SEM. Each symbol represents one pup in a litter. ns, not significant; **p* <0.05, ***p* < 0.01, ****p* < 0.001, *****p* < 0.0001. Statistical significance was determined using a one-way ANOVA (**D–N**) or log-rank (Mantel–Cox) test (**B**). Post-hoc analyses were performed using Tukey’s test (**D–I** and **K–M**) or Dunnett’ s T3 test (**J** and **N**).


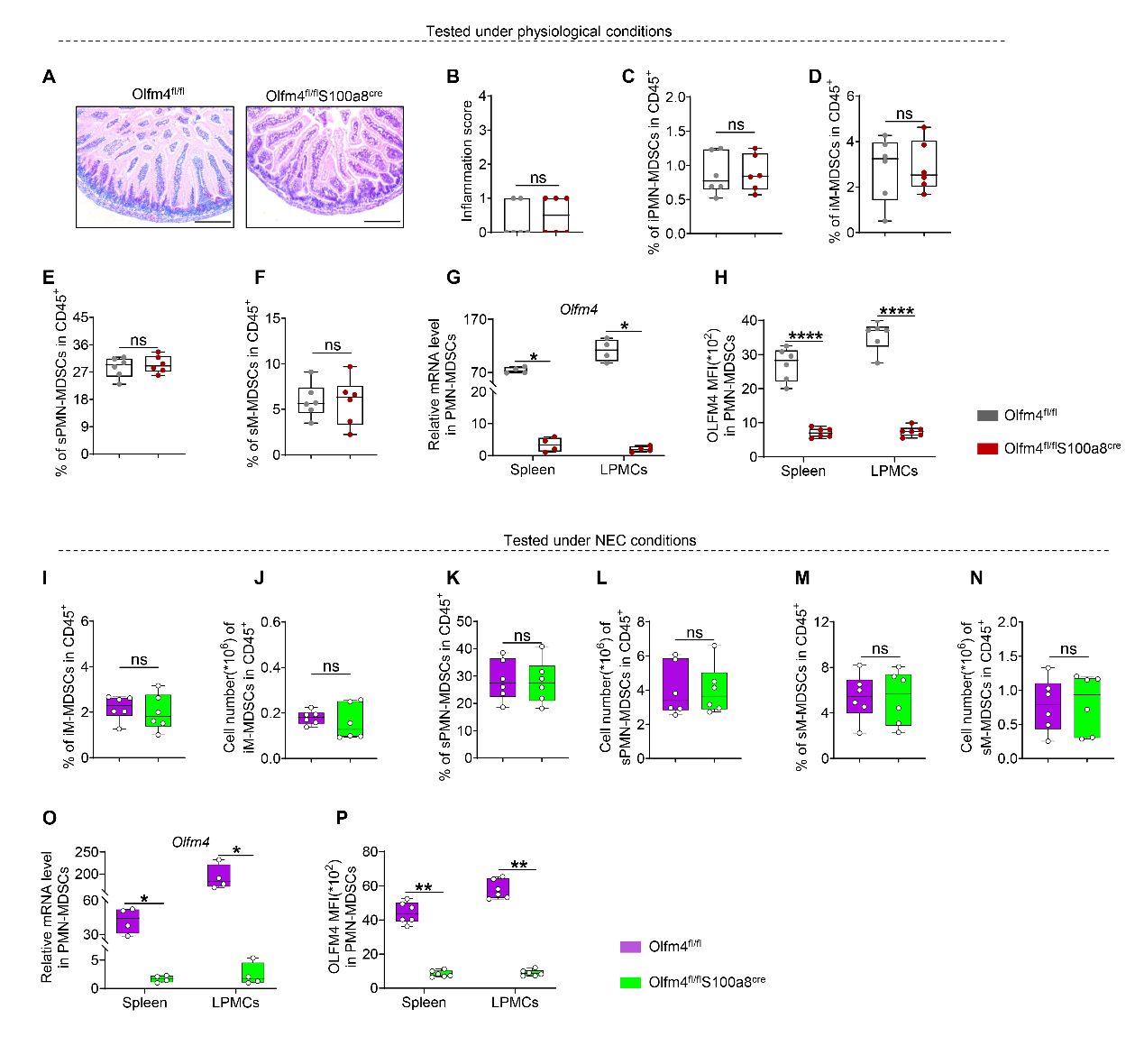


**Fig. S6 Neutrophil *Olfm4* deficiency does not alter MDSC subsets under physiological conditions and has no effect on iM-MDSC or splenic MDSC levels under NEC conditions. A**, **B** Representative H&E staining of the intestine (A) and statistical analysis of inflammation scores under physiological conditions (B, *n* = 6, scale bar: 50 μm). **C**, **D** Percentages of iPMN-MDSCs (C) and iM-MDSCs (D) among CD45^+^ cells under physiological conditions (*n* = 6). **E**, **F** Percentages of sPMN-MDSCs (E) and sM-MDSCs (F) among CD45^+^ cells under physiological conditions (*n* = 6). **G**, **H** mRNA expression levels (G, *n* = 4 biological replicates) and mean fluorescence intensity (MFI) (H, *n* = 6) of OLFM4 in PMN-MDSCs under physiological conditions. **I**, **J** Percentage (I) and cell number (J) of iM-MDSCs among CD45^+^ cells under NEC conditions (*n* = 6). **K**, **L** Percentage (K) and cell number (L) of sPMN-MDSCs among CD45^+^ cells under NEC conditions (*n* = 6). **M**, **N** Percentage (M) and cell number (N) of sM-MDSCs among CD45^+^ cells under NEC conditions (*n* = 6). **O**, **P** mRNA expression levels (O, *n* = 4 biological replicates) and MFI (P, *n* = 6) of OLFM4 in PMN-MDSCs under NEC conditions. Data are presented as mean ± SEM. Each symbol represents one pup in a litter. ns, not significant; **p* <0.05, ***p* < 0.01, ****p* < 0.001, *****p* < 0.0001. Statistical significance was determined using a Student’s *t-*test (**B–F** and **H–N**) or Mann–Whitney test (**G**, **O**, and **P**).

**
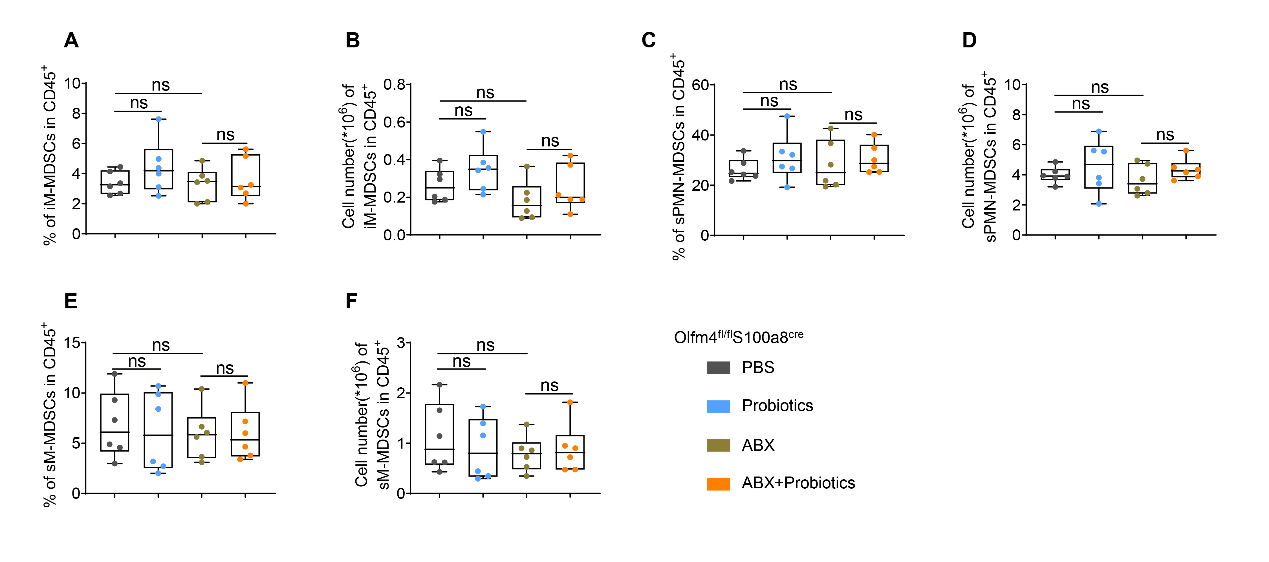
**

**Fig. S7 Antibiotic or probiotic intervention does not alter iM-MDSC or splenic MDSC levels in *Olfm4*-deficient neonates. A**, **B** Percentage (A) and cell number (B) of iM-MDSCs among CD45^+^ cells in Olfm4^fl/fl^S100a8^cre^ pups following different treatments (*n* = 6). **C**, **D** Percentage (C) and cell number (D) of sPMN-MDSCs among CD45^+^ cells (*n* = 6). **E**, **F** Percentage (E) and cell number (F) of sM-MDSCs among CD45^+^ cells (*n* = 6). Data are presented as mean ± SEM. Each symbol represents one pup in a litter. ns, not significant; **p* <0.05, ***p* < 0.01, ****p* < 0.001, *****p* < 0.0001. Statistical significance was determined using one-way ANOVA (**A–F**). Post-hoc analyses were performed using Tukey’s test (**A–F**).

**
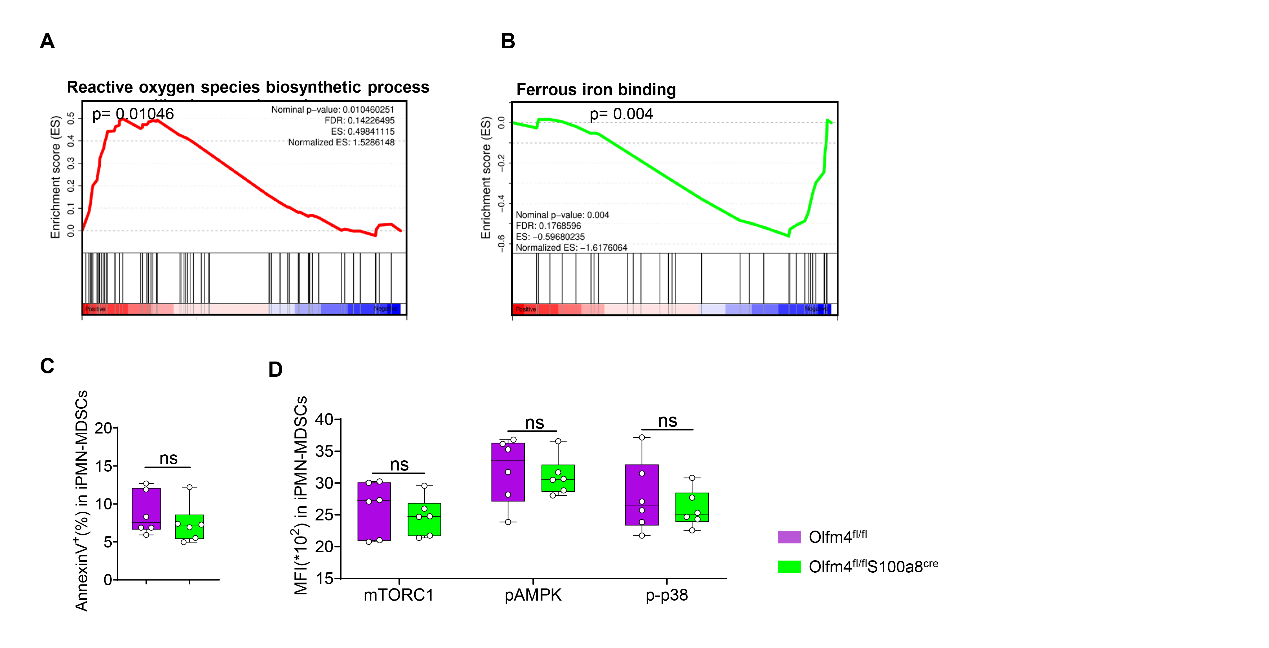
**

**Fig. S8** ***Olfm4* deficiency is associated with ferroptosis rather than apoptosis in iPMN-MDSCs.** **A**, **B** Gene set enrichment analysis (GSEA) showing upregulation of reactive oxygen species (ROS) biosynthetic process (A) and downregulation of ferrous iron binding (B). **C** Statistical analysis of Annexin V^+^ cells in iPMN-MDSCs (*n* = 6). **D** MFI of mTORC1, phosphorylated AMPK (pAMPK), and phosphorylated p38 (p-p38) in iPMN-MDSCs (*n* = 6). Data are presented as mean ± SEM. Each symbol represents one pup in a litter. ns, not significant; **p* <0.05, ***p* < 0.01, ****p* < 0.001, *****p* < 0.0001. Statistical significance was determined using a Student’s *t-*test (**C** and **D**).


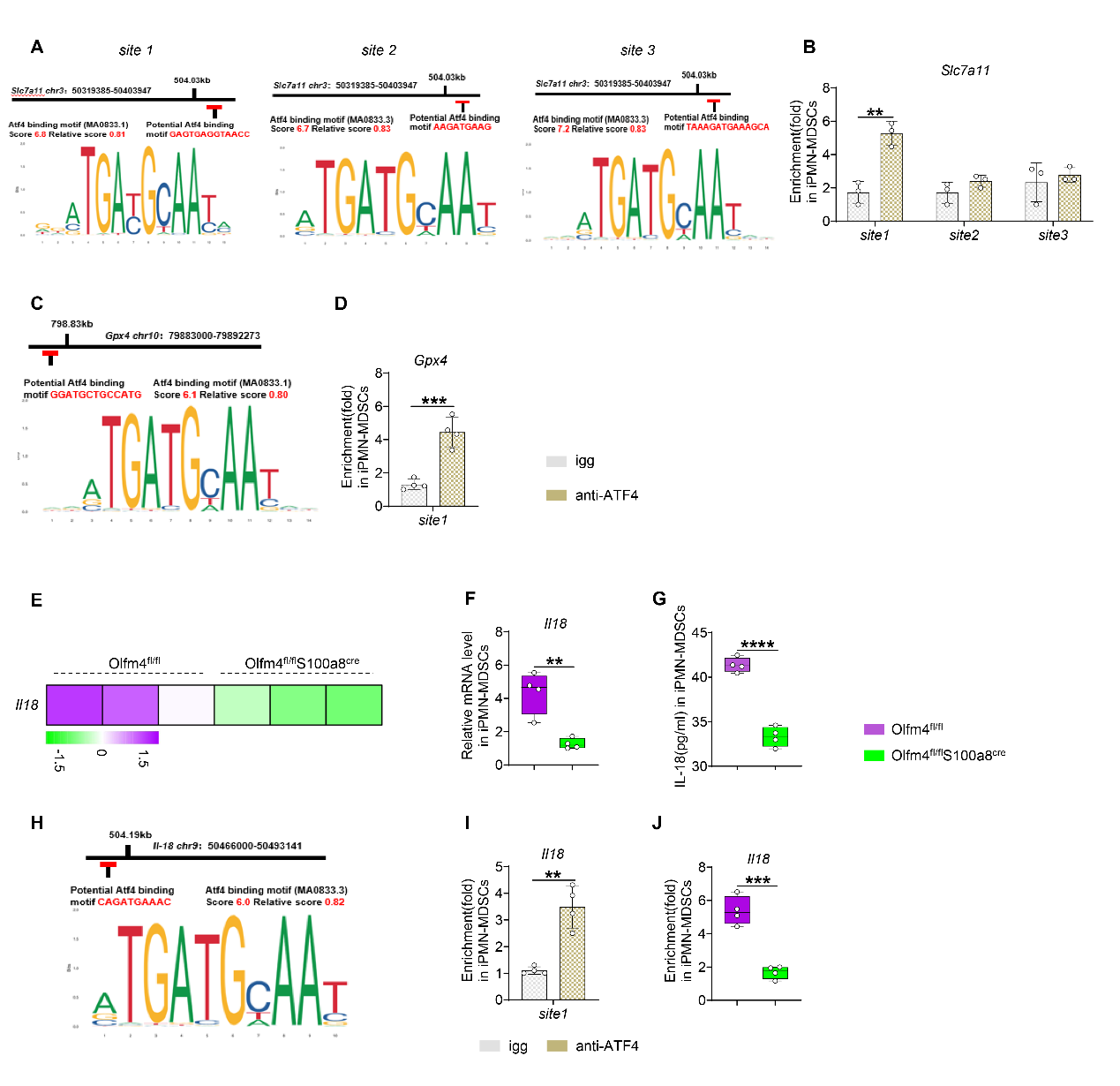


**Fig. S9 ATF4 directly binds to the promoters of Slc7a11, Gpx4, and Il18. *Olfm4* deficiency impairs this binding at the Il18 promoter and reduces IL-18 expression levels in iPMN-MDSCs. A**, **B** The predicted ATF4 binding site (A) and Chromatin immunoprecipitation (ChIP) assays for the Slc7a11 promoter in iPMN-MDSCs (B, *n* = 3). **C**, **D** The predicted ATF4 binding site (C) and ChIP assays for the Gpx4 promoter in iPMN-MDSCs (D, *n* = 4). **E** Heatmap of *Il18* expression in iPMN-MDSCs from Olfm4^fl/fl^ and Olfm4^fl/fl^S100a8^cre^ neonates (*n* = 3). **F**, **G** mRNA (F, *n* = 4 biological replicates) and secretion (G, *n* = 4) levels of IL-18 in iPMN-MDSCs. **H**, **I** The predicted ATF4 binding site (H) and ChIP assays for the Il18 promoter in iPMN-MDSCs (I, *n* = 4). **J** ATF4 binding to the Il18 promoter was impaired in iPMN-MDSCs from Olfm4^fl/fl^S100a8^cre^ neonates (*n* = 4). Data are presented as mean ± SEM. Each symbol represents one pup in a litter. ns, not significant; **p* <0.05, ***p* < 0.01, ****p* < 0.001, *****p* < 0.0001. Statistical significance was determined using a Student’s *t-*test (**B**, **D**, **F**, **G**, **I**, and **J**).


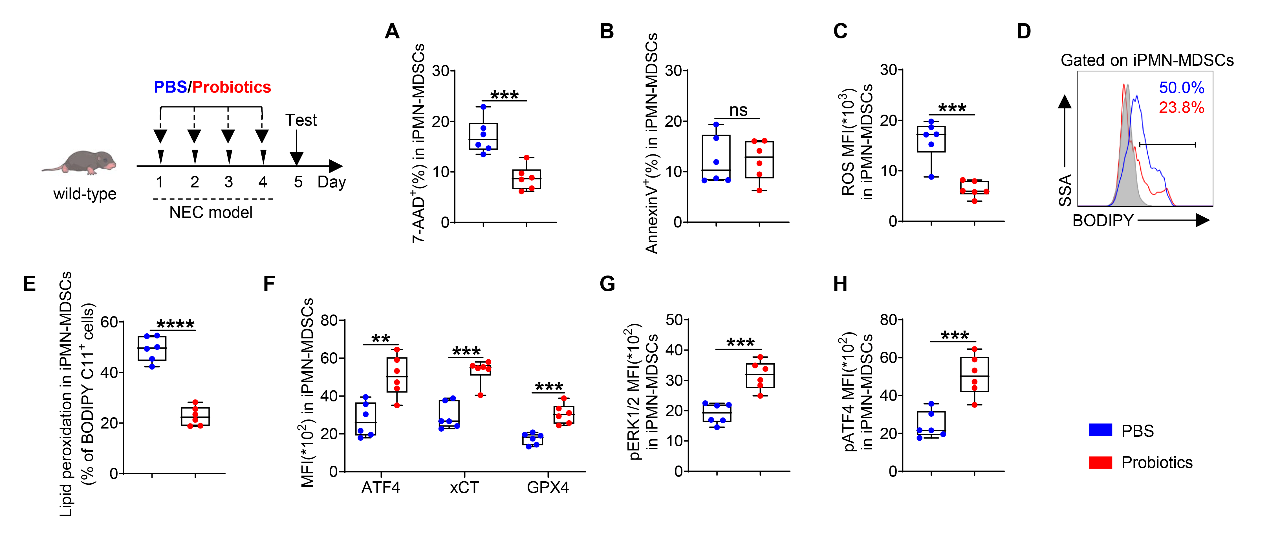


**Fig. S10 Probiotic treatment inhibits ferroptosis in iPMN-MDSCs from wild-type neonates under NEC conditions. A–E** Statistical analyses of 7-AAD^+^ cells, Annexin V^+^ cells, ROS levels, and percentage of BODIPY C11^+^ cells (lipid peroxidation) in iPMN-MDSCs from neonates treated with PBS or probiotic cocktail (*n* = 6). **F** MFI of ATF4, xCT, and GPX4 in iPMN-MDSCs (*n* = 6). **G**, **H** MFI of phosphorylated ERK1/2 (pERK1/2) and phosphorylated ATF4 (pATF4) in iPMN-MDSCs (*n* = 6). Data are presented as mean ± SEM. Each symbol represents one pup in a litter. ns, not significant; **p* <0.05, ***p* < 0.01, ****p* < 0.001, *****p* < 0.0001. Statistical significance was determined using a Student’s *t-*test (**A–C** and **E–H**).


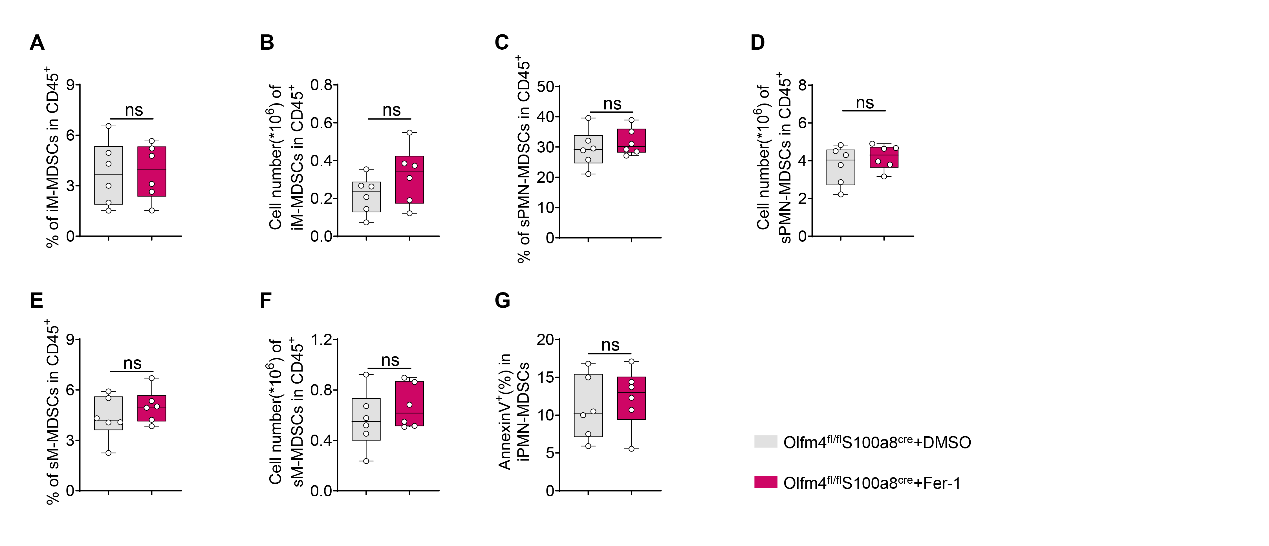


**Fig. S11 Ferrostatin-1 does not affect the levels of iM-MDSCs and splenic MDSCs, or the apoptosis of iPMN-MDSCs.** **A**, **B** Percentage (A) and cell number (B) of iM-MDSCs among CD45^+^ cells from Olfm4^fl/fl^S100a8^cre^ neonates treated with a vehicle control or ferrostatin-1 (*n* = 6). **C**, **D** Percentage (C) and cell number (D) of sPMN-MDSCs among CD45^+^ cells (*n* = 6). **E**, **F** Percentage (E) and cell number (F) of sM-MDSCs among CD45^+^ cells (*n* = 6). **G** Statistical analysis of Annexin V^+^ cells in iPMN-MDSCs (*n* = 6). Data are presented as mean ± SEM. Each symbol represents one pup in a litter. ns, not significant; **p* <0.05, ***p* < 0.01, ****p* < 0.001, *****p* < 0.0001. Statistical significance was determined using a Student’s *t-*test (**A–G**).

**
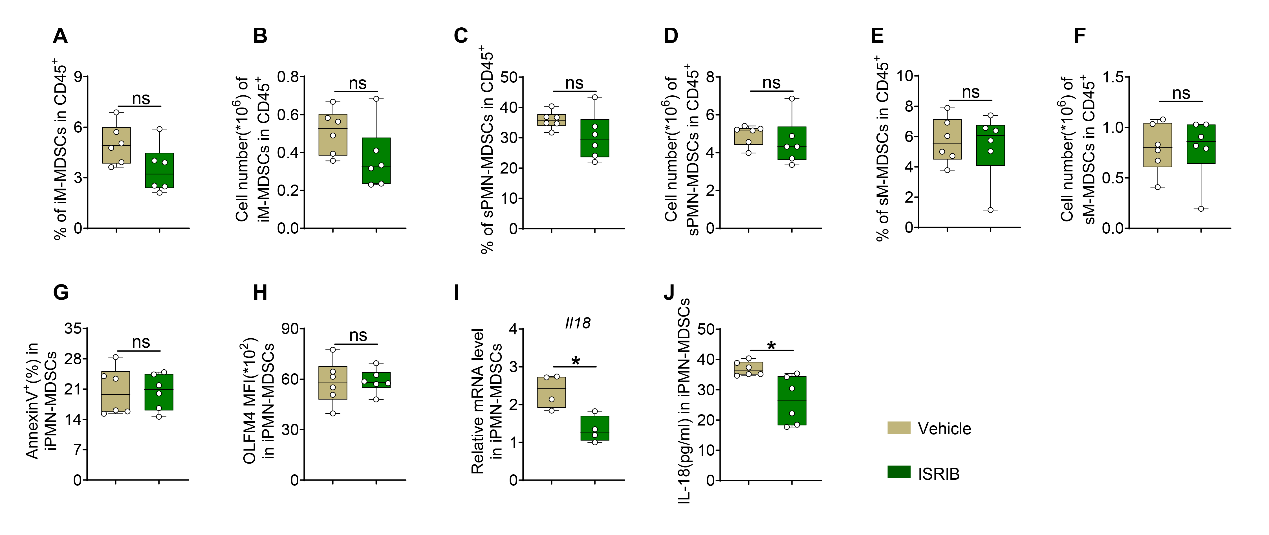
**

**Fig. S12 ISRIB reduces IL-18 levels but does not affect iM-MDSC levels, splenic MDSC levels, iPMN-MDSC apoptosis, or OLFM4 expression. A**, **B** Percentage (A) and cell number (B) of iM-MDSCs among CD45^+^ cells from wild-type neonates treated with a vehicle control or ISRIB (*n* = 6). **C**, **D** Percentage (C) and cell number (D) of sPMN-MDSCs among CD45^+^ cells (*n* = 6). **E**, **F** Percentage (E) and cell number (F) of sM-MDSCs among CD45^+^ cells (*n* = 6). **G** Statistical analysis of Annexin V^+^ cells in iPMN-MDSCs (*n* = 6). **H** MFI of OLFM4 in iPMN-MDSCs (*n* = 6). **I**, **J** mRNA (I, *n* = 4 biological replicates) and secretion (J, *n* = 6) levels of IL-18 in iPMN-MDSCs. Data are presented as mean ± SEM. Each symbol represents one pup in a litter. ns, not significant; **p* <0.05, ***p* < 0.01, ****p* < 0.001, *****p* < 0.0001. Statistical significance was determined using a Student’s *t-*test (**A–J**).

**
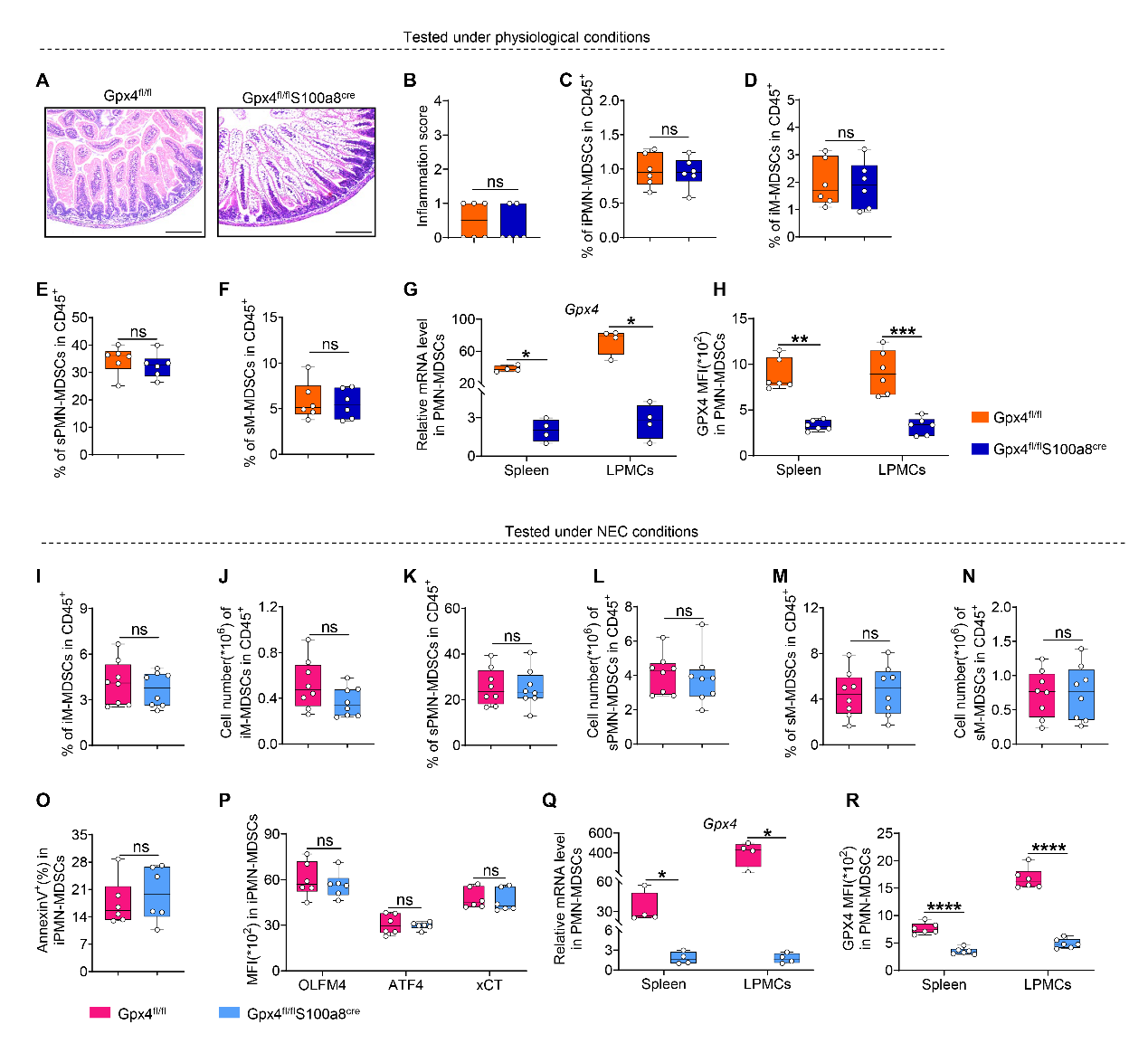
**

**Fig. S13 Neutrophil *Gpx4* deficiency does not alter MDSC subsets under physiological conditions, nor does it affect iM‑MDSC levels, splenic MDSC levels, or the anti‑ferroptosis axis under NEC. A**, **B** Representative H&E staining of the intestine (A) and statistical analysis of inflammation scores (B, *n* = 6, scale bar: 50 μm). **C**, **D** Percentages of iPMN-MDSCs (C) and iM-MDSCs (D) among CD45^+^ cells under physiological conditions (*n* = 6). **E**, **F** Percentages of sPMN-MDSCs (E) and sM-MDSCs (F) among CD45^+^ cells under physiological conditions (*n* = 6). **G**, **H** mRNA expression levels (G, *n* = 4 biological replicates) and MFI (H, *n* = 6) of GPX4 in PMN-MDSCs under physiological conditions. **I**, **J** Percentage (I) and cell number (J) of iM-MDSCs among CD45^+^ cells under NEC conditions (*n* = 8). **K**, **L** Percentage (K) and cell number (L) of sPMN-MDSCs among CD45^+^ cells under NEC conditions (*n* = 8). **M**, **N** Percentage (M) and cell number (N) of sM-MDSCs among CD45^+^ cells under NEC conditions (*n* = 8). **O** Statistical analysis of Annexin V^+^ cells in iPMN-MDSCs (*n* = 6). **P** MFI of OLFM4, ATF4, and xCT in iPMN-MDSCs (*n* = 6). **Q**, **R** mRNA expression levels (Q, *n* = 4 biological replicates) and MFI (R, *n* = 6) of GPX4 in PMN-MDSCs. Data are presented as mean ± SEM. Each symbol represents one pup in a litter. ns, not significant; **p* <0.05, ***p* < 0.01, ****p* < 0.001, *****p* < 0.0001. Statistical significance was determined using a Student’s *t-*test (**B–F**, **H–P**, and **R**) or Mann–Whitney test (**G**, **H**, and **Q**).

**
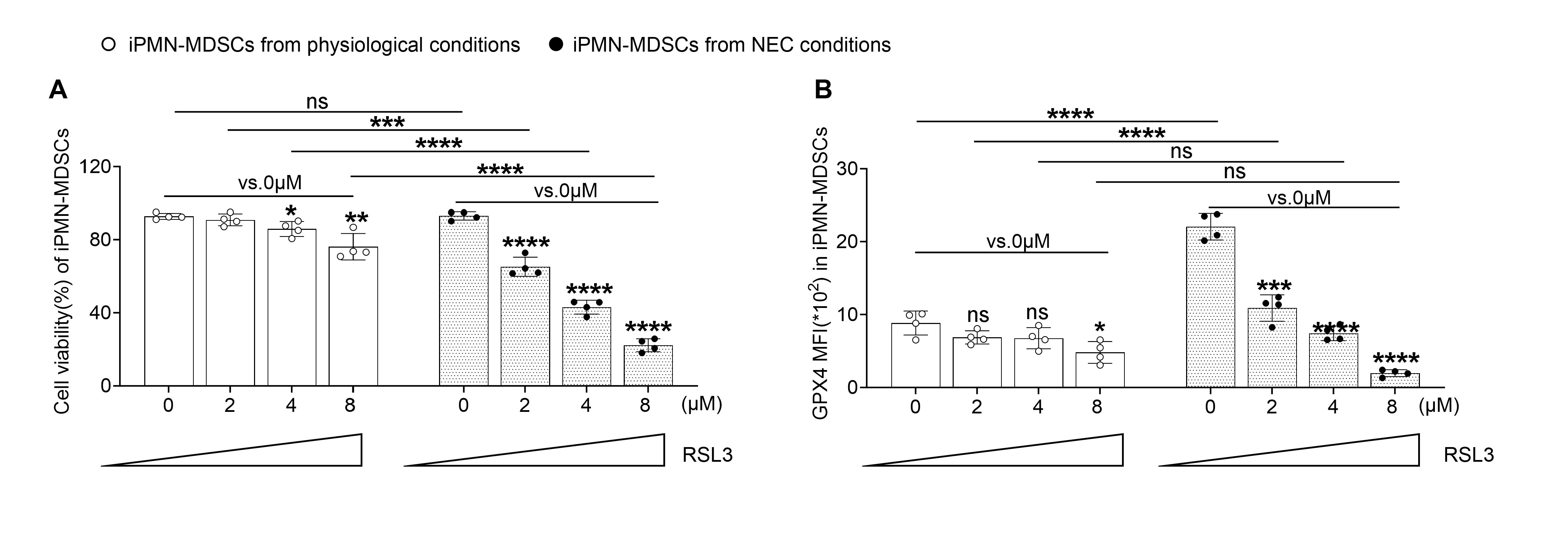
**

**Fig. S14 *In vitro* RSL3 treatment significantly reduces cell viability and GPX4 expression in iPMN-MDSCs from NEC conditions relative to physiological conditions. A**, **B** Cell viability (A) and GPX4 expression (B) in iPMN-MDSCs isolated from physiological or NEC conditions following *in vitro* treatment with RSL3 (*n* = 4). Data are presented as mean ± SEM. Each symbol represents one pup in a litter. ns, not significant; **p* <0.05, ***p* < 0.01, ****p* < 0.001, *****p* < 0.0001. Statistical significance was determined using a Student’s *t*-test (**A** and **B**) or one-way ANOVA (**A** and **B**). Post-hoc analyses were performed using Dunnett’s test (**A** and **B**).


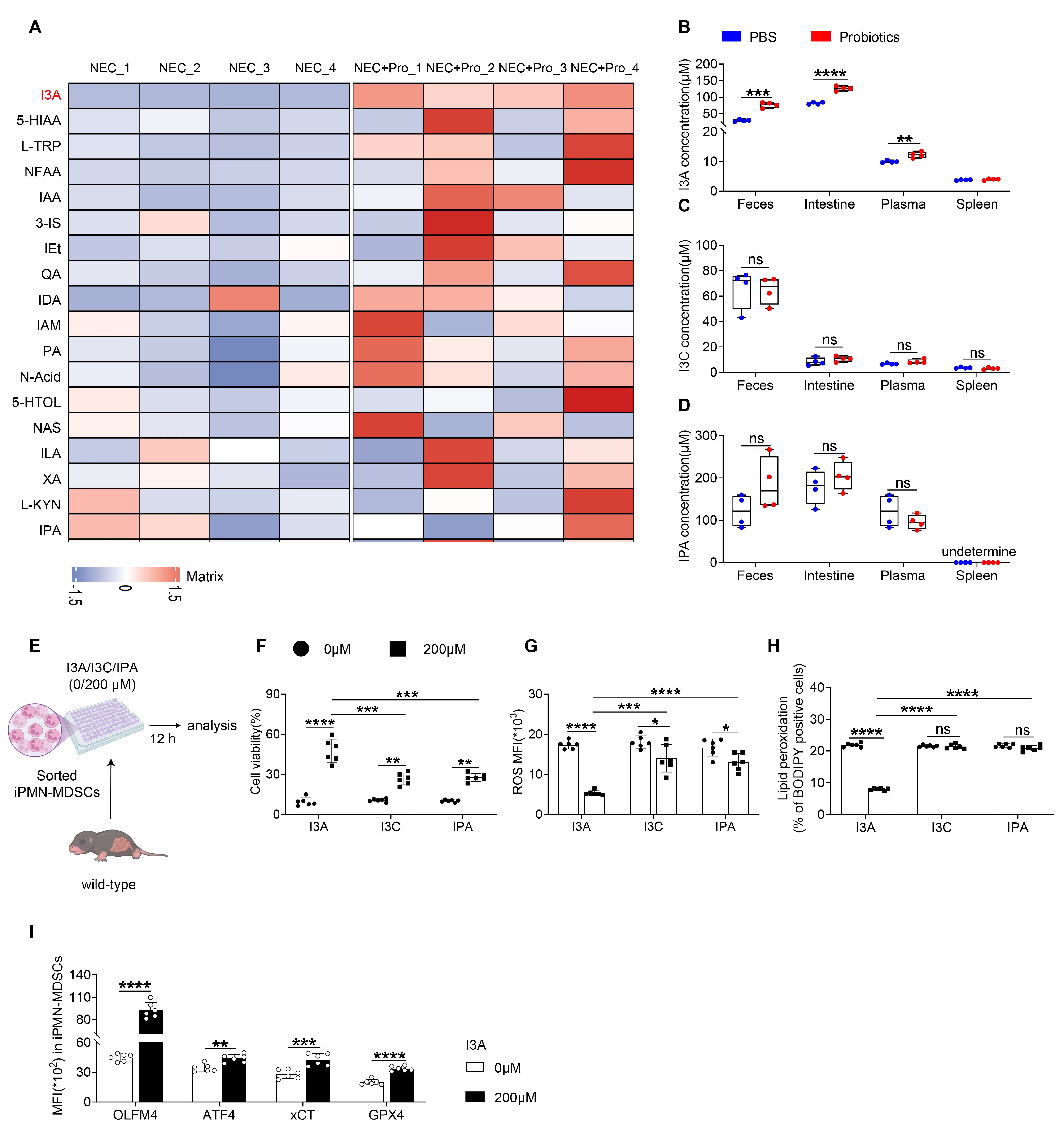


**Fig. S15 Probiotic treatment elevates *in vivo* I3A levels, and *in vitro* I3A treatment inhibits ferroptosis in iPMN-MDSCs. A** Liquid chromatography-mass spectrometry (LC-MS/MS) analysis of the fecal samples from neonates treated with PBS or probiotic cocktail (*n* = 4). **B–D** The concentrations of indole-3-aldehyde (I3A), indole-3-carbionol (I3C), and indole-3-propionic acid (IPA) in the feces, intestine, plasma, and spleen (*n* = 4). **E** Sorted iPMN-MDSCs were cultured with 0 or 200 μM of I3A, I3C, and IPA *in vitro*. **F–H** Statistical analysis of 7-AAD staining (F), ROS levels (G), and lipid ROS (H) of iPMN-MDSCs (*n* = 6). **I** MFI of OLFM4, ATF4, xCT, and GPX4 in iPMN-MDSCs treated with 0 or 200 μM I3A *in vitro* (*n* = 6). Data are presented as mean ± SEM. Each symbol represents one pup in a litter. ns, not significant; **p* <0.05, ***p* < 0.01, ****p* < 0.001, *****p* < 0.0001. Statistical significance was determined using a Student’s *t-*test (**B–D** and **F–I**) or Mann–Whitney test (**F–H**).


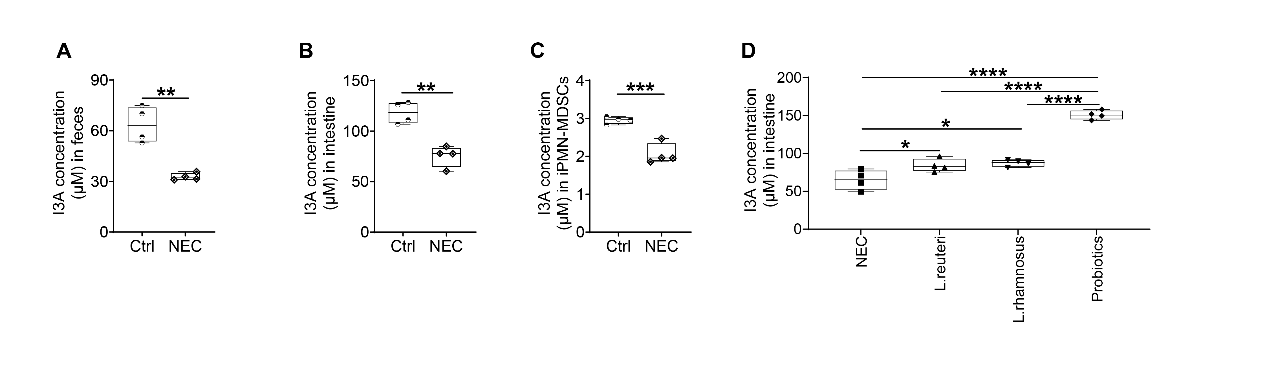


**Fig. S16 I3A levels are reduced under NEC conditions and restored by combined probiotic treatment. A–C** I3A concentrations in the feces (A), intestine (B), and iPMN-MDSCs (C) of controls and NEC model mice (*n* = 4). **D** Comparison of intestinal I3A levels in NEC-induced pups treated with PBS, *L. reuteri*, *L. rhamnosus*, or the combination of both strains (*n* = 4). Data are presented as mean ± SEM. Each symbol represents one pup in a litter. ns, not significant; **p* <0.05, ***p* < 0.01, ****p* < 0.001, *****p* < 0.0001. Statistical significance was determined using a Student’s *t-*test (**A–C**) or one-way ANOVA (**D**). Post-hoc analyses were performed using Tukey’s test (**D**).


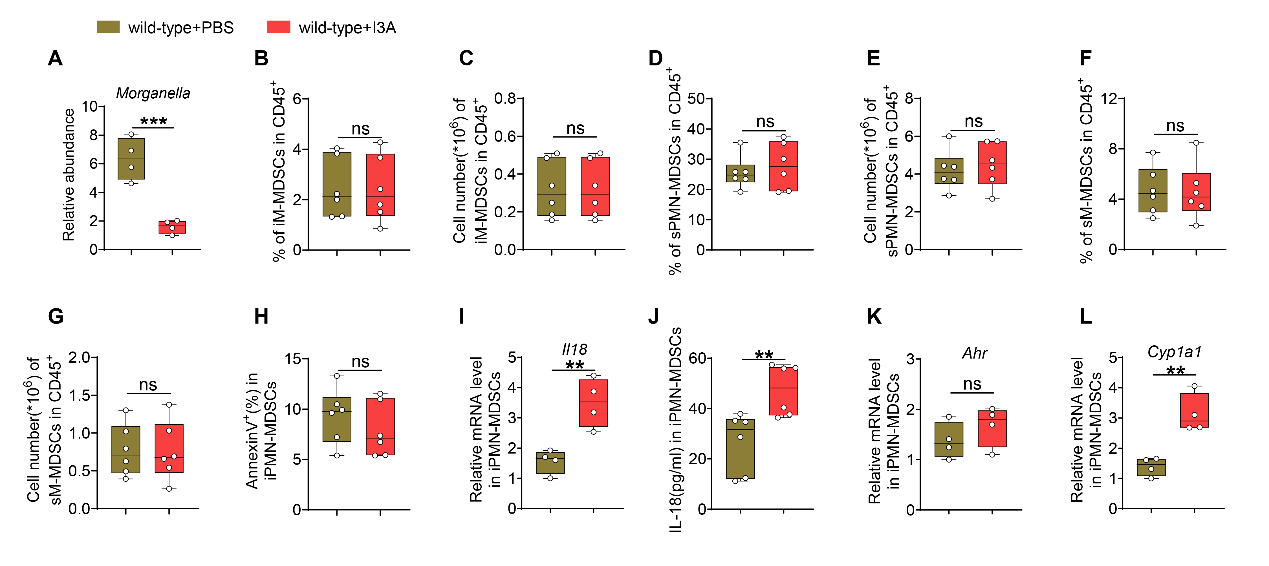


**Fig. S17 I3A treatment upregulates *Il18* and *Cyp1a1* in iPMN-MDSCs and has no effect on iM-MDSC or splenic MDSC levels. A** The relative abundance of *Morganella* in fecal samples (*n* = 4). **B**, **C** Percentage (B) and cell number (C) of iM-MDSCs among CD45^+^ cells (*n* = 6). **D**, **E** Percentage (D) and cell number (E) of sPMN-MDSCs among CD45^+^ cells (*n* = 6). **F**, **G** Percentage (F) and cell number (G) of sM-MDSCs among CD45^+^ cells (*n* = 6). **H** Statistical analysis of Annexin V^+^ cells in iPMN-MDSCs (*n* = 6). **I**, **J** mRNA (I, *n* = 4 biological replicates) and secretion (J, *n* = 6) levels of IL-18 in iPMN-MDSCs. **K**, **L** mRNA expression levels of *Ahr* (K) and *Cyp1a1* (L) in iPMN-MDSCs (*n* = 4 biological replicates). Data are presented as mean ± SEM. Each symbol represents one pup in a litter. ns, not significant; **p* <0.05, ***p* < 0.01, ****p* < 0.001, *****p* < 0.0001. Statistical significance was determined using a Student’s *t-*test (**A–L**).

**
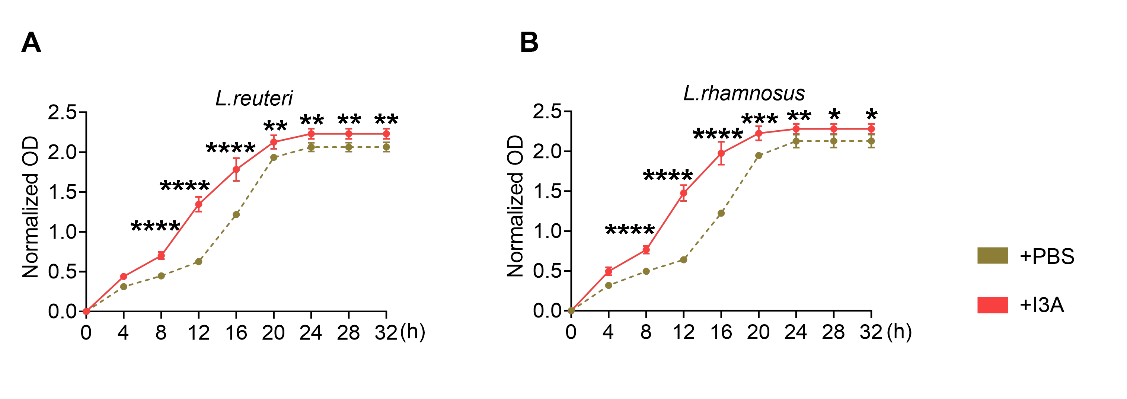
**

**Fig. S18 I3A promotes the proliferation of *L. reuteri* and *L. rhamnosus.* A**, **B** The proliferation of *L. reuteri* (A) and *L. rhamnosus* (B) cultured with I3A or PBS. The growth curves were monitored by measuring the optical density at 600 nm (OD_600_). Data are from five independent replicates.


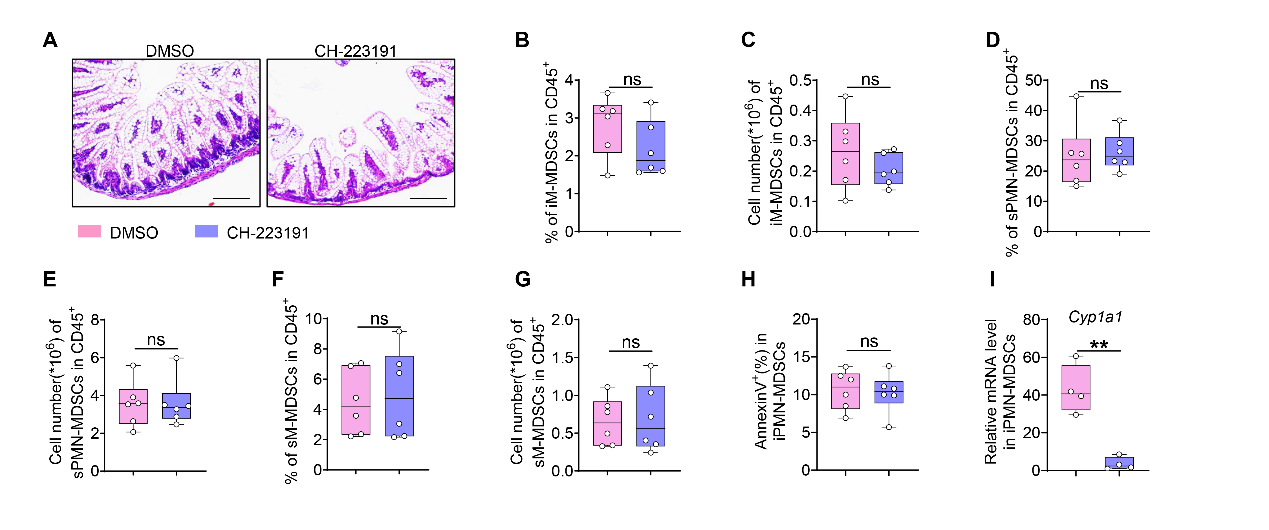


**Fig. S19 AHR inhibition has no effect on iM-MDSC levels, splenic MDSC levels, or iPMN-MDSC apoptosis. A** Representative H&E staining of the intestine (scale bar: 50 μm). **B**, **C** Percentage (B) and cell number (C) of iM-MDSCs among CD45^+^ cells following a vehicle control or CH-223191 treatment (*n* = 6). **D**, **E** Percentage (D) and cell number (E) of sPMN-MDSCs among CD45^+^ cells (*n* = 6). **F**, **G** Percentage (F) and cell number (G) of sM-MDSCs among CD45^+^ cells (*n* = 6). **H** Statistical analysis of Annexin V^+^ cells in iPMN-MDSCs (*n* = 6). **I**. mRNA expression levels of *Cyp1a1* in iPMN-MDSCs (*n* = 4 biological replicates). Data are presented as mean ± SEM. Each symbol represents one pup in a litter. ns, not significant; **p* <0.05, ***p* < 0.01, ****p* < 0.001, *****p* < 0.0001. Statistical significance was determined using a Student’s *t-*test (**B–I**).

***
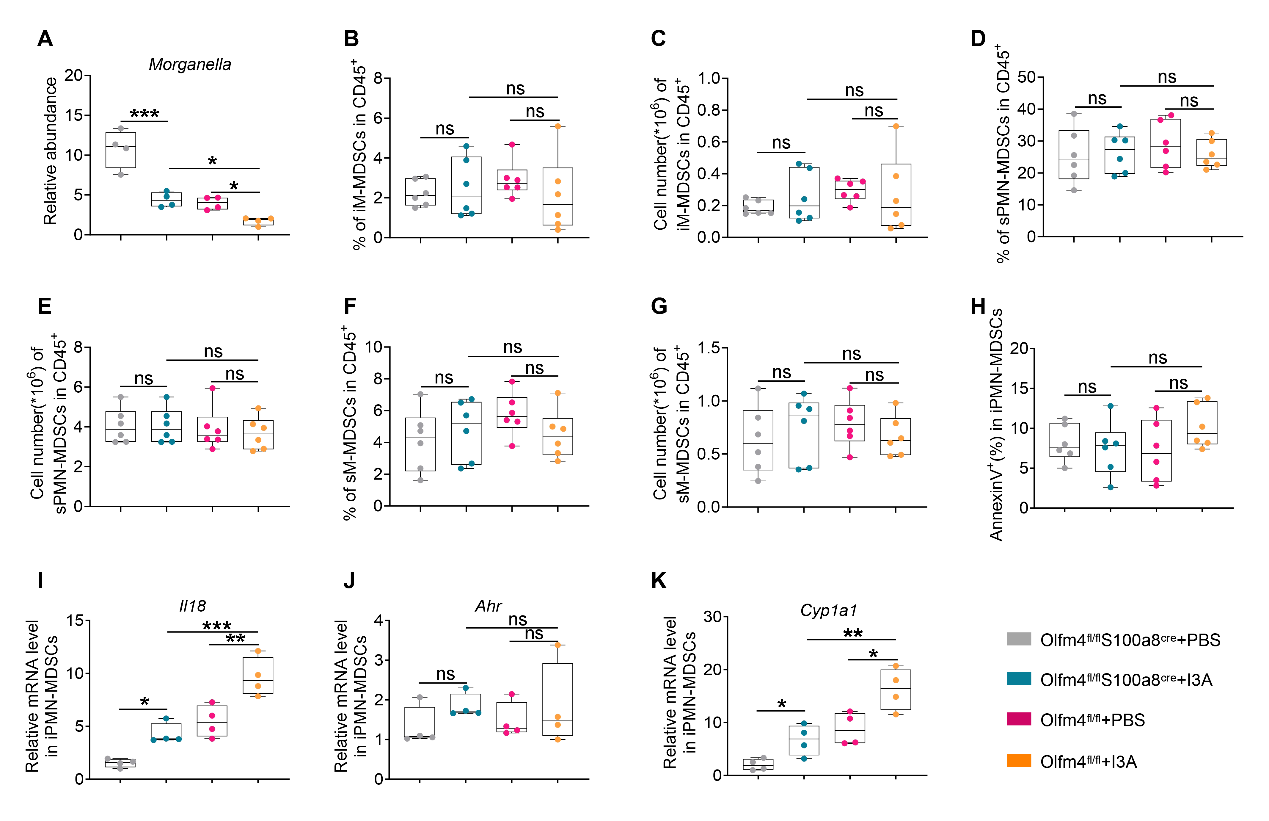
***

**Fig. S2****0 The presence of OLFM4 significantly enhances *Il18* and *Cyp1a1* expression in iPMN-MDSCs following I3A treatment but does not affect iM-MDSC or splenic MDSC levels.** **A** The relative abundance of *Morganella* in fecal samples (*n* = 4). **B**, **C** Percentage (B) and cell number (C) of iM-MDSCs among CD45^+^ cells (*n* = 6). **D**, **E** Percentage (D) and cell number (E) of sPMN-MDSCs among CD45^+^ cells (*n* = 6). **F**, **G** Percentage (F) and cell number (G) of sM-MDSCs among CD45^+^ cells (*n* = 6). **H** Statistical analysis of Annexin V^+^ cells in iPMN-MDSCs (*n* = 6). **I** mRNA expression levels of *Il18* in iPMN-MDSCs (*n* = 4 biological replicates). **J**, **K** mRNA expression levels of *Ahr* and *Cyp1a1* in iPMN-MDSCs (*n* = 4 biological replicates). Data are presented as mean ± SEM. Each symbol represents one pup in a litter. ns, not significant; **p* <0.05, ***p* < 0.01, ****p* < 0.001, *****p* < 0.0001. Statistical significance was determined using one-way ANOVA (**A–K**). Post-hoc analyses were performed using Turkey’s test (**B–K**) or Dunnett’s T3 test (**A**).

**
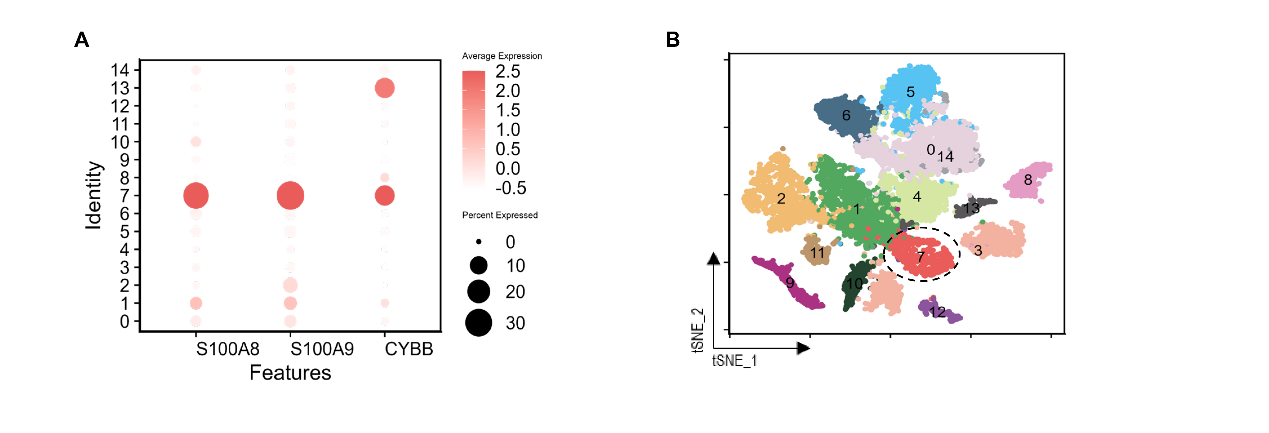
**

**Fig. S21 Single-cell RNA sequencing identifies a distinct neutrophil cluster in the infant intestine.** **A** Expression of canonical neutrophil marker genes among intestinal CD45^+^ immune cells. Cluster_7 was identified as neutrophils. **B** t-distributed stochastic neighbor embedding (t-SNE) visualization of the cells comprising the neutrophil cluster (the dotted line, *n* = 5).

**
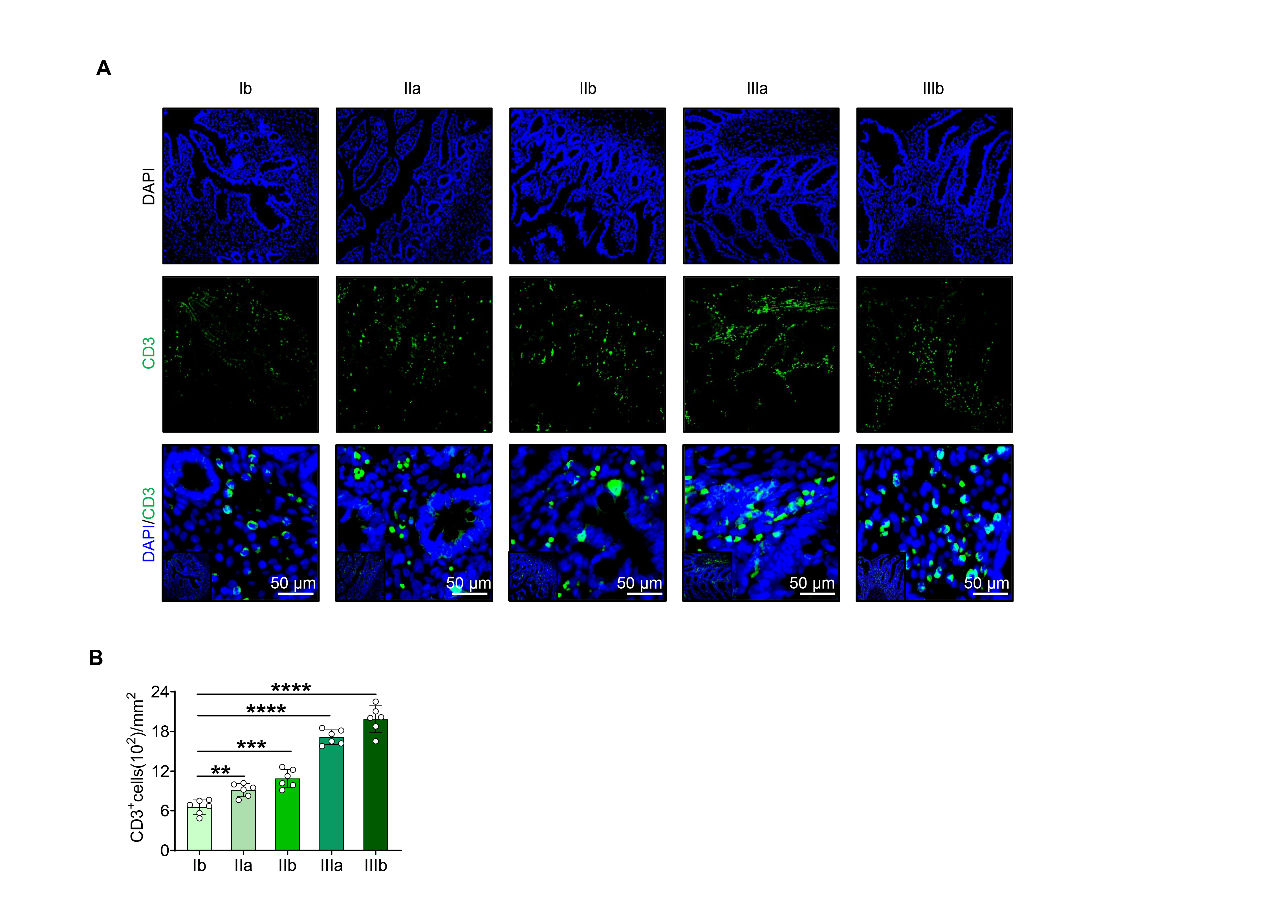
**

**Fig. S22 The levels of CD3^+^ T cells in the intestine during NEC progression. A** Representative immunofluorescence staining of CD3^+^ T cells in the intestine (scale bar: 50 μm). **B** Statistical analyses of CD3^+^ T cells/mm^2^ during NEC progression (*n* = 6). Data are represented as mean ± SEM. Each symbol represents an individual infant. ns, not significant; **p* <0.05, ***p* < 0.01, ****p* < 0.001, *****p* < 0.0001. Statistical significance was determined using one-way ANOVA (**B**). Post-hoc analyses were performed using Dunnett’s test (**B**).

**
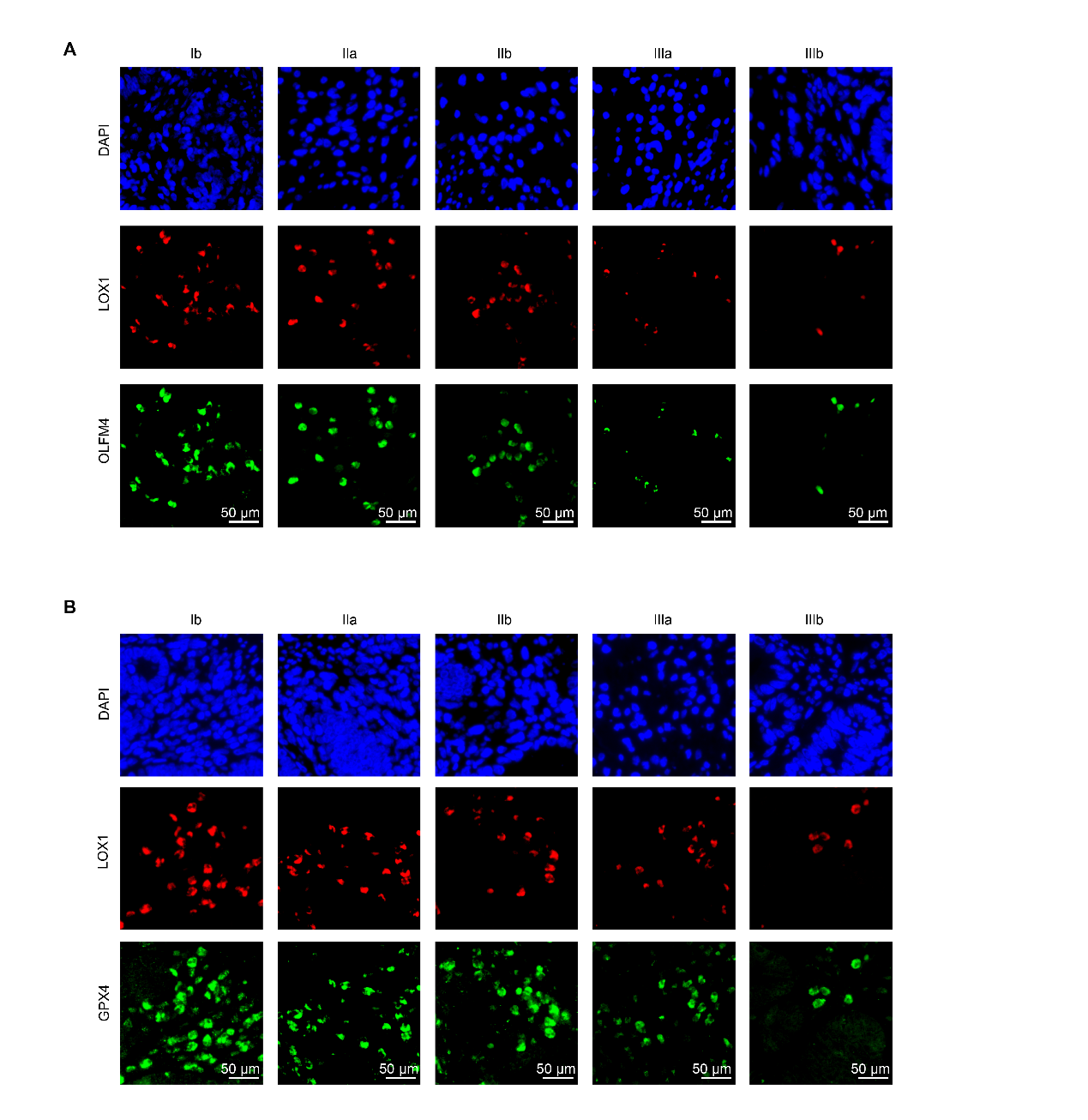
**

**Fig. S23** **Reduced OLFM4 and GPX4 expression with NEC progression.** **A** Representative immunofluorescence staining of OLFM4 expression in NEC development. **B** Representative immunofluorescence staining of GPX4 expression during NEC development (scale bar: 50 μm).

**
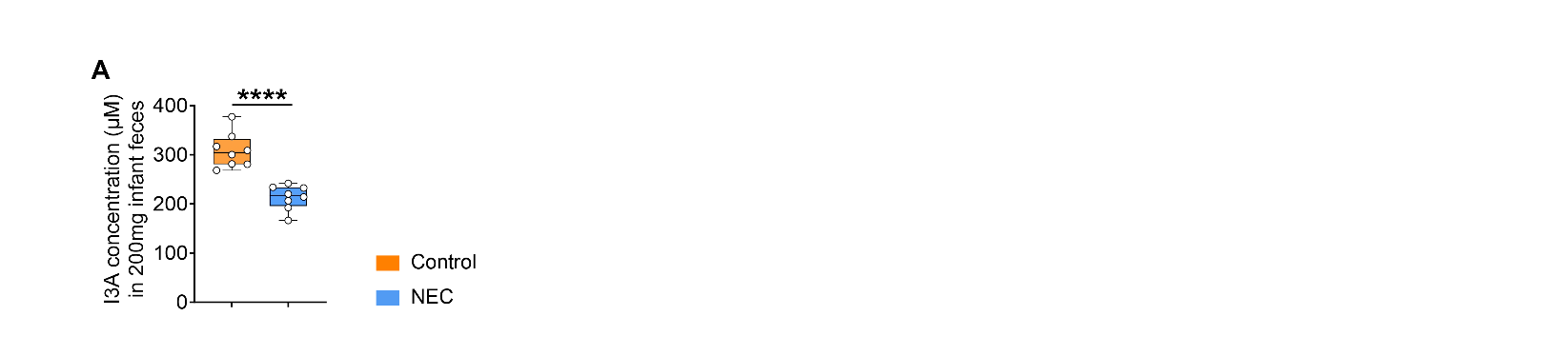
**

**Fig. S24 Fecal I3A levels are reduced in infants with NEC. A** I3A concentrations in fecal samples from the controls and infants with NEC (*n* = 8). Data are represented as mean ± SEM. Each symbol represents an individual infant. ns, not significant; **p* <0.05, ***p* < 0.01, ****p* < 0.001, *****p* < 0.0001. Statistical significance was determined using a Student’s *t-*test (**A**).


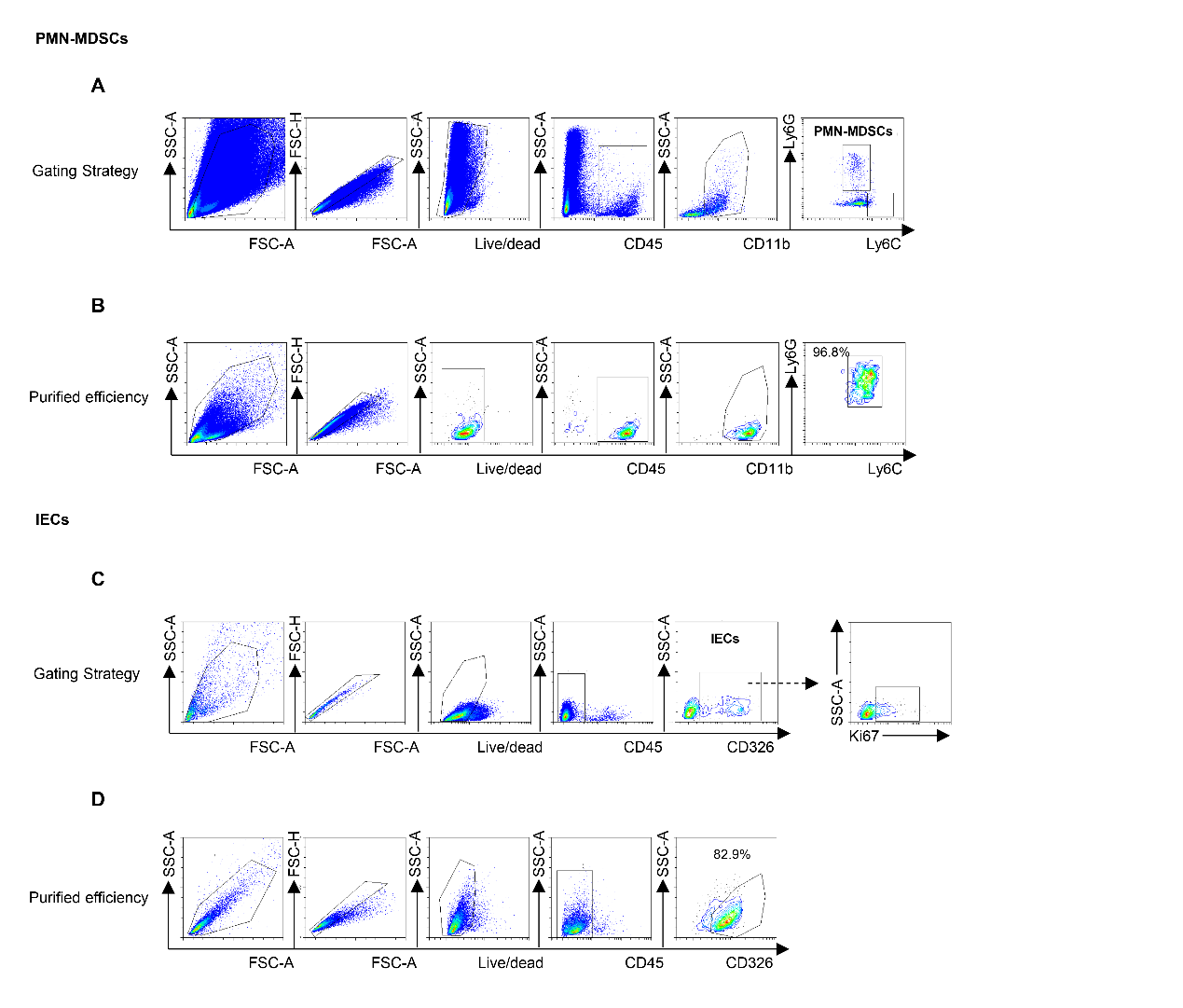


**Fig. S25** The gating strategy and purified efficiency of MDSC subsets and IECs. **A**, **B** The gating strategy (A) and purified efficiency (B) of iPMN-MDSCs and iM-MDSCs. **C**, **D** The gating strategy (C) and purified efficiency (D) of IECs.

**Supplementary Data**

**The full length uncropped original western blots**

**Fig. 1K**


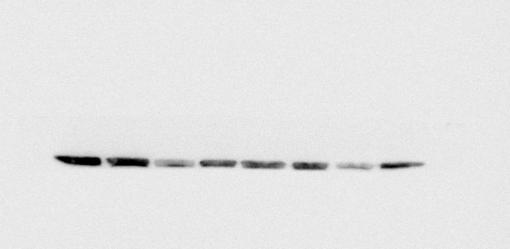


Zo-1

250 kDa

130 kDa


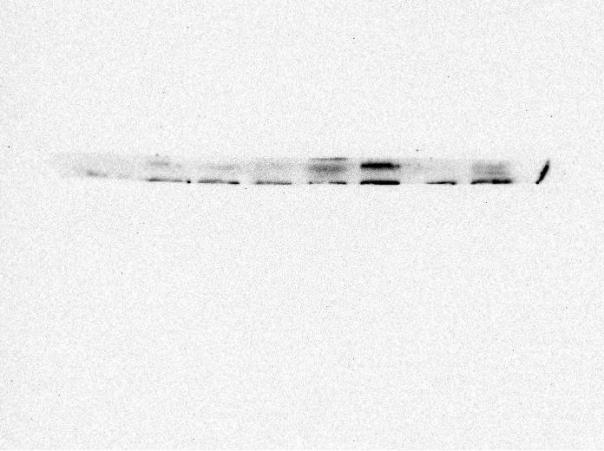


Occludin

75 kDa

55 kDa


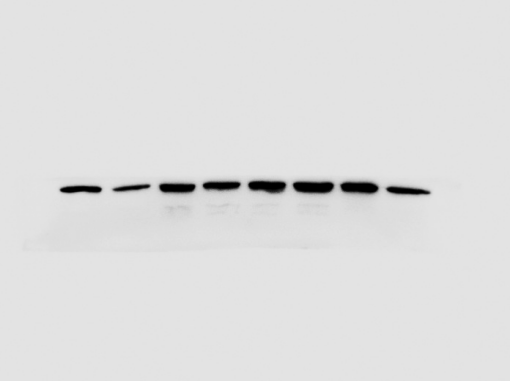


55 kDa

43 kDa

β-actin

**Fig. 2H**


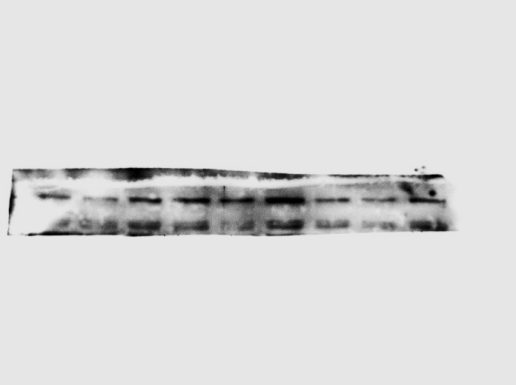


Occludin

75 kDa

55 kDa


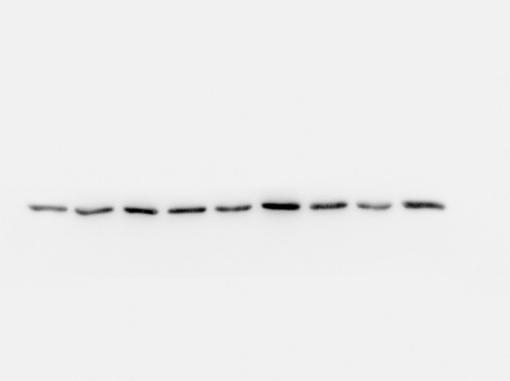


Zo-1

250 kDa

130 kDa


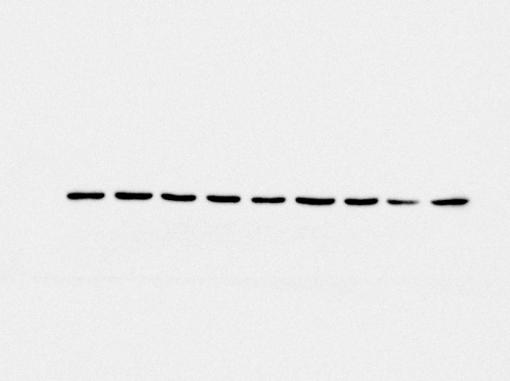


β-actin

55 kDa

43 kDa

**Fig. 3H**


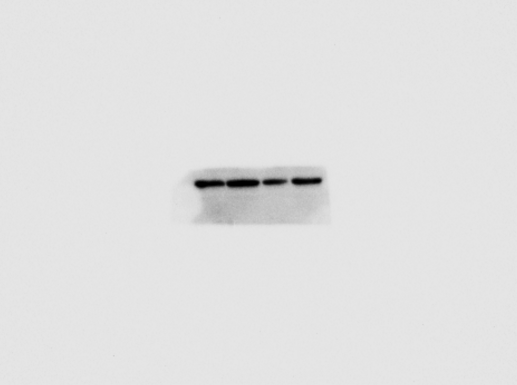


Occludin

75 kDa

55 kDa


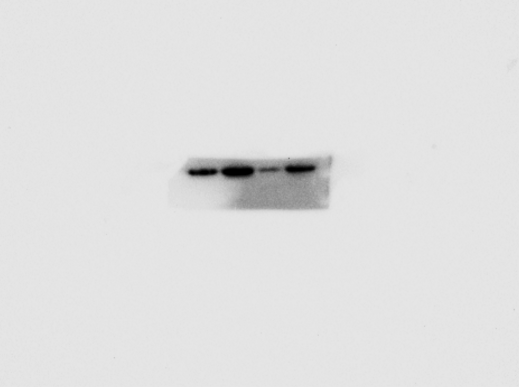


Zo-1

250 kDa

130 kDa


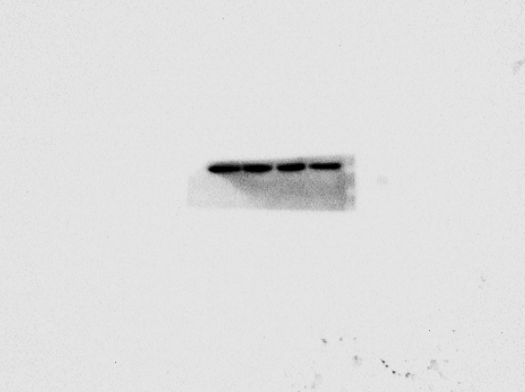


β-actin

55 kDa

43 kDa

**Fig. 4K**


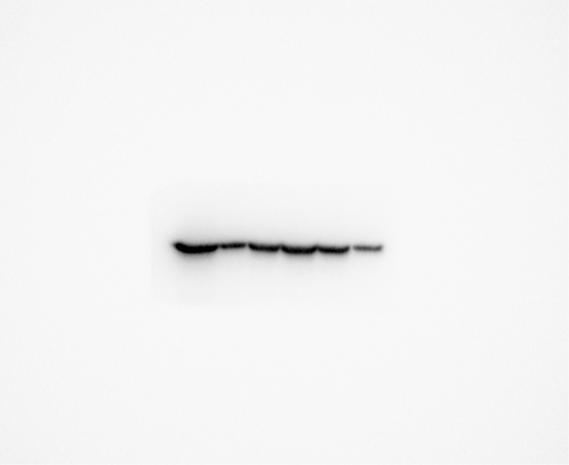


ATF4

55 kDa

43 kDa


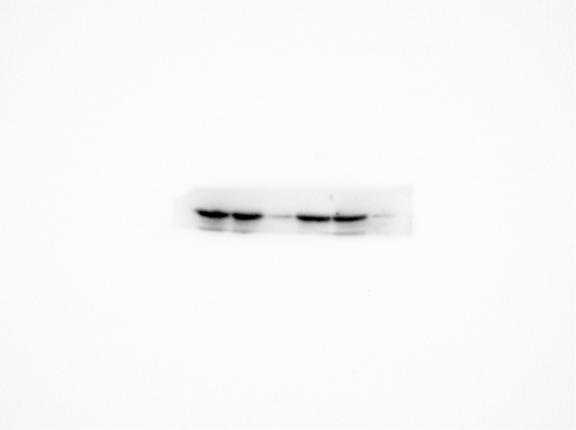


70 kDa

55 kDa

Tubulin


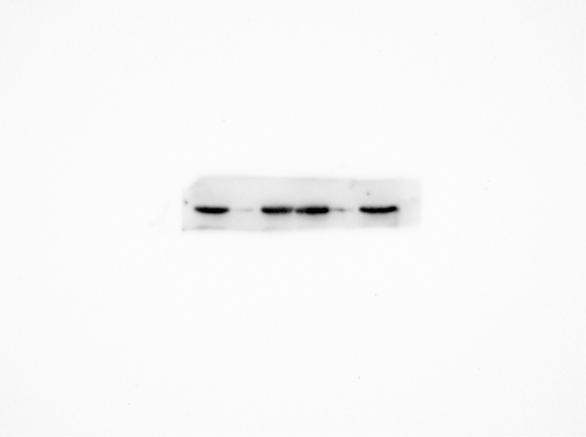


Histone

25 kDa

17 kDa

**Fig. 4S**


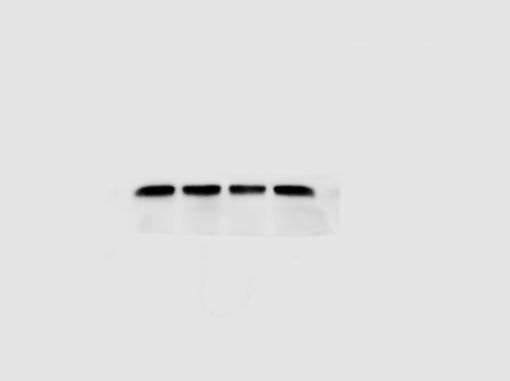


Occludin

75 kDa

55 kDa


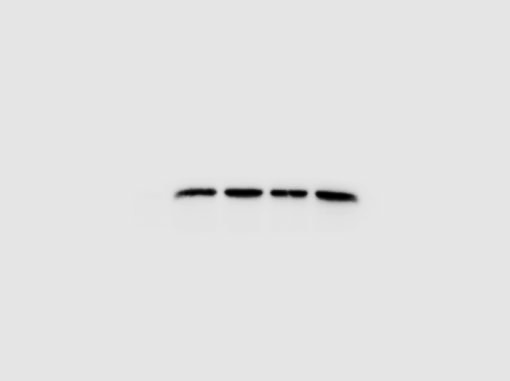


Zo-1

250 kDa

130 kDa


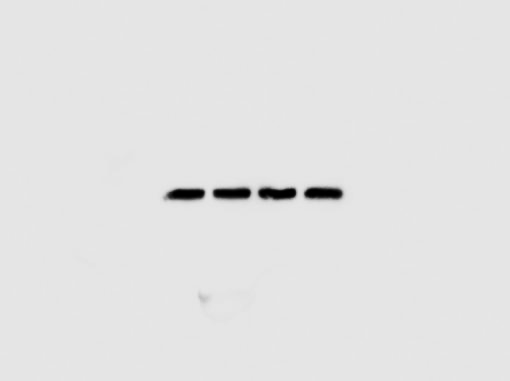


β-actin

55 kDa

43 kDa

**Fig. 4Z**


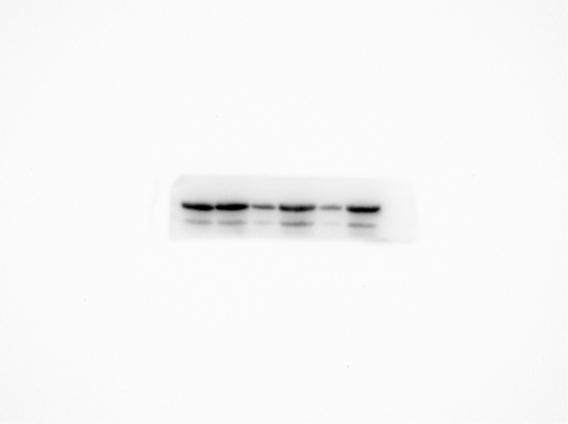


ATF4

55 kDa

43 kDa


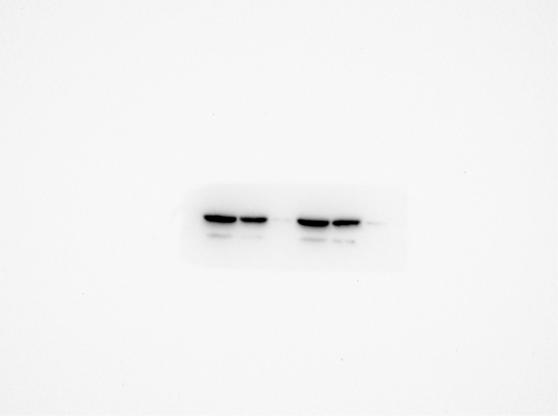


Tubulin

70 kDa

55 kDa


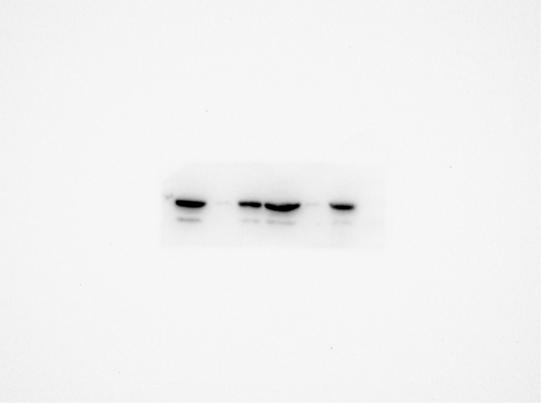


Histone

25 kDa

17 kDa

**Fig. 5H**


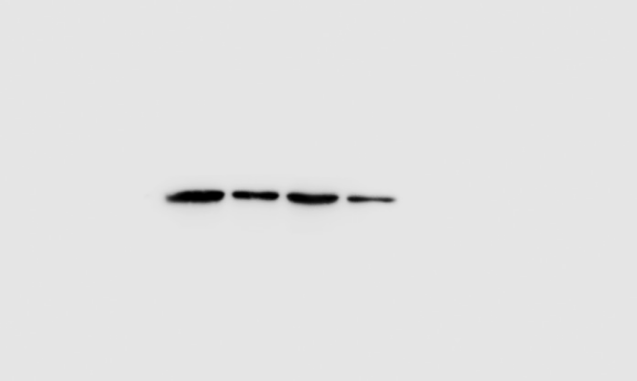


250 kDa

130 kDa

Zo-1


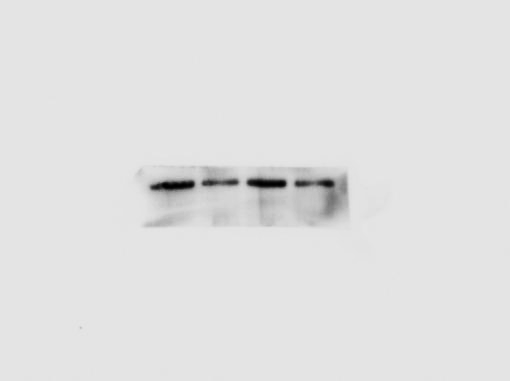


Occludin

75 kDa

55 kDa


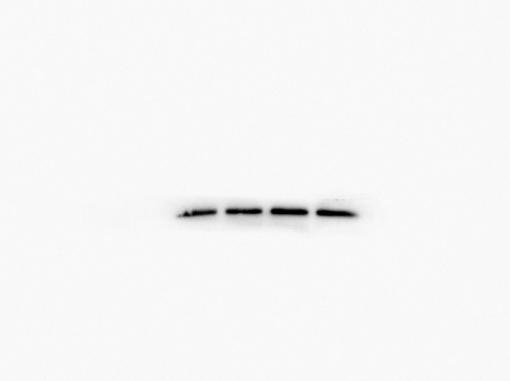


55 kDa

43 kDa

β-actin

**Fig. 5W**


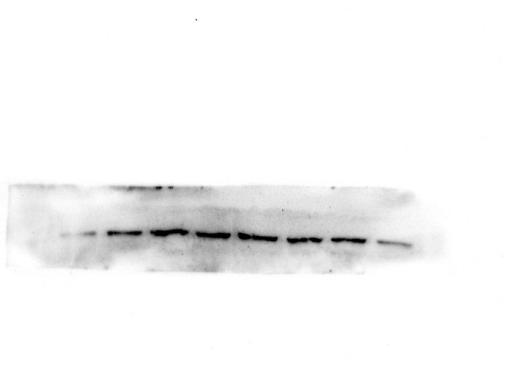


75 kDa

55 kDa


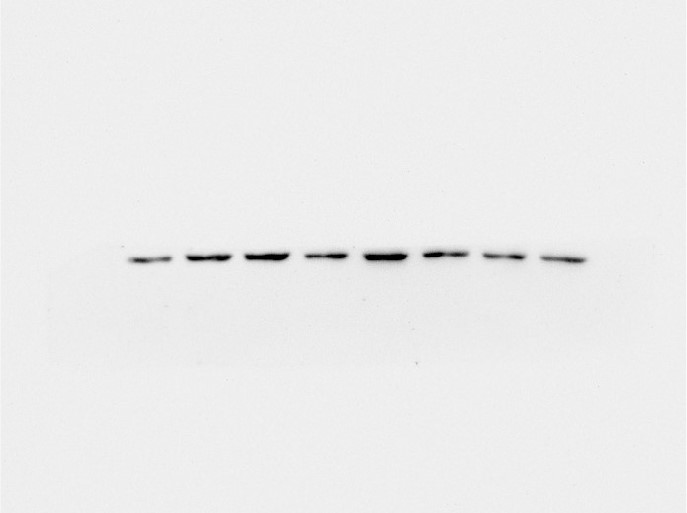


250 kDa

130 kDa

Zo-1

Occludin


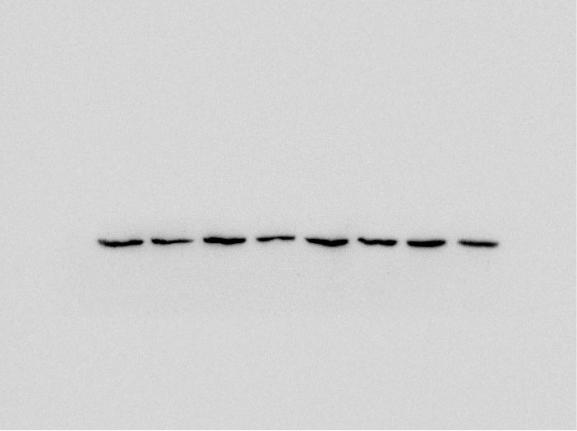


55 kDa

43 kDa

β-actin

**Fig. 6F**


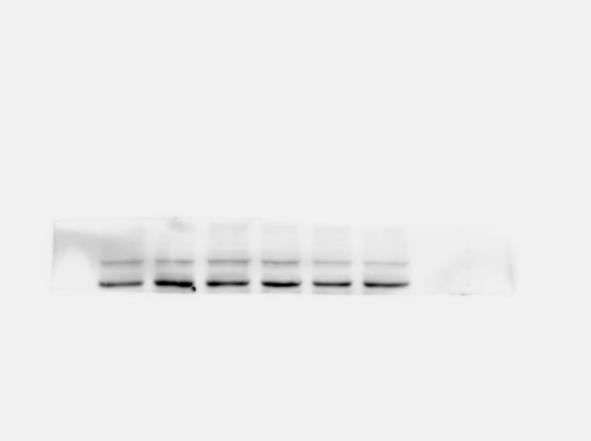


Occludin

75 kDa

55 kDa


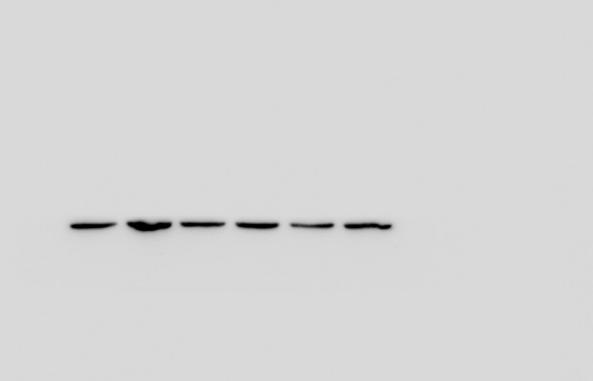


Zo-1

250 kDa

130 kDa


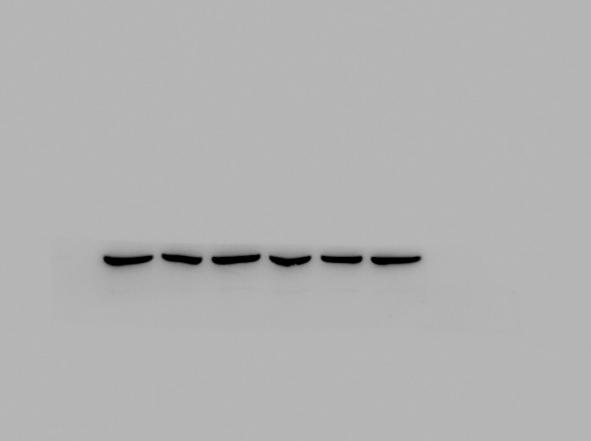


55 kDa

43 kDa

β-actin

**Supplementary Tables**

**Table S1.** The clinical data of infants with NEC.

|  | NEC group (*n* = 7) |
| --- | --- |
| NEC complications in infants |  |
| NEC Bell’s diagnosed stage Ib | 1/7 |
| NEC Bell’s diagnosed stage IIa | 3/7 |
| NEC Bell’s diagnosed stage IIb | 2/7 |
| NEC Bell’s diagnosed stage IIIa | 1/7 |
| NEC Bell’s diagnosed stage IIIb | - |

**Table S2.** The clinical data of the controls and infants with NEC.

|  | Control (*n* = 9) | NEC (*n* = 7) |
| --- | --- | --- |
| Gestational age (weeks) | 37.0 ± 2.244 | 30.9 ± 2.244* |
| Weight (g) | 3.47 ± 0.456 | 1.70 ± 0.456** |
| **Infant sex** |  |  |
| Female | 3/9 | 3/7 |
| Male | 6/9 | 4/7 |
| **Infant medical information** |  |  |
| Abdominal distension | 6/9 | 5/7 |
| Bloody stools | - | 3/7 |
| Ascites | 3/9 | 3/7 |
| **Diagnosis** |  |  |
| Congenital megacolon | 5/9 | - |
| Duodenal obstruction | 2/9 | - |
| Ileal atresia/Anal atresia | 2/9 | - |
| **NEC disease features** |  |  |
| Intestinal dilation | - | 4/7 |
| Pneumatosis | - | 4/7 |
| Pneumoperitoneum | - | 4/7 |
| Metabolic acidosis | - | 4/7 |
| **Region of bowel resected** |  |  |
| Small bowel resected | 3/9 | - |
| Large bowel resected | 4/9 | 4/7 |
| Combined large and small bowel resected | 2/9 | 3/7 |

The age-matched control and NEC groups contained infants who received an intestinal resection surgery. Data are represented as mean ± standard error of the mean (SEM). **p* < 0.05, **p* < 0.01, using a Student’s *t-*test.

**Table S3.** The antibodies and regents used in this study.

| **Antibodies** | **Source** | **Identifier** |
| --- | --- | --- |
| PE anti-human LOX-1 | BioLegend | 358604 |
| Ghost Dye Violet 510 | TONBO | 13-0870 |
| PE-eF610 anti-mouse CD45 | eBioscience | 61-0451-82 |
| APC anti-mouse CD4 | TONBO | 20-0042 |
| PE anti-mouse Foxp3 | eBioscience | 12-5773-82 |
| EF660 anti-mouse/rat IL-17A | eBioscience | 50-7177-82 |
| PB anti-mouse CD11b | BioLegend | 101224 |
| PE-Cy7 anti-mouse Ly-6G | eBioscience | 25-9668-82 |
| Percp-Cy5.5 anti-mouse Ly-6C | eBioscience | 45-5932-82 |
| PE anti-mouse CD326 | eBioscience | 12-9326-42 |
| APC anti-mouse Ki67 | ebioscience | 17-5698-82 |
| PE Anti-Hu/Mo Phospho-ERK1/2 | ebioscience | 12-9109-42 |
| APC Annexin V | BioLegend | 640920 |
| 7AAD cell Viability | BioLegend | 64093 |
| Alexa Fluor 647 anti-mouse GPX4 | Santa | sc-166570 |
| PE anti-mouse ACSL4 | Santa | sc-365230 |
| Rabbit xCT (SLC7A11) | Affinity | DF12509 |
| PE Donkey anti-Rabbit lgG | BioLegend | 406421 |
| Rabbit anti mouse/human GPX4 | Abmart | ab166570 |
| Biotin anti-mouse Ly-6G | BioLegend | 127604 |
| Biotin anti-mouse CD326 | BioLegend | 118210 |
| Biotin anti-human CD3 | BioLegend | 317320 |
| Alexa Fluor 488-goat anti-mouse IgG (H + L) | Abcam | ab150077 |
| m-1gGk BP CFL-647 | Santa | sc-516179 |
| Mouse ATF4 (B-3) | Santa | sc-390063 |
| Rabbit Occludin Recombinant mAb | Selleck | A5381 |
| Rabbit mTOR | Selleck | F0169 |
| Rabbit Phospho-AMPKα (Thr 172) | Selleck | F0151 |
| Rabbit anti mouse/human OFLM4 | Zen Bioscience | R382425 |
| Rabbit ZO-1 tight junction protein | Zen Bioscience | 164329 |
| Mouse monoclonal anti-beta-actin | Zen Bioscience | T200068-8F10 |
| Rabbit Phospho-p38 (Thr180/Tyr182) | Zen Bioscience | 310091 |
| ***Continued*** |  |  |
| Mouse MAPK (ERK1/2)4A4 | Zen Bioscience | 201245-4A4 |
| Rabbit ATF4 (D4B8) | Cell Signaling Technology | 11815 |
| **Regents** | **Source** | **Identifier** |
| Collagenase IV | Gibco | 17104019 |
| Dnase I | Solarbio | D8071 |
| Dithiothreitol | Amresco | P001008 |
| Penicillin-Streptomycin Solution | Biological Industries | 03-031-1B |
| C11-BODIPY (581/591) | Invitrogen | D3861 |
| DCFDA | Thermo | C369 |
| Foxp3/Transcription Factor Fix/Perm Diluent (1×) | TONBO | TNB-1022-L160 |
| Foxp3/Transcription Factor Fix/Perm Concentrate (4×) | TONBO | TNB-1020-L050 |
| Percoll | Cytiva | 17089109 |
| PMA | Alomone Labs | P-800 |
| Ionomycin | Alomone Labs | I-700 |
| Brefeldin A | Alomone Labs | B-275 |
| Streptavidin Particles | BD Biosciences | 557812 |
| RIPA Lysis Buffer | Beyotime | P003B |
| RNAEx ZOL Reagent | ECOTOP | EK-5301 |
| 2 × RealStar Universal SYBR | Genstar | A308 |
| StarScript II RT Kit | Genstar | A214 |
| DAPI Staining Solution | Beyotime | C1005 |
| HRP substrate | Millipore | WBKLS0500 |
| BCA Protein Assay Kit | Beytiome | P10010 |
| H&E staining | Servicebio | G1005 |
| Anti-mouse IL-18-InVivo | Selleck | A2134 |
| Mouse IL-18 Recombinant mAb | Novoprotein | CK06 |
| Mouse IL-18 ELISA Kit | Dogesce | DG30613M-96T |
| Indole-3-carbinol (I3C) | InvivoChem | 700-06-1 |
| Indole-3-propionic acid (IPA) | InvivoChem | 830-96-6 |
| Indole-3-carboxaldehyde (I3A) | InvivoChem | 487-89-8 |
| Ferrostatin-1 (Fer-1) | Selleck | 347174-05-4 |
| Puppy Milk Replacer Powder | PetAg | - |
| *Continued* |  |  |
| Infant Formula Powder | Similac Advance | - |
| FITC-dextran 70 (FD70) | Beyotime | ST2947 |
| Indole-3-carboxaldehyde ELISA KIT | MEIMIAN | MM-92874501-96T |
| Indole-3-carbinol ELISA KIT | Huabodeyi biology science | HBDY-927392O2-48T |
| Indole-3-propionic acid ELISA KIT | Huabodeyi biology science | HBDY-926075O2-48T |
| CH-223191 | Selleck | S77111 |
| PageRuler Plus Prestained Protein Ladder | Thermo | 26619 |
| Neomycin sulfate | OriLeaf | S17028 |
| Ampicillin sodium salt | OriLeaf | S17018 |
| Vancomycin hydrochloride | MACKLIN | V820413 |
| Metronidazole | MACKLIN | M813526 |

**Table S4.** The sequences of primers used in this study.

| **Gene** | | **Forward primer (5’-3’)** | **Reverse primer (3’-5’)** |
| --- | --- | --- | --- |
| **Mice** | | | |
| m*-Occludin* | | TTGAAAGTCCACCTCCTTACAGA | CCGGATAAAAAGAGTACGCTGG |
| m*-Cldn2* | | TTCGGGACTTCTACTCGCC | TCCGGCTACCAAAGAAAACAG |
| m*-Cldn3* | | ACCAACTGCGTACAAGACGAG | CGGGCACCAACGGGTTATAG |
| m*-Cldn4* | | GGAGGGCCTCTGGATGAACT | GATGCTGATGACCATAAGGGC |
| m*-Zo-1* | | TGCCAGCTTTAAGCCTCCG | GGGTGGCTTCACTTGAGGTT |
| m*-Olfm4* | | CATGCTCGAAGTGGAGATAAGGA | CGCCACCATGACTACAGCTT |
| m*-Vcl* | | TTCGATGAGGCTGAGGTTCG | CGGTGTTCCTGGTGAGTCAA |
| m*-Itgb6* | | ACTGTCTTGGTAGGTAACCTTCA | TGGCTTCATAGCAGTTGCCAC |
| m*-Femt1* | | CAAGATGAATGCAGGCTGGC | GGCTTGCTCGTAGAGTTGGT |
| m*-Il18* | | CCTCTTGGCCCAGGAACAAT | ACAGTGAAGTCGGCCAAAGT |
| m*-Gpx4* | | GTGGACCTGGACGCCAAAG | TCGCGGGATGCACACAAG |
| m-Atf4 | | TCCCTGGCCGAGGCTATAAA | CTGCTGCCTCTAATACGCCA |
| m*-Slc3a2* | | TGATGAATGCACCCTTGTACTTG | GCTCCCCAGTGAAAGTGGA |
| m*-Slc7a11* | | GGCACCGTCATCGGATCAG | CTCCACAGGCAGACCAGAAAA |
| m*-Acsl4* | | CCTTTGGCTCATGTGCTGGAAC | GCCATAAGTGTGGGTTTCAGTAC |
| m*-S100a8* | | GGAAATCACCATGCCCTCT | TTTATCACCATCGCAAGGAAC |
| m*-S100a9* | | AATGGTGGAAGCACAGTTGG | GCTGATTGTCCTGGTTTGTG |
| m*-Arg1* | | ATTATCGGAGCGCCTTTCTC | ACAGACCGTGGGTTCTTCAC |
| m*-Cybb* | | ATGAGGCTGCTCATCCCTTCCTTGA | CTTTTCTCATCTCGTTCTGCCACC |
| m*-Ahr* | AGCCGGTGCAGAAAACAGTAA | AGGCGGTCTAACTCTGTGTTC |  |
| m*-Cyp1a1* | CAATGAGTTTGGGGAGGTTACTG | CCCTTCTCAAATGTCCTGTAGTG |  |
| **Human** | | | |
| H*-OLFM4* | | AAATGCTCGAGAGTTGCGGA | CACAGCAATCGTGTTGGTGG |
| H*-GPX4* | | TCACCAAGTTTGGACACCGT | ATAGTGGGGCAGGTCCTTCT |
| H*-ATF4* | | GGACTTGATGTCCCCCTTCG | AGAAGGCATCCTCCTTGCTG |
| H*-SLC7A11* | | TGGAACGAGGAGGTGGAGAA | TGTGCTTTTTCCTTCACAGCG |
| ***Continued*** | |  |  |
| H*-SLC3A2* | | ACCCCTGTTTTCAGCTACGG | GGTCTTCACTCTGGCCCTTC |

**Table S5.** The primers of ChIP-qPCR used in this study.

| **Bacteria** | **Forward primer (5’-3’)** | **Reverse primer (3’-5’)** |
| --- | --- | --- |
| *Slc7a11-Chip-site-1* | GTAGTATGACAAGAGACAGA | CCCTTCCACACTTTAGTC |
| *Slc7a11-Chip-site-2* | GTAGTATGACAAGAGACAGA | CCTTCCACACTTTAGTCTT |
| *Slc7a11-Chip-site-3* | GTAGTATGACAAGAGACAGA | TTCCACACTTTAGTCTTCC |
| *Gpx4-Chip-site-1* | ACTGTGATTGTGGGCTTTAAGA | AGGAGGAGCTGAGAGTTCTAC |
| *Il18-Chip-site-1* | TCTGGCTGCATCATTCATTC | GCTAGGCTGGAGTGAACA |

**Table S6.** The sequences of primers used in this study.

| **Bacteria** | **Forward primer (5’-3’)** | **Reverse primer (3’-5’)** |
| --- | --- | --- |
| *16S-UniF340 R514* | ACTCCTACGGGAGGCAGCAGT | ATTACCGCGGCTGCTGGC |
| *L.reuteri* | TCGTGTCGTGAGATGTTGGG | GATGATCTGACGTCGTCCCC |
| *L.rhamnosus* | GCATTAAGCATTCCGCCTGG | CCTGGTAAGGTTCTTCGCGT |
| *Morganella* | CCTGGACAAAGACTGACGCT | TTTAACCTTGCGGCCGTACT |
