# Supplementary material for: Probiotic interventions maintain intestinal barrier function and alleviate necrotizing enterocolitis by inhibiting ferroptosis in intestinal PMN-MDSCs
Source: Cell Death Dis. 2026 May 15;17(1):623. doi: 10.1038/s41419-026-08869-w (PMC13346608; doi:10.1038/s41419-026-08869-w)
Supplement: Supplementary file 2 — Original Data [file 41419_2026_8869_MOESM2_ESM.docx]

**Probiotic interventions maintain intestinal barrier function and alleviate necrotizing enterocolitis by inhibiting ferroptosis in intestinal PMN-MDSCs**

Meiqi Chen, Qing Zhao, Laiqin Peng, Ziyang Chen, Shuaijun Lv, Zekai Zhuang, Shudan Zheng, Jiaxiu Ye, Junyu He, Yizhuang Lu, Gang Xiao, Yuxiong Guo, and Yumei He

Original Western Blot Data

Original qPCR Data

**Original Western Blot Data**

This section contains the full, uncropped original Western blot images corresponding to all Western blot panels presented in the manuscript, including Figure 1K, Figure 2H, Figure 3H, Figure 4K, Figure 4S, Figure 4Z, Figure 5H, Figure 5W, and Figure 6F.

For nuclear-cytoplasmic fractionation Western blot samples, two copies of the original uncropped images are provided (Figure 4K and Figure 4Z); for all other regular Western blot data, three copies are supplied. The specific copy containing the representative blots adopted for each final manuscript figure is clearly labeled and annotated.

All raw images are presented in their unaltered, native format—without cropping, splicing, or any other post-acquisition.

**Figure 1K**

3 copies

PBS

Probiotics

PBS

Probiotics

PBS

Probiotics

Occludin


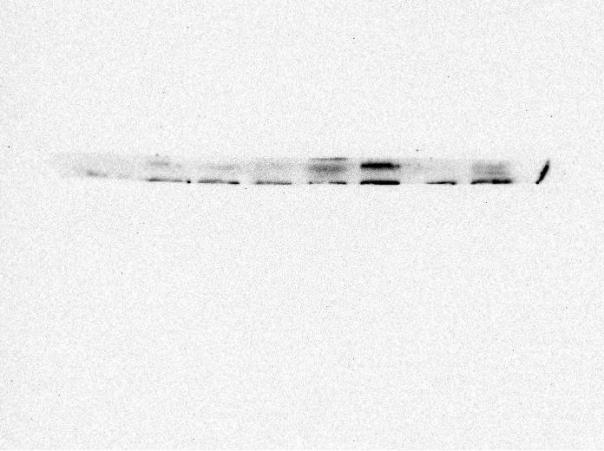


75 kDa

55 kDa


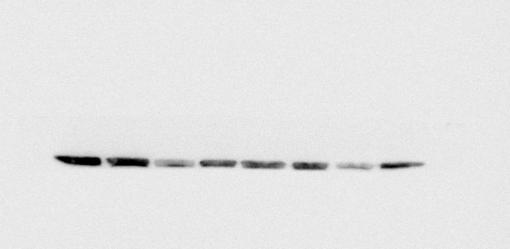


Zo-1

250 kDa

130 kDa

3 copies

PBS

Probiotics

PBS

Probiotics

PBS

Probiotics

The representative blot

230

Zo-1

α-β-actin

Occludin

65

43


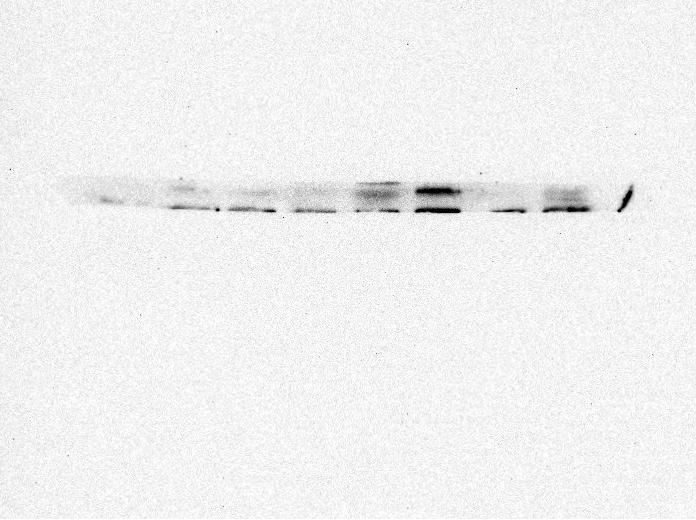

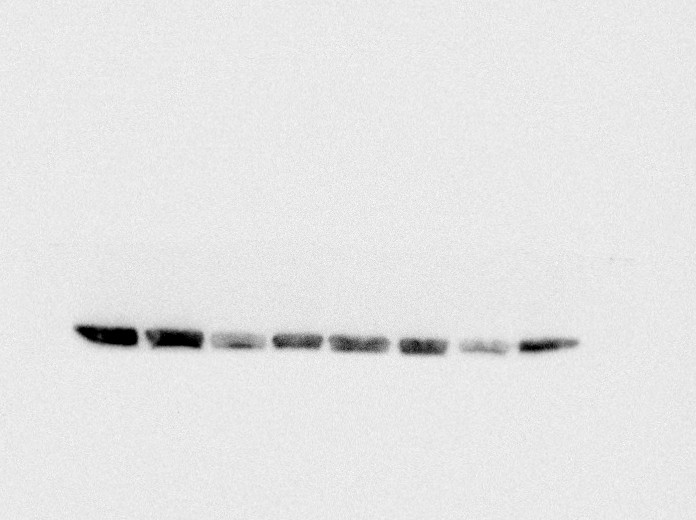

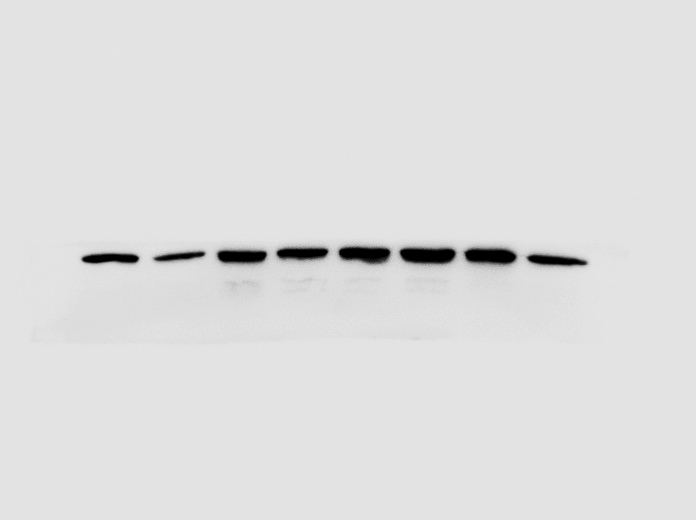


PBS

Probiotics

3 copies

PBS

Probiotics

PBS

Probiotics

PBS

Probiotics


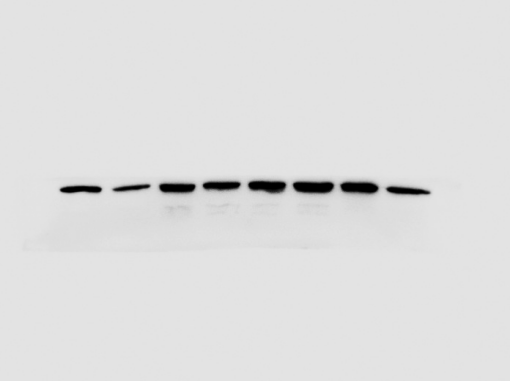


55 kDa

43 kDa

α-β-actin

**Figure 2H**

Occludin

2 copies

Olfm4^fl/fl^

Olfm4^fl/fl^S100a8^cre^

Olfm4^fl/fl^

Olfm4^fl/fl^S100a8^cre^

Olfm4^fl/fl^

Olfm4^fl/fl^S100a8^cre^

1 copy

**
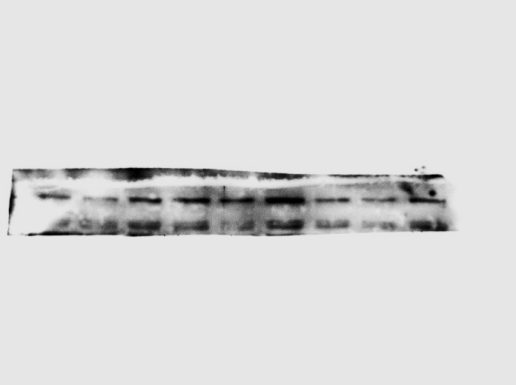
**

75 kDa

55 kDa

Zo-1

2 copies

Olfm4^fl/fl^

Olfm4^fl/fl^S100a8^cre^

Olfm4^fl/fl^

Olfm4^fl/fl^S100a8^cre^

Olfm4^fl/fl^

Olfm4^fl/fl^S100a8^cre^

1 copy

**
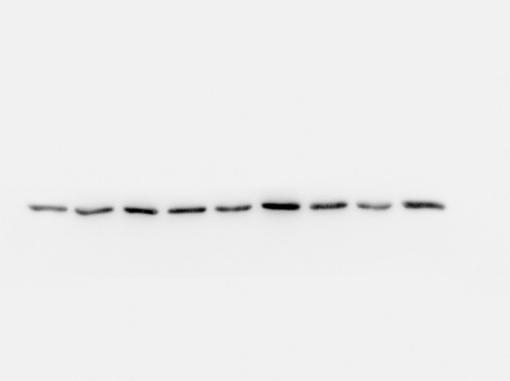
**

The representative blot

Zo-1

α-β-actin

Occludin

65

230

43


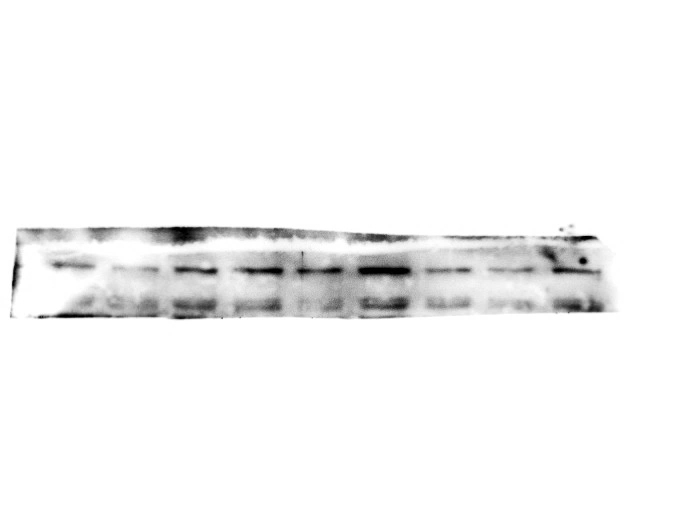

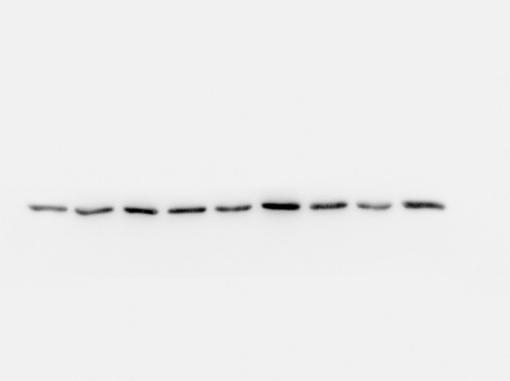

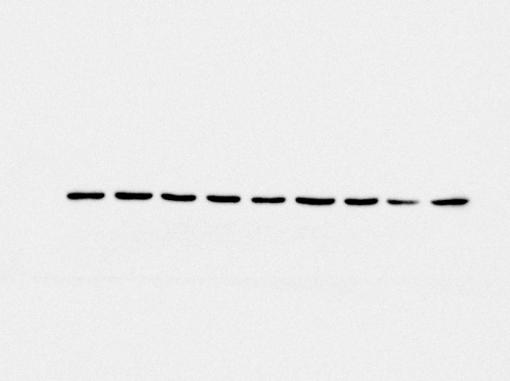


Olfm4^fl/fl^

Olfm4^fl/fl^S100a8^cre^

250 kDa

130 kDa

α-β-actin

2 copies

Olfm4^fl/fl^

Olfm4^fl/fl^S100a8^cre^

Olfm4^fl/fl^

Olfm4^fl/fl^S100a8^cre^

Olfm4^fl/fl^

Olfm4^fl/fl^S100a8^cre^

1 copy

**
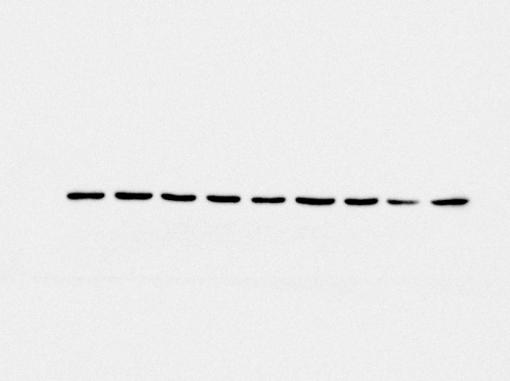
**

55 kDa

43 kDa

**Figure 3H**

1 copy

PBS

Probiotics

ABX

ABX+Probiotics

Occludin

Occludin

PBS

2 copies

PBS

Probiotics

ABX

ABX+Probiotics

Probiotics

ABX

ABX+Probiotics

**
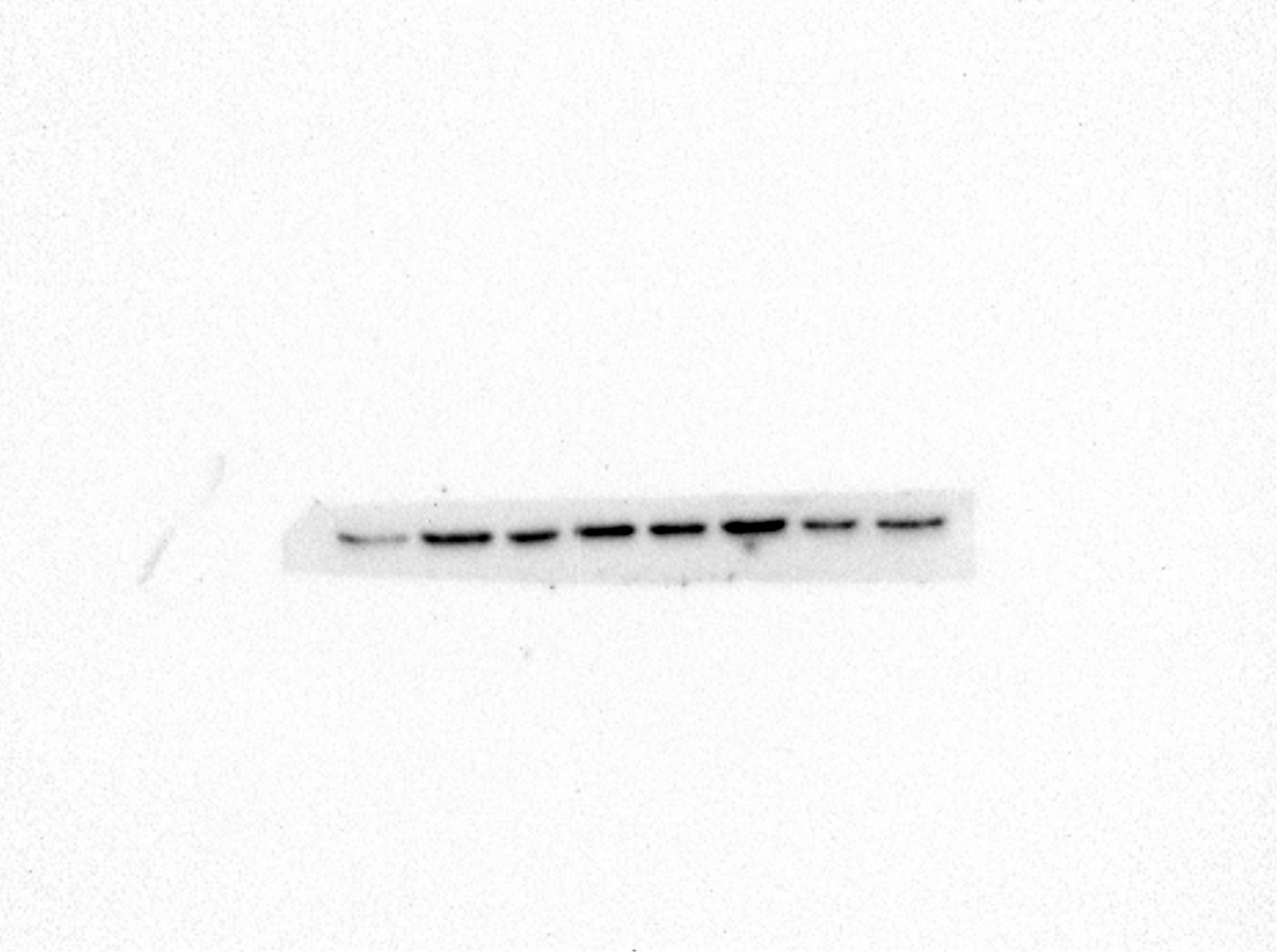
**


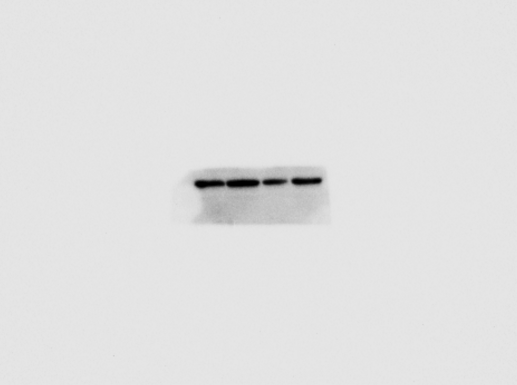


75 kDa

55 kDa

75 kDa

55 kDa

Zo-1

PBS

2 copies

PBS

Probiotics

ABX

ABX+Probiotics

Probiotics

ABX

ABX+Probiotics

**
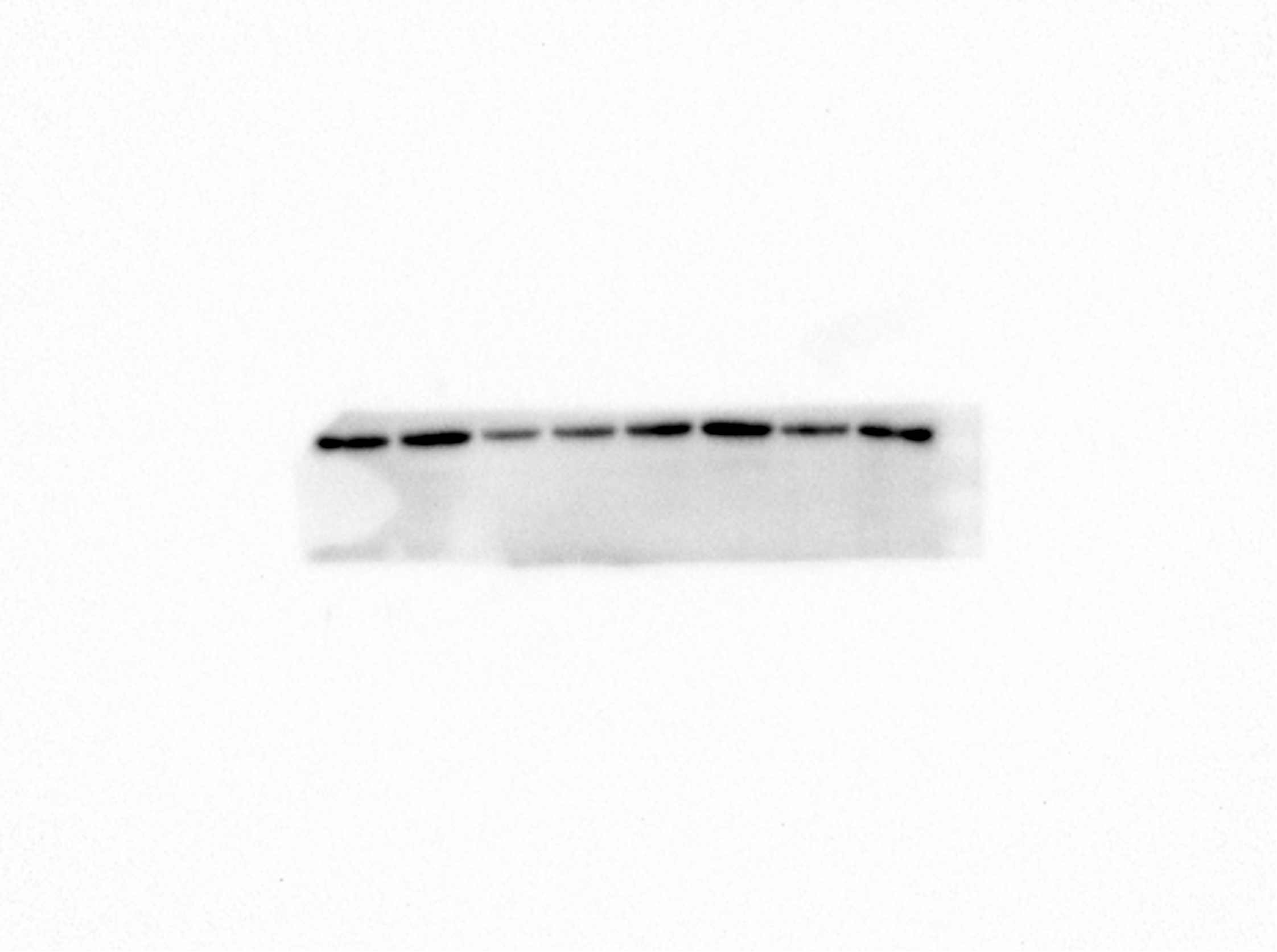
**


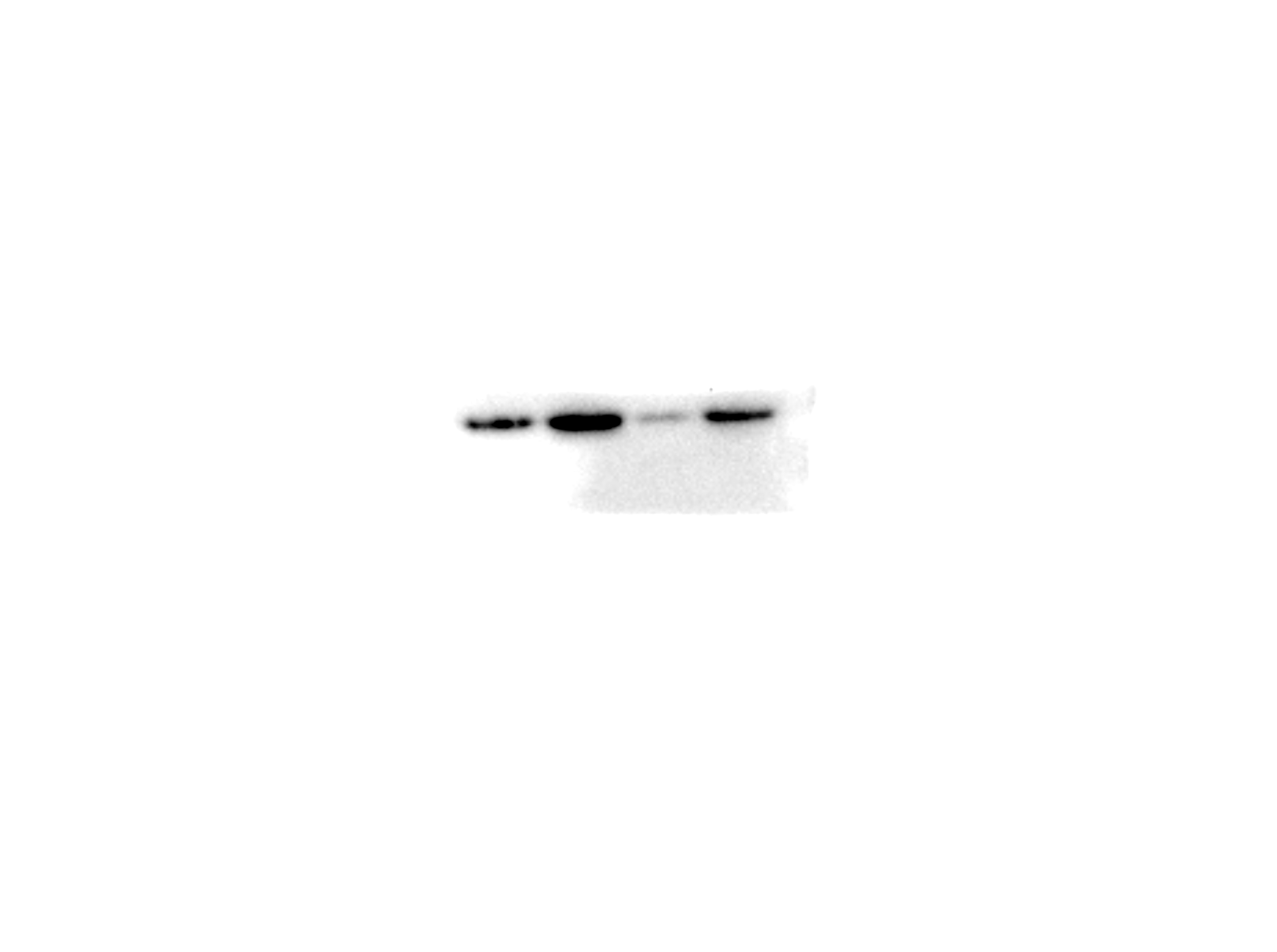


Zo-1

α-β-actin

Occludin


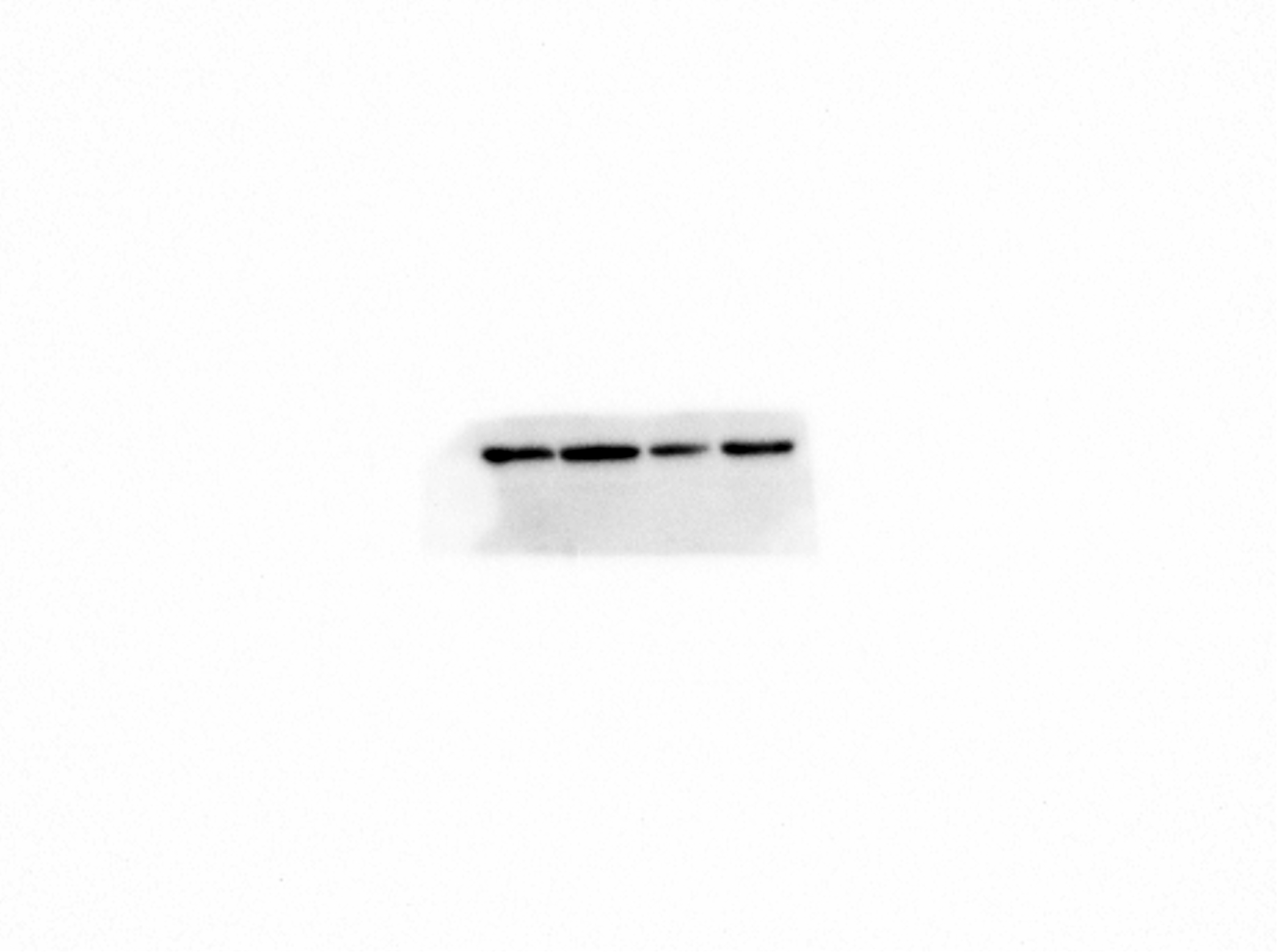


65

230

43


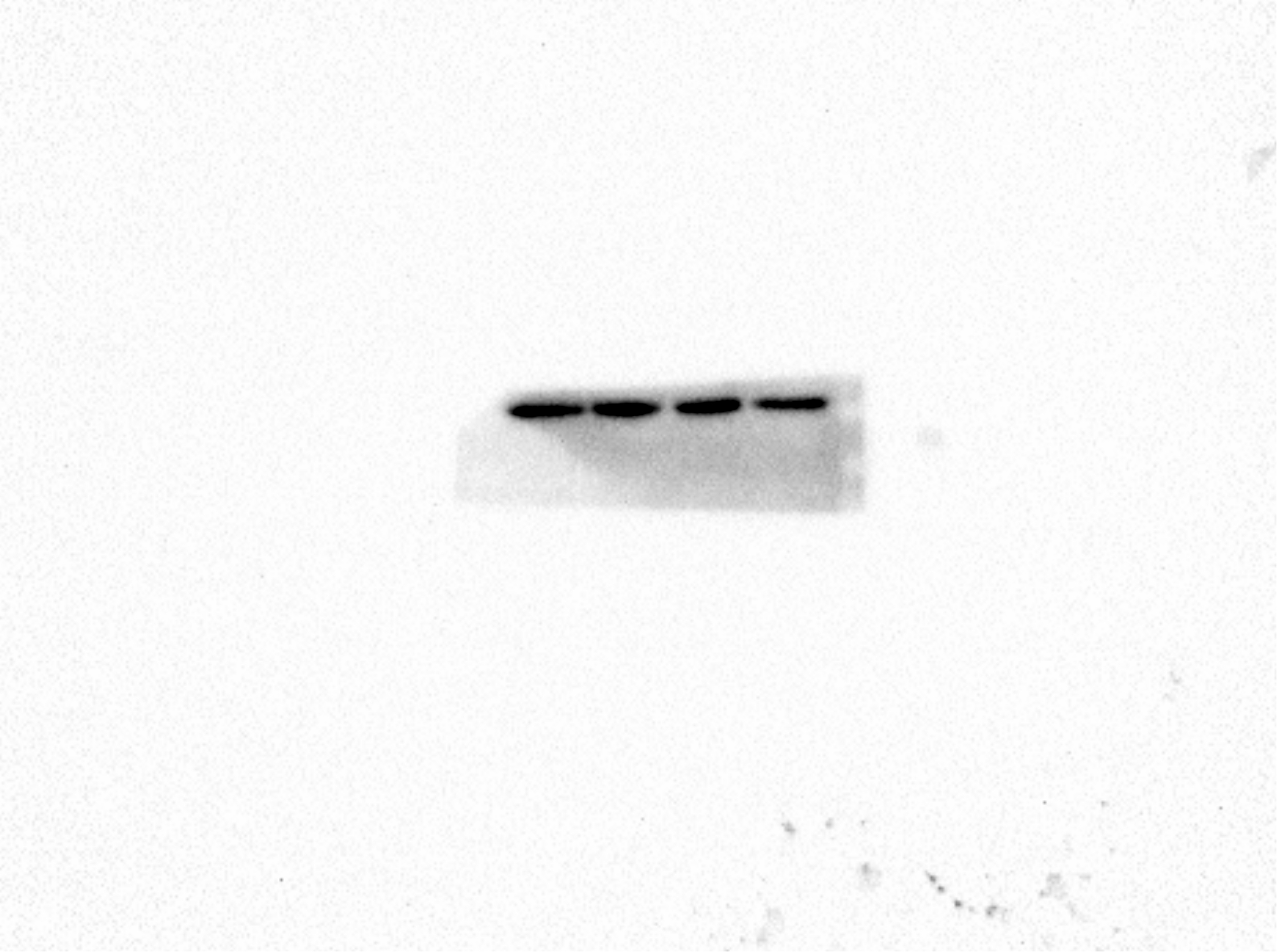


PBS

ABX+Probiotics

Probiotics

ABX

250 kDa

130 kDa

The representative blot

Zo-1

1 copy

PBS

Probiotics

ABX

ABX+Probiotics


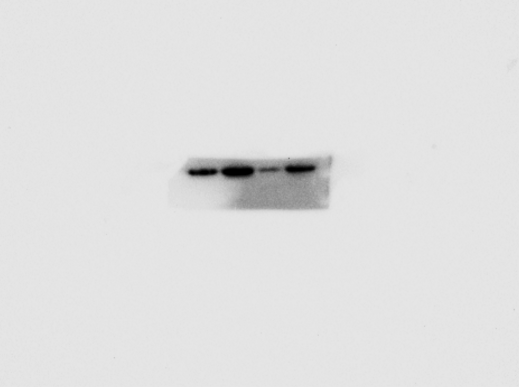


250 kDa

130 kDa

1 copy

PBS

Probiotics

ABX

ABX+Probiotics

α-β-actin

α-β-actin

PBS

2 copies

PBS

Probiotics

ABX

ABX+Probiotics

Probiotics

ABX

ABX+Probiotics

**
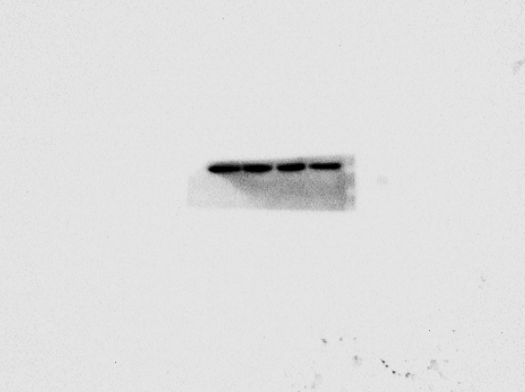
**

55 kDa

43 kDa

**
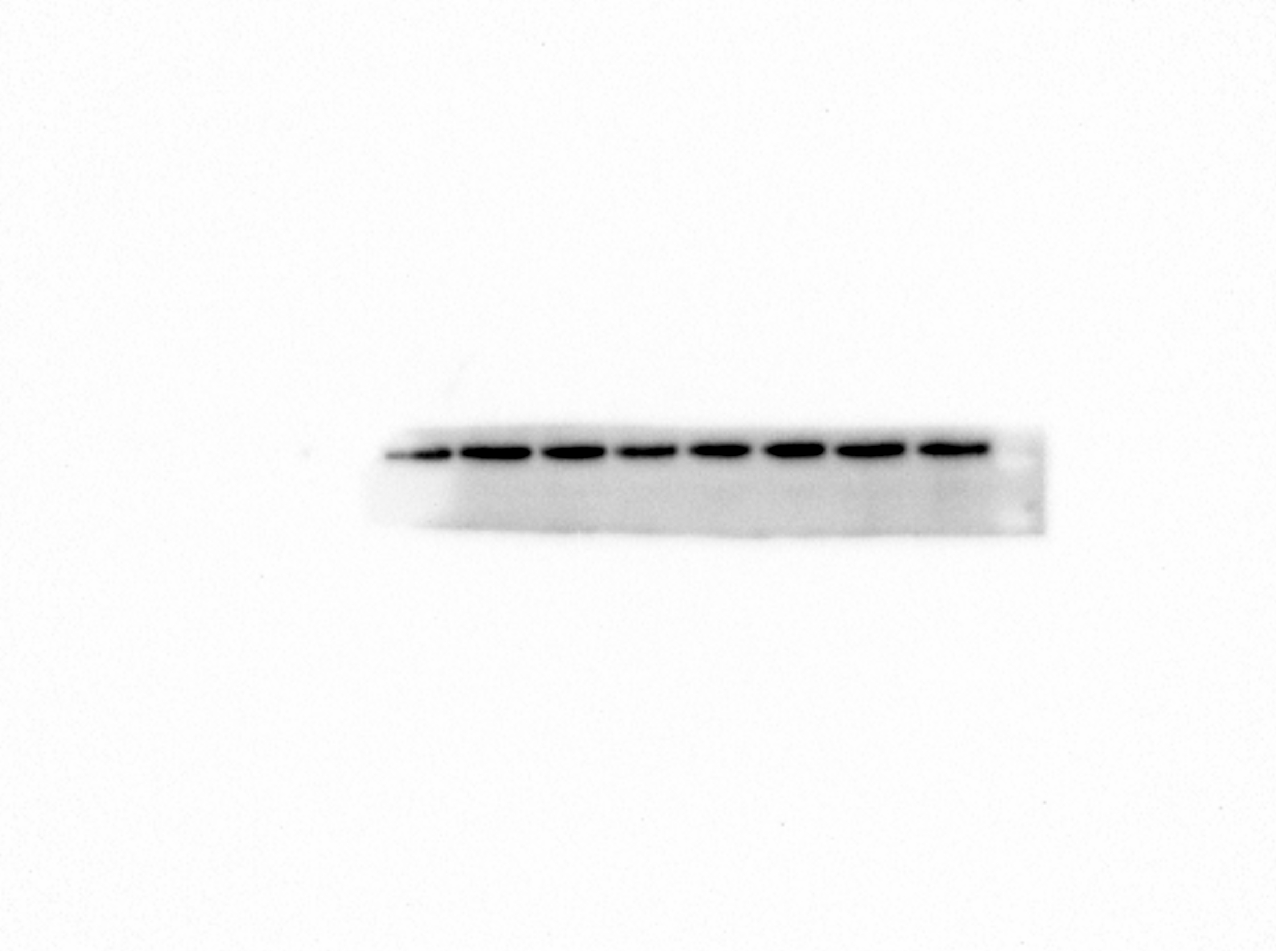
**

55 kDa

43 kDa

**Figure 4K**

**
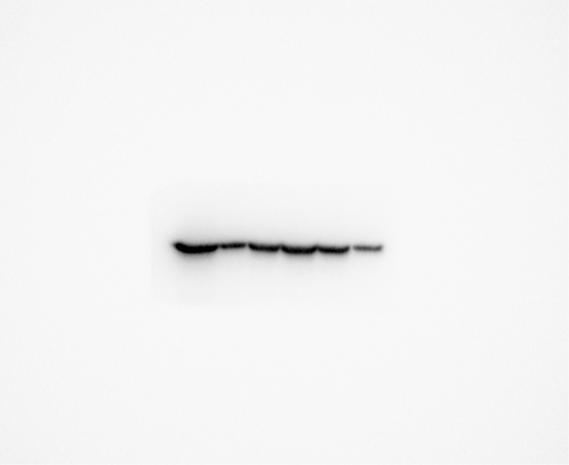
**

ATF4

1 copy

Olfm4^fl/fl^

Olfm4^fl/fl^S100a8^cre^

1 copy

Olfm4^fl/fl^

Olfm4^fl/fl^S100a8^cre^

ATF4


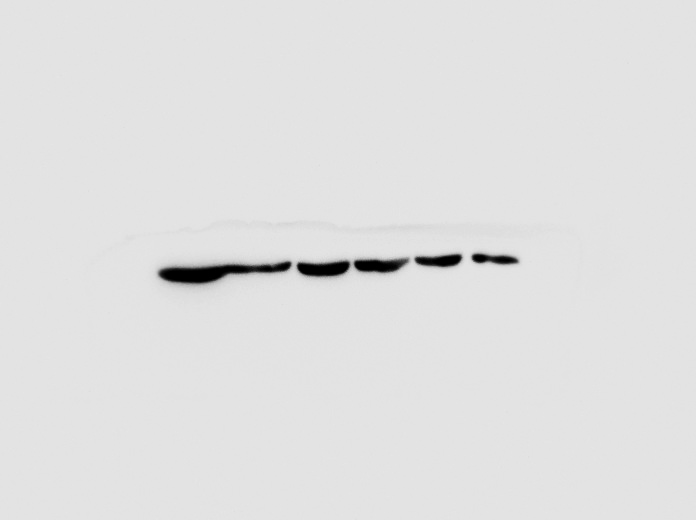


55 kDa

43 kDa

55 kDa

43 kDa

1 copy

Olfm4^fl/fl^

Olfm4^fl/fl^S100a8^cre^

Tubulin

The representative blot


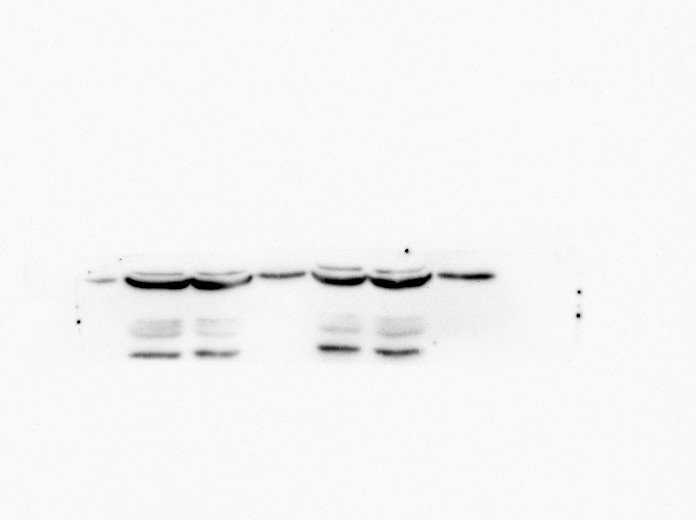


70 kDa

55 kDa

WCL

ATF4

Tubulin

Histone

C

N

WCL

C

N

49

55

17


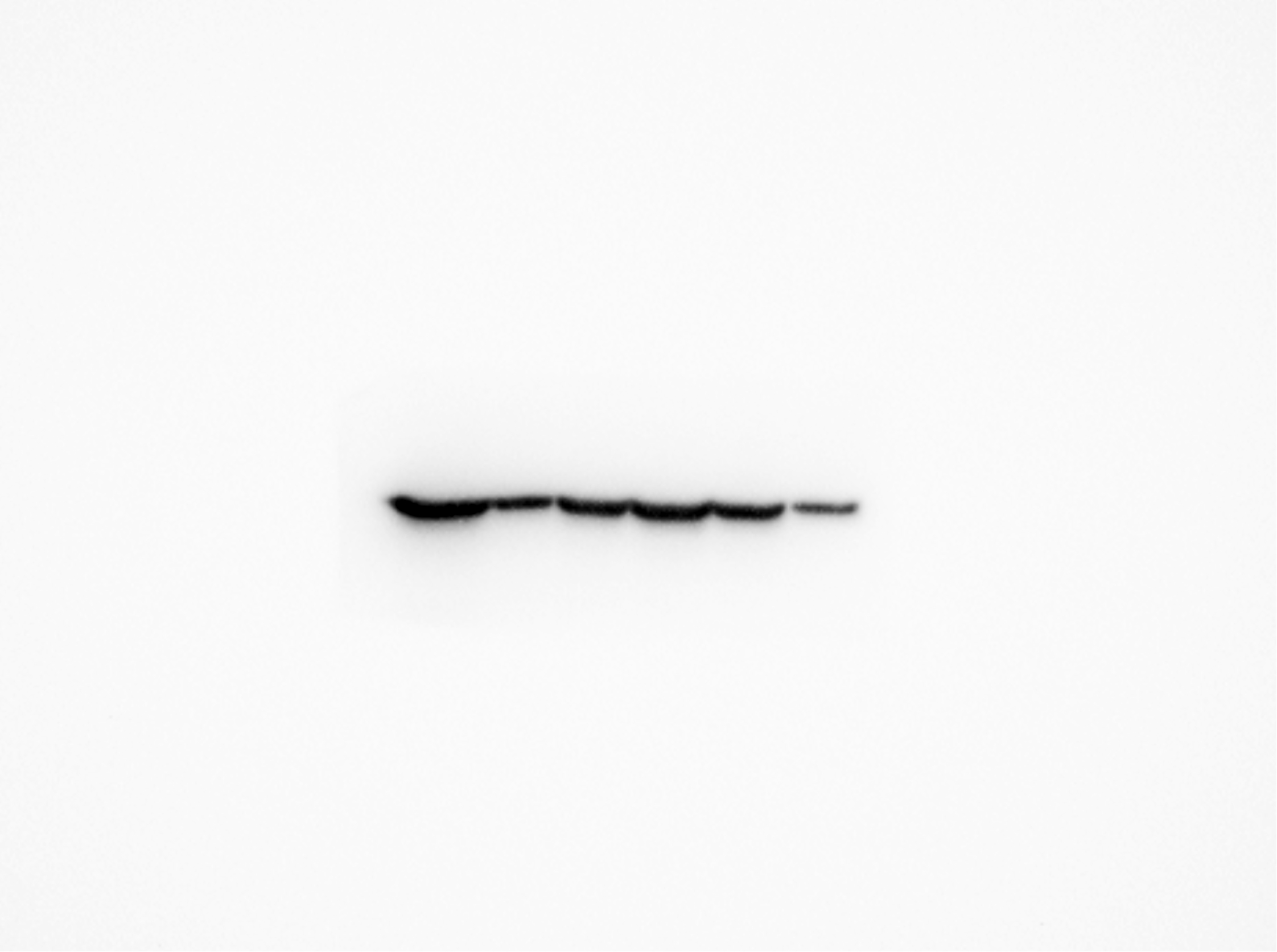

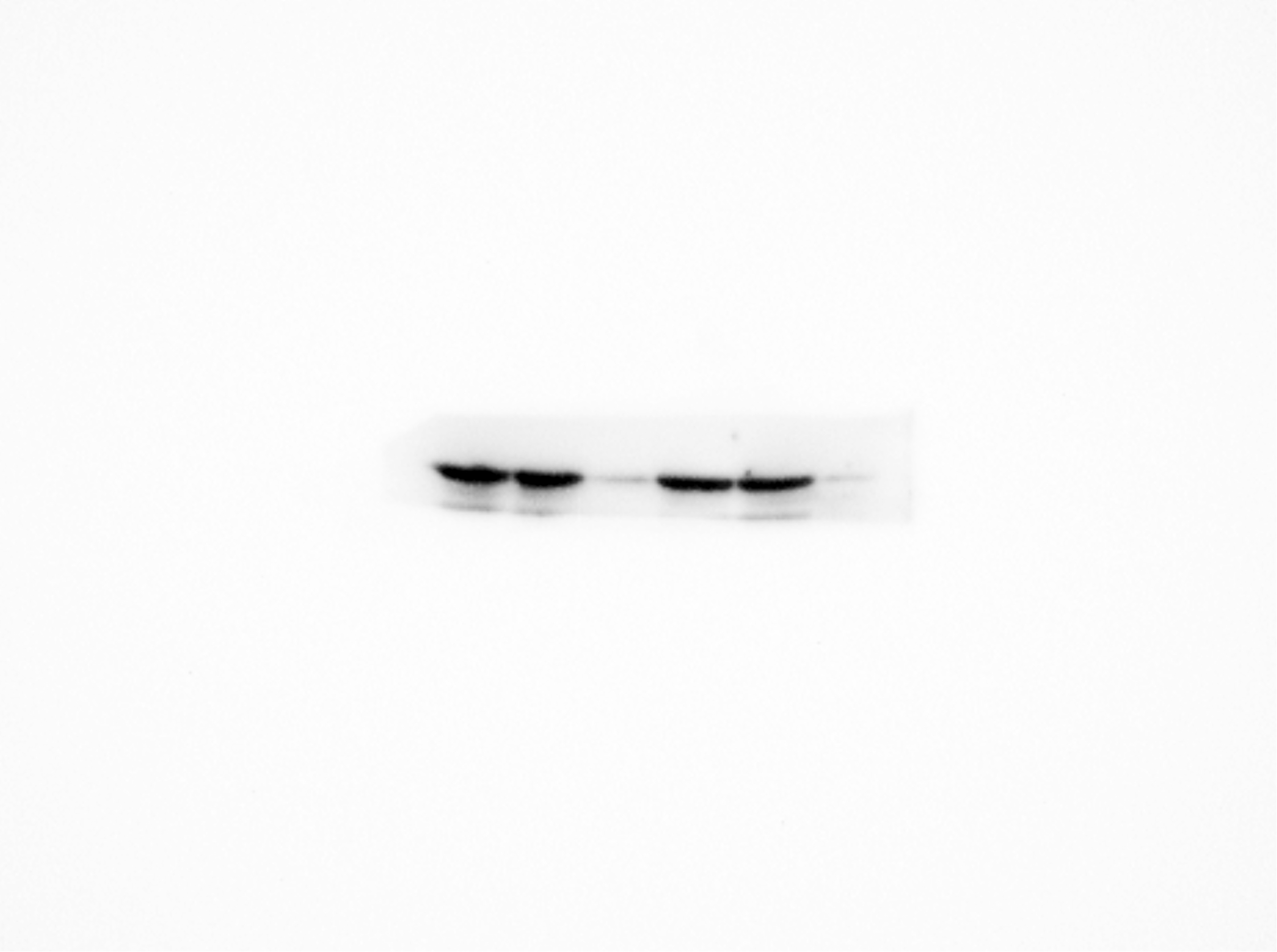

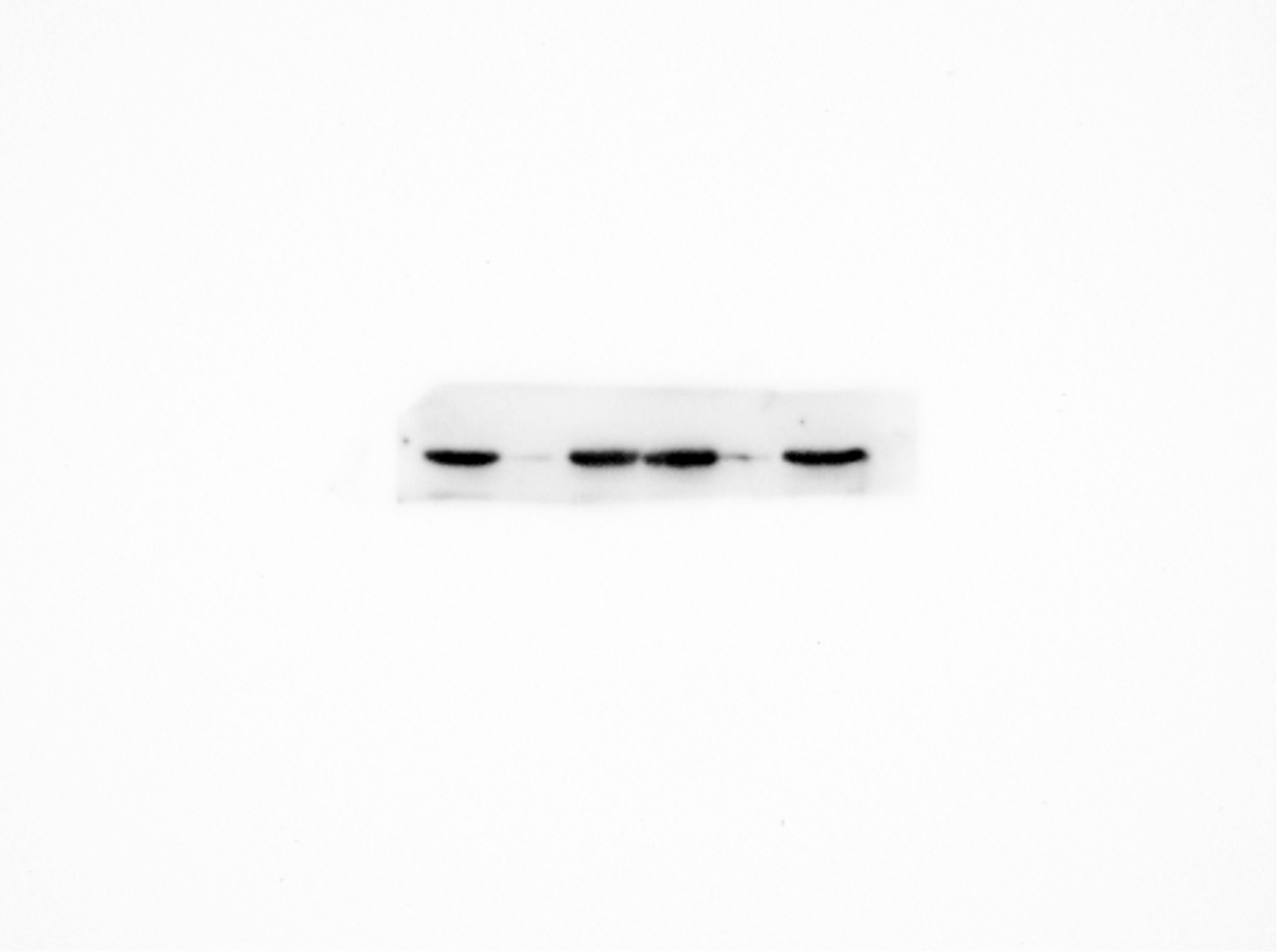


Olfm4^fl/fl^

Olfm4^fl/fl^S100a8^cre^

1 copy

Olfm4^fl/fl^

Olfm4^fl/fl^S100a8^cre^

Tubulin

**
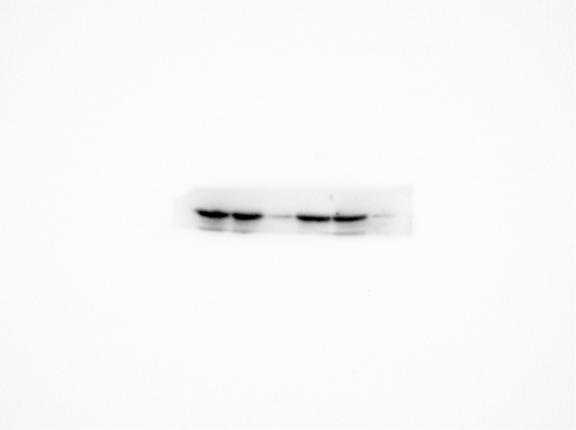
**

70 kDa

55 kDa

Histone

1 copy

Olfm4^fl/fl^

Olfm4^fl/fl^S100a8^cre^

1 copy

Olfm4^fl/fl^

Olfm4^fl/fl^S100a8^cre^

Histone


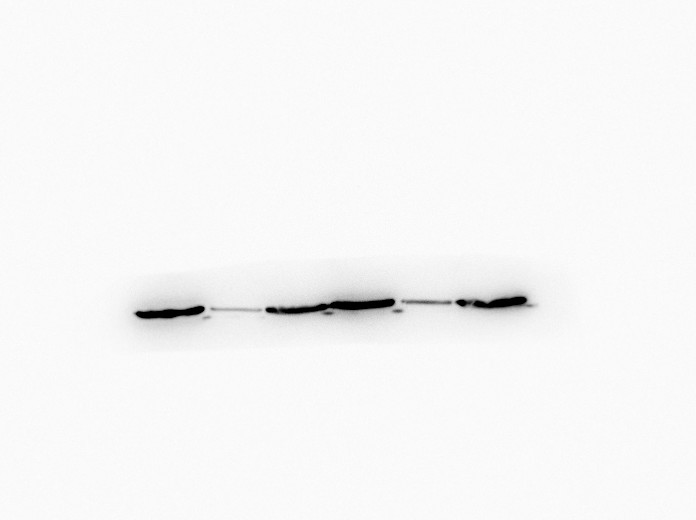


25 kDa

17 kDa

**
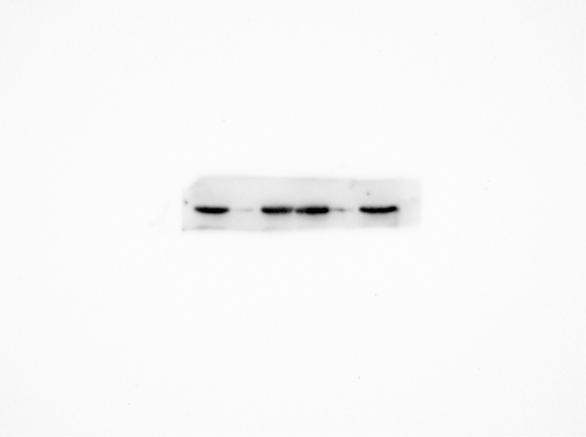
**

25 kDa

17 kDa

**Figure 4S**

2 copies

Olfm4^fl/fl^S100a8^cre^+DMSO

Olfm4^fl/fl^S100a8^cre^+Fer-1

Olfm4^fl/fl^S100a8^cre^+DMSO

Olfm4^fl/fl^S100a8^cre^+Fer-1

Occludin

1 copy

Olfm4^fl/fl^S100a8^cre^+DMSO

Olfm4^fl/fl^S100a8^cre^+Fer-1

Occludin

**
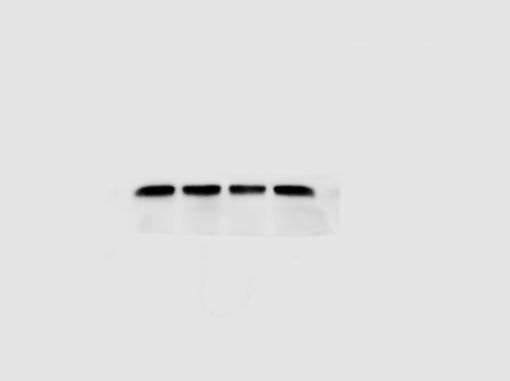
**

**
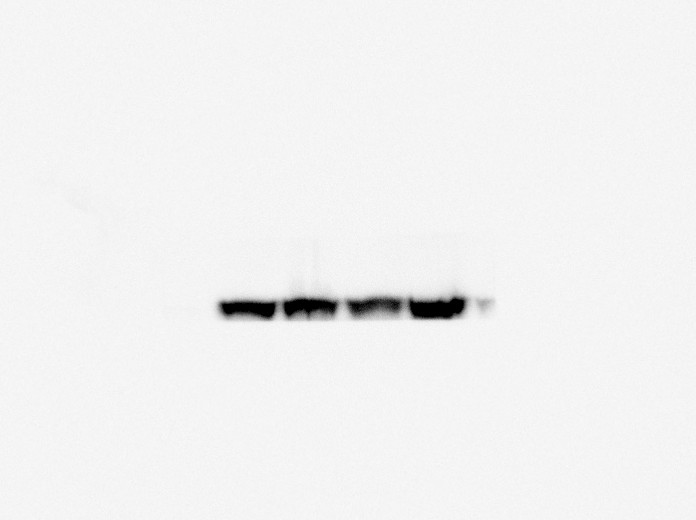
**

75 kDa

55 kDa

75 kDa

55 kDa

Zo-1

1 copy

Olfm4^fl/fl^S100a8^cre^+DMSO

Olfm4^fl/fl^S100a8^cre^+Fer-1

**
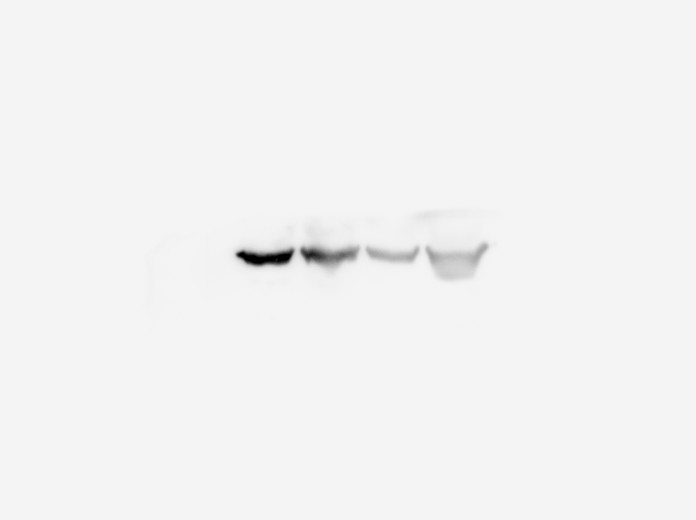
**

Olfm4^fl/fl^S100a8^cre^

+DMSO


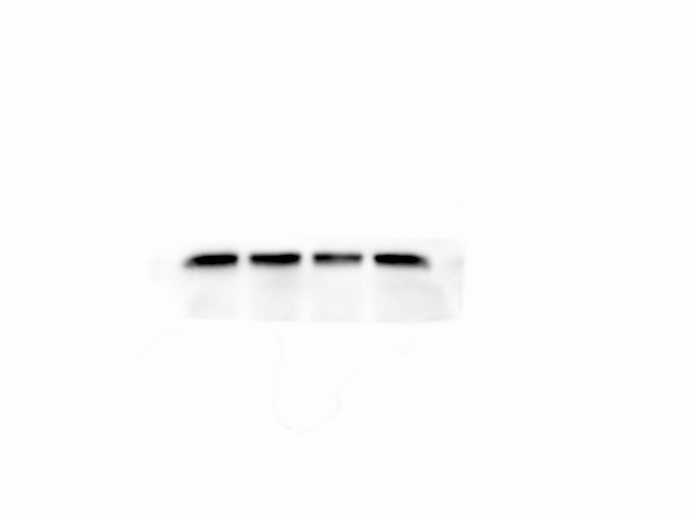

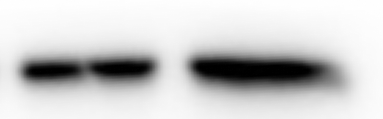


Zo-1

α-β-actin

Occludin

65

230

43


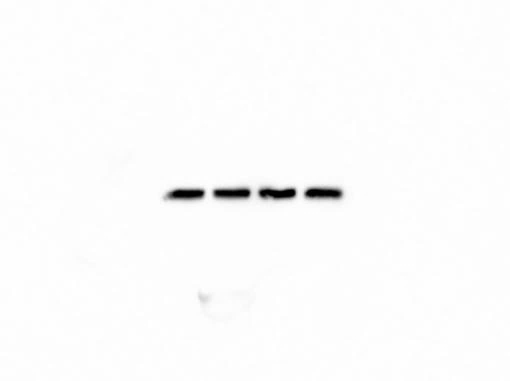


Olfm4^fl/fl^S100a8^cre^

+Fer-1

250 kDa

130 kDa

The representative blot


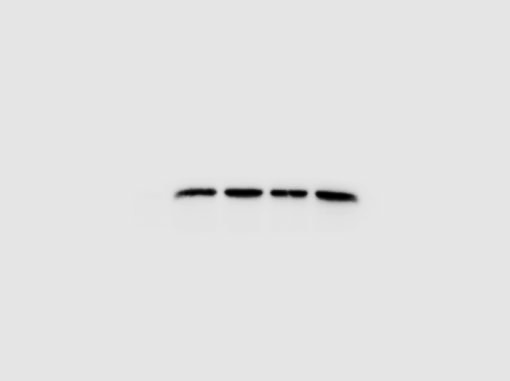


Zo-1

2 copies

Olfm4^fl/fl^S100a8^cre^+DMSO

Olfm4^fl/fl^S100a8^cre^+Fer-1

Olfm4^fl/fl^S100a8^cre^+DMSO

Olfm4^fl/fl^S100a8^cre^+Fer-1

α-β-actin

2 copies

Olfm4^fl/fl^S100a8^cre^+DMSO

Olfm4^fl/fl^S100a8^cre^+Fer-1

Olfm4^fl/fl^S100a8^cre^+DMSO

Olfm4^fl/fl^S100a8^cre^+Fer-1

250 kDa

130 kDa

1 copy

Olfm4^fl/fl^S100a8^cre^+DMSO

Olfm4^fl/fl^S100a8^cre^+Fer-1

**
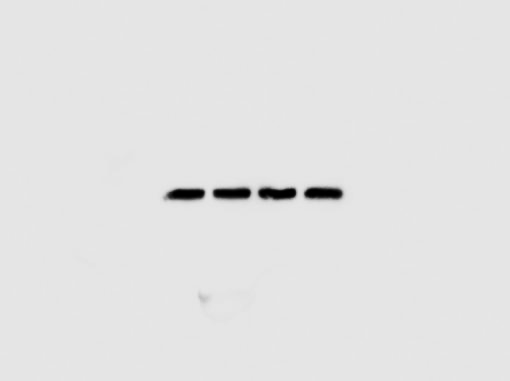
**

α-β-actin

**
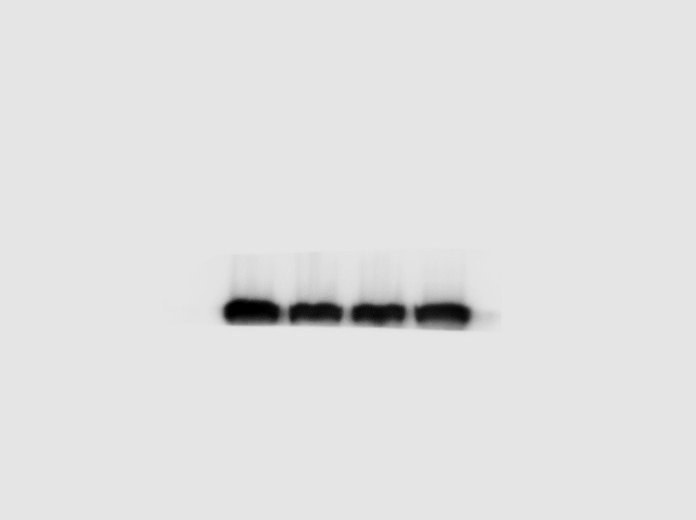
**

55 kDa

43 kDa

55 kDa

43 kDa

**Figure 4Z**

ATF4

55 kDa

43 kDa


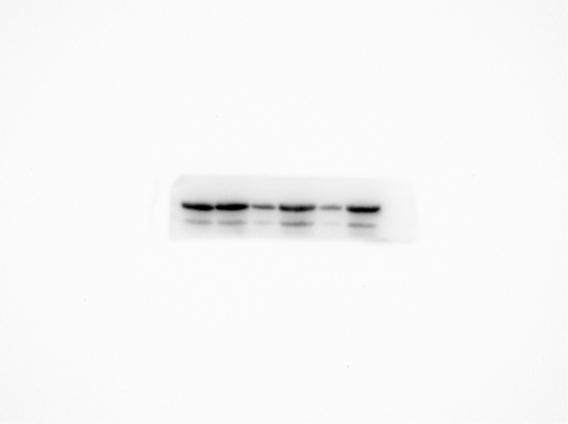


ATF4

1 copy

Olfm4^fl/fl^S100a8^cre^

+DMSO

Olfm4^fl/fl^S100a8^cre^

+Fer-1

1 copy

Olfm4^fl/fl^S100a8^cre^

+DMSO

Olfm4^fl/fl^S100a8^cre^

+Fer-1


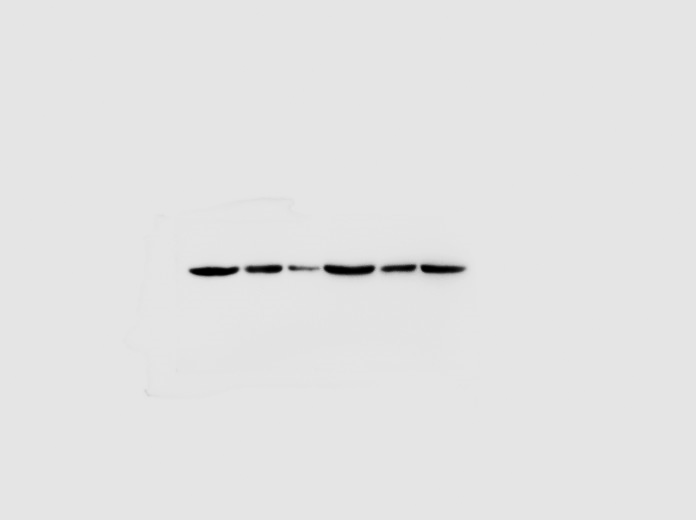


55 kDa

43 kDa

Tubulin

WCL

ATF4

Tubulin

Histone

C

N

WCL

C

N

49

55

17


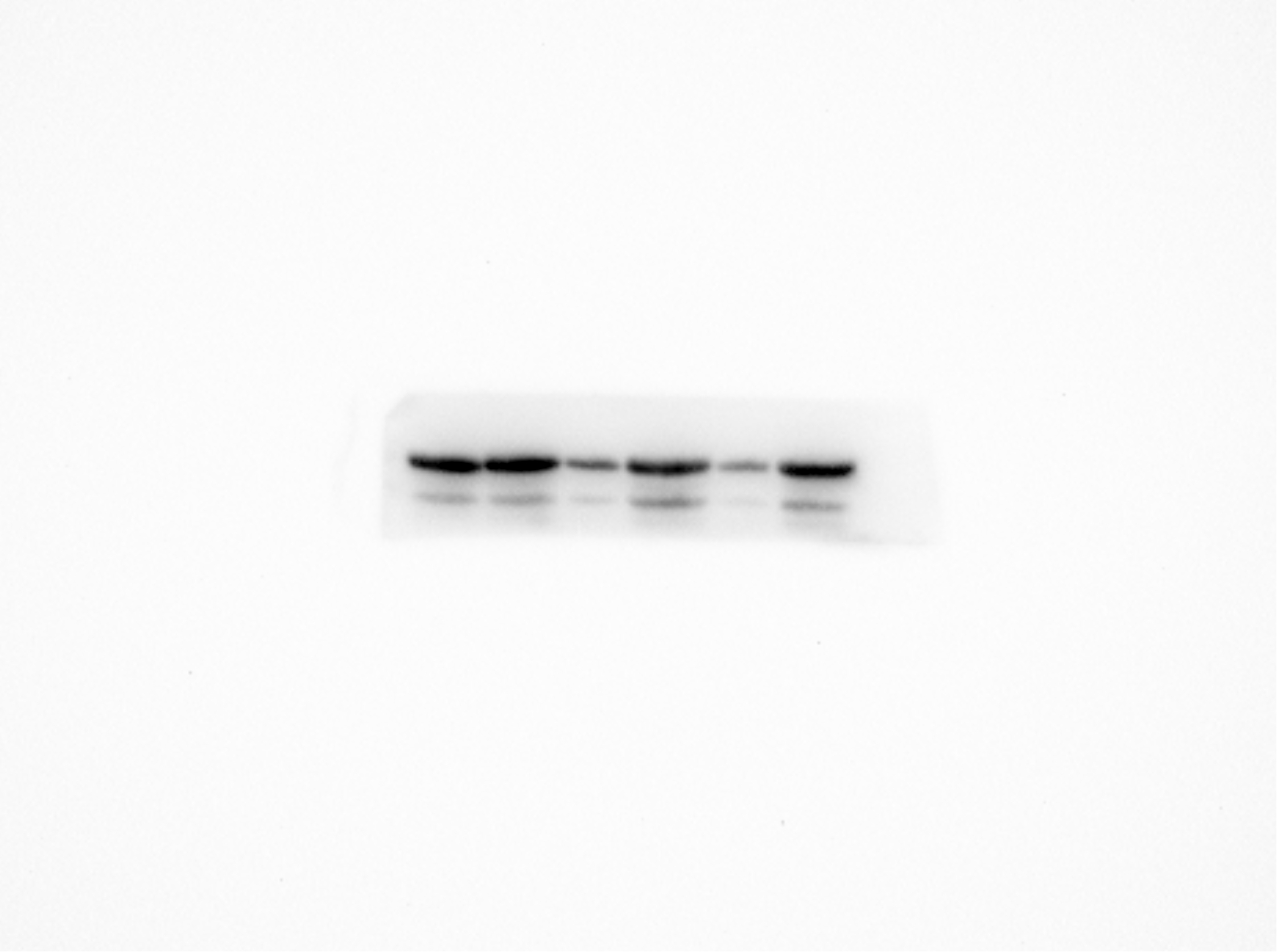

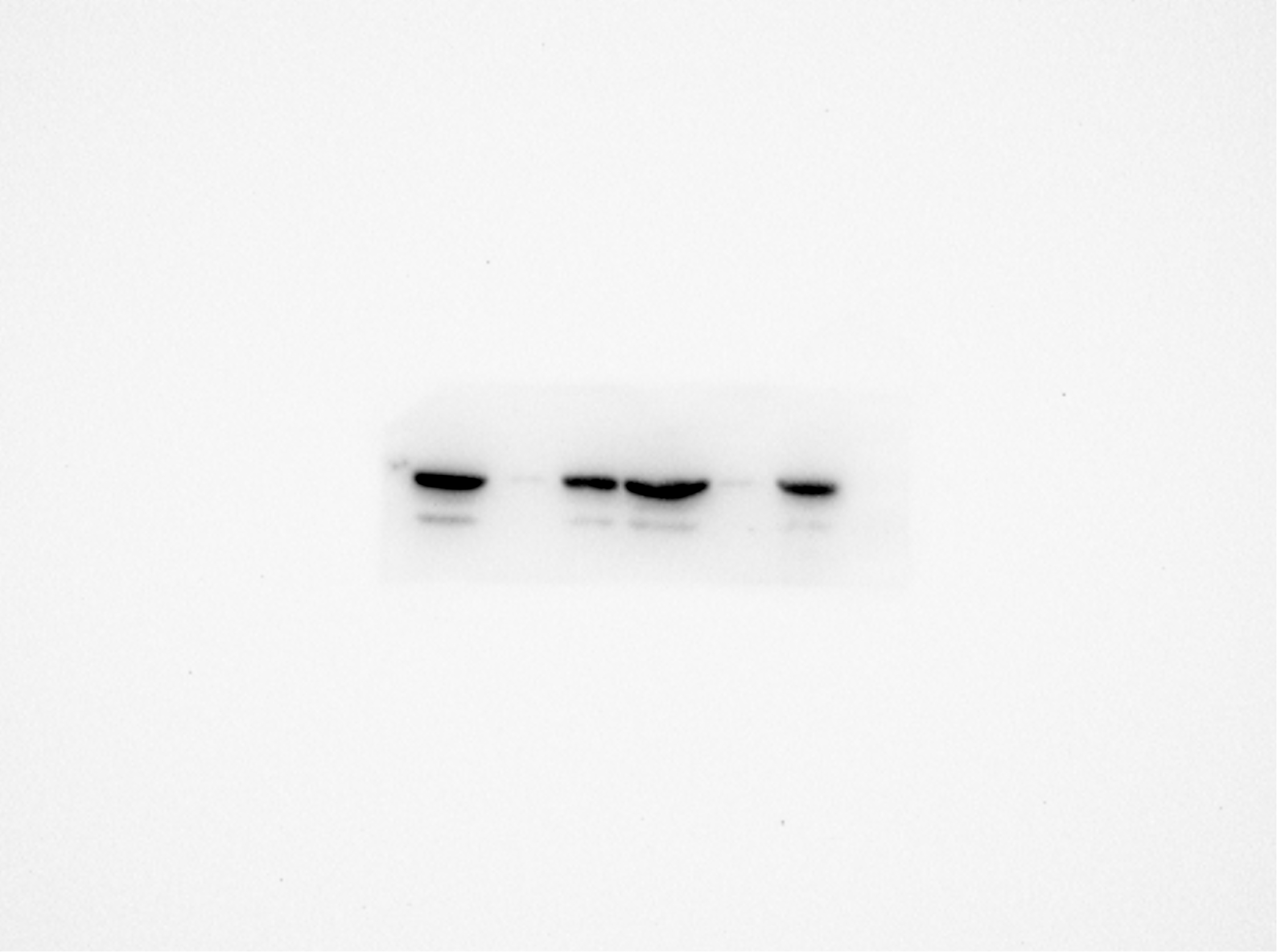

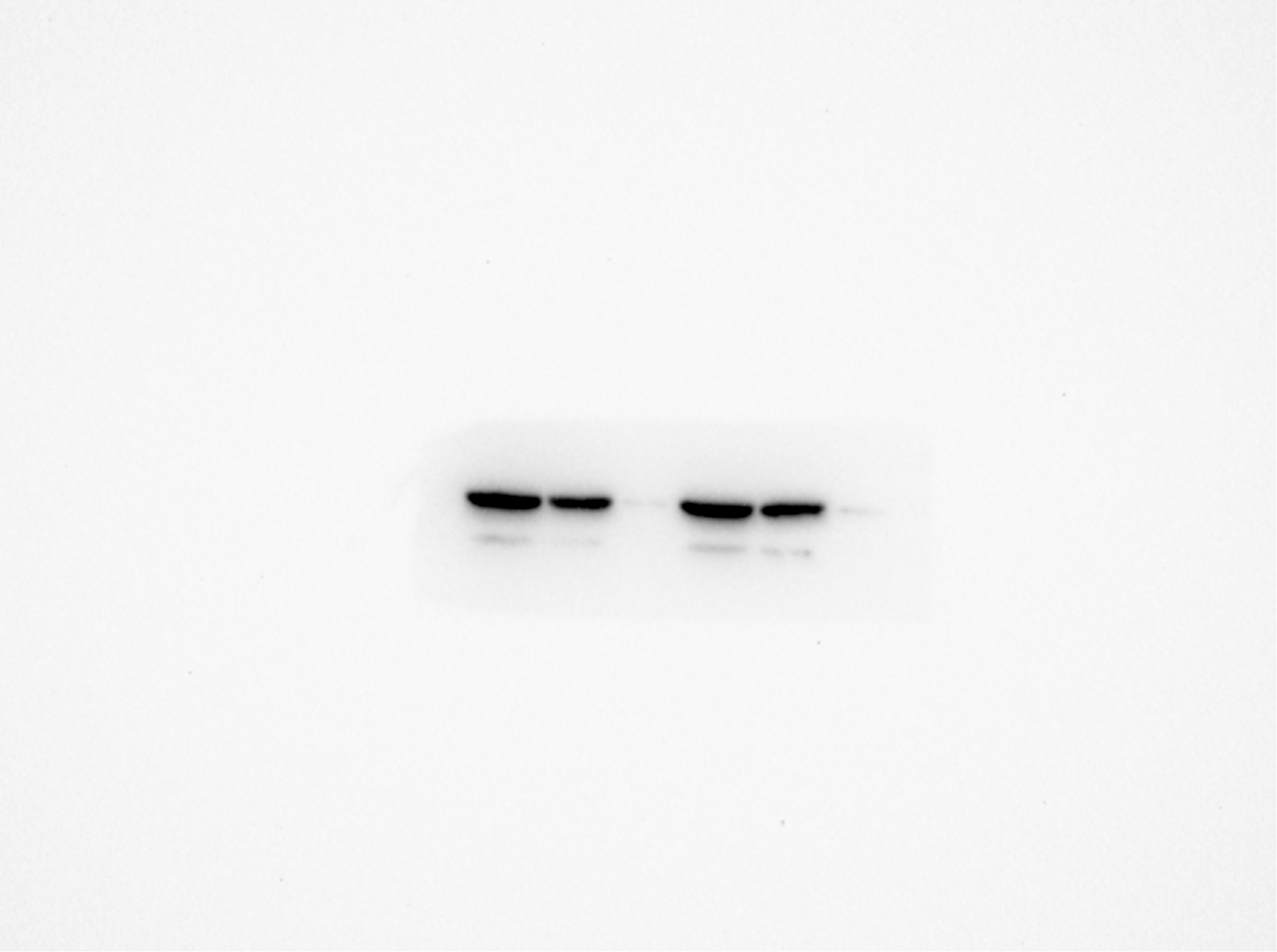


Olfm4^fl/fl^S100a8^cre^+Fer-1

Olfm4^fl/fl^S100a8^cre^+DMSO

1 copy

Olfm4^fl/fl^S100a8^cre^

+DMSO

Olfm4^fl/fl^S100a8^cre^

+Fer-1


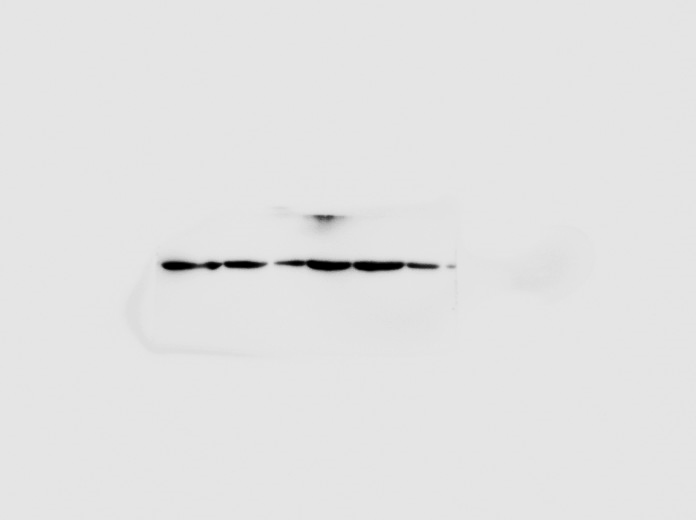


70 kDa

55 kDa

The representative blot

1 copy

Olfm4^fl/fl^S100a8^cre^

+DMSO

Olfm4^fl/fl^S100a8^cre^

+Fer-1


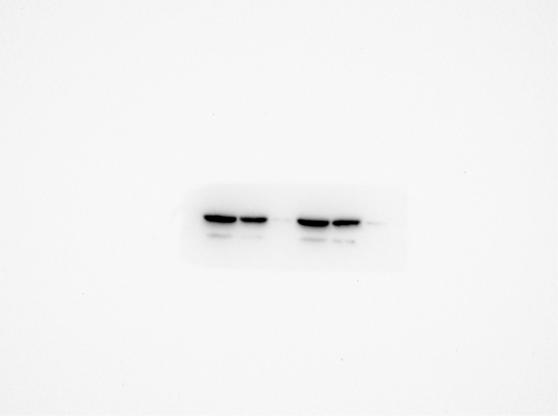


Tubulin

70 kDa

55 kDa


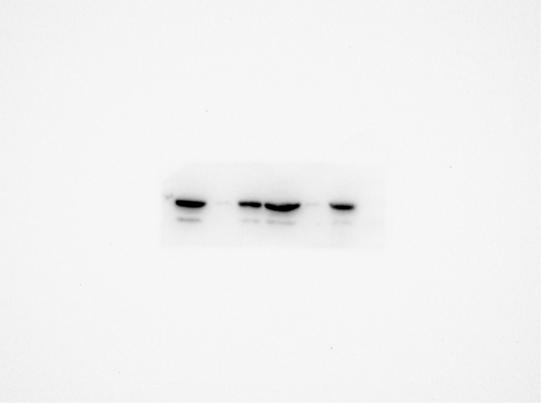


Histone


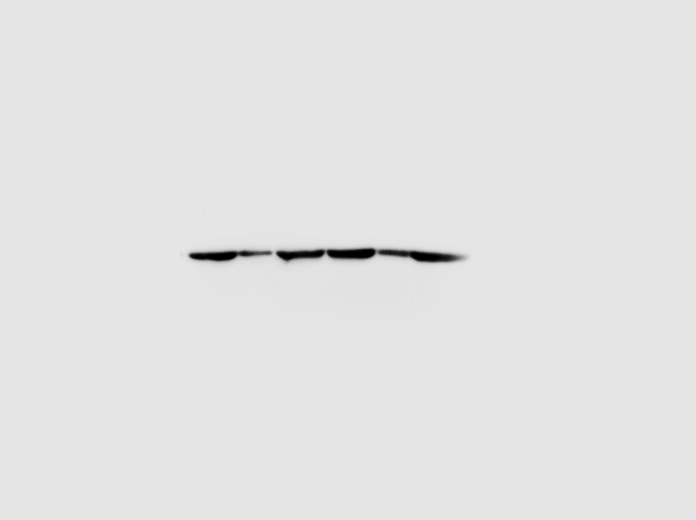


25 kDa

17 kDa

Histone

1 copy

Olfm4^fl/fl^S100a8^cre^

+DMSO

Olfm4^fl/fl^S100a8^cre^

+Fer-1

1 copy

Olfm4^fl/fl^S100a8^cre^

+DMSO

Olfm4^fl/fl^S100a8^cre^

+Fer-1

25 kDa

17 kDa

**Figure 5H**

Occludin

2 copies

Vehicle

ISRIB

Vehicle

ISRIB

ISRIB

Vehicle

1 copy

Occludin

**
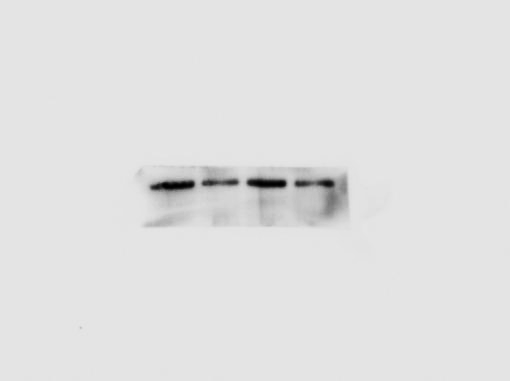

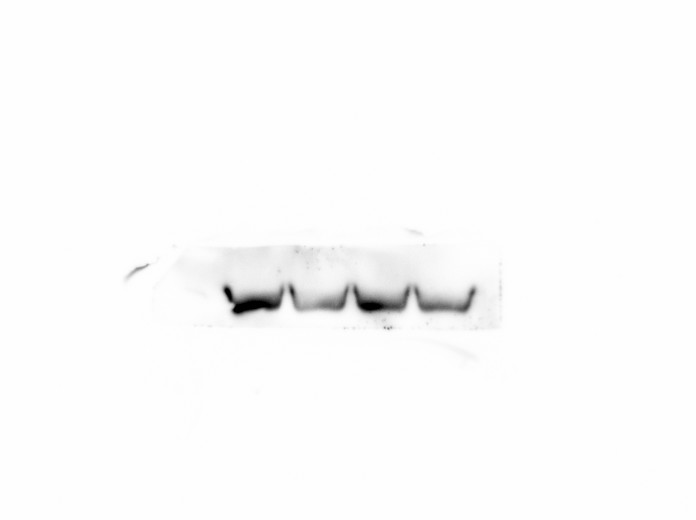
**

75 kDa

55 kDa

75 kDa

55 kDa

Zo-1

ISRIB

Vehicle

1 copy

**
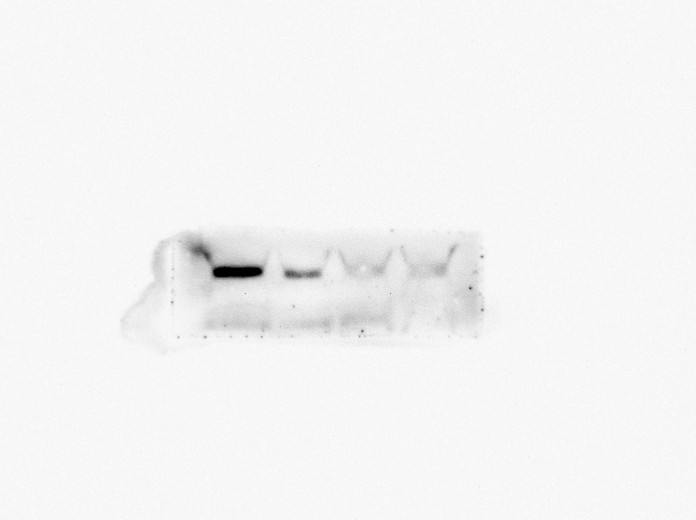
**

250 kDa

130 kDa

The representative blot


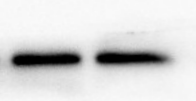

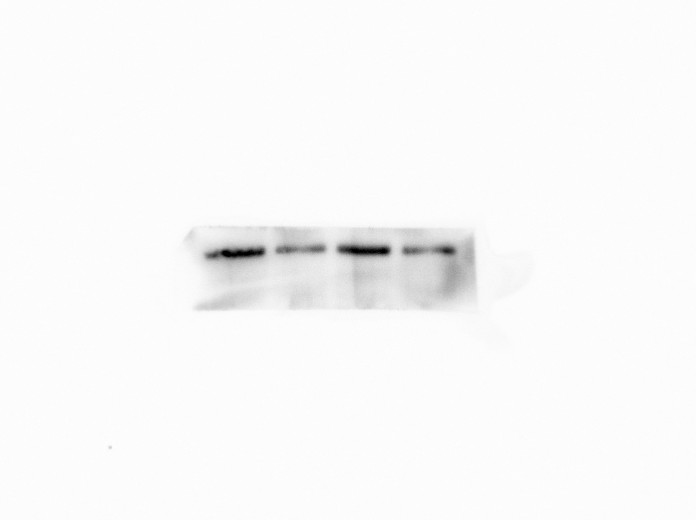

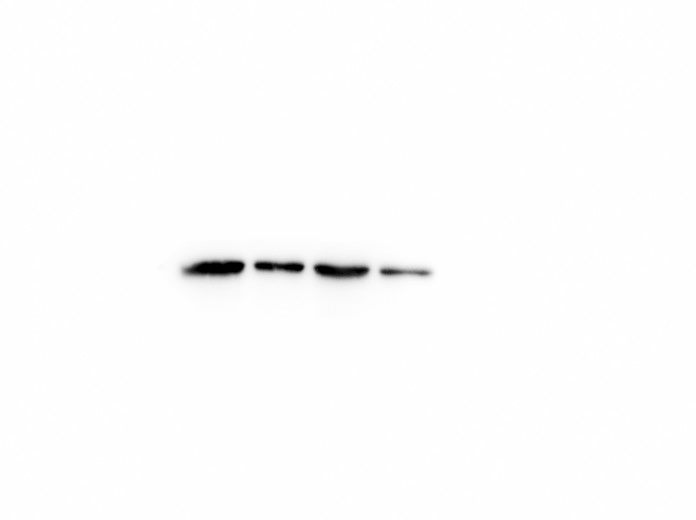


Zo-1

α-β-actin

Occludin

65

230

43

Vehicle

ISRIB

Zo-1

2 copies

Vehicle

ISRIB

Vehicle

ISRIB

**
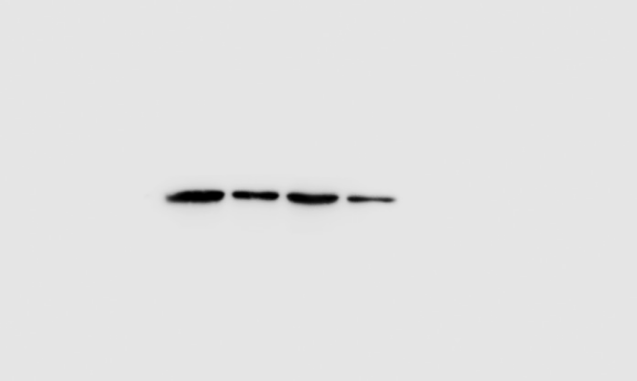
**

250 kDa

130 kDa

2 copies

Vehicle

ISRIB

Vehicle

ISRIB

α-β-actin


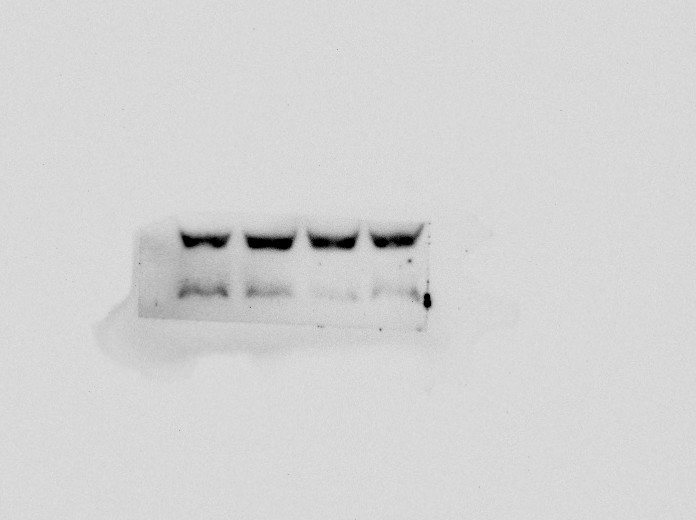


α-β-actin

55 kDa

43 kDa

ISRIB

Vehicle

1 copy

**
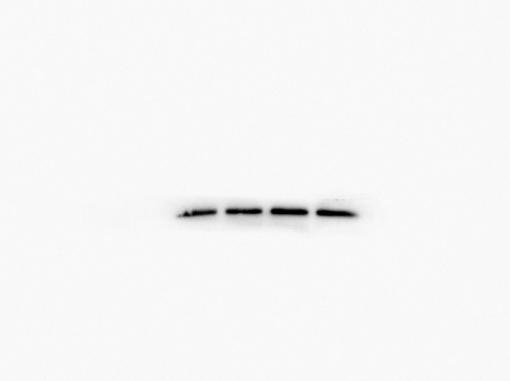
**

55 kDa

43 kDa

**Figure 5W**

3 copies

Gpx4^fl/fl^

Gpx4^fl/fl^S100a8^cre^

Gpx4^fl/fl^

Gpx4^fl/fl^S100a8^cre^

Gpx4^fl/fl^

Gpx4^fl/fl^S100a8^cre^

Occludin


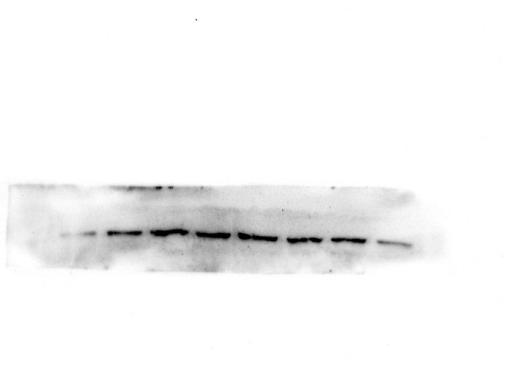


75 kDa

55 kDa

3 copies

Gpx4^fl/fl^

Gpx4^fl/fl^S100a8^cre^

Gpx4^fl/fl^

Gpx4^fl/fl^S100a8^cre^

Gpx4^fl/fl^

Gpx4^fl/fl^S100a8^cre^


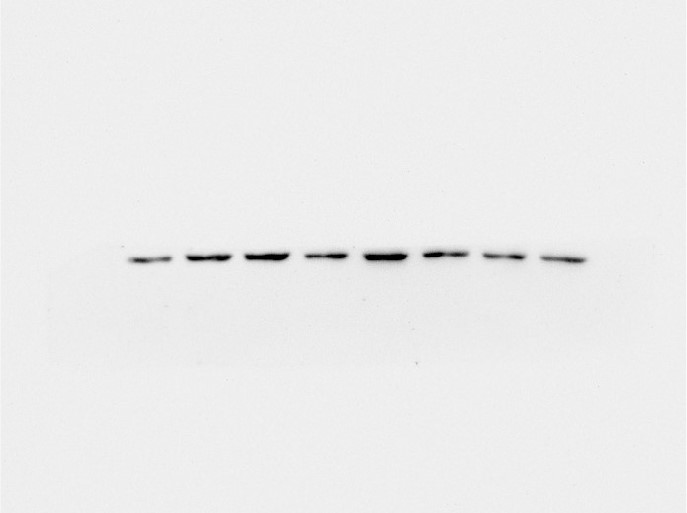


250 kDa

130 kDa

Zo-1

The representative blot

Zo-1

α-β-actin


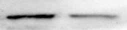


Occludin

65

230

43


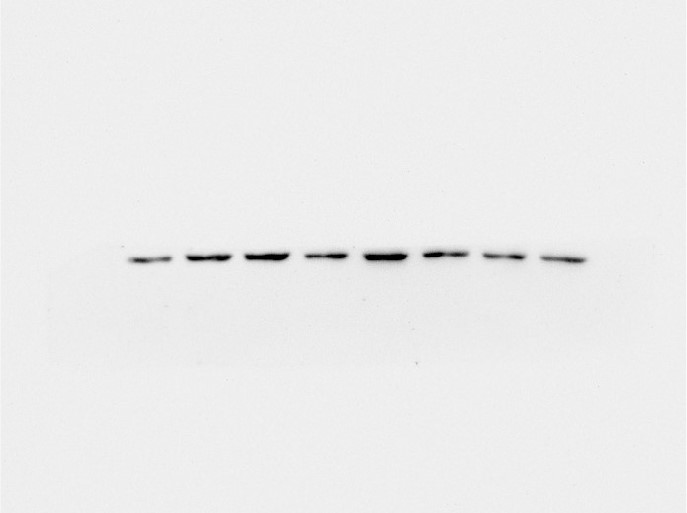

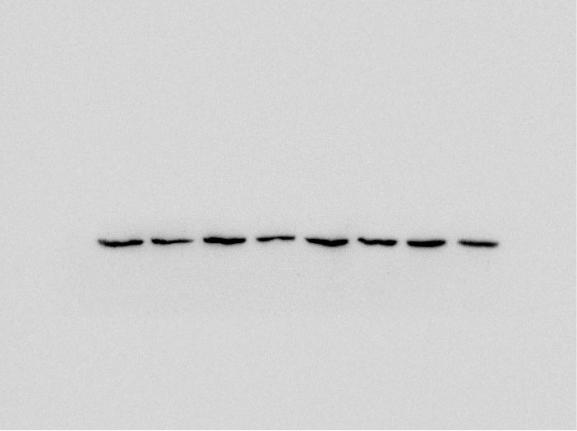


Gpx4^fl/fl^

Gpx4^fl/fl^S100a8^cre^

3 copies

Gpx4^fl/fl^

Gpx4^fl/fl^S100a8^cre^

Gpx4^fl/fl^

Gpx4^fl/fl^S100a8^cre^

Gpx4^fl/fl^

Gpx4^fl/fl^S100a8^cre^


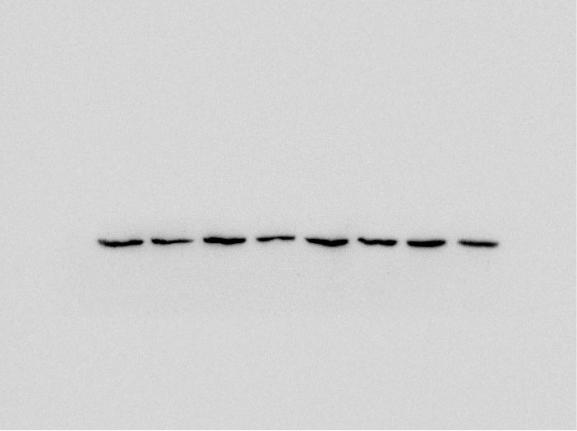


55 kDa

43 kDa

α-β-actin

**Figure 6F**


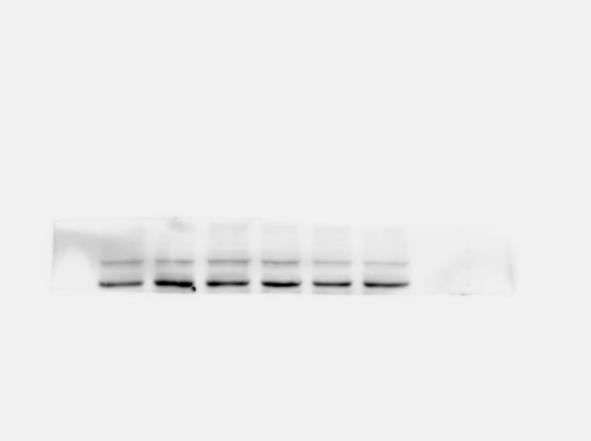


Occludin

75 kDa

55 kDa

3 copies

PBS

I3A

PBS

I3A

PBS

I3A


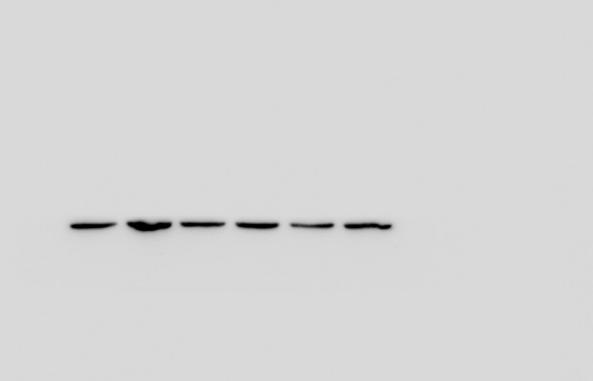


Zo-1

250 kDa

130 kDa

3 copies

PBS

I3A

PBS

I3A

PBS

I3A

The representative blot

Zo-1

α-β-actin

Occludin

65

230

43


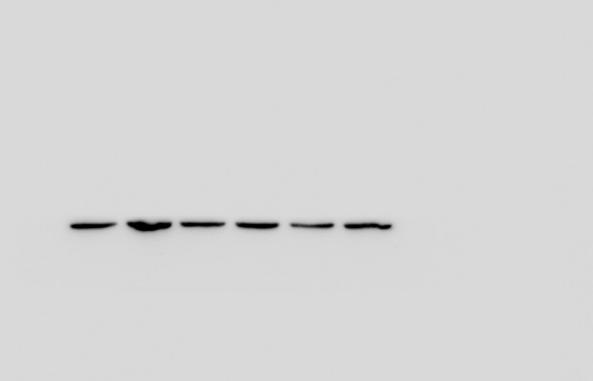

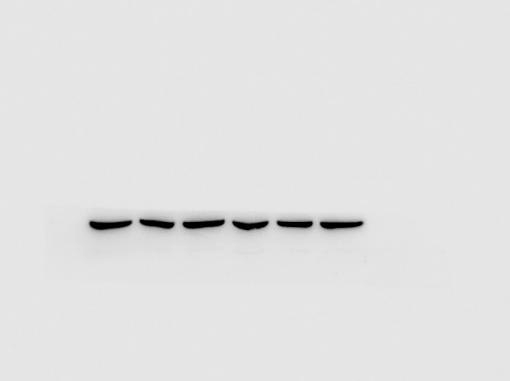

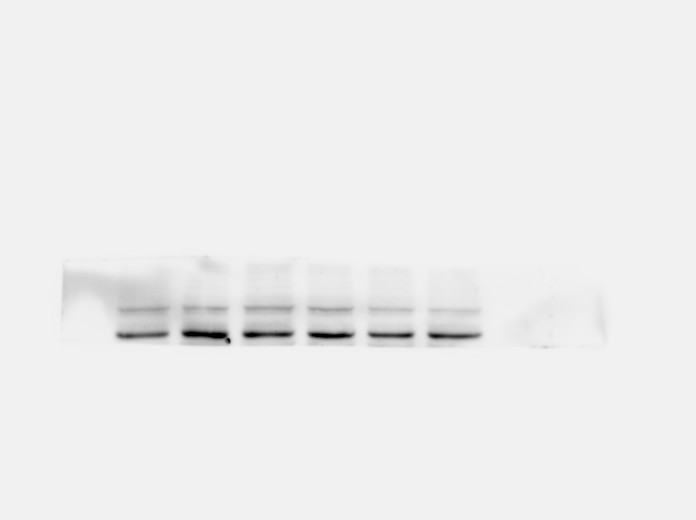


wild-type

+PBS

wild-type

+I3A


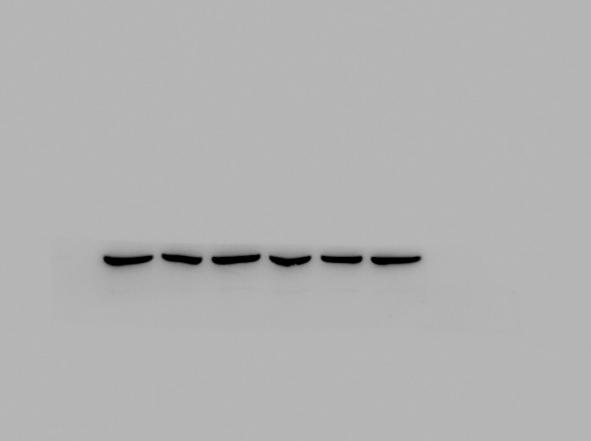


55 kDa

43 kDa

α-β-actin

3 copies

PBS

I3A

PBS

I3A

PBS

I3A

**Original qPCR Data**

This section presents the complete raw qPCR data corresponding to all qPCR-related panels in the manuscript, including Figure 1C, Figure 1D, Figure 1J, Figure 1O, Figure 1R, Figure 2G, Figure 2R, Figure 2S, Figure 2T, Figure 3G, Figure 3K, Figure 3L, Figure 3M, Figure 4G, Figure 4R, Figure 5G, Figure 5N, Figure 5V, Figure 6E, Figure 6N, Figure 6S, Figure 6Z, Figure 7F, Figure 7P, Figure 7Q, Figure 8J, Figure S4H, Figure S5F, Figure S5N, Figure S6G, Figure S6O, Figure S9F, Figure S12I, Figure S13G, Figure S13Q, Figure S17I, Figure S17K, Figure S17L, Figure S19I, Figure S20I, Figure S20J, and Figure S20K.

All original data tables on the following pages provide comprehensive experimental details, including technical replicate Ct values, mean Ct values, relative expression (RE), and normalized expression (NOR) values for each sample.

**Abbreviations:**

• Ct_1_, Ct_2_: Ct values of technical replicates 1 and 2

• Mean Ct: arithmetic mean of the two technical replicates (Ct_1_ and Ct_2_)

• RE: relative expression of the target gene, normalized to the reference gene

• NOR: ratio of the RE value of each sample to the minimum RE value across all experimental groups

**Figure 1C**

|  | **16S rDNA (reference gene)** | | | **L.reuteri (target gene)** | | |  |  |
| --- | --- | --- | --- | --- | --- | --- | --- | --- |
|  | **Ct_1_** | **Ct_2_** | **Mean Ct** | **Ct_1_** | **Ct_2_** | **Mean Ct** | **RE** | **NOR** |
| CON1 | 18.804 | 18.366 | 18.585 | 19.483 | 19.483 | 19.483 | 0.536630 | 47.062302 |
| CON2 | 18.609 | 18.287 | 18.448 | 19.684 | 19.684 | 19.684 | 0.424548 | 37.232743 |
| CON3 | 19.027 | 18.593 | 18.810 | 19.629 | 20.487 | 20.058 | 0.421031 | 36.924334 |
| CON4 | 18.283 | 18.283 | 18.283 | 20.389 | 20.541 | 20.465 | 0.220370 | 19.326386 |
| CON5 | 18.871 | 18.907 | 18.889 | 19.552 | 19.851 | 19.702 | 0.569394 | 49.935711 |
| CON6 | 17.604 | 17.795 | 17.700 | 19.218 | 19.218 | 19.218 | 0.349049 | 30.611461 |
| CON7 | 17.104 | 17.176 | 17.140 | 18.220 | 18.427 | 18.324 | 0.440282 | 38.612605 |
| CON8 | 17.365 | 17.453 | 17.409 | 18.725 | 18.172 | 18.449 | 0.486496 | 42.665558 |
| NEC1 | 18.640 | 18.510 | 18.575 | 22.392 | 22.392 | 22.392 | 0.070953 | 6.222524 |
| NEC2 | 17.374 | 17.042 | 17.208 | 21.357 | 21.412 | 21.385 | 0.055303 | 4.850051 |
| NEC3 | 17.155 | 16.672 | 16.914 | 20.362 | 20.362 | 20.362 | 0.091601 | 8.033340 |
| NEC4 | 16.223 | 16.275 | 16.249 | 20.673 | 20.647 | 20.660 | 0.047006 | 4.122444 |
| NEC5 | 14.822 | 14.428 | 14.625 | 17.737 | 18.394 | 18.066 | 0.092110 | 8.078010 |
| NEC6 | 16.991 | 16.900 | 16.946 | 23.400 | 23.400 | 23.400 | 0.011403 | 1.000000 |
| NEC7 | 20.570 | 20.498 | 20.534 | 23.640 | 23.671 | 23.656 | 0.114904 | 10.077040 |
| NEC8 | 21.905 | 21.905 | 21.905 | 24.451 | 24.451 | 24.451 | 0.171229 | 15.016743 |

**Figure 1D**

|  | **16S rDNA (reference gene)** | | | **L.rhamnosus (target gene)** | | |  |  |
| --- | --- | --- | --- | --- | --- | --- | --- | --- |
|  | **Ct_1_** | **Ct_2_** | **Mean Ct** | **Ct_1_** | **Ct_2_** | **Mean Ct** | **RE** | **NOR** |
| CON1 | 18.804 | 18.366 | 18.585 | 18.845 | 18.386 | 18.616 | 0.979081 | 35.126794 |
| CON2 | 18.609 | 18.287 | 18.448 | 19.375 | 19.194 | 19.285 | 0.560000 | 20.091314 |
| CON3 | 19.027 | 18.593 | 18.810 | 19.770 | 18.959 | 19.365 | 0.680893 | 24.428612 |
| CON4 | 18.283 | 18.283 | 18.283 | 19.731 | 19.731 | 19.731 | 0.366529 | 13.150083 |
| CON5 | 18.871 | 18.907 | 18.889 | 19.171 | 19.120 | 19.146 | 0.837116 | 30.033485 |
| CON6 | 17.604 | 17.795 | 17.700 | 18.370 | 18.454 | 18.412 | 0.610262 | 21.894552 |
| CON7 | 17.104 | 17.176 | 17.140 | 17.690 | 17.736 | 17.713 | 0.672217 | 24.117359 |
| CON8 | 17.365 | 17.453 | 17.409 | 17.628 | 17.714 | 17.671 | 0.833931 | 29.919206 |
| NEC1 | 20.570 | 20.498 | 20.534 | 23.940 | 23.605 | 23.773 | 0.105953 | 3.801319 |
| NEC2 | 18.640 | 18.510 | 18.575 | 21.828 | 21.889 | 21.859 | 0.102699 | 3.684580 |
| NEC3 | 17.374 | 17.042 | 17.208 | 21.128 | 21.128 | 21.128 | 0.066064 | 2.370186 |
| NEC4 | 16.991 | 16.900 | 16.946 | 22.209 | 22.012 | 22.111 | 0.027873 | 1.000000 |
| NEC5 | 16.223 | 16.275 | 16.249 | 20.134 | 19.966 | 20.050 | 0.071744 | 2.573978 |
| NEC6 | 14.822 | 14.428 | 14.625 | 18.010 | 18.200 | 18.105 | 0.089622 | 3.215404 |
| NEC7 | 20.460 | 20.509 | 20.485 | 22.700 | 23.578 | 23.139 | 0.158824 | 5.698175 |
| NEC8 | 21.905 | 21.905 | 21.905 | 24.496 | 24.494 | 24.495 | 0.166086 | 5.958710 |

**Figure 1J**

|  | **β-actin (reference gene)** | | | **Occludin (target gene)** | | |  |  |
| --- | --- | --- | --- | --- | --- | --- | --- | --- |
|  | **Ct_1_** | **Ct_2_** | **Mean Ct** | **Ct_1_** | **Ct_2_** | **Mean Ct** | **RE** | **NOR** |
| PBS1 | 22.761 | 22.762 | 22.762 | 34.762 | 33.377 | 34.069 | 0.000394 | 1.000000 |
| PBS2 | 19.368 | 19.672 | 19.520 | 30.159 | 30.159 | 30.159 | 0.000627 | 1.590138 |
| PBS3 | 22.186 | 22.595 | 22.391 | 32.738 | 32.145 | 32.442 | 0.000943 | 2.389328 |
| PBS4 | 19.669 | 19.647 | 19.658 | 29.708 | 30.168 | 29.938 | 0.000804 | 2.039294 |
| Probiotics1 | 22.184 | 22.904 | 22.544 | 29.149 | 28.929 | 29.039 | 0.011086 | 28.102550 |
| Probiotics2 | 21.299 | 21.739 | 21.519 | 28.225 | 28.029 | 28.127 | 0.010251 | 25.985940 |
| Probiotics3 | 20.896 | 20.289 | 20.593 | 27.071 | 27.433 | 27.252 | 0.009894 | 25.081580 |
| Probiotics4 | 19.706 | 19.384 | 19.545 | 26.273 | 25.931 | 26.102 | 0.010622 | 26.925731 |

|  | **β-actin (reference gene)** | | | **Cldn2 (target gene)** | | |  |  |
| --- | --- | --- | --- | --- | --- | --- | --- | --- |
|  | **Ct_1_** | **Ct_2_** | **Mean Ct** | **Ct_1_** | **Ct_2_** | **Mean Ct** | **RE** | **NOR** |
| PBS1 | 22.761 | 22.762 | 22.762 | 25.147 | 26.974 | 26.060 | 0.101645 | 1.160648 |
| PBS2 | 19.368 | 19.672 | 19.520 | 23.380 | 22.405 | 22.892 | 0.096572 | 1.102721 |
| PBS3 | 22.186 | 22.595 | 22.391 | 25.670 | 24.899 | 25.285 | 0.134508 | 1.535907 |
| PBS4 | 19.669 | 19.647 | 19.658 | 23.278 | 23.065 | 23.171 | 0.087576 | 1.000000 |
| Probiotics1 | 22.184 | 22.904 | 22.544 | 24.004 | 23.634 | 23.819 | 0.413082 | 4.716859 |
| Probiotics2 | 21.299 | 21.739 | 21.519 | 23.696 | 23.723 | 23.710 | 0.219049 | 2.501256 |
| Probiotics3 | 20.896 | 20.289 | 20.593 | 21.536 | 21.944 | 21.740 | 0.451508 | 5.155631 |
| Probiotics4 | 19.706 | 19.384 | 19.545 | 21.905 | 21.928 | 21.916 | 0.193243 | 2.206583 |

|  | **β-actin (reference gene)** | | | **Cldn3 (target gene)** | | |  |  |
| --- | --- | --- | --- | --- | --- | --- | --- | --- |
|  | **Ct_1_** | **Ct_2_** | **Mean Ct** | **Ct_1_** | **Ct_2_** | **Mean Ct** | **RE** | **NOR** |
| PBS1 | 22.761 | 22.762 | 22.762 | 25.943 | 25.320 | 25.632 | 0.136790 | 1.701905 |
| PBS2 | 19.368 | 19.672 | 19.520 | 22.733 | 23.581 | 23.157 | 0.080375 | 1.000000 |
| PBS3 | 22.186 | 22.595 | 22.391 | 25.207 | 25.954 | 25.581 | 0.109577 | 1.363322 |
| PBS4 | 19.669 | 19.647 | 19.658 | 22.492 | 22.838 | 22.665 | 0.124377 | 1.547456 |
| Probiotics1 | 22.184 | 22.904 | 22.544 | 23.256 | 22.912 | 23.084 | 0.687723 | 8.556444 |
| Probiotics2 | 21.299 | 21.739 | 21.519 | 22.158 | 22.068 | 22.113 | 0.662549 | 8.243237 |
| Probiotics3 | 20.896 | 20.289 | 20.593 | 23.022 | 23.338 | 23.180 | 0.166431 | 4.070682 |
| Probiotics4 | 19.706 | 19.384 | 19.545 | 21.820 | 21.200 | 21.510 | 0.256158 | 3.187043 |

|  | **β-actin (reference gene)** | | | **Cldn4 (target gene)** | | |  |  |
| --- | --- | --- | --- | --- | --- | --- | --- | --- |
|  | **Ct_1_** | **Ct_2_** | **Mean Ct** | **Ct_1_** | **Ct_2_** | **Mean Ct** | **RE** | **NOR** |
| PBS1 | 22.761 | 22.762 | 22.762 | 30.187 | 30.670 | 30.428 | 0.004922 | 1.016603 |
| PBS2 | 19.368 | 19.672 | 19.520 | 26.725 | 27.072 | 26.898 | 0.006010 | 1.241528 |
| PBS3 | 22.186 | 22.595 | 22.391 | 29.232 | 29.289 | 29.260 | 0.008551 | 1.766229 |
| PBS4 | 19.669 | 19.647 | 19.658 | 27.471 | 27.226 | 27.348 | 0.004841 | 1.000000 |
| Probiotics1 | 22.184 | 22.904 | 22.544 | 26.415 | 26.685 | 26.550 | 0.062230 | 12.854267 |
| Probiotics2 | 21.299 | 21.739 | 21.519 | 25.763 | 25.855 | 25.809 | 0.051123 | 10.560046 |
| Probiotics3 | 20.896 | 20.289 | 20.593 | 25.416 | 24.702 | 25.059 | 0.045243 | 9.345444 |
| Probiotics4 | 19.706 | 19.384 | 19.545 | 24.224 | 23.310 | 23.767 | 0.053574 | 11.066296 |

|  | **β-actin (reference gene)** | | | **Zo-1 (target gene)** | | |  |  |
| --- | --- | --- | --- | --- | --- | --- | --- | --- |
|  | **Ct_1_** | **Ct_2_** | **Mean Ct** | **Ct_1_** | **Ct_2_** | **Mean Ct** | **RE** | **NOR** |
| PBS1 | 22.761 | 22.762 | 22.762 | 26.558 | 26.887 | 26.722 | 0.064234 | 1.956566 |
| PBS2 | 19.368 | 19.672 | 19.520 | 23.379 | 22.724 | 23.051 | 0.086500 | 2.634786 |
| PBS3 | 22.186 | 22.595 | 22.391 | 25.648 | 26.341 | 25.995 | 0.082223 | 2.504496 |
| PBS4 | 19.669 | 19.647 | 19.658 | 24.511 | 24.663 | 24.587 | 0.032830 | 1.000000 |
| Probiotics1 | 22.184 | 22.904 | 22.544 | 23.480 | 24.030 | 23.755 | 0.431985 | 13.158187 |
| Probiotics2 | 21.299 | 21.739 | 21.519 | 22.716 | 23.105 | 22.911 | 0.381092 | 11.607999 |
| Probiotics3 | 20.896 | 20.289 | 20.593 | 22.569 | 22.534 | 22.551 | 0.257314 | 7.837736 |
| Probiotics4 | 19.706 | 19.384 | 19.545 | 22.669 | 19.110 | 20.890 | 0.393678 | 11.991368 |

**Figure 1O**

| **IECs** | | | | | | | | |
| --- | --- | --- | --- | --- | --- | --- | --- | --- |
|  | **β-actin (reference gene)** | | | **Il18 (target gene)** | | |  |  |
|  | **Ct_1_** | **Ct_2_** | **Mean Ct** | **Ct_1_** | **Ct_2_** | **Mean Ct** | **RE** | **NOR** |
| PBS1 | 24.162 | 24.447 | 24.305 | 28.387 | 28.239 | 28.313 | 0.062125 | 1.148316 |
| PBS2 | 23.751 | 23.631 | 23.691 | 27.097 | 27.991 | 27.544 | 0.069168 | 1.278497 |
| PBS3 | 23.203 | 23.265 | 23.234 | 27.674 | 27.018 | 27.346 | 0.057821 | 1.000000 |
| PBS4 | 24.102 | 24.056 | 24.079 | 28.039 | 27.493 | 27.766 | 0.077633 | 1.434955 |
| Probiotics1 | 21.391 | 21.376 | 21.384 | 25.335 | 25.318 | 25.326 | 0.065035 | 1.202102 |
| Probiotics2 | 22.147 | 21.918 | 22.032 | 25.214 | 25.903 | 25.559 | 0.086781 | 1.604044 |
| Probiotics3 | 22.741 | 22.754 | 22.747 | 26.424 | 26.394 | 26.409 | 0.079003 | 1.460279 |
| Probiotics4 | 23.080 | 22.653 | 22.866 | 25.976 | 26.496 | 26.236 | 0.096740 | 1.788126 |

| **iPMN-MDSCs** | | | | | | | | |
| --- | --- | --- | --- | --- | --- | --- | --- | --- |
|  | **β-actin (reference gene)** | | | **Il18 (target gene)** | | |  |  |
|  | **Ct_1_** | **Ct_2_** | **Mean Ct** | **Ct_1_** | **Ct_2_** | **Mean Ct** | **RE** | **NOR** |
| PBS1 | 22.396 | 22.459 | 22.427 | 26.338 | 26.933 | 26.635 | 0.054101 | 1.000000 |
| PBS2 | 23.225 | 23.043 | 23.134 | 26.275 | 26.912 | 26.594 | 0.090884 | 1.679890 |
| PBS3 | 21.842 | 21.870 | 21.856 | 25.940 | 25.396 | 25.668 | 0.071214 | 1.316306 |
| PBS4 | 22.383 | 22.918 | 22.650 | 26.376 | 27.180 | 26.778 | 0.057202 | 1.057311 |
| Probiotics1 | 19.853 | 20.163 | 20.008 | 21.983 | 22.186 | 22.084 | 0.237135 | 4.383177 |
| Probiotics2 | 21.857 | 21.063 | 21.460 | 23.047 | 23.447 | 23.247 | 0.289791 | 5.356450 |
| Probiotics3 | 21.300 | 21.244 | 21.272 | 23.417 | 23.717 | 23.567 | 0.203754 | 3.766155 |
| Probiotics4 | 22.356 | 22.356 | 22.356 | 24.587 | 24.096 | 24.341 | 0.252534 | 4.667810 |

**Figure 1R**

|  | **β-actin (reference gene)** | | | **Olfm4 (target gene)** | | |  |  |
| --- | --- | --- | --- | --- | --- | --- | --- | --- |
|  | **Ct_1_** | **Ct_2_** | **Mean Ct** | **Ct_1_** | **Ct_2_** | **Mean Ct** | **RE** | **NOR** |
| PBS1 | 19.818 | 19.710 | 19.764 | 24.381 | 23.953 | 24.167 | 0.047271 | 1.956539 |
| PBS2 | 17.869 | 17.605 | 17.737 | 21.389 | 21.212 | 21.300 | 0.084593 | 3.501259 |
| PBS3 | 19.672 | 19.914 | 19.793 | 25.339 | 24.989 | 25.164 | 0.024161 | 1.000000 |
| PBS4 | 23.080 | 22.903 | 22.991 | 27.939 | 27.431 | 27.685 | 0.038635 | 1.599088 |
| Probiotics1 | 18.925 | 18.194 | 18.559 | 20.660 | 21.071 | 20.865 | 0.202226 | 8.370039 |
| Probiotics2 | 18.971 | 19.190 | 19.080 | 21.204 | 21.932 | 21.568 | 0.178319 | 7.380529 |
| Probiotics3 | 21.685 | 21.321 | 21.503 | 23.500 | 23.286 | 23.393 | 0.269770 | 11.165649 |
| Probiotics4 | 18.590 | 18.173 | 18.382 | 20.590 | 20.620 | 20.605 | 0.214149 | 8.863537 |

|  | **β-actin (reference gene)** | | | **Itgb6 (target gene)** | | |  |  |
| --- | --- | --- | --- | --- | --- | --- | --- | --- |
|  | **Ct_1_** | **Ct_2_** | **Mean Ct** | **Ct_1_** | **Ct_2_** | **Mean Ct** | **RE** | **NOR** |
| PBS1 | 22.396 | 22.459 | 22.427 | 26.022 | 26.798 | 26.410 | 0.063260 | 1.423262 |
| PBS2 | 23.225 | 23.043 | 23.134 | 26.976 | 26.360 | 26.668 | 0.086336 | 1.424457 |
| PBS3 | 21.842 | 21.870 | 21.856 | 26.202 | 26.494 | 26.348 | 0.044447 | 1.000000 |
| PBS4 | 22.383 | 22.918 | 22.650 | 26.435 | 25.602 | 26.019 | 0.096834 | 2.178648 |
| Probiotics1 | 19.853 | 20.163 | 20.008 | 23.008 | 23.595 | 23.301 | 0.102028 | 2.295499 |
| Probiotics2 | 21.857 | 21.063 | 21.460 | 25.841 | 24.192 | 25.017 | 0.084984 | 1.912030 |
| Probiotics3 | 21.300 | 21.244 | 21.272 | 23.600 | 24.479 | 24.040 | 0.146828 | 3.303452 |
| Probiotics4 | 22.356 | 22.356 | 22.356 | 25.142 | 25.792 | 25.467 | 0.115766 | 2.604577 |

|  | **β-actin (reference gene)** | | | **Vcl (target gene)** | | |  |  |
| --- | --- | --- | --- | --- | --- | --- | --- | --- |
|  | **Ct_1_** | **Ct_2_** | **Mean Ct** | **Ct_1_** | **Ct_2_** | **Mean Ct** | **RE** | **NOR** |
| PBS1 | 22.396 | 22.459 | 22.427 | 25.946 | 25.503 | 25.725 | 0.101696 | 1.108418 |
| PBS2 | 23.225 | 23.043 | 23.134 | 26.531 | 26.629 | 26.580 | 0.091749 | 1.000000 |
| PBS3 | 21.842 | 21.870 | 21.856 | 23.522 | 23.296 | 23.409 | 0.340885 | 3.715422 |
| PBS4 | 22.383 | 22.918 | 22.650 | 24.143 | 23.776 | 23.960 | 0.403514 | 4.398035 |
| Probiotics1 | 19.853 | 20.163 | 20.008 | 21.856 | 20.884 | 21.370 | 0.389117 | 4.241116 |
| Probiotics2 | 21.857 | 21.063 | 21.460 | 21.499 | 23.694 | 22.596 | 0.454979 | 4.958976 |
| Probiotics3 | 21.300 | 21.244 | 21.272 | 21.289 | 21.243 | 21.266 | 1.004460 | 10.947959 |
| Probiotics4 | 22.356 | 22.356 | 22.356 | 22.469 | 22.705 | 22.587 | 0.851892 | 9.285068 |

|  | **β-actin (reference gene)** | | | **Fermt1 (target gene)** | | |  |  |
| --- | --- | --- | --- | --- | --- | --- | --- | --- |
|  | **Ct_1_** | **Ct_2_** | **Mean Ct** | **Ct_1_** | **Ct_2_** | **Mean Ct** | **RE** | **NOR** |
| PBS1 | 22.396 | 22.459 | 22.427 | 27.345 | 27.543 | 27.444 | 0.030886 | 1.000000 |
| PBS2 | 23.225 | 23.043 | 23.134 | 27.006 | 27.564 | 27.285 | 0.056293 | 1.822571 |
| PBS3 | 21.842 | 21.870 | 21.856 | 25.606 | 25.636 | 25.621 | 0.073573 | 2.382060 |
| PBS4 | 22.383 | 22.918 | 22.650 | 26.117 | 25.972 | 26.044 | 0.095127 | 3.079899 |
| Probiotics1 | 19.853 | 20.163 | 20.008 | 23.681 | 22.748 | 23.215 | 0.108337 | 3.507602 |
| Probiotics2 | 21.857 | 21.063 | 21.460 | 24.829 | 24.537 | 24.683 | 0.107088 | 3.467154 |
| Probiotics3 | 21.300 | 21.244 | 21.272 | 22.332 | 26.735 | 24.534 | 0.104277 | 3.376152 |
| Probiotics4 | 22.356 | 22.356 | 22.356 | 24.631 | 25.636 | 25.134 | 0.145837 | 4.721703 |

**Figure 2G**

|  | **β-actin (reference gene)** | | | **Occludin (target gene)** | | |  |  |
| --- | --- | --- | --- | --- | --- | --- | --- | --- |
|  | **Ct_1_** | **Ct_2_** | **Mean Ct** | **Ct_1_** | **Ct_2_** | **Mean Ct** | **RE** | **NOR** |
| FLOX1 | 20.916 | 20.673 | 20.795 | 30.685 | 30.613 | 30.649 | 0.001080 | 5.352492 |
| FLOX2 | 20.993 | 20.984 | 20.989 | 31.211 | 31.503 | 31.357 | 0.000756 | 3.748028 |
| FLOX3 | 23.426 | 22.912 | 23.169 | 32.805 | 32.654 | 32.730 | 0.001324 | 6.561022 |
| FLOX4 | 22.136 | 21.845 | 21.991 | 31.491 | 31.941 | 31.716 | 0.001181 | 5.854234 |
| CKO1 | 21.165 | 19.795 | 20.480 | 32.713 | 32.157 | 32.435 | 0.000252 | 1.248306 |
| CKO2 | 18.814 | 18.906 | 18.860 | 31.472 | 30.796 | 31.134 | 0.000202 | 1.000000 |
| CKO3 | 18.065 | 18.229 | 18.147 | 30.136 | 30.296 | 30.216 | 0.000233 | 1.153361 |
| CKO4 | 20.854 | 20.588 | 20.721 | 31.764 | 31.596 | 31.680 | 0.000502 | 2.490041 |

|  | **β-actin (reference gene)** | | | **Cldn2 (target gene)** | | |  |  |
| --- | --- | --- | --- | --- | --- | --- | --- | --- |
|  | **Ct_1_** | **Ct_2_** | **Mean Ct** | **Ct_1_** | **Ct_2_** | **Mean Ct** | **RE** | **NOR** |
| FLOX1 | 20.916 | 20.673 | 20.795 | 23.444 | 24.221 | 23.833 | 0.121735 | 4.019293 |
| FLOX2 | 20.993 | 20.984 | 20.989 | 23.989 | 23.184 | 23.586 | 0.165203 | 5.454478 |
| FLOX3 | 23.426 | 22.912 | 23.169 | 25.711 | 25.492 | 25.602 | 0.185229 | 6.115666 |
| FLOX4 | 22.136 | 21.845 | 21.991 | 24.949 | 24.351 | 24.650 | 0.158273 | 5.225680 |
| CKO1 | 21.165 | 19.795 | 20.480 | 24.434 | 24.197 | 24.316 | 0.070052 | 2.312907 |
| CKO2 | 18.814 | 18.906 | 18.860 | 23.975 | 23.835 | 23.905 | 0.030288 | 1.000000 |
| CKO3 | 18.065 | 18.229 | 18.147 | 22.350 | 22.501 | 22.425 | 0.051533 | 1.701463 |
| CKO4 | 20.854 | 20.588 | 20.721 | 24.744 | 24.670 | 24.707 | 0.063116 | 2.083875 |

|  | **β-actin (reference gene)** | | | **Cldn3 (target gene)** | | |  |  |
| --- | --- | --- | --- | --- | --- | --- | --- | --- |
|  | **Ct_1_** | **Ct_2_** | **Mean Ct** | **Ct_1_** | **Ct_2_** | **Mean Ct** | **RE** | **NOR** |
| FLOX1 | 20.916 | 20.673 | 20.795 | 21.839 | 21.832 | 21.836 | 0.485936 | 12.562839 |
| FLOX2 | 20.993 | 20.984 | 20.989 | 23.636 | 23.133 | 23.385 | 0.189983 | 4.911603 |
| FLOX3 | 23.426 | 22.912 | 23.169 | 24.254 | 25.077 | 24.665 | 0.354439 | 9.163265 |
| FLOX4 | 22.136 | 21.845 | 21.991 | 23.899 | 23.978 | 23.939 | 0.259201 | 6.701101 |
| CKO1 | 21.165 | 19.795 | 20.480 | 23.971 | 23.844 | 23.907 | 0.092955 | 2.403158 |
| CKO2 | 18.814 | 18.906 | 18.860 | 22.463 | 22.870 | 22.666 | 0.071457 | 1.847366 |
| CKO3 | 18.065 | 18.229 | 18.147 | 22.894 | 22.785 | 22.839 | 0.038680 | 1.000000 |
| CKO4 | 20.854 | 20.588 | 20.721 | 24.746 | 24.971 | 24.858 | 0.056831 | 1.469243 |

|  | **β-actin (reference gene)** | | | **Cldn4 (target gene)** | | |  |  |
| --- | --- | --- | --- | --- | --- | --- | --- | --- |
|  | **Ct_1_** | **Ct_2_** | **Mean Ct** | **Ct_1_** | **Ct_2_** | **Mean Ct** | **RE** | **NOR** |
| FLOX1 | 20.916 | 20.673 | 20.795 | 26.651 | 26.607 | 26.629 | 0.017524 | 3.408875 |
| FLOX2 | 20.993 | 20.984 | 20.989 | 24.907 | 25.377 | 25.142 | 0.056196 | 10.931362 |
| FLOX3 | 23.426 | 22.912 | 23.169 | 27.984 | 27.878 | 27.931 | 0.036855 | 7.168996 |
| FLOX4 | 22.136 | 21.845 | 21.991 | 26.909 | 26.365 | 26.637 | 0.039924 | 7.765968 |
| CKO1 | 21.165 | 19.795 | 20.480 | 26.961 | 26.574 | 26.768 | 0.012802 | 2.490259 |
| CKO2 | 18.814 | 18.906 | 18.860 | 26.346 | 25.072 | 25.709 | 0.008672 | 1.686976 |
| CKO3 | 18.065 | 18.229 | 18.147 | 24.625 | 26.876 | 25.751 | 0.005141 | 1.000000 |
| CKO4 | 20.854 | 20.588 | 20.721 | 27.963 | 27.613 | 27.788 | 0.007458 | 1.450811 |

|  | **β-actin (reference gene)** | | | **Zo-1 (target gene)** | | |  |  |
| --- | --- | --- | --- | --- | --- | --- | --- | --- |
|  | **Ct_1_** | **Ct_2_** | **Mean Ct** | **Ct_1_** | **Ct_2_** | **Mean Ct** | **RE** | **NOR** |
| FLOX1 | 20.916 | 20.673 | 20.795 | 25.307 | 25.310 | 25.309 | 0.043763 | 6.414891 |
| FLOX2 | 20.993 | 20.984 | 20.989 | 25.201 | 25.101 | 25.151 | 0.055831 | 8.183945 |
| FLOX3 | 23.426 | 22.912 | 23.169 | 27.156 | 27.812 | 27.484 | 0.050239 | 7.364260 |
| FLOX4 | 22.136 | 21.845 | 21.991 | 25.621 | 25.995 | 25.808 | 0.070945 | 10.399295 |
| CKO1 | 21.165 | 19.795 | 20.480 | 25.687 | 25.142 | 25.414 | 0.032708 | 4.794444 |
| CKO2 | 18.814 | 18.906 | 18.860 | 24.172 | 24.093 | 24.133 | 0.025861 | 3.790779 |
| CKO3 | 18.065 | 18.229 | 18.147 | 24.030 | 25.046 | 24.538 | 0.011919 | 1.747172 |
| CKO4 | 20.854 | 20.588 | 20.721 | 27.774 | 28.060 | 27.917 | 0.006822 | 1.000000 |

**Figure 2R**

|  | **16S rDNA (reference gene)** | | | **L.reuteri (target gene)** | | |  |  |
| --- | --- | --- | --- | --- | --- | --- | --- | --- |
|  | **Ct_1_** | **Ct_2_** | **Mean Ct** | **Ct_1_** | **Ct_2_** | **Mean Ct** | **RE** | **NOR** |
| FLOX1 | 22.354 | 22.898 | 22.626 | 25.571 | 24.803 | 25.187 | 0.169458 | 2.808890 |
| FLOX2 | 23.995 | 22.873 | 23.434 | 25.913 | 25.994 | 25.954 | 0.174403 | 2.890863 |
| FLOX3 | 22.393 | 22.405 | 22.399 | 25.504 | 25.342 | 25.423 | 0.122938 | 2.037782 |
| FLOX4 | 22.091 | 21.969 | 22.030 | 25.073 | 25.056 | 25.065 | 0.122046 | 2.023005 |
| CKO1 | 22.862 | 23.056 | 22.959 | 26.559 | 26.954 | 26.757 | 0.071918 | 1.192096 |
| CKO2 | 23.288 | 24.041 | 23.665 | 27.017 | 27.639 | 27.328 | 0.078918 | 1.308125 |
| CKO3 | 23.036 | 23.225 | 23.131 | 26.700 | 26.882 | 26.791 | 0.079082 | 1.310848 |
| CKO4 | 20.909 | 20.696 | 20.803 | 24.992 | 24.715 | 24.854 | 0.060329 | 1.000000 |

**Figure 2S**

|  | **16S rDNA (reference gene)** | | | **L.rhamnosus (target gene)** | | |  |  |
| --- | --- | --- | --- | --- | --- | --- | --- | --- |
|  | **Ct_1_** | **Ct_2_** | **Mean Ct** | **Ct_1_** | **Ct_2_** | **Mean Ct** | **RE** | **NOR** |
| FLOX1 | 22.354 | 22.898 | 22.626 | 25.184 | 25.176 | 25.180 | 0.170282 | 2.672148 |
| FLOX2 | 23.995 | 22.873 | 23.434 | 25.761 | 25.888 | 25.825 | 0.190716 | 2.992809 |
| FLOX3 | 22.393 | 22.405 | 22.399 | 25.165 | 25.309 | 25.237 | 0.139855 | 2.194664 |
| FLOX4 | 22.091 | 21.969 | 22.030 | 24.615 | 24.482 | 24.549 | 0.174524 | 2.738717 |
| CKO1 | 22.862 | 23.056 | 22.959 | 26.774 | 26.749 | 26.762 | 0.071669 | 1.124669 |
| CKO2 | 23.288 | 24.041 | 23.665 | 27.435 | 27.509 | 27.472 | 0.071421 | 1.120778 |
| CKO3 | 23.036 | 23.225 | 23.131 | 27.019 | 26.911 | 26.965 | 0.070097 | 1.099997 |
| CKO4 | 20.909 | 20.696 | 20.803 | 24.937 | 24.612 | 24.775 | 0.063725 | 1.000000 |

**Figure 2T**

|  | **16S rDNA (reference gene)** | | | **Morganella (target gene)** | | |  |  |
| --- | --- | --- | --- | --- | --- | --- | --- | --- |
|  | **Ct_1_** | **Ct_2_** | **Mean Ct** | **Ct_1_** | **Ct_2_** | **Mean Ct** | **RE** | **NOR** |
| FLOX1 | 23.199 | 23.900 | 23.549 | 28.623 | 28.468 | 28.546 | 0.031325 | 1.287639 |
| FLOX2 | 23.490 | 22.959 | 23.224 | 28.335 | 28.836 | 28.586 | 0.024327 | 1.000000 |
| FLOX3 | 22.814 | 22.157 | 22.486 | 26.295 | 27.641 | 26.968 | 0.044741 | 1.839125 |
| FLOX4 | 23.003 | 22.809 | 22.906 | 26.717 | 26.987 | 26.852 | 0.064912 | 2.668302 |
| CKO1 | 24.564 | 24.275 | 24.419 | 27.995 | 27.155 | 27.575 | 0.112213 | 4.612657 |
| CKO2 | 23.719 | 24.361 | 24.040 | 26.107 | 26.247 | 26.177 | 0.227335 | 9.344900 |
| CKO3 | 23.330 | 23.633 | 23.481 | 25.721 | 25.548 | 25.634 | 0.224825 | 9.241714 |
| CKO4 | 22.745 | 22.448 | 22.597 | 23.950 | 24.281 | 24.116 | 0.349002 | 14.346146 |

**Figure 3G**

|  | **β-actin (reference gene)** | | | **Occludin (target gene)** | | |  |  |
| --- | --- | --- | --- | --- | --- | --- | --- | --- |
|  | **Ct_1_** | **Ct_2_** | **Mean Ct** | **Ct_1_** | **Ct_2_** | **Mean Ct** | **RE** | **NOR** |
| PBS1 | 18.903 | 18.782 | 18.843 | 27.230 | 28.103 | 27.667 | 0.002207 | 5.828550 |
| PBS2 | 17.874 | 17.924 | 17.899 | 25.895 | 25.747 | 25.821 | 0.004122 | 10.887901 |
| PBS3 | 17.958 | 18.655 | 18.307 | 26.948 | 26.775 | 26.862 | 0.002658 | 7.021542 |
| PBS4 | 18.332 | 18.492 | 18.412 | 26.845 | 26.920 | 26.883 | 0.002818 | 7.444278 |
| Probiotics1 | 23.335 | 23.715 | 23.525 | 30.692 | 31.039 | 30.866 | 0.006170 | 16.297398 |
| Probiotics2 | 19.608 | 19.527 | 19.568 | 26.864 | 27.717 | 27.291 | 0.004733 | 12.501246 |
| Probiotics3 | 19.813 | 19.570 | 19.692 | 26.813 | 26.858 | 26.836 | 0.007070 | 18.674530 |
| Probiotics4 | 21.145 | 21.145 | 21.145 | 28.782 | 28.794 | 28.788 | 0.005003 | 13.214043 |
| ABX1 | 18.820 | 18.840 | 18.830 | 29.030 | 29.182 | 29.106 | 0.000807 | 2.130216 |
| ABX2 | 19.933 | 18.480 | 19.207 | 31.040 | 30.107 | 30.574 | 0.000379 | 1.000000 |
| ABX3 | 18.483 | 18.613 | 18.548 | 28.795 | 29.137 | 28.966 | 0.000731 | 1.930534 |
| ABX4 | 20.348 | 20.803 | 20.576 | 31.039 | 31.039 | 31.039 | 0.000708 | 1.870599 |
| ABX+Pro1 | 19.249 | 19.526 | 19.388 | 28.241 | 28.241 | 28.241 | 0.002162 | 5.710037 |
| ABX+Pro2 | 19.510 | 19.519 | 19.515 | 28.959 | 28.959 | 28.959 | 0.001435 | 3.790794 |
| ABX+Pro3 | 19.512 | 19.458 | 19.485 | 28.740 | 28.574 | 28.657 | 0.001734 | 4.578897 |
| ABX+Pro4 | 21.893 | 21.506 | 21.700 | 30.610 | 30.610 | 30.610 | 0.002078 | 5.488835 |

|  | **β-actin (reference gene)** | | | **Cldn2 (target gene)** | | |  |  |
| --- | --- | --- | --- | --- | --- | --- | --- | --- |
|  | **Ct_1_** | **Ct_2_** | **Mean Ct** | **Ct_1_** | **Ct_2_** | **Mean Ct** | **RE** | **NOR** |
| PBS1 | 18.903 | 18.782 | 18.843 | 24.852 | 24.226 | 24.539 | 0.019293 | 3.236060 |
| PBS2 | 17.874 | 17.924 | 17.899 | 23.431 | 23.431 | 23.431 | 0.021611 | 3.624809 |
| PBS3 | 17.958 | 18.655 | 18.307 | 22.861 | 22.861 | 22.861 | 0.042558 | 7.138333 |
| PBS4 | 18.332 | 18.492 | 18.412 | 23.697 | 22.911 | 23.304 | 0.033677 | 5.648703 |
| Probiotics1 | 23.335 | 23.715 | 23.525 | 26.942 | 27.353 | 27.148 | 0.081205 | 13.620489 |
| Probiotics2 | 19.608 | 19.527 | 19.568 | 23.453 | 23.921 | 23.687 | 0.057532 | 9.649806 |
| Probiotics3 | 19.813 | 19.570 | 19.692 | 23.682 | 23.682 | 23.682 | 0.062913 | 10.552405 |
| Probiotics4 | 21.145 | 21.145 | 21.145 | 25.041 | 25.041 | 25.041 | 0.067172 | 11.266754 |
| ABX1 | 18.820 | 18.840 | 18.830 | 26.468 | 25.972 | 26.220 | 0.005962 | 1.000000 |
| ABX2 | 19.933 | 18.480 | 19.207 | 25.167 | 25.551 | 25.359 | 0.014058 | 2.357896 |
| ABX3 | 18.483 | 18.613 | 18.548 | 25.656 | 25.707 | 25.682 | 0.007122 | 1.194577 |
| ABX4 | 20.348 | 20.803 | 20.576 | 26.656 | 26.707 | 26.681 | 0.014522 | 2.435700 |
| ABX+Pro1 | 19.249 | 19.526 | 19.388 | 24.603 | 23.892 | 24.248 | 0.034435 | 5.775717 |
| ABX+Pro2 | 19.510 | 19.519 | 19.515 | 24.114 | 24.114 | 24.114 | 0.041249 | 6.918695 |
| ABX+Pro3 | 19.512 | 19.458 | 19.485 | 24.466 | 24.466 | 24.466 | 0.031664 | 5.311061 |
| ABX+Pro4 | 21.893 | 21.506 | 21.700 | 26.586 | 25.858 | 26.222 | 0.043510 | 7.297994 |

|  | **β-actin (reference gene)** | | | **Cldn3 (target gene)** | | |  |  |
| --- | --- | --- | --- | --- | --- | --- | --- | --- |
|  | **Ct_1_** | **Ct_2_** | **Mean Ct** | **Ct_1_** | **Ct_2_** | **Mean Ct** | **RE** | **NOR** |
| PBS1 | 18.903 | 18.782 | 18.843 | 21.213 | 21.315 | 21.264 | 0.186704 | 6.016077 |
| PBS2 | 17.874 | 17.924 | 17.899 | 20.725 | 20.725 | 20.725 | 0.141013 | 4.543817 |
| PBS3 | 17.958 | 18.655 | 18.307 | 20.167 | 20.167 | 20.167 | 0.275397 | 8.874016 |
| PBS4 | 18.332 | 18.492 | 18.412 | 21.113 | 20.978 | 21.045 | 0.161150 | 5.192679 |
| Probiotics1 | 23.335 | 23.715 | 23.525 | 24.415 | 24.129 | 24.272 | 0.595840 | 19.199508 |
| Probiotics2 | 19.608 | 19.527 | 19.568 | 20.163 | 20.957 | 20.560 | 0.502606 | 16.195263 |
| Probiotics3 | 19.813 | 19.570 | 19.692 | 21.366 | 21.295 | 21.331 | 0.321079 | 10.345991 |
| Probiotics4 | 21.145 | 21.145 | 21.145 | 22.003 | 21.241 | 21.622 | 0.718470 | 23.150958 |
| ABX1 | 18.820 | 18.840 | 18.830 | 23.840 | 23.840 | 23.840 | 0.031034 | 1.000000 |
| ABX2 | 19.933 | 18.480 | 19.207 | 22.345 | 22.345 | 22.345 | 0.113558 | 3.659128 |
| ABX3 | 18.483 | 18.613 | 18.548 | 21.813 | 21.846 | 21.830 | 0.102842 | 3.313831 |
| ABX4 | 20.348 | 20.803 | 20.576 | 24.113 | 23.978 | 24.046 | 0.090246 | 2.907945 |
| ABX+Pro1 | 19.249 | 19.526 | 19.388 | 20.461 | 21.266 | 20.864 | 0.359484 | 11.583505 |
| ABX+Pro2 | 19.510 | 19.519 | 19.515 | 21.606 | 21.384 | 21.495 | 0.253402 | 8.165267 |
| ABX+Pro3 | 19.512 | 19.458 | 19.485 | 21.695 | 21.845 | 21.770 | 0.205185 | 6.611603 |
| ABX+Pro4 | 21.893 | 21.506 | 21.700 | 23.823 | 23.823 | 23.823 | 0.229489 | 7.394743 |

|  | **β-actin (reference gene)** | | | **Cldn4 (target gene)** | | |  |  |
| --- | --- | --- | --- | --- | --- | --- | --- | --- |
|  | **Ct_1_** | **Ct_2_** | **Mean Ct** | **Ct_1_** | **Ct_2_** | **Mean Ct** | **RE** | **NOR** |
| PBS1 | 18.903 | 18.782 | 18.843 | 26.618 | 27.173 | 26.896 | 0.003766 | 3.599566 |
| PBS2 | 17.874 | 17.924 | 17.899 | 24.488 | 24.764 | 24.626 | 0.009439 | 9.021064 |
| PBS3 | 17.958 | 18.655 | 18.307 | 24.637 | 25.219 | 24.928 | 0.010157 | 9.707415 |
| PBS4 | 18.332 | 18.492 | 18.412 | 26.560 | 26.828 | 26.694 | 0.003213 | 3.070541 |
| Probiotics1 | 23.335 | 23.715 | 23.525 | 28.941 | 29.130 | 29.036 | 0.021940 | 20.969297 |
| Probiotics2 | 19.608 | 19.527 | 19.568 | 25.574 | 24.788 | 25.181 | 0.020425 | 19.521608 |
| Probiotics3 | 19.813 | 19.570 | 19.692 | 26.538 | 26.330 | 26.434 | 0.009339 | 8.925915 |
| Probiotics4 | 21.145 | 21.145 | 21.145 | 26.758 | 26.871 | 26.815 | 0.019648 | 18.778371 |
| ABX1 | 18.820 | 18.840 | 18.830 | 28.844 | 28.617 | 28.731 | 0.001046 | 1.000000 |
| ABX2 | 19.933 | 18.480 | 19.207 | 29.063 | 28.398 | 28.731 | 0.001358 | 1.298189 |
| ABX3 | 18.483 | 18.613 | 18.548 | 27.612 | 27.612 | 27.612 | 0.001868 | 1.785713 |
| ABX4 | 20.348 | 20.803 | 20.576 | 29.520 | 29.767 | 29.644 | 0.001863 | 1.780769 |
| ABX+Pro1 | 19.249 | 19.526 | 19.388 | 25.412 | 25.673 | 25.543 | 0.014033 | 13.412442 |
| ABX+Pro2 | 19.510 | 19.519 | 19.515 | 25.780 | 25.780 | 25.780 | 0.012999 | 12.423502 |
| ABX+Pro3 | 19.512 | 19.458 | 19.485 | 26.175 | 26.059 | 26.117 | 0.010083 | 9.636438 |
| ABX+Pro4 | 21.893 | 21.506 | 21.700 | 27.416 | 27.811 | 27.614 | 0.016585 | 15.850979 |

|  | **β-actin (reference gene)** | | | **Zo-1 (target gene)** | | |  |  |
| --- | --- | --- | --- | --- | --- | --- | --- | --- |
|  | **Ct_1_** | **Ct_2_** | **Mean Ct** | **Ct_1_** | **Ct_2_** | **Mean Ct** | **RE** | **NOR** |
| PBS1 | 18.903 | 18.782 | 18.843 | 25.689 | 25.689 | 25.689 | 0.008692 | 1.797913 |
| PBS2 | 17.874 | 17.924 | 17.899 | 23.931 | 23.931 | 23.931 | 0.015281 | 3.161052 |
| PBS3 | 17.958 | 18.655 | 18.307 | 23.890 | 24.583 | 24.236 | 0.016403 | 3.393187 |
| PBS4 | 18.332 | 18.492 | 18.412 | 24.847 | 25.188 | 25.018 | 0.010268 | 2.124051 |
| Probiotics1 | 23.335 | 23.715 | 23.525 | 28.583 | 28.942 | 28.763 | 0.026510 | 5.483916 |
| Probiotics2 | 19.608 | 19.527 | 19.568 | 25.357 | 24.564 | 24.961 | 0.023798 | 4.922871 |
| Probiotics3 | 19.813 | 19.570 | 19.692 | 24.444 | 24.444 | 24.444 | 0.037098 | 7.674113 |
| Probiotics4 | 21.145 | 21.145 | 21.145 | 25.892 | 25.892 | 25.892 | 0.037240 | 7.703425 |
| ABX1 | 18.820 | 18.840 | 18.830 | 26.039 | 26.039 | 26.039 | 0.006759 | 1.398131 |
| ABX2 | 19.933 | 18.480 | 19.207 | 26.899 | 26.899 | 26.899 | 0.004834 | 1.000000 |
| ABX3 | 18.483 | 18.613 | 18.548 | 25.826 | 25.859 | 25.843 | 0.006370 | 1.317680 |
| ABX4 | 20.348 | 20.803 | 20.576 | 28.117 | 27.679 | 27.898 | 0.006248 | 1.292353 |
| ABX+Pro1 | 19.249 | 19.526 | 19.388 | 25.619 | 25.619 | 25.619 | 0.013309 | 2.752991 |
| ABX+Pro2 | 19.510 | 19.519 | 19.515 | 25.645 | 25.645 | 25.645 | 0.014274 | 2.952629 |
| ABX+Pro3 | 19.512 | 19.458 | 19.485 | 25.864 | 25.864 | 25.864 | 0.012015 | 2.485438 |
| ABX+Pro4 | 21.893 | 21.506 | 21.700 | 28.174 | 28.004 | 28.089 | 0.011928 | 2.467414 |

**Figure 3K**

|  | **16S rDNA (reference gene)** | | | **L.reuteri (target gene)** | | |  |  |
| --- | --- | --- | --- | --- | --- | --- | --- | --- |
|  | **Ct_1_** | **Ct_2_** | **Mean Ct** | **Ct_1_** | **Ct_2_** | **Mean Ct** | **RE** | **NOR** |
| PBS1 | 14.889 | 15.074 | 14.982 | 18.681 | 18.776 | 18.729 | 0.074480 | 13.445020 |
| PBS2 | 15.097 | 15.319 | 15.208 | 19.473 | 20.056 | 19.765 | 0.042497 | 7.671454 |
| PBS3 | 20.190 | 18.616 | 19.403 | 22.895 | 23.201 | 23.048 | 0.079937 | 14.430006 |
| PBS4 | 15.533 | 15.887 | 15.710 | 19.749 | 19.779 | 19.764 | 0.060204 | 10.867890 |
| Probiotics1 | 18.830 | 18.937 | 18.884 | 21.653 | 20.976 | 21.315 | 0.185437 | 33.474718 |
| Probiotics2 | 19.234 | 19.390 | 19.312 | 21.166 | 21.239 | 21.203 | 0.269714 | 48.688191 |
| Probiotics3 | 15.385 | 15.212 | 15.299 | 18.566 | 18.089 | 18.328 | 0.122512 | 22.115715 |
| Probiotics4 | 16.054 | 16.047 | 16.051 | 18.137 | 18.167 | 18.152 | 0.233016 | 42.063588 |
| ABX1 | 17.268 | 17.268 | 17.268 | 24.764 | 24.764 | 24.764 | 0.005540 | 1.000000 |
| ABX2 | 18.189 | 18.120 | 18.155 | 24.639 | 24.639 | 24.639 | 0.011168 | 2.016006 |
| ABX3 | 16.836 | 16.755 | 16.796 | 23.880 | 23.716 | 23.798 | 0.007799 | 1.407856 |
| ABX4 | 16.481 | 16.692 | 16.587 | 23.942 | 23.922 | 23.932 | 0.006149 | 1.109954 |
| ABX+Pro1 | 20.640 | 20.912 | 20.776 | 23.764 | 23.764 | 23.764 | 0.126044 | 22.753239 |
| ABX+Pro2 | 18.967 | 18.967 | 18.967 | 22.506 | 22.027 | 22.267 | 0.101567 | 18.334640 |
| ABX+Pro3 | 15.360 | 15.310 | 15.335 | 18.299 | 18.299 | 18.299 | 0.128158 | 23.134917 |
| ABX+Pro4 | 15.012 | 15.012 | 15.012 | 17.519 | 17.519 | 17.519 | 0.175921 | 31.756940 |

**Figure 3L**

|  | **16S rDNA (reference gene)** | | | **L.rhamnosus (target gene)** | | |  |  |
| --- | --- | --- | --- | --- | --- | --- | --- | --- |
|  | **Ct_1_** | **Ct_2_** | **Mean Ct** | **Ct_1_** | **Ct_2_** | **Mean Ct** | **RE** | **NOR** |
| PBS1 | 14.889 | 15.074 | 14.982 | 20.187 | 20.187 | 20.187 | 0.027101 | 8.969329 |
| PBS2 | 15.097 | 15.319 | 15.208 | 19.854 | 19.834 | 19.844 | 0.040218 | 13.310566 |
| PBS3 | 20.190 | 18.616 | 19.403 | 23.451 | 23.451 | 23.451 | 0.060455 | 20.007930 |
| PBS4 | 15.533 | 15.887 | 15.710 | 21.154 | 20.840 | 20.997 | 0.025613 | 8.476684 |
| Probiotics1 | 18.830 | 18.937 | 18.884 | 21.379 | 21.759 | 21.569 | 0.155448 | 51.446463 |
| Probiotics2 | 19.234 | 19.390 | 19.312 | 22.389 | 21.825 | 22.107 | 0.144086 | 47.686203 |
| Probiotics3 | 15.385 | 15.212 | 15.299 | 18.626 | 18.446 | 18.536 | 0.106027 | 35.090291 |
| Probiotics4 | 16.054 | 16.047 | 16.051 | 19.594 | 19.571 | 19.583 | 0.086449 | 28.611039 |
| ABX1 | 17.268 | 17.268 | 17.268 | 24.956 | 24.043 | 24.500 | 0.006654 | 2.202283 |
| ABX2 | 18.189 | 18.120 | 18.155 | 25.973 | 25.973 | 25.973 | 0.004430 | 1.466117 |
| ABX3 | 16.836 | 16.755 | 16.796 | 24.838 | 24.838 | 24.838 | 0.003793 | 1.255272 |
| ABX4 | 16.481 | 16.692 | 16.587 | 25.312 | 24.602 | 24.957 | 0.003022 | 1.000000 |
| ABX+Pro1 | 20.640 | 20.912 | 20.776 | 24.718 | 24.414 | 24.566 | 0.072293 | 23.925879 |
| ABX+Pro2 | 18.967 | 18.967 | 18.967 | 22.991 | 22.991 | 22.991 | 0.061469 | 20.343556 |
| ABX+Pro3 | 15.360 | 15.310 | 15.335 | 19.420 | 19.420 | 19.420 | 0.058924 | 19.501322 |
| ABX+Pro4 | 15.012 | 15.012 | 15.012 | 18.258 | 18.258 | 18.258 | 0.105404 | 34.884155 |

**Figure 3M**

|  | **16S rDNA (reference gene)** | | | **Morganella (target gene)** | | |  |  |
| --- | --- | --- | --- | --- | --- | --- | --- | --- |
|  | **Ct_1_** | **Ct_2_** | **Mean Ct** | **Ct_1_** | **Ct_2_** | **Mean Ct** | **RE** | **NOR** |
| PBS1 | 18.751 | 18.751 | 18.751 | 18.476 | 18.476 | 18.476 | 1.209994 | 11.660033 |
| PBS2 | 15.337 | 16.780 | 16.059 | 16.679 | 16.365 | 16.522 | 0.725225 | 6.988583 |
| PBS3 | 16.085 | 16.085 | 16.085 | 16.207 | 16.207 | 16.207 | 0.918913 | 8.855047 |
| PBS4 | 18.177 | 18.177 | 18.177 | 17.867 | 17.867 | 17.867 | 1.239708 | 11.946367 |
| Probiotics1 | 21.336 | 22.443 | 21.890 | 24.715 | 25.601 | 25.158 | 0.103773 | 1.000000 |
| Probiotics2 | 16.961 | 16.961 | 16.961 | 19.683 | 19.683 | 19.683 | 0.151564 | 1.460538 |
| Probiotics3 | 15.957 | 15.224 | 15.591 | 18.094 | 18.350 | 18.222 | 0.161376 | 1.555092 |
| Probiotics4 | 20.256 | 20.486 | 20.371 | 22.927 | 22.962 | 22.945 | 0.167996 | 1.618884 |
| ABX1 | 22.975 | 22.422 | 22.699 | 20.773 | 21.567 | 21.170 | 2.884857 | 27.799750 |
| ABX2 | 17.171 | 16.842 | 17.007 | 16.006 | 16.506 | 16.256 | 1.682376 | 16.212110 |
| ABX3 | 21.065 | 21.714 | 21.390 | 19.774 | 19.829 | 19.802 | 3.006323 | 28.970245 |
| ABX4 | 19.884 | 19.230 | 19.557 | 17.999 | 17.518 | 17.759 | 3.478584 | 33.521156 |
| ABX+Pro1 | 19.539 | 18.923 | 19.231 | 21.544 | 21.795 | 21.670 | 0.184475 | 1.777685 |
| ABX+Pro2 | 17.284 | 17.469 | 17.377 | 19.399 | 18.885 | 19.142 | 0.294125 | 2.834315 |
| ABX+Pro3 | 16.776 | 16.924 | 16.850 | 19.409 | 19.409 | 19.409 | 0.169693 | 1.635237 |
| ABX+Pro4 | 16.845 | 17.502 | 17.174 | 20.247 | 20.317 | 20.282 | 0.115944 | 1.117287 |

**Figure 4G**

|  | **β-actin (reference gene)** | | | **Atf4 (target gene)** | | |  |  |
| --- | --- | --- | --- | --- | --- | --- | --- | --- |
|  | **Ct_1_** | **Ct_2_** | **Mean Ct** | **Ct_1_** | **Ct_2_** | **Mean Ct** | **RE** | **NOR** |
| FLOX1 | 20.638 | 20.503 | 20.571 | 24.179 | 25.237 | 24.708 | 0.056837 | 3.317922 |
| FLOX2 | 21.857 | 21.828 | 21.842 | 25.564 | 25.371 | 25.467 | 0.081056 | 4.761051 |
| FLOX3 | 22.398 | 22.053 | 22.226 | 26.140 | 26.222 | 26.181 | 0.064474 | 3.178354 |
| FLOX4 | 22.891 | 23.239 | 23.065 | 27.555 | 27.213 | 27.384 | 0.050106 | 2.445127 |
| CKO1 | 24.091 | 23.635 | 23.863 | 29.894 | 29.467 | 29.681 | 0.017730 | 1.000000 |
| CKO2 | 23.916 | 23.530 | 23.723 | 28.420 | 28.316 | 28.368 | 0.039969 | 2.067346 |
| CKO3 | 22.605 | 22.980 | 22.793 | 27.098 | 27.456 | 27.277 | 0.044675 | 2.788095 |
| CKO4 | 20.612 | 20.258 | 20.435 | 26.019 | 26.253 | 26.136 | 0.019220 | 1.211446 |

|  | **β-actin (reference gene)** | | | **Slc7a11 (target gene)** | | |  |  |
| --- | --- | --- | --- | --- | --- | --- | --- | --- |
|  | **Ct_1_** | **Ct_2_** | **Mean Ct** | **Ct_1_** | **Ct_2_** | **Mean Ct** | **RE** | **NOR** |
| FLOX1 | 20.638 | 20.503 | 20.571 | 25.857 | 25.945 | 25.901 | 0.024864 | 6.268629 |
| FLOX2 | 21.857 | 21.828 | 21.842 | 28.229 | 27.972 | 28.100 | 0.013070 | 3.295127 |
| FLOX3 | 22.398 | 22.053 | 22.226 | 28.490 | 28.048 | 28.269 | 0.015165 | 3.823329 |
| FLOX4 | 22.891 | 23.239 | 23.065 | 29.084 | 29.024 | 29.054 | 0.015741 | 4.968401 |
| CKO1 | 24.091 | 23.635 | 23.863 | 30.490 | 30.670 | 30.580 | 0.009507 | 2.396720 |
| CKO2 | 23.916 | 23.530 | 23.723 | 30.752 | 30.114 | 30.433 | 0.009554 | 2.408652 |
| CKO3 | 22.605 | 22.980 | 22.793 | 29.785 | 29.455 | 29.620 | 0.008808 | 1.220507 |
| CKO4 | 20.612 | 20.258 | 20.435 | 28.582 | 28.244 | 28.413 | 0.003966 | 1.000000 |

|  | **β-actin (reference gene)** | | | **Slc3a2 (target gene)** | | |  |  |
| --- | --- | --- | --- | --- | --- | --- | --- | --- |
|  | **Ct_1_** | **Ct_2_** | **Mean Ct** | **Ct_1_** | **Ct_2_** | **Mean Ct** | **RE** | **NOR** |
| FLOX1 | 20.638 | 20.503 | 20.571 | 22.586 | 22.615 | 22.600 | 0.244950 | 2.896712 |
| FLOX2 | 21.857 | 21.828 | 21.842 | 23.972 | 24.362 | 24.167 | 0.199670 | 2.361245 |
| FLOX3 | 22.398 | 22.053 | 22.226 | 23.331 | 23.918 | 23.624 | 0.379242 | 4.484805 |
| FLOX4 | 22.891 | 23.239 | 23.065 | 23.932 | 24.571 | 24.251 | 0.439464 | 5.196975 |
| CKO1 | 24.091 | 23.635 | 23.863 | 27.219 | 27.575 | 27.397 | 0.086336 | 1.020990 |
| CKO2 | 23.916 | 23.530 | 23.723 | 27.312 | 27.262 | 27.287 | 0.084562 | 1.000000 |
| CKO3 | 22.605 | 22.980 | 22.793 | 25.534 | 25.534 | 25.534 | 0.149532 | 1.768317 |
| CKO4 | 20.612 | 20.258 | 20.435 | 22.837 | 23.006 | 22.921 | 0.178456 | 2.110365 |

|  | **β-actin (reference gene)** | | | **Gpx4 (target gene)** | | |  |  |
| --- | --- | --- | --- | --- | --- | --- | --- | --- |
|  | **Ct_1_** | **Ct_2_** | **Mean Ct** | **Ct_1_** | **Ct_2_** | **Mean Ct** | **RE** | **NOR** |
| FLOX1 | 20.638 | 20.503 | 20.571 | 24.218 | 24.458 | 24.338 | 0.073468 | 3.297468 |
| FLOX2 | 21.857 | 21.828 | 21.842 | 26.073 | 26.298 | 26.186 | 0.049264 | 2.211132 |
| FLOX3 | 22.398 | 22.053 | 22.226 | 26.795 | 26.094 | 26.445 | 0.053700 | 2.410215 |
| FLOX4 | 22.891 | 23.239 | 23.065 | 27.112 | 27.097 | 27.104 | 0.060810 | 2.729356 |
| CKO1 | 24.091 | 23.635 | 23.863 | 28.789 | 29.913 | 29.351 | 0.022280 | 1.000000 |
| CKO2 | 23.916 | 23.530 | 23.723 | 28.907 | 28.671 | 28.789 | 0.029849 | 1.339726 |
| CKO3 | 22.605 | 22.980 | 22.793 | 27.856 | 27.095 | 27.475 | 0.038936 | 1.747561 |
| CKO4 | 20.612 | 20.258 | 20.435 | 25.067 | 25.905 | 25.486 | 0.030170 | 1.354126 |

**Figure 4R**

|  | **β-actin (reference gene)** | | | **Occludin (target gene)** | | |  |  |
| --- | --- | --- | --- | --- | --- | --- | --- | --- |
|  | **Ct_1_** | **Ct_2_** | **Mean Ct** | **Ct_1_** | **Ct_2_** | **Mean Ct** | **RE** | **NOR** |
| DMSO1 | 18.793 | 19.089 | 18.941 | 28.494 | 28.935 | 28.715 | 0.001142 | 3.781510 |
| DMSO2 | 21.306 | 21.325 | 21.316 | 31.392 | 31.542 | 31.467 | 0.000879 | 2.910333 |
| DMSO3 | 20.387 | 20.305 | 20.346 | 31.854 | 32.224 | 32.039 | 0.000302 | 1.000000 |
| DMSO4 | 20.320 | 20.106 | 20.213 | 30.699 | 31.296 | 30.998 | 0.000567 | 1.876140 |
| Fer1 | 21.044 | 21.391 | 21.218 | 29.564 | 29.907 | 29.736 | 0.002727 | 9.026840 |
| Fer2 | 18.986 | 18.730 | 18.858 | 27.334 | 28.116 | 27.725 | 0.002142 | 7.088693 |
| Fer3 | 18.571 | 18.680 | 18.626 | 26.828 | 26.061 | 26.445 | 0.004429 | 14.660435 |
| Fer4 | 21.870 | 21.457 | 21.664 | 29.457 | 29.363 | 29.410 | 0.004658 | 15.418642 |

|  | **β-actin (reference gene)** | | | **Cldn2 (target gene)** | | |  |  |
| --- | --- | --- | --- | --- | --- | --- | --- | --- |
|  | **Ct_1_** | **Ct_2_** | **Mean Ct** | **Ct_1_** | **Ct_2_** | **Mean Ct** | **RE** | **NOR** |
| DMSO1 | 18.793 | 19.089 | 18.941 | 22.637 | 22.815 | 22.726 | 0.072555 | 2.827814 |
| DMSO2 | 21.306 | 21.325 | 21.316 | 25.205 | 25.440 | 25.323 | 0.062197 | 2.424118 |
| DMSO3 | 20.387 | 20.305 | 20.346 | 24.927 | 24.616 | 24.771 | 0.046562 | 1.814724 |
| DMSO4 | 20.320 | 20.106 | 20.213 | 25.456 | 25.538 | 25.497 | 0.025658 | 1.000000 |
| Fer1 | 21.044 | 21.391 | 21.218 | 23.987 | 24.095 | 24.041 | 0.141285 | 5.506534 |
| Fer2 | 18.986 | 18.730 | 18.858 | 22.210 | 21.505 | 21.858 | 0.125016 | 4.872462 |
| Fer3 | 18.571 | 18.680 | 18.626 | 22.572 | 22.002 | 22.287 | 0.079017 | 3.079675 |
| Fer4 | 21.870 | 21.457 | 21.664 | 24.734 | 25.128 | 24.931 | 0.103863 | 4.048034 |

|  | **β-actin (reference gene)** | | | **Cldn3 (target gene)** | | |  |  |
| --- | --- | --- | --- | --- | --- | --- | --- | --- |
|  | **Ct_1_** | **Ct_2_** | **Mean Ct** | **Ct_1_** | **Ct_2_** | **Mean Ct** | **RE** | **NOR** |
| DMSO1 | 18.793 | 19.089 | 18.941 | 23.648 | 24.756 | 24.202 | 0.026087 | 1.000000 |
| DMSO2 | 21.306 | 21.325 | 21.316 | 25.318 | 24.878 | 25.098 | 0.072674 | 2.785836 |
| DMSO3 | 20.387 | 20.305 | 20.346 | 25.145 | 25.011 | 25.078 | 0.037637 | 1.442755 |
| DMSO4 | 20.320 | 20.106 | 20.213 | 24.608 | 24.075 | 24.341 | 0.057171 | 2.191576 |
| Fer1 | 21.044 | 21.391 | 21.218 | 22.505 | 22.314 | 22.409 | 0.437727 | 16.779628 |
| Fer2 | 18.986 | 18.730 | 18.858 | 20.826 | 20.448 | 20.637 | 0.291383 | 11.169746 |
| Fer3 | 18.571 | 18.680 | 18.626 | 20.507 | 20.916 | 20.711 | 0.235597 | 9.031251 |
| Fer4 | 21.870 | 21.457 | 21.664 | 23.588 | 23.589 | 23.589 | 0.263356 | 10.095350 |

|  | **β-actin (reference gene)** | | | **Cldn4 (target gene)** | | |  |  |
| --- | --- | --- | --- | --- | --- | --- | --- | --- |
|  | **Ct_1_** | **Ct_2_** | **Mean Ct** | **Ct_1_** | **Ct_2_** | **Mean Ct** | **RE** | **NOR** |
| DMSO1 | 18.793 | 19.089 | 18.941 | 27.340 | 27.472 | 27.406 | 0.002830 | 1.000000 |
| DMSO2 | 21.306 | 21.325 | 21.316 | 28.560 | 28.209 | 28.385 | 0.007446 | 2.631667 |
| DMSO3 | 20.387 | 20.305 | 20.346 | 27.822 | 27.855 | 27.838 | 0.005555 | 1.963229 |
| DMSO4 | 20.320 | 20.106 | 20.213 | 27.265 | 27.880 | 27.573 | 0.006088 | 2.151426 |
| Fer1 | 21.044 | 21.391 | 21.218 | 26.366 | 26.259 | 26.312 | 0.029265 | 10.342520 |
| Fer2 | 18.986 | 18.730 | 18.858 | 23.992 | 23.431 | 23.711 | 0.034592 | 12.225432 |
| Fer3 | 18.571 | 18.680 | 18.626 | 24.270 | 24.203 | 24.237 | 0.020462 | 7.231557 |
| Fer4 | 21.870 | 21.457 | 21.664 | 28.102 | 28.669 | 28.386 | 0.009474 | 3.348215 |

|  | **β-actin (reference gene)** | | | **Zo-1 (target gene)** | | |  |  |
| --- | --- | --- | --- | --- | --- | --- | --- | --- |
|  | **Ct_1_** | **Ct_2_** | **Mean Ct** | **Ct_1_** | **Ct_2_** | **Mean Ct** | **RE** | **NOR** |
| DMSO1 | 18.793 | 19.089 | 18.941 | 24.426 | 24.398 | 24.412 | 0.022547 | 1.595175 |
| DMSO2 | 21.306 | 21.325 | 21.316 | 26.331 | 25.843 | 26.087 | 0.036613 | 2.590330 |
| DMSO3 | 20.387 | 20.305 | 20.346 | 26.602 | 26.380 | 26.491 | 0.014134 | 1.000000 |
| DMSO4 | 20.320 | 20.106 | 20.213 | 25.524 | 25.889 | 25.707 | 0.022193 | 1.570123 |
| Fer1 | 21.044 | 21.391 | 21.218 | 24.784 | 24.712 | 24.748 | 0.086548 | 6.123186 |
| Fer2 | 18.986 | 18.730 | 18.858 | 23.215 | 24.065 | 23.640 | 0.036347 | 2.571486 |
| Fer3 | 18.571 | 18.680 | 18.626 | 23.169 | 23.345 | 23.257 | 0.040350 | 2.854728 |
| Fer4 | 21.870 | 21.457 | 21.664 | 25.697 | 25.289 | 25.493 | 0.070344 | 4.976739 |

**Figure 5G**

|  | **β-actin (reference gene)** | | | **Occludin (target gene)** | | |  |  |
| --- | --- | --- | --- | --- | --- | --- | --- | --- |
|  | **Ct_1_** | **Ct_2_** | **Mean Ct** | **Ct_1_** | **Ct_2_** | **Mean Ct** | **RE** | **NOR** |
| Vehicle1 | 20.536 | 20.536 | 20.536 | 23.236 | 23.446 | 23.341 | 0.143129 | 12.248031 |
| Vehicle2 | 22.049 | 21.317 | 21.683 | 24.410 | 24.733 | 24.572 | 0.135017 | 11.553858 |
| Vehicle3 | 19.715 | 18.840 | 19.277 | 22.952 | 22.662 | 22.807 | 0.086574 | 7.408458 |
| Vehicle4 | 21.463 | 20.479 | 20.971 | 23.727 | 24.517 | 24.122 | 0.112605 | 9.636019 |
| ISRIB1 | 20.250 | 19.705 | 19.977 | 25.654 | 26.784 | 26.219 | 0.013216 | 1.130915 |
| ISRIB2 | 20.526 | 20.765 | 20.645 | 25.833 | 25.952 | 25.892 | 0.026333 | 2.253402 |
| ISRIB3 | 22.046 | 22.046 | 22.046 | 26.957 | 27.593 | 27.275 | 0.026667 | 2.282000 |
| ISRIB4 | 20.696 | 20.519 | 20.607 | 27.226 | 26.827 | 27.026 | 0.011686 | 1.000000 |

|  | **β-actin (reference gene)** | | | **Cldn2 (target gene)** | | |  |  |
| --- | --- | --- | --- | --- | --- | --- | --- | --- |
|  | **Ct_1_** | **Ct_2_** | **Mean Ct** | **Ct_1_** | **Ct_2_** | **Mean Ct** | **RE** | **NOR** |
| Vehicle1 | 20.536 | 20.536 | 20.536 | 23.318 | 23.308 | 23.313 | 0.145941 | 8.340650 |
| Vehicle2 | 22.049 | 21.317 | 21.683 | 23.871 | 23.228 | 23.549 | 0.274223 | 15.672095 |
| Vehicle3 | 19.715 | 18.840 | 19.277 | 21.634 | 21.866 | 21.750 | 0.180181 | 10.297516 |
| Vehicle4 | 21.463 | 20.479 | 20.971 | 22.825 | 22.643 | 22.734 | 0.294590 | 16.836092 |
| ISRIB1 | 20.250 | 19.705 | 19.977 | 25.342 | 25.585 | 25.463 | 0.022309 | 1.274989 |
| ISRIB2 | 20.526 | 20.765 | 20.645 | 25.583 | 25.575 | 25.579 | 0.032722 | 1.870076 |
| ISRIB3 | 22.046 | 22.046 | 22.046 | 27.112 | 27.024 | 27.068 | 0.030788 | 1.759536 |
| ISRIB4 | 20.696 | 20.519 | 20.607 | 26.168 | 26.720 | 26.444 | 0.017498 | 1.000000 |

|  | **β-actin (reference gene)** | | | **Cldn3 (target gene)** | | |  |  |
| --- | --- | --- | --- | --- | --- | --- | --- | --- |
|  | **Ct_1_** | **Ct_2_** | **Mean Ct** | **Ct_1_** | **Ct_2_** | **Mean Ct** | **RE** | **NOR** |
| Vehicle1 | 20.536 | 20.536 | 20.536 | 22.984 | 22.337 | 22.660 | 0.229406 | 1.284985 |
| Vehicle2 | 22.049 | 21.317 | 21.683 | 22.284 | 22.585 | 22.435 | 0.593917 | 3.326740 |
| Vehicle3 | 19.715 | 18.840 | 19.277 | 20.336 | 21.174 | 20.755 | 0.358985 | 2.010801 |
| Vehicle4 | 21.463 | 20.479 | 20.971 | 22.421 | 22.539 | 22.480 | 0.351302 | 1.967770 |
| ISRIB1 | 20.250 | 19.705 | 19.977 | 22.413 | 22.513 | 22.463 | 0.178528 | 1.000000 |
| ISRIB2 | 20.526 | 20.765 | 20.645 | 22.361 | 22.208 | 22.285 | 0.321053 | 1.798332 |
| ISRIB3 | 22.046 | 22.046 | 22.046 | 23.870 | 23.985 | 23.927 | 0.271439 | 1.520429 |
| ISRIB4 | 20.696 | 20.519 | 20.607 | 22.721 | 22.753 | 22.737 | 0.228566 | 1.280279 |

|  | **β-actin (reference gene)** | | | **Cldn4 (target gene)** | | |  |  |
| --- | --- | --- | --- | --- | --- | --- | --- | --- |
|  | **Ct_1_** | **Ct_2_** | **Mean Ct** | **Ct_1_** | **Ct_2_** | **Mean Ct** | **RE** | **NOR** |
| Vehicle1 | 20.536 | 20.536 | 20.536 | 24.775 | 24.312 | 24.543 | 0.062200 | 14.945181 |
| Vehicle2 | 22.049 | 21.317 | 21.683 | 26.309 | 25.645 | 25.977 | 0.050978 | 12.248907 |
| Vehicle3 | 19.715 | 18.840 | 19.277 | 24.602 | 24.260 | 24.431 | 0.028093 | 6.750072 |
| Vehicle4 | 21.463 | 20.479 | 20.971 | 26.530 | 26.521 | 26.525 | 0.021280 | 5.113023 |
| ISRIB1 | 20.250 | 19.705 | 19.977 | 27.452 | 27.423 | 27.438 | 0.005677 | 1.364098 |
| ISRIB2 | 20.526 | 20.765 | 20.645 | 28.260 | 28.785 | 28.522 | 0.004254 | 1.022211 |
| ISRIB3 | 22.046 | 22.046 | 22.046 | 30.151 | 29.759 | 29.955 | 0.004162 | 1.000000 |
| ISRIB4 | 20.696 | 20.519 | 20.607 | 27.966 | 27.764 | 27.865 | 0.006534 | 1.570060 |

|  | **β-actin (reference gene)** | | | **Zo-1 (target gene)** | | |  |  |
| --- | --- | --- | --- | --- | --- | --- | --- | --- |
|  | **Ct_1_** | **Ct_2_** | **Mean Ct** | **Ct_1_** | **Ct_2_** | **Mean Ct** | **RE** | **NOR** |
| Vehicle1 | 20.536 | 20.536 | 20.536 | 25.009 | 25.708 | 25.358 | 0.035354 | 3.027033 |
| Vehicle2 | 22.049 | 21.317 | 21.683 | 25.603 | 25.102 | 25.353 | 0.078574 | 6.727656 |
| Vehicle3 | 19.715 | 18.840 | 19.277 | 24.429 | 24.651 | 24.540 | 0.026048 | 2.230244 |
| Vehicle4 | 21.463 | 20.479 | 20.971 | 26.220 | 26.359 | 26.290 | 0.025059 | 2.145599 |
| ISRIB1 | 20.250 | 19.705 | 19.977 | 26.149 | 25.922 | 26.035 | 0.015009 | 1.285108 |
| ISRIB2 | 20.526 | 20.765 | 20.645 | 27.239 | 26.709 | 26.974 | 0.012442 | 1.065340 |
| ISRIB3 | 22.046 | 22.046 | 22.046 | 27.530 | 28.066 | 27.798 | 0.018560 | 1.589126 |
| ISRIB4 | 20.696 | 20.519 | 20.607 | 27.239 | 26.815 | 27.027 | 0.011679 | 1.000000 |

**Figure 5N**

|  | **β-actin (reference gene)** | | | **Slc7a11 (target gene)** | | |  |  |
| --- | --- | --- | --- | --- | --- | --- | --- | --- |
|  | **Ct_1_** | **Ct_2_** | **Mean Ct** | **Ct_1_** | **Ct_2_** | **Mean Ct** | **RE** | **NOR** |
| Vehicle1 | 20.760 | 20.619 | 20.690 | 28.426 | 28.161 | 28.294 | 0.005140 | 13.823016 |
| Vehicle2 | 20.947 | 20.558 | 20.752 | 28.916 | 28.946 | 28.931 | 0.003451 | 9.280821 |
| Vehicle3 | 22.454 | 22.213 | 22.333 | 29.957 | 29.917 | 29.937 | 0.005141 | 13.824478 |
| Vehicle4 | 21.330 | 21.330 | 21.330 | 28.727 | 28.517 | 28.622 | 0.006381 | 14.161160 |
| ISRIB1 | 20.224 | 20.332 | 20.278 | 31.907 | 31.435 | 31.671 | 0.000372 | 1.000000 |
| ISRIB2 | 21.662 | 21.851 | 21.756 | 31.970 | 32.391 | 32.180 | 0.000728 | 1.957732 |
| ISRIB3 | 20.709 | 20.997 | 20.853 | 31.447 | 31.420 | 31.433 | 0.000653 | 1.756516 |
| ISRIB4 | 21.494 | 21.621 | 21.558 | 32.192 | 30.953 | 31.572 | 0.000967 | 2.599897 |

|  | **β-actin (reference gene)** | | | **Slc3a2 (target gene)** | | |  |  |
| --- | --- | --- | --- | --- | --- | --- | --- | --- |
|  | **Ct_1_** | **Ct_2_** | **Mean Ct** | **Ct_1_** | **Ct_2_** | **Mean Ct** | **RE** | **NOR** |
| Vehicle1 | 20.760 | 20.619 | 20.690 | 28.508 | 28.193 | 28.351 | 0.004941 | 5.676591 |
| Vehicle2 | 20.947 | 20.558 | 20.752 | 28.377 | 28.900 | 28.639 | 0.004227 | 4.855716 |
| Vehicle3 | 22.454 | 22.213 | 22.333 | 30.444 | 30.235 | 30.340 | 0.003889 | 4.467521 |
| Vehicle4 | 21.330 | 21.330 | 21.330 | 29.825 | 29.643 | 29.734 | 0.002951 | 3.390351 |
| ISRIB1 | 20.224 | 20.332 | 20.278 | 30.174 | 30.509 | 30.342 | 0.000935 | 1.073654 |
| ISRIB2 | 21.662 | 21.851 | 21.756 | 31.870 | 31.975 | 31.922 | 0.000870 | 1.000000 |
| ISRIB3 | 20.709 | 20.997 | 20.853 | 30.665 | 30.345 | 30.505 | 0.001243 | 1.427747 |
| ISRIB4 | 21.494 | 21.621 | 21.558 | 31.450 | 31.578 | 31.514 | 0.001006 | 1.156259 |

|  | **β-actin (reference gene)** | | | **Gpx4 (target gene)** | | |  |  |
| --- | --- | --- | --- | --- | --- | --- | --- | --- |
|  | **Ct_1_** | **Ct_2_** | **Mean Ct** | **Ct_1_** | **Ct_2_** | **Mean Ct** | **RE** | **NOR** |
| Vehicle1 | 20.760 | 20.619 | 20.690 | 26.153 | 26.774 | 26.464 | 0.018275 | 8.852329 |
| Vehicle2 | 20.947 | 20.558 | 20.752 | 26.273 | 26.868 | 26.571 | 0.017724 | 8.585096 |
| Vehicle3 | 22.454 | 22.213 | 22.333 | 28.678 | 28.178 | 28.428 | 0.014633 | 7.087929 |
| Vehicle4 | 21.330 | 21.330 | 21.330 | 27.421 | 27.539 | 27.480 | 0.014077 | 6.818850 |
| ISRIB1 | 20.224 | 20.332 | 20.278 | 29.111 | 29.116 | 29.114 | 0.002188 | 1.060076 |
| ISRIB2 | 21.662 | 21.851 | 21.756 | 30.345 | 30.274 | 30.309 | 0.002663 | 1.289948 |
| ISRIB3 | 20.709 | 20.997 | 20.853 | 29.834 | 29.712 | 29.773 | 0.002064 | 1.000000 |
| ISRIB4 | 21.494 | 21.621 | 21.558 | 30.126 | 30.151 | 30.139 | 0.002612 | 1.265230 |

**Figure 5V**

|  | **β-actin (reference gene)** | | | **Occludin (target gene)** | | |  |  |
| --- | --- | --- | --- | --- | --- | --- | --- | --- |
|  | **Ct_1_** | **Ct_2_** | **Mean Ct** | **Ct_1_** | **Ct_2_** | **Mean Ct** | **RE** | **NOR** |
| FLOX1 | 23.220 | 23.440 | 23.330 | 36.571 | 35.662 | 36.117 | 0.000142 | 3.547060 |
| FLOX2 | 21.768 | 21.715 | 21.741 | 34.223 | 34.920 | 34.571 | 0.000137 | 3.441818 |
| FLOX3 | 21.536 | 20.764 | 21.150 | 33.655 | 32.468 | 33.061 | 0.000260 | 6.505436 |
| FLOX4 | 23.765 | 23.440 | 23.602 | 35.266 | 35.956 | 35.611 | 0.000243 | 6.081223 |
| CKO1 | 23.198 | 23.433 | 23.315 | 37.626 | 37.626 | 37.626 | 0.000049 | 1.233766 |
| CKO2 | 22.326 | 21.788 | 22.057 | 36.456 | 36.884 | 36.670 | 0.000040 | 1.000000 |
| CKO3 | 22.959 | 22.953 | 22.956 | 36.469 | 36.092 | 36.280 | 0.000097 | 2.443293 |
| CKO4 | 23.561 | 23.723 | 23.642 | 37.623 | 36.304 | 36.963 | 0.000098 | 2.448549 |

|  | **β-actin (reference gene)** | | | **Cldn2 (target gene)** | | |  |  |
| --- | --- | --- | --- | --- | --- | --- | --- | --- |
|  | **Ct_1_** | **Ct_2_** | **Mean Ct** | **Ct_1_** | **Ct_2_** | **Mean Ct** | **RE** | **NOR** |
| FLOX1 | 23.220 | 23.440 | 23.330 | 25.306 | 25.121 | 25.213 | 0.271076 | 4.136989 |
| FLOX2 | 21.768 | 21.715 | 21.741 | 24.298 | 24.374 | 24.336 | 0.165505 | 2.525839 |
| FLOX3 | 21.536 | 20.764 | 21.150 | 22.891 | 23.102 | 22.997 | 0.277958 | 4.242022 |
| FLOX4 | 23.765 | 23.440 | 23.602 | 25.926 | 25.474 | 25.700 | 0.233722 | 3.566929 |
| CKO1 | 23.198 | 23.433 | 23.315 | 25.522 | 25.872 | 25.697 | 0.191949 | 2.929407 |
| CKO2 | 22.326 | 21.788 | 22.057 | 26.291 | 25.686 | 25.989 | 0.065525 | 1.000000 |
| CKO3 | 22.959 | 22.953 | 22.956 | 25.507 | 26.488 | 25.997 | 0.121462 | 1.853673 |
| CKO4 | 23.561 | 23.723 | 23.642 | 27.478 | 26.230 | 26.854 | 0.107919 | 1.646993 |

|  | **β-actin (reference gene)** | | | **Cldn3 (target gene)** | | |  |  |
| --- | --- | --- | --- | --- | --- | --- | --- | --- |
|  | **Ct_1_** | **Ct_2_** | **Mean Ct** | **Ct_1_** | **Ct_2_** | **Mean Ct** | **RE** | **NOR** |
| FLOX1 | 23.220 | 23.440 | 23.330 | 24.586 | 24.358 | 24.472 | 0.453057 | 2.425564 |
| FLOX2 | 21.768 | 21.715 | 21.741 | 23.544 | 23.297 | 23.421 | 0.312182 | 1.671348 |
| FLOX3 | 21.536 | 20.764 | 21.150 | 23.440 | 23.059 | 23.250 | 0.233281 | 1.248934 |
| FLOX4 | 23.765 | 23.440 | 23.602 | 25.728 | 24.895 | 25.312 | 0.305858 | 1.637495 |
| CKO1 | 23.198 | 23.433 | 23.315 | 25.491 | 25.431 | 25.461 | 0.226016 | 1.210039 |
| CKO2 | 22.326 | 21.788 | 22.057 | 23.834 | 24.002 | 23.918 | 0.275286 | 1.473818 |
| CKO3 | 22.959 | 22.953 | 22.956 | 24.708 | 24.808 | 24.758 | 0.286820 | 1.535569 |
| CKO4 | 23.561 | 23.723 | 23.642 | 26.371 | 25.754 | 26.062 | 0.186784 | 1.000000 |

|  | **β-actin (reference gene)** | | | **Cldn4 (target gene)** | | |  |  |
| --- | --- | --- | --- | --- | --- | --- | --- | --- |
|  | **Ct_1_** | **Ct_2_** | **Mean Ct** | **Ct_1_** | **Ct_2_** | **Mean Ct** | **RE** | **NOR** |
| FLOX1 | 23.220 | 23.440 | 23.330 | 27.734 | 28.351 | 28.042 | 0.038149 | 3.130706 |
| FLOX2 | 21.768 | 21.715 | 21.741 | 26.891 | 27.139 | 27.015 | 0.025847 | 2.121148 |
| FLOX3 | 21.536 | 20.764 | 21.150 | 27.045 | 26.343 | 26.694 | 0.021431 | 1.758730 |
| FLOX4 | 23.765 | 23.440 | 23.602 | 29.075 | 28.517 | 28.796 | 0.027327 | 2.242602 |
| CKO1 | 23.198 | 23.433 | 23.315 | 29.174 | 29.279 | 29.226 | 0.016619 | 1.363845 |
| CKO2 | 22.326 | 21.788 | 22.057 | 27.376 | 27.878 | 27.627 | 0.021046 | 1.727208 |
| CKO3 | 22.959 | 22.953 | 22.956 | 29.421 | 28.053 | 28.737 | 0.018184 | 1.492302 |
| CKO4 | 23.561 | 23.723 | 23.642 | 29.799 | 30.202 | 30.001 | 0.012185 | 1.000000 |

|  | **β-actin (reference gene)** | | | **Zo-1 (target gene)** | | |  |  |
| --- | --- | --- | --- | --- | --- | --- | --- | --- |
|  | **Ct_1_** | **Ct_2_** | **Mean Ct** | **Ct_1_** | **Ct_2_** | **Mean Ct** | **RE** | **NOR** |
| FLOX1 | 23.220 | 23.440 | 23.330 | 24.633 | 24.753 | 24.693 | 0.388697 | 19.496882 |
| FLOX2 | 21.768 | 21.715 | 21.741 | 23.997 | 24.445 | 24.221 | 0.179260 | 8.991608 |
| FLOX3 | 21.536 | 20.764 | 21.150 | 22.420 | 22.975 | 22.697 | 0.342095 | 17.159311 |
| FLOX4 | 23.765 | 23.440 | 23.602 | 25.583 | 26.783 | 26.183 | 0.167173 | 8.385339 |
| CKO1 | 23.198 | 23.433 | 23.315 | 27.934 | 26.504 | 27.219 | 0.066815 | 3.351417 |
| CKO2 | 22.326 | 21.788 | 22.057 | 27.298 | 28.113 | 27.705 | 0.019936 | 1.000000 |
| CKO3 | 22.959 | 22.953 | 22.956 | 27.963 | 28.050 | 28.006 | 0.030178 | 1.513718 |
| CKO4 | 23.561 | 23.723 | 23.642 | 26.704 | 27.586 | 27.145 | 0.088201 | 4.424102 |

**Figure 6E**

|  | **β-actin (reference gene)** | | | **Occludin (target gene)** | | |  |  |
| --- | --- | --- | --- | --- | --- | --- | --- | --- |
|  | **Ct_1_** | **Ct_2_** | **Mean Ct** | **Ct_1_** | **Ct_2_** | **Mean Ct** | **RE** | **NOR** |
| PBS1 | 22.157 | 22.678 | 22.417 | 34.537 | 33.703 | 34.120 | 0.000300 | 1.558244 |
| PBS2 | 23.484 | 22.844 | 23.164 | 34.708 | 33.180 | 33.944 | 0.000569 | 2.953673 |
| PBS3 | 22.089 | 22.231 | 22.160 | 34.203 | 34.495 | 34.349 | 0.000214 | 1.112237 |
| PBS4 | 22.489 | 22.495 | 22.492 | 34.281 | 35.388 | 34.834 | 0.000193 | 1.000000 |
| I3A1 | 20.530 | 20.916 | 20.723 | 28.541 | 29.123 | 28.832 | 0.003622 | 18.811356 |
| I3A2 | 21.190 | 20.929 | 21.059 | 29.957 | 29.698 | 29.828 | 0.002294 | 11.911330 |
| I3A3 | 19.851 | 20.345 | 20.098 | 27.888 | 28.315 | 28.102 | 0.003896 | 20.234069 |
| I3A4 | 19.947 | 20.481 | 20.214 | 28.088 | 28.531 | 28.310 | 0.003655 | 18.982489 |

|  | **β-actin (reference gene)** | | | **Cldn2 (target gene)** | | |  |  |
| --- | --- | --- | --- | --- | --- | --- | --- | --- |
|  | **Ct_1_** | **Ct_2_** | **Mean Ct** | **Ct_1_** | **Ct_2_** | **Mean Ct** | **RE** | **NOR** |
| PBS1 | 22.157 | 22.678 | 22.417 | 27.788 | 27.600 | 27.694 | 0.025794 | 1.208763 |
| PBS2 | 23.484 | 22.844 | 23.164 | 26.686 | 26.915 | 26.800 | 0.080402 | 3.767746 |
| PBS3 | 22.089 | 22.231 | 22.160 | 26.338 | 26.410 | 26.374 | 0.053891 | 2.525403 |
| PBS4 | 22.489 | 22.495 | 22.492 | 28.511 | 27.574 | 28.042 | 0.021340 | 1.000000 |
| I3A1 | 20.530 | 20.916 | 20.723 | 23.978 | 24.130 | 24.054 | 0.099383 | 4.657208 |
| I3A2 | 21.190 | 20.929 | 21.059 | 25.096 | 24.561 | 24.829 | 0.073343 | 3.436946 |
| I3A3 | 19.851 | 20.345 | 20.098 | 23.345 | 23.069 | 23.207 | 0.115896 | 5.431034 |
| I3A4 | 19.947 | 20.481 | 20.214 | 22.888 | 23.023 | 22.956 | 0.149495 | 7.005523 |

|  | **β-actin (reference gene)** | | | **Cldn3 (target gene)** | | |  |  |
| --- | --- | --- | --- | --- | --- | --- | --- | --- |
|  | **Ct_1_** | **Ct_2_** | **Mean Ct** | **Ct_1_** | **Ct_2_** | **Mean Ct** | **RE** | **NOR** |
| PBS1 | 22.157 | 22.678 | 22.417 | 26.963 | 26.364 | 26.664 | 0.052688 | 1.000000 |
| PBS2 | 23.484 | 22.844 | 23.164 | 26.484 | 27.645 | 27.065 | 0.066938 | 1.270447 |
| PBS3 | 22.089 | 22.231 | 22.160 | 25.336 | 25.484 | 25.410 | 0.105118 | 1.995095 |
| PBS4 | 22.489 | 22.495 | 22.492 | 26.424 | 26.425 | 26.425 | 0.065490 | 1.242974 |
| I3A1 | 20.530 | 20.916 | 20.723 | 22.636 | 22.646 | 22.641 | 0.264678 | 5.023472 |
| I3A2 | 21.190 | 20.929 | 21.059 | 22.088 | 23.272 | 22.680 | 0.325274 | 6.173559 |
| I3A3 | 19.851 | 20.345 | 20.098 | 22.122 | 22.216 | 22.169 | 0.238002 | 4.517181 |
| I3A4 | 19.947 | 20.481 | 20.214 | 21.158 | 20.843 | 21.001 | 0.579615 | 11.000844 |

|  | **β-actin (reference gene)** | | | **Cldn4 (target gene)** | | |  |  |
| --- | --- | --- | --- | --- | --- | --- | --- | --- |
|  | **Ct_1_** | **Ct_2_** | **Mean Ct** | **Ct_1_** | **Ct_2_** | **Mean Ct** | **RE** | **NOR** |
| PBS1 | 22.157 | 22.678 | 22.417 | 26.245 | 29.677 | 27.961 | 0.021438 | 2.148334 |
| PBS2 | 23.484 | 22.844 | 23.164 | 28.446 | 29.297 | 28.872 | 0.019131 | 1.917126 |
| PBS3 | 22.089 | 22.231 | 22.160 | 26.673 | 27.218 | 26.945 | 0.036264 | 3.633999 |
| PBS4 | 22.489 | 22.495 | 22.492 | 28.961 | 29.317 | 29.139 | 0.009979 | 1.000000 |
| I3A1 | 20.530 | 20.916 | 20.723 | 24.844 | 23.054 | 23.949 | 0.106907 | 10.713204 |
| I3A2 | 21.190 | 20.929 | 21.059 | 25.340 | 24.627 | 24.984 | 0.065864 | 6.600307 |
| I3A3 | 19.851 | 20.345 | 20.098 | 23.456 | 22.981 | 23.219 | 0.114963 | 11.520471 |
| I3A4 | 19.947 | 20.481 | 20.214 | 24.477 | 23.064 | 23.771 | 0.084974 | 8.515266 |

|  | **β-actin (reference gene)** | | | **Zo-1 (target gene)** | | |  |  |
| --- | --- | --- | --- | --- | --- | --- | --- | --- |
|  | **Ct_1_** | **Ct_2_** | **Mean Ct** | **Ct_1_** | **Ct_2_** | **Mean Ct** | **RE** | **NOR** |
| PBS1 | 22.157 | 22.678 | 22.417 | 26.005 | 27.088 | 26.547 | 0.057138 | 2.895535 |
| PBS2 | 23.484 | 22.844 | 23.164 | 27.854 | 28.108 | 27.981 | 0.035473 | 1.797648 |
| PBS3 | 22.089 | 22.231 | 22.160 | 27.207 | 28.440 | 27.823 | 0.019733 | 1.000000 |
| PBS4 | 22.489 | 22.495 | 22.492 | 26.590 | 27.025 | 26.808 | 0.050222 | 2.545032 |
| I3A1 | 20.530 | 20.916 | 20.723 | 23.516 | 24.198 | 23.857 | 0.113915 | 5.772788 |
| I3A2 | 21.190 | 20.929 | 21.059 | 22.602 | 23.137 | 22.870 | 0.285153 | 14.450413 |
| I3A3 | 19.851 | 20.345 | 20.098 | 22.864 | 22.751 | 22.807 | 0.152893 | 7.748016 |
| I3A4 | 19.947 | 20.481 | 20.214 | 23.391 | 22.517 | 22.954 | 0.149646 | 7.583481 |

**Figure 6N**

|  | **β-actin (reference gene)** | | | **Slc7a11 (target gene)** | | |  |  |
| --- | --- | --- | --- | --- | --- | --- | --- | --- |
|  | **Ct_1_** | **Ct_2_** | **Mean Ct** | **Ct_1_** | **Ct_2_** | **Mean Ct** | **RE** | **NOR** |
| PBS1 | 20.388 | 20.515 | 20.451 | 28.259 | 28.632 | 28.446 | 0.003921 | 1.056481 |
| PBS2 | 20.453 | 20.258 | 20.356 | 28.454 | 28.404 | 28.429 | 0.003712 | 1.000000 |
| PBS3 | 22.996 | 22.996 | 22.996 | 30.189 | 30.100 | 30.144 | 0.007048 | 1.898757 |
| PBS4 | 21.539 | 21.935 | 21.737 | 29.889 | 29.117 | 29.503 | 0.004595 | 1.238081 |
| I3A1 | 22.717 | 21.008 | 21.862 | 28.488 | 27.588 | 28.038 | 0.013833 | 3.726730 |
| I3A2 | 20.136 | 20.136 | 20.136 | 26.108 | 26.380 | 26.244 | 0.014495 | 3.905250 |
| I3A3 | 21.457 | 21.959 | 21.708 | 27.969 | 27.047 | 27.508 | 0.017950 | 4.835984 |
| I3A4 | 20.147 | 20.246 | 20.196 | 26.789 | 26.087 | 26.438 | 0.013213 | 3.559763 |

|  | **β-actin (reference gene)** | | | **Gpx4 (target gene)** | | |  |  |
| --- | --- | --- | --- | --- | --- | --- | --- | --- |
|  | **Ct_1_** | **Ct_2_** | **Mean Ct** | **Ct_1_** | **Ct_2_** | **Mean Ct** | **RE** | **NOR** |
| PBS1 | 20.617 | 20.485 | 20.551 | 26.541 | 26.846 | 26.693 | 0.014157 | 1.279872 |
| PBS2 | 20.267 | 19.857 | 20.062 | 26.181 | 26.130 | 26.155 | 0.014647 | 1.000000 |
| PBS3 | 20.915 | 20.896 | 20.906 | 26.825 | 26.963 | 26.894 | 0.015748 | 1.118449 |
| PBS4 | 22.437 | 22.152 | 22.295 | 28.871 | 28.907 | 28.889 | 0.010347 | 1.171616 |
| I3A1 | 19.288 | 19.626 | 19.457 | 24.523 | 24.292 | 24.407 | 0.032338 | 3.480996 |
| I3A2 | 19.513 | 19.521 | 19.517 | 24.767 | 24.712 | 24.739 | 0.026786 | 2.922089 |
| I3A3 | 20.189 | 20.311 | 20.250 | 25.440 | 25.021 | 25.230 | 0.031677 | 3.092985 |
| I3A4 | 18.321 | 18.115 | 18.218 | 23.320 | 22.798 | 23.059 | 0.034882 | 3.897368 |

**Figure 6S**

|  | **β-actin (reference gene)** | | | **Occludin (target gene)** | | |  |  |
| --- | --- | --- | --- | --- | --- | --- | --- | --- |
|  | **Ct_1_** | **Ct_2_** | **Mean Ct** | **Ct_1_** | **Ct_2_** | **Mean Ct** | **RE** | **NOR** |
| DMSO1 | 22.499 | 22.271 | 22.385 | 24.323 | 24.323 | 24.323 | 0.260978 | 6.785711 |
| DMSO2 | 19.952 | 19.897 | 19.925 | 21.771 | 21.792 | 21.782 | 0.276050 | 7.177592 |
| DMSO3 | 21.233 | 21.361 | 21.297 | 23.203 | 23.203 | 23.203 | 0.266831 | 6.937905 |
| DMSO4 | 20.602 | 20.544 | 20.573 | 22.677 | 22.677 | 22.677 | 0.232612 | 6.048175 |
| CH-223191-1 | 19.801 | 19.732 | 19.767 | 24.386 | 24.386 | 24.386 | 0.040681 | 1.057751 |
| CH-223191-2 | 21.519 | 21.528 | 21.524 | 24.724 | 24.724 | 24.724 | 0.108781 | 2.828427 |
| CH-223191-3 | 19.883 | 20.135 | 20.009 | 24.342 | 24.342 | 24.342 | 0.049618 | 1.290115 |
| CH-223191-4 | 19.002 | 19.335 | 19.169 | 23.085 | 24.653 | 23.869 | 0.038460 | 1.000000 |

|  | **β-actin (reference gene)** | | | **Cldn2 (target gene)** | | |  |  |
| --- | --- | --- | --- | --- | --- | --- | --- | --- |
|  | **Ct_1_** | **Ct_2_** | **Mean Ct** | **Ct_1_** | **Ct_2_** | **Mean Ct** | **RE** | **NOR** |
| DMSO1 | 22.499 | 22.271 | 22.385 | 24.274 | 24.807 | 24.541 | 0.224455 | 5.880749 |
| DMSO2 | 19.952 | 19.897 | 19.925 | 21.930 | 21.930 | 21.930 | 0.249049 | 6.525100 |
| DMSO3 | 21.233 | 21.361 | 21.297 | 23.890 | 22.732 | 23.311 | 0.247586 | 6.486769 |
| DMSO4 | 20.602 | 20.544 | 20.573 | 22.707 | 22.494 | 22.601 | 0.245280 | 6.426352 |
| CH-223191-1 | 19.801 | 19.732 | 19.767 | 23.200 | 23.200 | 23.200 | 0.092558 | 2.425026 |
| CH-223191-2 | 21.519 | 21.528 | 21.524 | 26.007 | 26.463 | 26.235 | 0.038168 | 1.000000 |
| CH-223191-3 | 19.883 | 20.135 | 20.009 | 23.200 | 23.200 | 23.200 | 0.109500 | 2.868905 |
| CH-223191-4 | 19.002 | 19.335 | 19.169 | 23.324 | 23.324 | 23.324 | 0.056114 | 1.470187 |

|  | **β-actin (reference gene)** | | | **Cldn3 (target gene)** | | |  |  |
| --- | --- | --- | --- | --- | --- | --- | --- | --- |
|  | **Ct_1_** | **Ct_2_** | **Mean Ct** | **Ct_1_** | **Ct_2_** | **Mean Ct** | **RE** | **NOR** |
| DMSO1 | 22.499 | 22.271 | 22.385 | 21.494 | 21.494 | 21.494 | 1.854461 | 10.443258 |
| DMSO2 | 19.952 | 19.897 | 19.925 | 20.976 | 20.311 | 20.644 | 0.607518 | 3.421194 |
| DMSO3 | 21.233 | 21.361 | 21.297 | 20.875 | 20.875 | 20.875 | 1.339784 | 7.544891 |
| DMSO4 | 20.602 | 20.544 | 20.573 | 20.195 | 20.195 | 20.195 | 1.299539 | 7.318257 |
| CH-223191-1 | 19.801 | 19.732 | 19.767 | 22.022 | 22.498 | 22.260 | 0.177575 | 1.000000 |
| CH-223191-2 | 21.519 | 21.528 | 21.524 | 23.364 | 23.205 | 23.285 | 0.295044 | 1.661516 |
| CH-223191-3 | 19.883 | 20.135 | 20.009 | 21.608 | 21.608 | 21.608 | 0.330106 | 1.858966 |
| CH-223191-4 | 19.002 | 19.335 | 19.169 | 20.241 | 20.487 | 20.364 | 0.436635 | 2.458878 |

|  | **β-actin (reference gene)** | | | **Cldn4 (target gene)** | | |  |  |
| --- | --- | --- | --- | --- | --- | --- | --- | --- |
|  | **Ct_1_** | **Ct_2_** | **Mean Ct** | **Ct_1_** | **Ct_2_** | **Mean Ct** | **RE** | **NOR** |
| DMSO1 | 22.499 | 22.271 | 22.385 | 25.554 | 26.387 | 25.971 | 0.083302 | 5.759725 |
| DMSO2 | 19.952 | 19.897 | 19.925 | 24.137 | 24.137 | 24.137 | 0.053940 | 3.729546 |
| DMSO3 | 21.233 | 21.361 | 21.297 | 24.957 | 24.957 | 24.957 | 0.079110 | 5.469845 |
| DMSO4 | 20.602 | 20.544 | 20.573 | 23.435 | 24.528 | 23.982 | 0.094176 | 6.511545 |
| CH-223191-1 | 19.801 | 19.732 | 19.767 | 24.906 | 24.906 | 24.906 | 0.028370 | 1.961558 |
| CH-223191-2 | 21.519 | 21.528 | 21.524 | 25.992 | 25.973 | 25.983 | 0.045468 | 3.143779 |
| CH-223191-3 | 19.883 | 20.135 | 20.009 | 25.716 | 26.127 | 25.922 | 0.016602 | 1.147902 |
| CH-223191-4 | 19.002 | 19.335 | 19.169 | 25.090 | 25.470 | 25.280 | 0.014463 | 1.000000 |

|  | **β-actin (reference gene)** | | | **Zo-1 (target gene)** | | |  |  |
| --- | --- | --- | --- | --- | --- | --- | --- | --- |
|  | **Ct_1_** | **Ct_2_** | **Mean Ct** | **Ct_1_** | **Ct_2_** | **Mean Ct** | **RE** | **NOR** |
| DMSO1 | 22.499 | 22.271 | 22.385 | 29.141 | 29.188 | 29.165 | 0.009103 | 3.019898 |
| DMSO2 | 19.952 | 19.897 | 19.925 | 26.678 | 26.678 | 26.678 | 0.009268 | 3.074816 |
| DMSO3 | 21.233 | 21.361 | 21.297 | 28.498 | 28.366 | 28.432 | 0.007115 | 2.360349 |
| DMSO4 | 20.602 | 20.544 | 20.573 | 27.641 | 27.326 | 27.484 | 0.008313 | 2.757766 |
| CH-223191-1 | 19.801 | 19.732 | 19.767 | 27.835 | 27.119 | 27.477 | 0.004774 | 1.583921 |
| CH-223191-2 | 21.519 | 21.528 | 21.524 | 29.153 | 29.153 | 29.153 | 0.005050 | 1.675394 |
| CH-223191-3 | 19.883 | 20.135 | 20.009 | 27.401 | 27.401 | 27.401 | 0.005954 | 1.975202 |
| CH-223191-4 | 19.002 | 19.335 | 19.169 | 27.499 | 27.586 | 27.543 | 0.003014 | 1.000000 |

**Figure 6Z**

|  | **β-actin (reference gene)** | | | **Slc7a11 (target gene)** | | |  |  |
| --- | --- | --- | --- | --- | --- | --- | --- | --- |
|  | **Ct_1_** | **Ct_2_** | **Mean Ct** | **Ct_1_** | **Ct_2_** | **Mean Ct** | **RE** | **NOR** |
| DMSO1 | 22.654 | 22.557 | 22.606 | 27.447 | 27.570 | 27.509 | 0.033423 | 4.851732 |
| DMSO2 | 22.204 | 22.134 | 22.169 | 26.372 | 26.926 | 26.649 | 0.044811 | 6.504779 |
| DMSO3 | 20.449 | 20.879 | 20.664 | 26.618 | 26.146 | 26.382 | 0.018998 | 2.757766 |
| DMSO4 | 21.443 | 21.455 | 21.449 | 26.796 | 27.123 | 26.960 | 0.021937 | 3.184352 |
| CH-223191-1 | 21.159 | 21.820 | 21.490 | 27.875 | 27.935 | 27.905 | 0.011715 | 1.700548 |
| CH-223191-2 | 21.466 | 20.974 | 21.220 | 27.339 | 26.871 | 27.105 | 0.016921 | 2.456323 |
| CH-223191-3 | 21.469 | 21.454 | 21.462 | 28.854 | 27.990 | 28.422 | 0.008029 | 1.165541 |
| CH-223191-4 | 24.070 | 24.149 | 24.110 | 31.663 | 30.919 | 31.291 | 0.006889 | 1.000000 |

|  | **β-actin (reference gene)** | | | **Gpx4 (target gene)** | | |  |  |
| --- | --- | --- | --- | --- | --- | --- | --- | --- |
|  | **Ct_1_** | **Ct_2_** | **Mean Ct** | **Ct_1_** | **Ct_2_** | **Mean Ct** | **RE** | **NOR** |
| DMSO1 | 22.654 | 22.557 | 22.606 | 28.246 | 28.123 | 28.185 | 0.020920 | 5.577041 |
| DMSO2 | 22.204 | 22.134 | 22.169 | 27.309 | 26.630 | 26.970 | 0.035884 | 9.566558 |
| DMSO3 | 20.449 | 20.879 | 20.664 | 26.279 | 26.053 | 26.166 | 0.022066 | 5.882788 |
| DMSO4 | 21.443 | 21.455 | 21.449 | 27.520 | 27.020 | 27.270 | 0.017689 | 4.715792 |
| CH-223191-1 | 21.159 | 21.820 | 21.490 | 29.394 | 29.371 | 29.383 | 0.004207 | 1.121555 |
| CH-223191-2 | 21.466 | 20.974 | 21.220 | 28.034 | 27.662 | 27.848 | 0.010111 | 2.695401 |
| CH-223191-3 | 21.469 | 21.454 | 21.462 | 29.520 | 29.520 | 29.520 | 0.003751 | 1.000000 |
| CH-223191-4 | 24.070 | 24.149 | 24.110 | 31.237 | 31.237 | 31.237 | 0.007152 | 1.906597 |

**Figure 7F**

|  | **β-actin (reference gene)** | | | **Occludin (target gene)** | | |  |  |
| --- | --- | --- | --- | --- | --- | --- | --- | --- |
|  | **Ct_1_** | **Ct_2_** | **Mean Ct** | **Ct_1_** | **Ct_2_** | **Mean Ct** | **RE** | **NOR** |
| CKO1 | 24.165 | 23.592 | 23.879 | 34.654 | 34.022 | 34.338 | 0.000710 | 2.330772 |
| CKO2 | 19.434 | 19.142 | 19.288 | 30.311 | 30.516 | 30.414 | 0.000447 | 1.468488 |
| CKO3 | 21.756 | 21.299 | 21.527 | 33.461 | 32.955 | 33.208 | 0.000305 | 1.000000 |
| CKO4 | 21.549 | 21.217 | 21.383 | 33.331 | 32.349 | 32.840 | 0.000356 | 1.167582 |
| CKO+I3A1 | 21.044 | 21.050 | 21.047 | 29.675 | 29.966 | 29.821 | 0.002285 | 7.499019 |
| CKO+I3A2 | 22.191 | 21.699 | 21.945 | 30.847 | 30.900 | 30.873 | 0.002053 | 6.737594 |
| CKO+I3A3 | 21.493 | 21.491 | 21.492 | 31.373 | 31.388 | 31.380 | 0.001055 | 3.462344 |
| CKO+I3A4 | 22.191 | 21.699 | 21.945 | 30.346 | 30.847 | 30.596 | 0.002488 | 8.165677 |
| FLOX+I3A1 | 21.421 | 21.553 | 21.487 | 29.373 | 29.161 | 29.267 | 0.004550 | 14.933032 |
| FLOX+I3A2 | 20.209 | 19.426 | 19.818 | 26.862 | 26.120 | 26.491 | 0.009797 | 32.154198 |
| FLOX+I3A3 | 20.323 | 19.800 | 20.062 | 27.678 | 26.904 | 27.291 | 0.006664 | 21.870817 |
| FLOX+I3A4 | 19.437 | 19.740 | 19.589 | 27.352 | 26.992 | 27.172 | 0.005214 | 17.111985 |

|  | **β-actin (reference gene)** | | | **Cldn2 (target gene)** | | |  |  |
| --- | --- | --- | --- | --- | --- | --- | --- | --- |
|  | **Ct_1_** | **Ct_2_** | **Mean Ct** | **Ct_1_** | **Ct_2_** | **Mean Ct** | **RE** | **NOR** |
| CKO1 | 24.165 | 23.592 | 23.879 | 27.149 | 27.740 | 27.444 | 0.084468 | 1.388962 |
| CKO2 | 19.434 | 19.142 | 19.288 | 23.324 | 23.330 | 23.327 | 0.060814 | 1.000000 |
| CKO3 | 21.756 | 21.299 | 21.527 | 25.815 | 25.204 | 25.509 | 0.063293 | 1.040774 |
| CKO4 | 21.549 | 21.217 | 21.383 | 24.637 | 25.259 | 24.948 | 0.084491 | 1.389339 |
| CKO+I3A1 | 21.044 | 21.050 | 21.047 | 24.352 | 23.612 | 23.982 | 0.130759 | 2.150154 |
| CKO+I3A2 | 22.191 | 21.699 | 21.945 | 24.442 | 23.704 | 24.073 | 0.228822 | 3.762662 |
| CKO+I3A3 | 21.493 | 21.491 | 21.492 | 24.614 | 24.693 | 24.653 | 0.111752 | 1.837609 |
| CKO+I3A4 | 22.191 | 21.699 | 21.945 | 25.232 | 24.860 | 25.046 | 0.116566 | 1.916775 |
| FLOX+I3A1 | 21.421 | 21.553 | 21.487 | 22.370 | 22.859 | 22.615 | 0.457708 | 7.526391 |
| FLOX+I3A2 | 20.209 | 19.426 | 19.818 | 20.587 | 22.059 | 21.323 | 0.352208 | 5.791585 |
| FLOX+I3A3 | 20.323 | 19.800 | 20.062 | 20.554 | 20.554 | 20.554 | 0.710792 | 11.688017 |
| FLOX+I3A4 | 19.437 | 19.740 | 19.589 | 21.451 | 21.523 | 21.487 | 0.268222 | 4.410549 |

|  | **β-actin (reference gene)** | | | **Cldn3 (target gene)** | | |  |  |
| --- | --- | --- | --- | --- | --- | --- | --- | --- |
|  | **Ct_1_** | **Ct_2_** | **Mean Ct** | **Ct_1_** | **Ct_2_** | **Mean Ct** | **RE** | **NOR** |
| CKO1 | 24.165 | 23.592 | 23.879 | 27.611 | 27.657 | 27.634 | 0.074057 | 2.354829 |
| CKO2 | 19.434 | 19.142 | 19.288 | 23.699 | 22.829 | 23.264 | 0.063538 | 2.020355 |
| CKO3 | 21.756 | 21.299 | 21.527 | 26.097 | 26.940 | 26.518 | 0.031449 | 1.000000 |
| CKO4 | 21.549 | 21.217 | 21.383 | 25.281 | 25.925 | 25.603 | 0.053651 | 1.705989 |
| CKO+I3A1 | 21.044 | 21.050 | 21.047 | 22.888 | 23.287 | 23.088 | 0.243072 | 7.729135 |
| CKO+I3A2 | 22.191 | 21.699 | 21.945 | 24.150 | 24.254 | 24.202 | 0.209199 | 6.652041 |
| CKO+I3A3 | 21.493 | 21.491 | 21.492 | 23.432 | 23.439 | 23.435 | 0.259950 | 8.265816 |
| CKO+I3A4 | 22.191 | 21.699 | 21.945 | 25.393 | 25.000 | 25.197 | 0.105012 | 3.339152 |
| FLOX+I3A1 | 21.421 | 21.553 | 21.487 | 22.856 | 22.856 | 22.856 | 0.387160 | 12.310782 |
| FLOX+I3A2 | 20.209 | 19.426 | 19.818 | 20.173 | 20.224 | 20.199 | 0.767905 | 24.417616 |
| FLOX+I3A3 | 20.323 | 19.800 | 20.062 | 20.979 | 20.773 | 20.876 | 0.568606 | 18.080347 |
| FLOX+I3A4 | 19.437 | 19.740 | 19.589 | 21.045 | 21.230 | 21.138 | 0.341747 | 10.866764 |

|  | **β-actin (reference gene)** | | | **Cldn4 (target gene)** | | |  |  |
| --- | --- | --- | --- | --- | --- | --- | --- | --- |
|  | **Ct_1_** | **Ct_2_** | **Mean Ct** | **Ct_1_** | **Ct_2_** | **Mean Ct** | **RE** | **NOR** |
| CKO1 | 24.165 | 23.592 | 23.879 | 31.337 | 31.898 | 31.617 | 0.004682 | 1.007390 |
| CKO2 | 19.434 | 19.142 | 19.288 | 26.444 | 27.305 | 26.874 | 0.005202 | 1.119442 |
| CKO3 | 21.756 | 21.299 | 21.527 | 29.420 | 29.133 | 29.277 | 0.004647 | 1.000000 |
| CKO4 | 21.549 | 21.217 | 21.383 | 29.220 | 28.809 | 29.015 | 0.005043 | 1.085091 |
| CKO+I3A1 | 21.044 | 21.050 | 21.047 | 26.977 | 27.014 | 26.996 | 0.016192 | 3.484305 |
| CKO+I3A2 | 22.191 | 21.699 | 21.945 | 26.812 | 27.044 | 26.928 | 0.031626 | 6.805296 |
| CKO+I3A3 | 21.493 | 21.491 | 21.492 | 28.334 | 28.440 | 28.387 | 0.008400 | 1.807439 |
| CKO+I3A4 | 22.191 | 21.699 | 21.945 | 26.904 | 27.333 | 27.119 | 0.027708 | 5.962295 |
| FLOX+I3A1 | 21.421 | 21.553 | 21.487 | 25.134 | 25.531 | 25.333 | 0.069565 | 14.968969 |
| FLOX+I3A2 | 20.209 | 19.426 | 19.818 | 23.069 | 22.762 | 22.916 | 0.116791 | 25.131119 |
| FLOX+I3A3 | 20.323 | 19.800 | 20.062 | 24.373 | 23.693 | 24.033 | 0.063747 | 13.717094 |
| FLOX+I3A4 | 19.437 | 19.740 | 19.589 | 23.279 | 23.256 | 23.268 | 0.078075 | 16.800159 |

|  | **β-actin (reference gene)** | | | **Zo-1 (target gene)** | | |  |  |
| --- | --- | --- | --- | --- | --- | --- | --- | --- |
|  | **Ct_1_** | **Ct_2_** | **Mean Ct** | **Ct_1_** | **Ct_2_** | **Mean Ct** | **RE** | **NOR** |
| CKO1 | 24.165 | 23.592 | 23.879 | 29.609 | 28.916 | 29.263 | 0.023946 | 1.000000 |
| CKO2 | 19.434 | 19.142 | 19.288 | 23.778 | 23.736 | 23.757 | 0.045138 | 1.884981 |
| CKO3 | 21.756 | 21.299 | 21.527 | 25.961 | 25.658 | 25.810 | 0.051394 | 2.146229 |
| CKO4 | 21.549 | 21.217 | 21.383 | 26.398 | 25.994 | 26.196 | 0.035571 | 1.485458 |
| CKO+I3A1 | 21.044 | 21.050 | 21.047 | 23.730 | 24.000 | 23.865 | 0.141805 | 5.921767 |
| CKO+I3A2 | 22.191 | 21.699 | 21.945 | 24.962 | 24.786 | 24.874 | 0.131293 | 5.482787 |
| CKO+I3A3 | 21.493 | 21.491 | 21.492 | 24.708 | 25.263 | 24.986 | 0.088754 | 3.706385 |
| CKO+I3A4 | 22.191 | 21.699 | 21.945 | 26.134 | 26.003 | 26.068 | 0.057395 | 2.396830 |
| FLOX+I3A1 | 21.421 | 21.553 | 21.487 | 22.768 | 22.839 | 22.804 | 0.401508 | 16.766984 |
| FLOX+I3A2 | 20.209 | 19.426 | 19.818 | 21.258 | 20.741 | 21.000 | 0.440740 | 18.405325 |
| FLOX+I3A3 | 20.323 | 19.800 | 20.062 | 22.040 | 22.110 | 22.075 | 0.247672 | 10.342774 |
| FLOX+I3A4 | 19.437 | 19.740 | 19.589 | 21.225 | 21.172 | 21.199 | 0.327598 | 13.680522 |

**Figure 7P**

|  | **β-actin (reference gene)** | | | **Slc7a11 (target gene)** | | |  |  |
| --- | --- | --- | --- | --- | --- | --- | --- | --- |
|  | **Ct_1_** | **Ct_2_** | **Mean Ct** | **Ct_1_** | **Ct_2_** | **Mean Ct** | **RE** | **NOR** |
| CKO1 | 20.960 | 20.450 | 20.705 | 31.463 | 31.710 | 31.587 | 0.000530 | 1.000000 |
| CKO2 | 20.838 | 20.317 | 20.577 | 31.130 | 31.743 | 31.437 | 0.000538 | 1.015094 |
| CKO3 | 21.892 | 21.289 | 21.591 | 32.412 | 31.959 | 32.185 | 0.000647 | 1.220755 |
| CKO4 | 21.438 | 21.007 | 21.223 | 31.266 | 31.007 | 31.136 | 0.001037 | 1.956604 |
| CKO+I3A1 | 20.385 | 20.690 | 20.537 | 29.887 | 29.270 | 29.578 | 0.001898 | 3.581132 |
| CKO+I3A2 | 20.190 | 20.464 | 20.327 | 28.810 | 28.606 | 28.708 | 0.003000 | 5.660377 |
| CKO+I3A3 | 21.880 | 21.297 | 21.588 | 29.697 | 29.973 | 29.835 | 0.003293 | 6.213208 |
| CKO+I3A4 | 20.987 | 20.417 | 20.702 | 27.827 | 29.382 | 28.604 | 0.004180 | 7.886792 |
| FLOX+I3A1 | 21.148 | 21.003 | 21.076 | 26.809 | 26.552 | 26.681 | 0.020546 | 38.766001 |
| FLOX+I3A2 | 21.305 | 21.001 | 21.153 | 27.260 | 27.285 | 27.273 | 0.014383 | 27.137577 |
| FLOX+I3A3 | 21.335 | 21.438 | 21.387 | 27.922 | 27.699 | 27.811 | 0.011646 | 21.973915 |
| FLOX+I3A4 | 21.351 | 20.775 | 21.063 | 27.851 | 27.764 | 27.808 | 0.009326 | 17.596542 |

**Figure 7Q**

|  | **β-actin (reference gene)** | | | **Gpx4 (target gene)** | | |  |  |
| --- | --- | --- | --- | --- | --- | --- | --- | --- |
|  | **Ct_1_** | **Ct_2_** | **Mean Ct** | **Ct_1_** | **Ct_2_** | **Mean Ct** | **RE** | **NOR** |
| CKO1 | 19.554 | 19.512 | 19.533 | 32.639 | 32.125 | 32.382 | 0.000136 | 1.248331 |
| CKO2 | 19.281 | 19.741 | 19.511 | 30.522 | 30.522 | 30.522 | 0.000485 | 4.462957 |
| CKO3 | 17.971 | 18.247 | 18.109 | 32.278 | 30.278 | 31.278 | 0.000109 | 1.000000 |
| CKO4 | 18.649 | 18.700 | 18.675 | 29.366 | 30.357 | 29.862 | 0.000429 | 3.950403 |
| CKO+I3A1 | 24.392 | 24.602 | 24.497 | 34.667 | 34.142 | 34.405 | 0.001041 | 9.589795 |
| CKO+I3A2 | 19.917 | 20.110 | 20.014 | 30.870 | 29.990 | 30.430 | 0.000732 | 6.738839 |
| CKO+I3A3 | 20.420 | 19.683 | 20.052 | 29.060 | 29.485 | 29.273 | 0.001676 | 15.433571 |
| CKO+I3A4 | 25.194 | 25.123 | 25.159 | 35.732 | 35.732 | 35.732 | 0.000656 | 6.043985 |
| FLOX+I3A1 | 21.148 | 21.003 | 21.076 | 28.581 | 28.581 | 28.581 | 0.005503 | 50.685459 |
| FLOX+I3A2 | 21.305 | 21.001 | 21.153 | 28.959 | 28.959 | 28.959 | 0.004468 | 41.155119 |
| FLOX+I3A3 | 21.335 | 21.438 | 21.387 | 29.828 | 29.828 | 29.828 | 0.002876 | 26.492278 |
| FLOX+I3A4 | 21.351 | 20.775 | 21.063 | 29.502 | 29.502 | 29.502 | 0.002881 | 26.538225 |

**Figure 8J**

|  | **β-actin (reference gene)** | | | **OLFM4 (target gene)** | | |  |  |
| --- | --- | --- | --- | --- | --- | --- | --- | --- |
|  | **Ct_1_** | **Ct_2_** | **Mean Ct** | **Ct_1_** | **Ct_2_** | **Mean Ct** | **RE** | **NOR** |
| CON1 | 22.033 | 21.867 | 21.950 | 26.175 | 26.142 | 26.159 | 0.054079 | 10.044420 |
| CON2 | 20.861 | 20.393 | 20.627 | 24.594 | 24.533 | 24.563 | 0.065324 | 12.132992 |
| CON3 | 21.399 | 20.825 | 21.112 | 24.782 | 24.488 | 24.635 | 0.087011 | 16.160955 |
| CON4 | 23.287 | 22.866 | 23.077 | 26.173 | 26.092 | 26.132 | 0.120250 | 22.334560 |
| NEC1 | 24.018 | 23.581 | 23.799 | 28.744 | 28.585 | 28.665 | 0.034309 | 6.372300 |
| NEC2 | 21.405 | 20.991 | 21.198 | 27.039 | 27.239 | 27.139 | 0.016280 | 3.023843 |
| NEC3 | 23.937 | 23.898 | 23.918 | 30.303 | 29.658 | 29.981 | 0.014957 | 2.778079 |
| NEC4 | 21.810 | 21.681 | 21.745 | 29.946 | 28.619 | 29.282 | 0.005384 | 1.000000 |

|  | **β-actin (reference gene)** | | | **ATF4 (target gene)** | | |  |  |
| --- | --- | --- | --- | --- | --- | --- | --- | --- |
|  | **Ct_1_** | **Ct_2_** | **Mean Ct** | **Ct_1_** | **Ct_2_** | **Mean Ct** | **RE** | **NOR** |
| CON1 | 22.033 | 21.867 | 21.950 | 24.552 | 24.307 | 24.429 | 0.179338 | 31.534516 |
| CON2 | 20.861 | 20.393 | 20.627 | 25.250 | 24.082 | 24.666 | 0.060854 | 10.700400 |
| CON3 | 21.399 | 20.825 | 21.112 | 24.516 | 23.827 | 24.171 | 0.119974 | 21.096092 |
| CON4 | 23.287 | 22.866 | 23.077 | 27.161 | 27.868 | 27.515 | 0.046133 | 8.111989 |
| NEC1 | 24.018 | 23.581 | 23.799 | 29.462 | 29.183 | 29.323 | 0.021742 | 3.823092 |
| NEC2 | 21.405 | 20.991 | 21.198 | 25.660 | 25.321 | 25.491 | 0.051025 | 8.972080 |
| NEC3 | 23.937 | 23.898 | 23.918 | 30.612 | 31.088 | 30.850 | 0.008188 | 1.439815 |
| NEC4 | 21.810 | 21.681 | 21.745 | 28.746 | 29.660 | 29.203 | 0.005687 | 1.000000 |

|  | **β-actin (reference gene)** | | | **SLC7A11 (target gene)** | | |  |  |
| --- | --- | --- | --- | --- | --- | --- | --- | --- |
|  | **Ct_1_** | **Ct_2_** | **Mean Ct** | **Ct_1_** | **Ct_2_** | **Mean Ct** | **RE** | **NOR** |
| CON1 | 22.033 | 21.867 | 21.950 | 22.152 | 22.515 | 22.333 | 0.766688 | 28.138809 |
| CON2 | 20.861 | 20.393 | 20.627 | 21.356 | 21.338 | 21.347 | 0.607161 | 22.283874 |
| CON3 | 21.399 | 20.825 | 21.112 | 20.062 | 20.423 | 20.243 | 1.827269 | 67.064005 |
| CON4 | 23.287 | 22.866 | 23.077 | 21.829 | 21.905 | 21.867 | 2.312403 | 84.869266 |
| NEC1 | 24.018 | 23.581 | 23.799 | 27.748 | 27.881 | 27.815 | 0.061831 | 2.269318 |
| NEC2 | 21.405 | 20.991 | 21.198 | 25.881 | 25.869 | 25.875 | 0.039099 | 1.435011 |
| NEC3 | 23.937 | 23.898 | 23.918 | 28.870 | 28.861 | 28.865 | 0.032402 | 1.189210 |
| NEC4 | 21.810 | 21.681 | 21.745 | 26.711 | 27.174 | 26.943 | 0.027247 | 1.000000 |

|  | **β-actin (reference gene)** | | | **GPX4 (target gene)** | | |  |  |
| --- | --- | --- | --- | --- | --- | --- | --- | --- |
|  | **Ct_1_** | **Ct_2_** | **Mean Ct** | **Ct_1_** | **Ct_2_** | **Mean Ct** | **RE** | **NOR** |
| CON1 | 22.033 | 21.867 | 21.950 | 25.461 | 25.840 | 25.650 | 0.076934 | 5.299160 |
| CON2 | 20.861 | 20.393 | 20.627 | 24.644 | 25.357 | 25.001 | 0.048241 | 3.322819 |
| CON3 | 21.399 | 20.825 | 21.112 | 24.012 | 24.109 | 24.060 | 0.129575 | 8.924953 |
| CON4 | 23.287 | 22.866 | 23.077 | 26.493 | 26.995 | 26.744 | 0.078693 | 5.420289 |
| NEC1 | 24.018 | 23.581 | 23.799 | 28.004 | 28.912 | 28.458 | 0.039600 | 2.727576 |
| NEC2 | 21.405 | 20.991 | 21.198 | 26.120 | 26.050 | 26.085 | 0.033796 | 2.327853 |
| NEC3 | 23.937 | 23.898 | 23.918 | 28.607 | 28.194 | 28.401 | 0.044724 | 3.080526 |
| NEC4 | 21.810 | 21.681 | 21.745 | 27.477 | 28.225 | 27.851 | 0.014518 | 1.000000 |

**Figure S4H**

|  | **β-actin (reference gene)** | | | **S100a8 (target gene)** | | |  |  |
| --- | --- | --- | --- | --- | --- | --- | --- | --- |
|  | **Ct_1_** | **Ct_2_** | **Mean Ct** | **Ct_1_** | **Ct_2_** | **Mean Ct** | **RE** | **NOR** |
| PBS1 | 22.396 | 22.459 | 22.427 | 28.709 | 29.540 | 29.125 | 0.009635 | 1.502051 |
| PBS2 | 23.225 | 23.043 | 23.134 | 29.571 | 28.852 | 29.211 | 0.014809 | 2.308715 |
| PBS3 | 21.842 | 21.870 | 21.856 | 28.637 | 29.645 | 29.141 | 0.006415 | 1.000000 |
| PBS4 | 22.383 | 22.918 | 22.650 | 28.426 | 29.706 | 29.066 | 0.011712 | 1.825786 |
| Probiotics1 | 19.853 | 20.163 | 20.008 | 25.852 | 24.717 | 25.285 | 0.025800 | 4.022094 |
| Probiotics2 | 21.857 | 21.063 | 21.460 | 25.076 | 25.647 | 25.362 | 0.066913 | 10.431450 |
| Probiotics3 | 21.300 | 21.244 | 21.272 | 26.374 | 26.374 | 26.374 | 0.029109 | 4.538014 |
| Probiotics4 | 22.356 | 22.356 | 22.356 | 26.033 | 26.033 | 26.033 | 0.078206 | 12.191900 |

|  | **β-actin (reference gene)** | | | **S100a9 (target gene)** | | |  |  |
| --- | --- | --- | --- | --- | --- | --- | --- | --- |
|  | **Ct_1_** | **Ct_2_** | **Mean Ct** | **Ct_1_** | **Ct_2_** | **Mean Ct** | **RE** | **NOR** |
| PBS1 | 22.396 | 22.459 | 22.427 | 26.226 | 25.938 | 26.082 | 0.079381 | 1.417949 |
| PBS2 | 23.225 | 23.043 | 23.134 | 26.285 | 26.785 | 26.535 | 0.094660 | 1.690880 |
| PBS3 | 21.842 | 21.870 | 21.856 | 26.489 | 25.541 | 26.015 | 0.055983 | 1.000000 |
| PBS4 | 22.383 | 22.918 | 22.650 | 25.536 | 26.287 | 25.911 | 0.104317 | 1.863364 |
| Probiotics1 | 19.853 | 20.163 | 20.008 | 23.050 | 23.123 | 23.087 | 0.118379 | 2.114563 |
| Probiotics2 | 21.857 | 21.063 | 21.460 | 23.524 | 24.237 | 23.880 | 0.186814 | 3.336986 |
| Probiotics3 | 21.300 | 21.244 | 21.272 | 24.448 | 24.448 | 24.448 | 0.110689 | 1.977200 |
| Probiotics4 | 22.356 | 22.356 | 22.356 | 24.385 | 24.698 | 24.542 | 0.219774 | 3.925731 |

|  | **β-actin (reference gene)** | | | **Arg1 (target gene)** | | |  |  |
| --- | --- | --- | --- | --- | --- | --- | --- | --- |
|  | **Ct_1_** | **Ct_2_** | **Mean Ct** | **Ct_1_** | **Ct_2_** | **Mean Ct** | **RE** | **NOR** |
| PBS1 | 22.396 | 22.459 | 22.427 | 28.591 | 28.757 | 28.674 | 0.013170 | 1.000000 |
| PBS2 | 23.225 | 23.043 | 23.134 | 29.020 | 28.029 | 28.525 | 0.023834 | 1.809659 |
| PBS3 | 21.842 | 21.870 | 21.856 | 28.941 | 27.239 | 28.090 | 0.013287 | 1.008887 |
| PBS4 | 22.383 | 22.918 | 22.650 | 28.223 | 28.091 | 28.157 | 0.021995 | 1.670061 |
| Probiotics1 | 19.853 | 20.163 | 20.008 | 24.713 | 25.456 | 25.084 | 0.029644 | 2.250828 |
| Probiotics2 | 21.857 | 21.063 | 21.460 | 24.851 | 25.298 | 25.074 | 0.081656 | 6.200022 |
| Probiotics3 | 21.300 | 21.244 | 21.272 | 25.221 | 25.036 | 25.129 | 0.069035 | 5.241700 |
| Probiotics4 | 22.356 | 22.356 | 22.356 | 26.621 | 25.036 | 25.829 | 0.090079 | 6.839512 |

|  | **β-actin (reference gene)** | | | **Cybb (target gene)** | | |  |  |
| --- | --- | --- | --- | --- | --- | --- | --- | --- |
|  | **Ct_1_** | **Ct_2_** | **Mean Ct** | **Ct_1_** | **Ct_2_** | **Mean Ct** | **RE** | **NOR** |
| PBS1 | 22.396 | 22.459 | 22.427 | 26.369 | 26.855 | 26.612 | 0.054996 | 1.000000 |
| PBS2 | 23.225 | 23.043 | 23.134 | 27.562 | 26.411 | 26.986 | 0.069233 | 1.258888 |
| PBS3 | 21.842 | 21.870 | 21.856 | 25.442 | 25.170 | 25.306 | 0.091542 | 1.664530 |
| PBS4 | 22.383 | 22.918 | 22.650 | 25.853 | 26.401 | 26.127 | 0.089836 | 1.633523 |
| Probiotics1 | 19.853 | 20.163 | 20.008 | 22.117 | 23.670 | 22.893 | 0.135362 | 2.461331 |
| Probiotics2 | 21.857 | 21.063 | 21.460 | 23.199 | 25.024 | 24.112 | 0.159105 | 2.893049 |
| Probiotics3 | 21.300 | 21.244 | 21.272 | 26.471 | 22.819 | 24.645 | 0.096551 | 1.755615 |
| Probiotics4 | 22.356 | 22.356 | 22.356 | 25.551 | 25.484 | 25.517 | 0.111760 | 2.032174 |

**Figure S5F**

|  | **β-actin (reference gene)** | | | **Occludin (target gene)** | | |  |  |
| --- | --- | --- | --- | --- | --- | --- | --- | --- |
|  | **Ct_1_** | **Ct_2_** | **Mean Ct** | **Ct_1_** | **Ct_2_** | **Mean Ct** | **RE** | **NOR** |
| IL18+/+1 | 19.920 | 19.867 | 19.894 | 28.284 | 28.313 | 28.299 | 0.002950 | 6.901931 |
| IL18+/+2 | 20.325 | 20.499 | 20.412 | 27.567 | 27.455 | 27.511 | 0.007294 | 17.065372 |
| IL18+/+3 | 22.513 | 22.259 | 22.386 | 30.207 | 29.501 | 29.854 | 0.005648 | 13.214043 |
| IL18+/+4 | 20.932 | 21.302 | 21.117 | 28.776 | 28.776 | 28.776 | 0.004948 | 11.575479 |
| IL18-/-1 | 23.976 | 24.396 | 24.186 | 34.569 | 34.569 | 34.569 | 0.000749 | 1.751997 |
| IL18-/-2 | 24.685 | 24.619 | 24.652 | 34.598 | 34.598 | 34.598 | 0.001014 | 2.371829 |
| IL18-/-3 | 24.910 | 24.910 | 24.910 | 35.272 | 35.272 | 35.272 | 0.000760 | 1.777685 |
| IL18-/-4 | 24.404 | 24.488 | 24.446 | 35.638 | 35.638 | 35.638 | 0.000427 | 1.000000 |
| iPMN-MDSCs-1 | 22.184 | 22.904 | 22.544 | 30.541 | 31.929 | 31.235 | 0.002420 | 5.660879 |
| iPMN-MDSCs-2 | 21.299 | 21.739 | 21.519 | 29.525 | 30.243 | 29.884 | 0.003032 | 7.094282 |
| iPMN-MDSCs-3 | 20.896 | 20.289 | 20.593 | 28.543 | 28.414 | 28.479 | 0.004228 | 9.891498 |
| iPMN-MDSCs-4 | 19.706 | 19.384 | 19.545 | 27.529 | 28.931 | 28.230 | 0.002430 | 5.685360 |

|  | **β-actin (reference gene)** | | | **Cldn2 (target gene)** | | |  |  |
| --- | --- | --- | --- | --- | --- | --- | --- | --- |
|  | **Ct_1_** | **Ct_2_** | **Mean Ct** | **Ct_1_** | **Ct_2_** | **Mean Ct** | **RE** | **NOR** |
| IL18+/+1 | 20.325 | 20.499 | 20.412 | 25.240 | 25.588 | 25.414 | 0.031207 | 3.458149 |
| IL18+/+2 | 22.513 | 22.259 | 22.386 | 25.948 | 25.948 | 25.948 | 0.084670 | 9.382680 |
| IL18+/+3 | 21.401 | 21.185 | 21.293 | 25.328 | 25.328 | 25.328 | 0.061002 | 6.759891 |
| IL18+/+4 | 20.932 | 21.302 | 21.117 | 25.554 | 25.531 | 25.543 | 0.046536 | 5.156885 |
| IL18-/-1 | 24.976 | 24.396 | 24.686 | 31.628 | 31.328 | 31.478 | 0.009024 | 1.000000 |
| IL18-/-2 | 24.685 | 24.619 | 24.652 | 30.667 | 30.887 | 30.777 | 0.014328 | 1.587768 |
| IL18-/-3 | 24.910 | 24.910 | 24.910 | 30.908 | 31.368 | 31.138 | 0.013341 | 1.478362 |
| IL18-/-4 | 24.404 | 24.488 | 24.446 | 31.182 | 31.182 | 31.182 | 0.009381 | 1.039579 |
| iPMN-MDSCs-1 | 22.184 | 22.904 | 22.544 | 25.004 | 25.254 | 25.129 | 0.166603 | 18.461950 |
| iPMN-MDSCs-2 | 21.299 | 21.739 | 21.519 | 25.572 | 25.786 | 25.679 | 0.055936 | 6.198475 |
| iPMN-MDSCs-3 | 20.896 | 20.289 | 20.593 | 23.525 | 22.944 | 23.235 | 0.160225 | 17.755196 |
| iPMN-MDSCs-4 | 19.706 | 19.384 | 19.545 | 22.752 | 22.453 | 22.603 | 0.120098 | 13.308587 |

|  | **β-actin (reference gene)** | | | **Cldn3 (target gene)** | | |  |  |
| --- | --- | --- | --- | --- | --- | --- | --- | --- |
|  | **Ct_1_** | **Ct_2_** | **Mean Ct** | **Ct_1_** | **Ct_2_** | **Mean Ct** | **RE** | **NOR** |
| IL18+/+1 | 19.920 | 19.867 | 19.894 | 20.865 | 20.865 | 20.865 | 0.509976 | 3.232163 |
| IL18+/+2 | 20.325 | 20.499 | 20.412 | 21.923 | 21.637 | 21.780 | 0.387428 | 2.455471 |
| IL18+/+3 | 21.401 | 21.185 | 21.293 | 22.560 | 22.499 | 22.530 | 0.424401 | 2.689802 |
| IL18+/+4 | 20.932 | 21.302 | 21.117 | 22.285 | 22.120 | 22.203 | 0.471229 | 2.986592 |
| IL18-/-1 | 24.976 | 24.396 | 24.686 | 25.973 | 26.901 | 26.437 | 0.297096 | 1.882957 |
| IL18-/-2 | 24.685 | 24.619 | 24.652 | 27.437 | 26.749 | 27.093 | 0.184156 | 1.167158 |
| IL18-/-3 | 24.910 | 25.418 | 25.164 | 27.273 | 27.359 | 27.316 | 0.225000 | 1.426026 |
| IL18-/-4 | 24.404 | 24.488 | 24.446 | 27.489 | 26.731 | 27.110 | 0.157782 | 1.000000 |
| iPMN-MDSCs-1 | 22.184 | 22.904 | 22.544 | 24.256 | 23.575 | 23.916 | 0.386441 | 2.449217 |
| iPMN-MDSCs-2 | 21.299 | 21.739 | 21.519 | 23.576 | 23.068 | 23.322 | 0.286513 | 1.815887 |
| iPMN-MDSCs-3 | 20.896 | 20.289 | 20.593 | 22.527 | 21.527 | 22.027 | 0.369967 | 2.344808 |
| iPMN-MDSCs-4 | 19.706 | 19.384 | 19.545 | 21.455 | 21.200 | 21.327 | 0.290733 | 1.842633 |

|  | **β-actin (reference gene)** | | | **Cldn4 (target gene)** | | |  |  |
| --- | --- | --- | --- | --- | --- | --- | --- | --- |
|  | **Ct_1_** | **Ct_2_** | **Mean Ct** | **Ct_1_** | **Ct_2_** | **Mean Ct** | **RE** | **NOR** |
| IL18+/+1 | 19.920 | 19.867 | 19.894 | 25.382 | 26.080 | 25.731 | 0.017488 | 2.455471 |
| IL18+/+2 | 22.513 | 22.259 | 22.386 | 27.281 | 27.497 | 27.389 | 0.031185 | 4.378692 |
| IL18+/+3 | 21.401 | 21.185 | 21.293 | 26.446 | 26.797 | 26.622 | 0.024886 | 3.494292 |
| IL18+/+4 | 20.932 | 21.302 | 21.117 | 27.283 | 26.525 | 26.904 | 0.018111 | 2.542945 |
| IL18-/-1 | 24.976 | 24.396 | 24.686 | 31.451 | 30.951 | 31.201 | 0.010934 | 1.535278 |
| IL18-/-2 | 24.685 | 24.619 | 24.652 | 31.648 | 31.347 | 31.498 | 0.008696 | 1.220947 |
| IL18-/-3 | 24.910 | 24.910 | 24.910 | 32.437 | 31.650 | 32.044 | 0.007122 | 1.000000 |
| IL18-/-4 | 24.404 | 24.488 | 24.446 | 31.089 | 30.907 | 30.998 | 0.010657 | 1.496404 |
| iPMN-MDSCs-1 | 22.184 | 22.904 | 22.544 | 27.414 | 27.543 | 27.478 | 0.032701 | 4.591545 |
| iPMN-MDSCs-2 | 21.299 | 21.739 | 21.519 | 25.763 | 25.855 | 25.809 | 0.051123 | 7.178176 |
| iPMN-MDSCs-3 | 20.896 | 20.289 | 20.593 | 25.416 | 24.702 | 25.059 | 0.045243 | 6.352552 |
| iPMN-MDSCs-4 | 19.706 | 19.384 | 19.545 | 25.573 | 25.042 | 25.308 | 0.018418 | 2.586129 |

|  | **β-actin (reference gene)** | | | **Zo-1 (target gene)** | | |  |  |
| --- | --- | --- | --- | --- | --- | --- | --- | --- |
|  | **Ct_1_** | **Ct_2_** | **Mean Ct** | **Ct_1_** | **Ct_2_** | **Mean Ct** | **RE** | **NOR** |
| IL18+/+1 | 20.325 | 20.499 | 20.412 | 25.535 | 25.643 | 25.589 | 0.027642 | 4.877020 |
| IL18+/+2 | 22.630 | 22.397 | 22.514 | 28.028 | 28.680 | 28.354 | 0.017452 | 3.079081 |
| IL18+/+3 | 21.401 | 21.185 | 21.293 | 26.337 | 26.377 | 26.357 | 0.029894 | 5.274374 |
| IL18+/+4 | 20.932 | 20.932 | 20.932 | 25.898 | 25.898 | 25.898 | 0.031995 | 5.645103 |
| IL18-/-1 | 25.117 | 25.229 | 25.173 | 32.636 | 32.636 | 32.636 | 0.005668 | 1.000000 |
| IL18-/-2 | 24.362 | 25.011 | 24.687 | 31.468 | 31.468 | 31.468 | 0.009090 | 1.603806 |
| IL18-/-3 | 23.394 | 23.917 | 23.656 | 30.452 | 30.452 | 30.452 | 0.008996 | 1.587218 |
| IL18-/-4 | 24.356 | 25.226 | 24.791 | 31.250 | 31.250 | 31.250 | 0.011367 | 2.005553 |
| iPMN-MDSCs-1 | 22.184 | 22.904 | 22.544 | 26.752 | 26.543 | 26.648 | 0.058158 | 10.261199 |
| iPMN-MDSCs-2 | 21.299 | 21.739 | 21.519 | 25.176 | 25.424 | 25.300 | 0.072737 | 12.833487 |
| iPMN-MDSCs-3 | 20.896 | 20.289 | 20.593 | 25.743 | 25.542 | 25.642 | 0.030196 | 5.327727 |
| iPMN-MDSCs-4 | 19.706 | 19.384 | 19.545 | 22.837 | 23.544 | 23.190 | 0.079902 | 14.097611 |

**Figure S5N**

| **iPMN-MDSCs** | | | | | | | | |
| --- | --- | --- | --- | --- | --- | --- | --- | --- |
|  | **β-actin (reference gene)** | | | **Il18 (target gene)** | | |  |  |
|  | **Ct_1_** | **Ct_2_** | **Mean Ct** | **Ct_1_** | **Ct_2_** | **Mean Ct** | **RE** | **NOR** |
| IL18+/+1 | 21.472 | 21.782 | 21.627 | 26.808 | 26.008 | 26.408 | 0.036373 | 56.336577 |
| IL18+/+2 | 21.823 | 22.037 | 21.930 | 26.548 | 26.031 | 26.290 | 0.048715 | 75.452667 |
| IL18+/+3 | 21.723 | 21.723 | 21.723 | 26.209 | 26.809 | 26.509 | 0.036247 | 56.141667 |
| IL18+/+4 | 21.935 | 21.590 | 21.763 | 26.142 | 25.756 | 25.949 | 0.054921 | 85.065368 |
| IL18-/-1 | 23.684 | 23.840 | 23.762 | 34.265 | 34.265 | 34.265 | 0.000689 | 1.067325 |
| IL18-/-2 | 24.527 | 24.631 | 24.579 | 35.176 | 35.176 | 35.176 | 0.000646 | 1.000000 |
| IL18-/-3 | 24.446 | 24.347 | 24.397 | 34.974 | 34.974 | 34.974 | 0.000654 | 1.013608 |
| IL18-/-4 | 23.817 | 23.942 | 23.880 | 33.949 | 33.949 | 33.949 | 0.000931 | 1.441429 |
| iPMN-MDSCs-1 | 22.525 | 20.981 | 21.753 | 27.052 | 26.810 | 26.931 | 0.027623 | 42.784016 |
| iPMN-MDSCs-2 | 20.232 | 20.587 | 20.410 | 26.524 | 25.954 | 26.239 | 0.017585 | 27.237077 |
| iPMN-MDSCs-3 | 21.525 | 21.225 | 21.375 | 27.254 | 27.841 | 27.548 | 0.013864 | 21.473717 |
| iPMN-MDSCs-4 | 21.257 | 21.456 | 21.357 | 28.054 | 27.874 | 27.964 | 0.010255 | 15.883974 |

**Figure S6G**

| **Spleen** | | | | | | | | |
| --- | --- | --- | --- | --- | --- | --- | --- | --- |
|  | **β-actin (reference gene)** | | | **Olfm4 (target gene)** | | |  |  |
|  | **Ct_1_** | **Ct_2_** | **Mean Ct** | **Ct_1_** | **Ct_2_** | **Mean Ct** | **RE** | **NOR** |
| FLOX1 | 19.729 | 19.569 | 19.649 | 22.630 | 21.905 | 22.268 | 0.162837 | 81.008422 |
| FLOX2 | 19.778 | 19.746 | 19.762 | 22.453 | 22.469 | 22.461 | 0.154000 | 76.612081 |
| FLOX3 | 20.122 | 19.975 | 20.049 | 22.878 | 22.893 | 22.886 | 0.139952 | 69.623382 |
| FLOX4 | 20.022 | 19.742 | 19.882 | 22.846 | 22.624 | 22.735 | 0.138408 | 68.855500 |
| CKO1 | 18.702 | 18.512 | 18.607 | 27.469 | 27.662 | 27.566 | 0.002010 | 1.000000 |
| CKO2 | 19.384 | 19.793 | 19.589 | 25.899 | 26.078 | 25.989 | 0.011842 | 5.890949 |
| CKO3 | 21.883 | 21.711 | 21.797 | 28.504 | 28.622 | 28.563 | 0.009188 | 4.570969 |
| CKO4 | 18.149 | 18.349 | 18.249 | 26.554 | 25.830 | 26.192 | 0.004064 | 2.021603 |

| **LPMCs** | | | | | | | | |
| --- | --- | --- | --- | --- | --- | --- | --- | --- |
|  | **β-actin (reference gene)** | | | **Olfm4 (target gene)** | | |  |  |
|  | **Ct_1_** | **Ct_2_** | **Mean Ct** | **Ct_1_** | **Ct_2_** | **Mean Ct** | **RE** | **NOR** |
| FLOX1 | 21.559 | 21.161 | 21.360 | 22.989 | 22.759 | 22.874 | 0.350139 | 134.456902 |
| FLOX2 | 20.379 | 20.476 | 20.428 | 22.590 | 22.528 | 22.559 | 0.228220 | 87.638932 |
| FLOX3 | 20.526 | 20.478 | 20.502 | 22.450 | 22.379 | 22.415 | 0.265632 | 102.005279 |
| FLOX4 | 20.565 | 20.681 | 20.623 | 22.232 | 22.315 | 22.274 | 0.318530 | 122.318601 |
| CKO1 | 23.306 | 23.304 | 23.305 | 30.959 | 30.730 | 30.845 | 0.005375 | 2.064082 |
| CKO2 | 23.254 | 23.488 | 23.371 | 30.303 | 30.303 | 30.303 | 0.008190 | 3.144869 |
| CKO3 | 20.611 | 19.905 | 20.258 | 28.102 | 28.251 | 28.177 | 0.004133 | 1.587218 |
| CKO4 | 22.594 | 22.369 | 22.482 | 30.720 | 31.413 | 31.067 | 0.002604 | 1.000000 |

**Figure S6O**

| **Spleen** | | | | | | | | |
| --- | --- | --- | --- | --- | --- | --- | --- | --- |
|  | **β-actin (reference gene)** | | | **Olfm4 (target gene)** | | |  |  |
|  | **Ct_1_** | **Ct_2_** | **Mean Ct** | **Ct_1_** | **Ct_2_** | **Mean Ct** | **RE** | **NOR** |
| FLOX1 | 20.57 | 20.44 | 20.51 | 28.95 | 28.58 | 28.76 | 0.0033 | 28.5563 |
| FLOX2 | 18.17 | 17.99 | 18.08 | 25.79 | 25.32 | 25.55 | 0.0056 | 50.7325 |
| FLOX3 | 17.16 | 16.98 | 17.07 | 24.76 | 25.08 | 24.92 | 0.0043 | 37.7716 |
| FLOX4 | 17.65 | 17.97 | 17.81 | 24.83 | 25.61 | 25.22 | 0.0059 | 52.3342 |
| CKO1 | 21.35 | 21.34 | 21.35 | 34.99 | 33.90 | 34.44 | 0.0001 | 1.0000 |
| CKO2 | 20.32 | 20.07 | 20.20 | 33.04 | 32.63 | 32.84 | 0.0002 | 1.4365 |
| CKO3 | 21.67 | 21.66 | 21.66 | 33.44 | 33.97 | 33.70 | 0.0002 | 2.2847 |
| CKO4 | 20.53 | 20.75 | 20.64 | 32.92 | 32.48 | 32.70 | 0.0002 | 2.0722 |

| **LPMCs** | | | | | | | | |
| --- | --- | --- | --- | --- | --- | --- | --- | --- |
|  | **β-actin (reference gene)** | | | **Olfm4 (target gene)** | | |  |  |
|  | **Ct_1_** | **Ct_2_** | **Mean Ct** | **Ct_1_** | **Ct_2_** | **Mean Ct** | **RE** | **NOR** |
| FLOX1 | 19.66 | 19.31 | 19.49 | 21.87 | 22.41 | 22.14 | 0.1593 | 169.4834 |
| FLOX2 | 18.31 | 18.10 | 18.20 | 20.69 | 20.68 | 20.68 | 0.1794 | 190.8771 |
| FLOX3 | 18.49 | 18.49 | 18.49 | 20.69 | 20.68 | 20.68 | 0.2185 | 232.4054 |
| FLOX4 | 18.49 | 18.49 | 18.49 | 20.83 | 21.31 | 21.07 | 0.1668 | 177.4169 |
| CKO1 | 22.00 | 22.09 | 22.05 | 29.82 | 29.53 | 29.67 | 0.0051 | 5.3796 |
| CKO2 | 19.89 | 20.00 | 19.94 | 29.35 | 29.94 | 29.64 | 0.0012 | 1.2790 |
| CKO3 | 19.36 | 20.14 | 19.75 | 28.76 | 28.80 | 28.78 | 0.0019 | 2.0399 |
| CKO4 | 20.81 | 20.94 | 20.88 | 30.89 | 30.98 | 30.93 | 0.0009 | 1.0000 |

**Figure S9F**

|  | **β-actin (reference gene)** | | | **Il18 (target gene)** | | |  |  |
| --- | --- | --- | --- | --- | --- | --- | --- | --- |
|  | **Ct_1_** | **Ct_2_** | **Mean Ct** | **Ct_1_** | **Ct_2_** | **Mean Ct** | **RE** | **NOR** |
| FLOX1 | 19.738 | 19.235 | 19.486 | 22.671 | 23.341 | 23.006 | 0.087194 | 4.775875 |
| FLOX2 | 19.370 | 19.559 | 19.464 | 23.367 | 24.430 | 23.899 | 0.046253 | 2.533442 |
| FLOX3 | 20.865 | 20.525 | 20.695 | 24.144 | 24.425 | 24.284 | 0.083076 | 4.550352 |
| FLOX4 | 20.995 | 20.849 | 20.922 | 24.315 | 24.139 | 24.227 | 0.101214 | 5.543816 |
| CKO1 | 20.409 | 20.779 | 20.594 | 26.489 | 26.023 | 26.256 | 0.019757 | 1.082135 |
| CKO2 | 17.160 | 16.436 | 16.798 | 21.730 | 21.853 | 21.791 | 0.031392 | 1.719440 |
| CKO3 | 17.541 | 17.102 | 17.321 | 23.121 | 23.072 | 23.097 | 0.018257 | 1.000000 |
| CKO4 | 18.677 | 18.665 | 18.671 | 23.541 | 24.635 | 24.088 | 0.023397 | 1.281522 |

**Figure 12I**

|  | **β-actin (reference gene)** | | | **Il18 (target gene)** | | |  |  |
| --- | --- | --- | --- | --- | --- | --- | --- | --- |
|  | **Ct_1_** | **Ct_2_** | **Mean Ct** | **Ct_1_** | **Ct_2_** | **Mean Ct** | **RE** | **NOR** |
| Vehicle1 | 20.923 | 20.291 | 20.607 | 25.205 | 25.586 | 25.395 | 0.036183 | 2.741716 |
| Vehicle2 | 19.224 | 19.754 | 19.489 | 24.106 | 24.472 | 24.289 | 0.035900 | 2.720264 |
| Vehicle3 | 20.759 | 20.094 | 20.426 | 25.374 | 25.771 | 25.572 | 0.028236 | 2.139551 |
| Vehicle4 | 20.765 | 20.902 | 20.834 | 26.373 | 26.022 | 26.198 | 0.024278 | 1.839655 |
| ISRIB1 | 21.617 | 21.820 | 21.719 | 27.925 | 27.491 | 27.708 | 0.015741 | 1.192741 |
| ISRIB2 | 19.267 | 19.948 | 19.607 | 25.887 | 25.815 | 25.851 | 0.013197 | 1.000000 |
| ISRIB3 | 19.859 | 19.875 | 19.867 | 24.984 | 25.502 | 25.243 | 0.024081 | 1.824664 |
| ISRIB4 | 19.936 | 19.729 | 19.832 | 25.997 | 25.291 | 25.644 | 0.017800 | 1.348772 |

**Figure S13G**

| **Spleen** | | | | | | | | |
| --- | --- | --- | --- | --- | --- | --- | --- | --- |
|  | **β-actin (reference gene)** | | | **Gpx4 (target gene)** | | |  |  |
|  | **Ct_1_** | **Ct_2_** | **Mean Ct** | **Ct_1_** | **Ct_2_** | **Mean Ct** | **RE** | **NOR** |
| FLOX1 | 20.104 | 19.652 | 19.878 | 23.851 | 23.993 | 23.922 | 0.060623 | 37.798571 |
| FLOX2 | 20.465 | 19.889 | 20.177 | 24.225 | 24.489 | 24.357 | 0.055169 | 34.398167 |
| FLOX3 | 17.921 | 18.237 | 18.079 | 22.259 | 22.071 | 22.165 | 0.058883 | 36.714036 |
| FLOX4 | 18.828 | 18.429 | 18.629 | 22.380 | 22.589 | 22.485 | 0.069060 | 43.059504 |
| CKO1 | 21.492 | 21.593 | 21.543 | 30.418 | 29.768 | 30.093 | 0.002667 | 1.663065 |
| CKO2 | 23.670 | 23.763 | 23.716 | 31.947 | 31.576 | 31.761 | 0.003787 | 2.361275 |
| CKO3 | 21.471 | 22.236 | 21.854 | 31.378 | 30.898 | 31.138 | 0.001604 | 1.000000 |
| CKO4 | 24.072 | 24.026 | 24.049 | 31.947 | 31.576 | 31.761 | 0.004768 | 2.972843 |

| **LPMCs** | | | | | | | | |
| --- | --- | --- | --- | --- | --- | --- | --- | --- |
|  | **β-actin (reference gene)** | | | **Gpx4 (target gene)** | | |  |  |
|  | **Ct_1_** | **Ct_2_** | **Mean Ct** | **Ct_1_** | **Ct_2_** | **Mean Ct** | **RE** | **NOR** |
| FLOX1 | 21.793 | 22.382 | 22.088 | 26.233 | 26.233 | 26.233 | 0.056504 | 82.911482 |
| FLOX2 | 20.810 | 21.157 | 20.984 | 26.240 | 25.542 | 25.891 | 0.033319 | 48.891101 |
| FLOX3 | 21.272 | 21.795 | 21.534 | 25.812 | 25.577 | 25.695 | 0.055900 | 82.025467 |
| FLOX4 | 21.591 | 21.543 | 21.567 | 25.847 | 25.797 | 25.822 | 0.052374 | 76.851419 |
| CKO1 | 22.141 | 22.141 | 22.141 | 32.503 | 32.817 | 32.660 | 0.000681 | 1.000000 |
| CKO2 | 21.471 | 21.344 | 21.408 | 30.610 | 30.610 | 30.610 | 0.001697 | 2.490612 |
| CKO3 | 24.545 | 24.545 | 24.545 | 32.985 | 32.970 | 32.978 | 0.002894 | 4.247165 |
| CKO4 | 22.509 | 22.340 | 22.425 | 31.335 | 31.335 | 31.335 | 0.002078 | 3.049346 |

**Figure S13Q**

| **Spleen** | | | | | | | | |
| --- | --- | --- | --- | --- | --- | --- | --- | --- |
|  | **β-actin (reference gene)** | | | **Gpx4 (target gene)** | | |  |  |
|  | **Ct_1_** | **Ct_2_** | **Mean Ct** | **Ct_1_** | **Ct_2_** | **Mean Ct** | **RE** | **NOR** |
| FLOX1 | 20.44 | 20.34 | 20.39 | 27.08 | 26.81 | 26.95 | 0.0106 | 23.6695 |
| FLOX2 | 19.24 | 19.18 | 19.21 | 24.59 | 24.41 | 24.50 | 0.0256 | 56.3122 |
| FLOX3 | 19.73 | 19.80 | 19.77 | 26.22 | 26.04 | 26.13 | 0.0121 | 26.5880 |
| FLOX4 | 19.91 | 19.73 | 19.82 | 25.88 | 26.70 | 26.29 | 0.0113 | 24.4980 |
| CKO1 | 22.64 | 22.43 | 22.53 | 32.44 | 32.86 | 32.65 | 0.0009 | 1.1164 |
| CKO2 | 21.33 | 21.99 | 21.66 | 32.95 | 32.57 | 32.76 | 0.0005 | 1.0000 |
| CKO3 | 23.34 | 23.52 | 23.43 | 32.99 | 32.96 | 32.98 | 0.0013 | 2.0100 |
| CKO4 | 22.28 | 22.27 | 22.27 | 31.80 | 31.82 | 31.81 | 0.0013 | 2.9654 |

| **LPMCs** | | | | | | | | |
| --- | --- | --- | --- | --- | --- | --- | --- | --- |
|  | **β-actin (reference gene)** | | | **Gpx4 (target gene)** | | |  |  |
|  | **Ct_1_** | **Ct_2_** | **Mean Ct** | **Ct_1_** | **Ct_2_** | **Mean Ct** | **RE** | **NOR** |
| FLOX1 | 21.53 | 21.66 | 21.60 | 26.51 | 26.51 | 26.51 | 0.0332 | 446.0309 |
| FLOX2 | 20.70 | 20.74 | 20.72 | 25.46 | 25.47 | 25.46 | 0.0372 | 499.7283 |
| FLOX3 | 21.62 | 21.21 | 21.42 | 26.93 | 28.79 | 27.86 | 0.0115 | 207.6486 |
| FLOX4 | 20.49 | 20.44 | 20.47 | 25.54 | 25.38 | 25.46 | 0.0314 | 421.5325 |
| CKO1 | 18.86 | 19.25 | 19.05 | 32.98 | 32.55 | 32.77 | 0.0001 | 1.0000 |
| CKO2 | 18.09 | 18.10 | 18.09 | 29.91 | 30.85 | 30.38 | 0.0002 | 2.6870 |
| CKO3 | 19.38 | 19.44 | 19.41 | 31.96 | 32.35 | 32.15 | 0.0001 | 1.9616 |
| CKO4 | 18.22 | 18.09 | 18.16 | 31.41 | 31.55 | 31.48 | 0.0001 | 1.3315 |

**Figure S17I**

|  | **β-actin (reference gene)** | | | **Il18 (target gene)** | | |  |  |
| --- | --- | --- | --- | --- | --- | --- | --- | --- |
|  | **Ct_1_** | **Ct_2_** | **Mean Ct** | **Ct_1_** | **Ct_2_** | **Mean Ct** | **RE** | **NOR** |
| PBS1 | 20.909 | 20.216 | 20.563 | 25.379 | 25.919 | 25.649 | 0.029438 | 1.600746 |
| PBS2 | 20.662 | 20.625 | 20.644 | 25.490 | 25.442 | 25.466 | 0.035342 | 1.921740 |
| PBS3 | 20.884 | 20.884 | 20.884 | 26.037 | 25.727 | 25.882 | 0.031293 | 1.701605 |
| PBS4 | 19.621 | 19.921 | 19.771 | 25.997 | 25.075 | 25.536 | 0.018390 | 1.000000 |
| I3A1 | 18.838 | 19.260 | 19.049 | 22.605 | 23.116 | 22.860 | 0.071249 | 3.874264 |
| I3A2 | 19.988 | 19.568 | 19.778 | 24.007 | 23.741 | 23.874 | 0.058488 | 3.180355 |
| I3A3 | 19.952 | 19.761 | 19.857 | 23.913 | 23.055 | 23.484 | 0.080912 | 4.399699 |
| I3A4 | 19.541 | 19.703 | 19.622 | 24.102 | 23.982 | 24.042 | 0.046714 | 2.540135 |

**Figure S17K**

|  | **β-actin (reference gene)** | | | **Ahr (target gene)** | | |  |  |
| --- | --- | --- | --- | --- | --- | --- | --- | --- |
|  | **Ct_1_** | **Ct_2_** | **Mean Ct** | **Ct_1_** | **Ct_2_** | **Mean Ct** | **RE** | **NOR** |
| PBS1 | 22.178 | 21.728 | 21.953 | 29.844 | 30.122 | 29.983 | 0.003826 | 1.851250 |
| PBS2 | 21.272 | 21.795 | 21.534 | 30.505 | 30.399 | 30.452 | 0.002067 | 1.000000 |
| PBS3 | 21.591 | 21.543 | 21.567 | 30.002 | 29.981 | 29.992 | 0.002911 | 1.408344 |
| PBS4 | 21.793 | 22.382 | 22.088 | 30.628 | 30.751 | 30.690 | 0.002574 | 1.245306 |
| I3A1 | 23.925 | 23.560 | 23.743 | 31.655 | 31.655 | 31.655 | 0.004150 | 2.008335 |
| I3A2 | 20.693 | 20.645 | 20.669 | 29.795 | 29.108 | 29.452 | 0.002271 | 1.098854 |
| I3A3 | 20.400 | 20.287 | 20.344 | 28.091 | 28.595 | 28.343 | 0.003908 | 1.890804 |
| I3A4 | 22.730 | 22.850 | 22.790 | 29.694 | 32.197 | 30.946 | 0.003507 | 1.697016 |

**Figure S17L**

|  | **β-actin (reference gene)** | | | **Cyp1a1 (target gene)** | | |  |  |
| --- | --- | --- | --- | --- | --- | --- | --- | --- |
|  | **Ct_1_** | **Ct_2_** | **Mean Ct** | **Ct_1_** | **Ct_2_** | **Mean Ct** | **RE** | **NOR** |
| PBS1 | 22.178 | 21.728 | 21.953 | 28.490 | 28.412 | 28.451 | 0.011064 | 1.000000 |
| PBS2 | 21.272 | 21.795 | 21.534 | 27.346 | 27.346 | 27.346 | 0.017794 | 1.608259 |
| PBS3 | 21.591 | 21.543 | 21.567 | 27.396 | 27.950 | 27.673 | 0.014518 | 1.312211 |
| PBS4 | 21.793 | 22.382 | 22.088 | 27.850 | 27.870 | 27.860 | 0.018294 | 1.653474 |
| I3A1 | 23.925 | 23.560 | 23.743 | 28.220 | 28.220 | 28.220 | 0.044889 | 4.057244 |
| I3A2 | 20.693 | 20.645 | 20.669 | 25.516 | 25.550 | 25.533 | 0.034339 | 3.103723 |
| I3A3 | 20.400 | 20.287 | 20.344 | 25.419 | 25.419 | 25.419 | 0.029657 | 2.680496 |
| I3A4 | 22.730 | 22.850 | 22.790 | 27.850 | 27.870 | 27.860 | 0.029770 | 2.690734 |

**Figure S19I**

|  | **β-actin (reference gene)** | | | **Ahr (target gene)** | | |  |  |
| --- | --- | --- | --- | --- | --- | --- | --- | --- |
|  | **Ct_1_** | **Ct_2_** | **Mean Ct** | **Ct_1_** | **Ct_2_** | **Mean Ct** | **RE** | **NOR** |
| DMSO1 | 22.245 | 21.972 | 22.109 | 27.253 | 26.006 | 26.629 | 0.043567 | 39.415017 |
| DMSO2 | 22.933 | 22.599 | 22.766 | 27.019 | 26.313 | 26.666 | 0.067006 | 60.619859 |
| DMSO3 | 20.249 | 20.419 | 20.334 | 25.622 | 24.923 | 25.272 | 0.032616 | 29.507561 |
| DMSO4 | 21.710 | 21.674 | 21.692 | 26.403 | 25.850 | 26.127 | 0.046244 | 41.836884 |
| CH-223191-1 | 20.655 | 20.852 | 20.754 | 30.435 | 30.715 | 30.575 | 0.001105 | 1.000000 |
| CH-223191-2 | 21.509 | 21.552 | 21.531 | 30.816 | 30.424 | 30.620 | 0.001836 | 1.660855 |
| CH-223191-3 | 21.886 | 21.845 | 21.866 | 28.961 | 28.226 | 28.593 | 0.009436 | 8.536514 |
| CH-223191-4 | 21.653 | 21.823 | 21.738 | 29.651 | 30.158 | 29.904 | 0.003480 | 3.148255 |

**Figure S20I**

|  | **β-actin (reference gene)** | | | **Il18 (target gene)** | | |  |  |
| --- | --- | --- | --- | --- | --- | --- | --- | --- |
|  | **Ct_1_** | **Ct_2_** | **Mean Ct** | **Ct_1_** | **Ct_2_** | **Mean Ct** | **RE** | **NOR** |
| CKO1 | 23.515 | 23.914 | 23.714 | 29.934 | 30.513 | 30.224 | 0.010978 | 1.946657 |
| CKO2 | 22.434 | 22.564 | 22.499 | 29.374 | 29.108 | 29.241 | 0.009344 | 1.656821 |
| CKO3 | 22.807 | 23.111 | 22.959 | 30.605 | 30.254 | 30.429 | 0.005639 | 1.000000 |
| CKO4 | 22.515 | 22.334 | 22.424 | 29.662 | 28.949 | 29.305 | 0.008483 | 1.504177 |
| CKO+I3A1 | 20.328 | 20.973 | 20.651 | 26.013 | 26.351 | 26.182 | 0.021622 | 3.834005 |
| CKO+I3A2 | 18.480 | 18.857 | 18.668 | 23.405 | 23.831 | 23.618 | 0.032359 | 5.737984 |
| CKO+I3A3 | 20.241 | 20.130 | 20.185 | 25.996 | 25.542 | 25.769 | 0.020848 | 3.696888 |
| CKO+I3A4 | 20.661 | 20.494 | 20.577 | 26.121 | 26.131 | 26.126 | 0.021372 | 3.789638 |
| FLOX+I3A1 | 20.202 | 19.992 | 20.097 | 24.182 | 23.755 | 23.969 | 0.068322 | 12.115016 |
| FLOX+I3A2 | 20.541 | 19.703 | 20.122 | 24.602 | 23.982 | 24.292 | 0.055553 | 9.850687 |
| FLOX+I3A3 | 20.227 | 20.496 | 20.362 | 24.330 | 25.060 | 24.695 | 0.049611 | 8.797139 |
| FLOX+I3A4 | 20.708 | 20.978 | 20.843 | 25.378 | 25.308 | 25.343 | 0.044198 | 7.837296 |

**Figure S20J**

|  | **β-actin (reference gene)** | | | **Ahr (target gene)** | | |  |  |
| --- | --- | --- | --- | --- | --- | --- | --- | --- |
|  | **Ct_1_** | **Ct_2_** | **Mean Ct** | **Ct_1_** | **Ct_2_** | **Mean Ct** | **RE** | **NOR** |
| CKO1 | 18.916 | 18.840 | 18.878 | 27.610 | 27.600 | 27.605 | 0.002360 | 1.098093 |
| CKO2 | 19.181 | 19.068 | 19.125 | 27.912 | 27.912 | 27.912 | 0.002263 | 1.052996 |
| CKO3 | 19.040 | 18.670 | 18.855 | 27.303 | 28.065 | 27.684 | 0.002199 | 1.023137 |
| CKO4 | 22.010 | 21.958 | 21.984 | 29.823 | 29.778 | 29.801 | 0.004436 | 2.064082 |
| CKO+I3A1 | 18.554 | 18.512 | 18.533 | 26.653 | 26.653 | 26.653 | 0.003594 | 1.672493 |
| CKO+I3A2 | 21.687 | 21.040 | 21.364 | 29.685 | 29.195 | 29.440 | 0.003705 | 1.723690 |
| CKO+I3A3 | 19.971 | 19.247 | 19.609 | 27.269 | 27.269 | 27.269 | 0.004944 | 2.300584 |
| CKO+I3A4 | 18.281 | 18.741 | 18.511 | 26.713 | 26.568 | 26.641 | 0.003571 | 1.661516 |
| FLOX+I3A1 | 21.472 | 21.782 | 21.627 | 30.489 | 30.489 | 30.489 | 0.002149 | 1.000000 |
| FLOX+I3A2 | 21.723 | 21.723 | 21.723 | 29.962 | 29.906 | 29.934 | 0.003375 | 1.570256 |
| FLOX+I3A3 | 21.590 | 21.590 | 21.590 | 28.393 | 28.996 | 28.695 | 0.007267 | 3.381117 |
| FLOX+I3A4 | 22.070 | 22.070 | 22.070 | 30.478 | 30.478 | 30.478 | 0.002944 | 1.369833 |

**Figure S20K**

|  | **β-actin (reference gene)** | | | **Cyp1a1 (target gene)** | | |  |  |
| --- | --- | --- | --- | --- | --- | --- | --- | --- |
|  | **Ct_1_** | **Ct_2_** | **Mean Ct** | **Ct_1_** | **Ct_2_** | **Mean Ct** | **RE** | **NOR** |
| CKO1 | 18.916 | 18.840 | 18.878 | 25.769 | 26.028 | 25.899 | 0.007702 | 3.324183 |
| CKO2 | 19.181 | 19.068 | 19.125 | 27.878 | 27.878 | 27.878 | 0.002317 | 1.000000 |
| CKO3 | 19.040 | 18.670 | 18.855 | 26.244 | 26.310 | 26.277 | 0.005831 | 2.516642 |
| CKO4 | 22.010 | 21.958 | 21.984 | 30.371 | 30.371 | 30.371 | 0.002987 | 1.289221 |
| CKO+I3A1 | 18.554 | 18.512 | 18.533 | 23.686 | 24.290 | 23.988 | 0.022797 | 9.838920 |
| CKO+I3A2 | 21.687 | 21.040 | 21.364 | 27.610 | 27.600 | 27.605 | 0.013217 | 5.704103 |
| CKO+I3A3 | 19.971 | 19.247 | 19.609 | 25.369 | 25.369 | 25.369 | 0.018453 | 3.164549 |
| CKO+I3A4 | 18.281 | 18.741 | 18.511 | 24.400 | 24.100 | 24.250 | 0.018724 | 8.080810 |
| FLOX+I3A1 | 21.472 | 21.782 | 21.627 | 25.850 | 26.170 | 26.010 | 0.047928 | 20.684813 |
| FLOX+I3A2 | 21.723 | 21.723 | 21.723 | 26.868 | 27.023 | 26.946 | 0.026784 | 11.559443 |
| FLOX+I3A3 | 21.590 | 21.590 | 21.590 | 26.396 | 25.950 | 26.173 | 0.041723 | 18.007175 |
| FLOX+I3A4 | 22.070 | 22.070 | 22.070 | 26.952 | 26.909 | 26.931 | 0.034423 | 14.856270 |
